# Uncovering the anti-cancer mechanism of cucurbitacin D against colorectal cancer through network pharmacology and molecular docking

**DOI:** 10.1007/s12672-025-02056-7

**Published:** 2025-04-17

**Authors:** Hae-In Lim, Ga Yoon Kim, Yu-Jeong Choi, Kangwook Lee, Seong-Gyu Ko

**Affiliations:** 1 Department of Science in Korean Medicine, Graduate School, Kyung Hee University, Seoul, 02447 Republic of Korea; 2 College of Pharmacy, Natural Products Research Institute, Seoul National University, Seoul, 08826 Republic of Korea; 3 Department of Food and Biotechnology, Korea University, Sejong, 30019 Republic of Korea; 4 Department of Preventive Medicine, College of Korean Medicine, Kyung Hee University, Seoul, 02447 Republic of Korea

**Keywords:** Colorectal cancer, Cucurbitacin D, Network pharmacology, Gene ontology, KEGG, Molecular docking

## Abstract

Colorectal cancer is a significant global health challenge due to chemoresistance, necessitating new treatments. Cucurbitacin D, with its anti-cancer properties, shows promise, but its effects on colorectal cancer are not well understood. We investigated the impact of cucurbitacin D on colorectal cancer cell lines using MTT assays and Annexin V/7-AAD staining followed by flow cytometry for apoptosis analysis. Public databases helped identify cucurbitacin D and colorectal cancer-related gene targets for network pharmacology analysis. Protein–protein interaction networks were constructed using STRING and analyzed in Cytoscape. Gene ontology and KEGG pathway enrichment analyses were performed using ClueGo. Molecular docking studies were conducted via Autodock Vina and visualized in Discovery Studio. Western blot assessed protein expression changes in key targets under cucurbitacin D. Cucurbitacin D dose-dependently reduced colorectal cancer cell viability and induced apoptosis. Network pharmacology pinpointed crucial targets like *STAT3*, *AKT1*, *CCND1*, and *CASP3*. Molecular docking confirmed strong interactions with these targets. Enrichment analysis highlighted involvement in the 'PI3K-AKT,' 'JAK-STAT,' and 'ErbB' signaling pathways. These findings suggest cucurbitacin D as a potential anti-colorectal cancer agent, demonstrating significant effects on cell viability and apoptosis, and engaging critical cancer-related pathways, making it a promising candidate for further colorectal cancer therapeutic research.

**Supplementary Information:**

The online version contains supplementary material available at 10.1007/s12672-025-02056-7.

## Introduction

Colorectal cancer (CRC) is one of the most common cancers worldwide, and despite advancements in screening and treatment, it remains a significant cause of morbidity and mortality [1–3]. The current therapeutic options for CRC include surgery, chemotherapy, and radiation therapy [4, 5]. However, there are limitations to these treatments including the development of chemoresistance and severe adverse effects [6–8] that the development of new drugs may offer new options for treating CRC.

Cucurbitacin D (CuD), also known as elatericin, is a naturally occurring dietary compound found in certain plants, including some members of the cucurbitaceae family, such as cucumber, pumpkin, watermelon, Trichosanthes kirilowii Maximowicz and Picrorhizae Rhizoma [9–13]. Preclinical studies have reported the possible health-beneficial effects of CuD such as anti-HIV [14], anti-SARS-CoV-2 [15], anti-inflammation [16] and anti-diabetes [17]. Meanwhile, the anti-cancer effect of CuD has been widely researched, as it has been found to inhibit tumor cell growth, migration, invasion and metastasis in various types of cancer including gastric cancer [18, 19], prostate cancer [20], breast cancer [21], lung cancer [22–24], pancreatic cancer [25, 26], T-cell leukemia [27–29], ovarian cancer, endometrial cancer [30], hepatocellular carcinoma [13, 21] and melanoma [31]. In addition, CuD also exhibits analgesic effect in paclitaxel-induced neuropathic pain in mice [32, 33]. In CRC, CuD inhibits cell viability of CRC cell lines HT-29 and SW-480 [34]. Thus, CuD could be the potential novel drug candidate to treat cancer patients whereas more studies about its anti-CRC effect and related molecular mechanisms are still unclear.

In recent years, network pharmacology has emerged as a powerful tool for investigating how drugs interact with multiple targets and pathways within biological networks. This integrative approach aims to understand the mechanisms of action of drugs at a system-level, by analyzing their interactions with multiple targets and pathways in the context of biological networks and to provide insights into the underlying molecular mechanisms of their therapeutic effects [35–38]. Recent studies using network pharmacology have been applied to investigate compounds with multitargets and multi pathways with improved efficacy and reduced toxicity in CRC [39–42]. Since compounds derived from natural products interact with multiple targets, not single target, to modulate complex disease pathways, understanding multitargets of active compounds has the potential to address the heterogeneity and complexity of CRC and overcome the limitations of single-target drugs or conventional cytotoxic drugs, which include drug resistance and side effects [35, 37, 43, 44]. Thus, the present study conducted network pharmacology to identify multitargets and multi pathways related to the anti-cancer efficacy of CuD on CRC.

## Materials and methods

### Chemicals and reagents

RPMI1640 medium, penicillin/streptomycin, trypsin–EDTA and DPBS were obtained from WelGENE (WelGENE Inc., Daegu, South Korea). Fetal bovine serum (FBS) was purchased from JR Scientific (Woodland, CA, USA). CuD was obtained from Extrasynthese (Genay, France), dissolved in dimethyl sulfoxide (Sigma-Aldrich, St. Louis, MO, USA) and stored in aliquots at −20 °C until use. 3-(4,5-dimethylthiazol-2-yl)−2,5-diphenyltetrazolium bromide (MTT) was purchased from Sigma-Aldrich (St. Louis, MO, USA). Annexin V and 7-aminoactinomycin D (7-AAD) were obtained from BD Biosciences (Franklin Lakes, NJ, USA). For western blot, ice-cold radioimmunoprecipitation assay buffer (R2002, Biosesang, Seongnam, South Korea), bovine serum albumin (Sigma-Aldrich, St. Louis, MO, USA), Bradford solution (#5,000,006, Bio-Rad, Hercules, CA, USA), PVDF membrane (IPVH00010, Merck Millipore Ltd., MA, USA), tween-20 (Sigma-Aldrich, St. Louis, MO, USA), primary antibodies including anti-STAT3 (1:1,000, #4904, Cell Signaling), anti-phospho-STAT3 (1:1,000, #9145, Cell Signaling), anti-AKT (1:1,000, #9272, Cell Signaling), anti-phospho-AKT (1:1,000, #9271, Cell Signaling), anti-Cyclin D1 (1:1,000), anti-cleaved caspase-3 (1:1,000) and anti-alpha-tubulin (1:3,000, #3873, Cell Signaling), secondary IgG antibodies (Cell Signaling, MA, USA) and Immobilon Western chemiluminescent HRP substrate (WBKLS0500, Merck Millipore Ltd., MA, USA) were purchased.

### Cell culture

We purchased human CRC cell line, HCT-8, HCT-15, HT-29 and DLD1 from American Type Culture Collection (ATCC, Rochville, MD, USA). Cell lines were cultured in RPMI1640 medium supplemented with 10% FBS and 1% penicillin/streptomycin and were maintained at 37 °C in a humidified atmosphere with 5% CO2.

### Cell viability assay

Cell lines were seeded into 96 wells plate at a density of 5 × 10^3^ cells/well and were treated with CuD (0.05, 0.5, 5 µM) for 24 h. At the end of treatment, cells were further incubated with MTT working solution (0.5 mg/mL in complete medium) for 1 h. The MTT formazan was dissolved in dimethyl sulfoxide and the absorbance was measured at 570 nm by a spectrophotometer (Molecular Devises, CA, USA). The cells were photographed with an inverted phase-contrast microscope.

### Annexin V/7-aminoactinomycin D apoptosis assay

Apoptotic cell death was determined by an Annexin-V and 7-AAD double staining. CRC cell lines were treated with desired concentrations of CuD and then were detached by incubating with 0.25% trypsin–EDTA. The pellet was incubated with annexin-V for 15 min, followed by further incubation with 7-AAD for 15 min. Both incubations were performed in the dark at 4 °C. The percentage of viable, early apoptotic and late apoptotic cells was ana-lyzed by FACSCalibur flow cytometer instrument configured with BD CellQuest Pro software (BD Bioscience, CA, USA).

### Western blot

To extract whole proteins from cell lines, we used ice-cold radioimmunoprecipitation assay buffer supplemented with protease and phosphatase inhibitors. The protein concentration was determined using the Bradford assay, and equal amounts of protein were separated by 10–12% SDS-PAGE. The separated proteins were transferred onto a PVDF membrane and blocked with 5% bovine serum albumin in tris-buffered saline with Tween-20 (TBST). The membrane was then incubated with primary antibodies at 4 °C for overnight. After washing with TBST, the membrane was incubated with secondary antibodies at room temperature for 1 h. To detect the horseradish peroxidase signal, we used Immobilon Western chemiluminescent HRP substrate.

### Identification of targets for CuD and CRC

Investigating the polypharmacological properties of active biomolecules is important for developing novel drug candidates with improved therapeutic outcomes [37, 38]. Molecular targets of CuD were collected from SwissTargetPrediction (http://swisstargetprediction.ch) [45] PharmMapper (http://lilab-ecust.cn/pharmmapper) [46] and PubChem (https://pubchem.ncbi.nlm.nih.gov/) [47]. CRC-associated genes were integrated from DisGeNET (http://www.disgenet.org) [48] and GeneCards (http://www.genecards.org) [49] database by using keywords ‘colorectal cancer’ with the species limited to ‘Homo sapiens’. The integrated genes were standardized by UniProt database [50].

### Network construction

Overlapping targets between CuD and CRC were determined as the potential anti-CRC targets of CuD. CuD-target network and CuD-anti-CRC target network were constructed. The protein–protein interaction (PPI) network was constructed based on the interactions between anti-CRC targets using the Search Toll for the Retrieval of Interacting Genes/Proteins (STRING 11.5, http://string-db.org) [51]. The minimum score was set as the high confidence score > 0.7 and species were limited to ‘Homo sapiens’. The STRING results were imported into Cytoscape to analysis the PPI network’s topology properties [52]. The topology analysis of the constructed PPI network was performed and the hub targets in this network were identified based on their degree, betweenness centrality and closeness centrality.

### Gene ontology and KEGG pathway enrichment analysis

Gene ontology (GO) functional enrichment analysis and KEGG pathway enrichment analysis for the candidate targets were carried out using ClueGo of Cytoscape [53]. The enrichment results of GO including biological process (BP), molecular function (MF), and cellular component (CC) and KEGG pathways with p value < 0.001 were selected. For the KEGG pathway enrichment analysis, we utilized the ClueGo plug-in in Cytoscape. In ClueGo, three key parameters (Gene Ratio, Count, and p-value) describe pathway enrichment. GeneRatio refers to the ratio of the number of genes associated with a specific pathway to the total number of genes analyzed. Count represents the absolute number of genes linked to that pathway, and the p-value, corrected with the Benjamini–Hochberg method, indicates the statistical significance of the enrichment for the pathway. These values provide both relative and absolute perspectives on the number of genes involved in each pathway. Since these parameters are based on different calculations, they do not always show a direct correlation in the graphical representation.

### Molecular docking analysis

The top 4 targets with the highest “degree” values, STAT3, AKT1, cyclin D1 and caspase-3, were defined. The crystal structures of candidates and SDF formats of active compounds were obtained from the RCSB PDB database (https://www.rcsb.org/) and the PubChem database (https://pubchem.ncbi.nlm.nih.gov/), respectively. The heteroatoms of proteins including small molecules, ions and water were removed and hydrogen atoms were added by using Discovery Studio program. To predict the binding activities of STAT3 (PDB ID: 6NJS), AKT1 (PDB ID: 3MVH), cyclin D1 (PDB ID: 2W96) and caspase-3 (PDB ID: 2J30 to CuD, the molecular docking was performed using Autodock Vina in PyRx 0.8 [54]. A putative binding sites of each protein was determined according to previously published results [55–58]. The grid box was centered to cover the desired domain and all residues. The lower binding energy (kcal/mol) between the ligand and the receptor indicates more stable binding. The docking results were visualized by Discovery Studio program.

### Statistical analysis

The enrichment analysis employed a statistical test based on the Benjamini–Hochberg method for multiple test correction, and only terms and pathways with a p-value less than 0.001 were deemed statistically significant. The Shapiro–Wilk test was used to assess whether the data followed a normal distribution. When the data did not conform to normality, the Mann–Whitney test was applied. For data that were normally distributed, the two-tailed unpaired Student's t-test with Welch’s correction was used. Data are shown as mean ± standard error mean (SEM). Statistical significance was determined by p-values less than 0.05.

## Results

### Inhibition of viability and induction of apoptosis by CuD in CRC cell lines

**Fig. 1:**
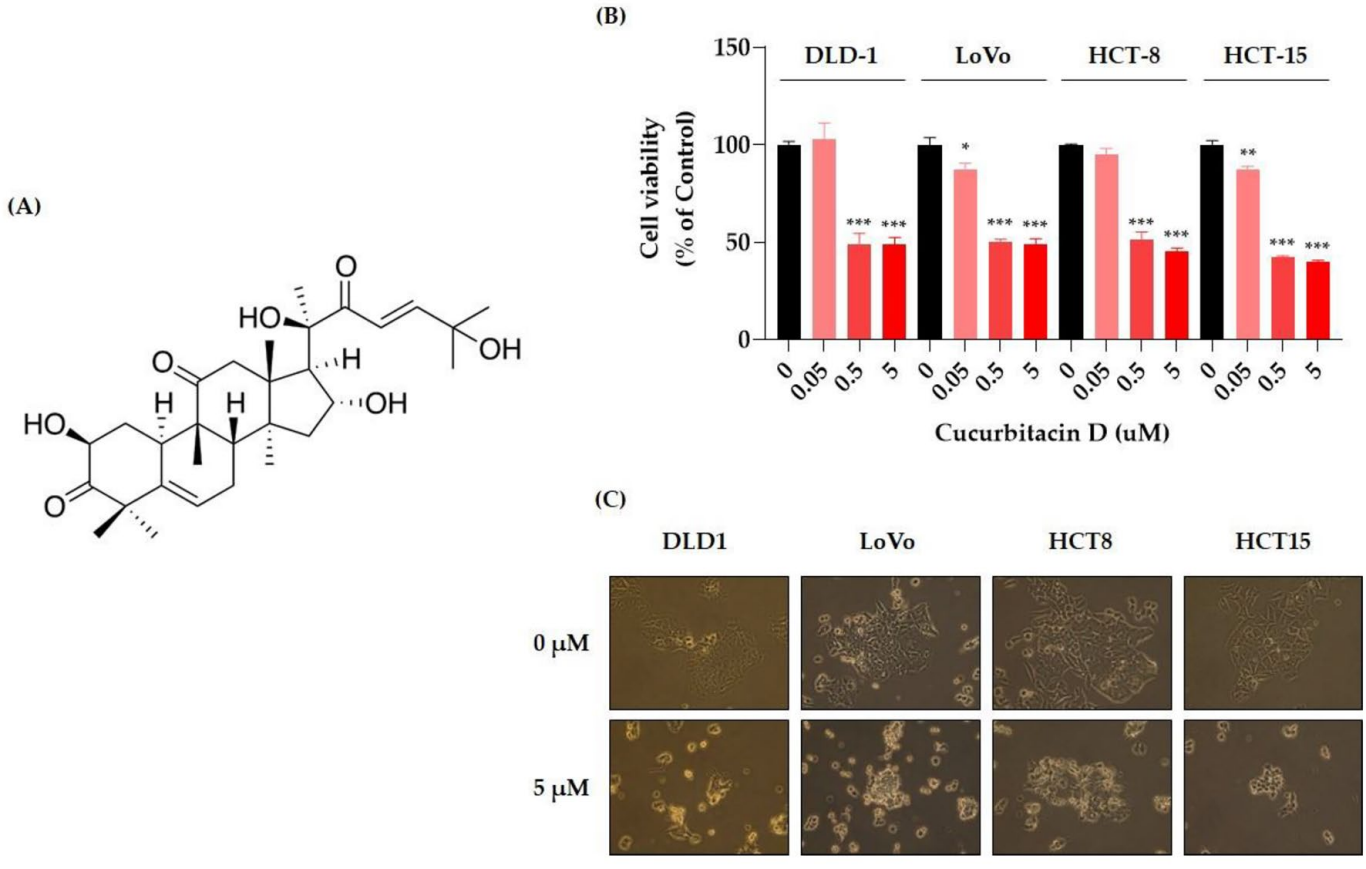
Cucurbitacin D (CuD) inhibits the cell viability of Colorectal cancer (CRC) cell lines. **A** 2D Structure depiction of CuD, **B** CRC cell lines—DLD-1, LoVo, HCT-8, and HCT-15—were treated with the indicated doses of CuD for 24 h, and cell viability was measured using the MTT assay. (0: Control, cells treated with 0.1% DMSO at the final concentration as vehicle; CuD: cells were treated with CuD at final concentrations of 0.05, 0.5, or 5 μM)." **C** Representative images of untreated and 5 μM CuD-treated CRC cells after 24 h, highlighting morphological changes indicating cell death. The Shapiro–Wilk test was utilized to assess the normality of the data. Differences were analyzed using an unpaired two-tailed Student's t-test with Welch's correction. The data are presented as means ± standard errors of the mean (SEM), based on three independent experiments. *p < 0.05, **p < 0.01, and ***p < 0.001 (vs. “0” for each cell lines)

**Fig. 2:**
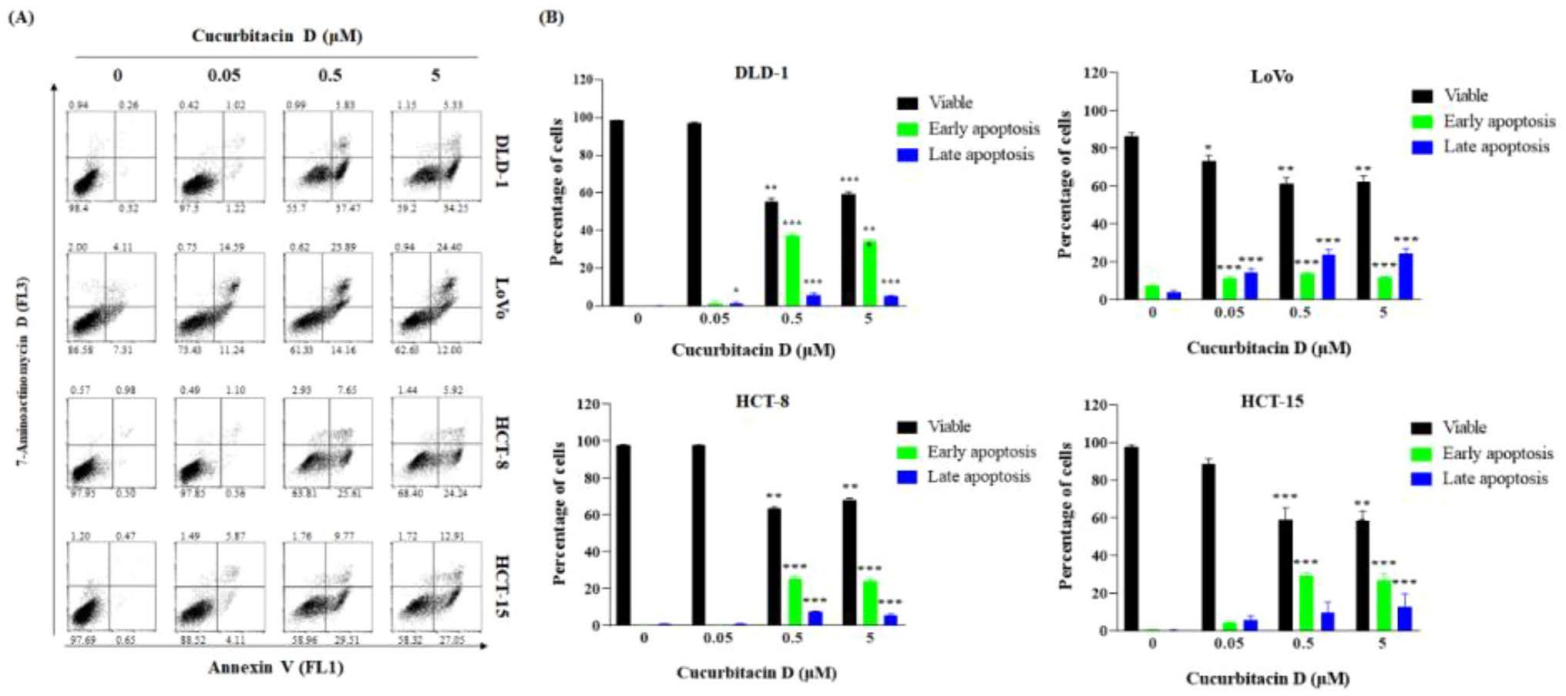
CuD treatment induces apoptotic cell death of CRC cell lines. **A** Representative flow cytometry scatter plots showing cells undergoing apoptosis in response to CuD. Percentages corresponding to the four quadrants have been added to indicate viable cells, early apoptotic cells, late apoptotic cells, and necrotic cells. **B** Bar graph shows percentage of DLD-1, LoVo, HCT-8, and HCT-15 cells experiencing apoptosis after exposure to 0.05, 0.5, and 5 μM CuD for 24 h. The Shapiro–Wilk test was utilized to assess the normality of the data. Differences were analyzed using an unpaired two-tailed Student's t-test with Welch's correction. The data are presented as means ± standard errors of the mean (SEM), based on three independent experiments

We evaluated the anticancer potential of CuD in CRC cell lines using an MTT cell viability assay. CuD's chemical structure is depicted in Fig. 1A. As shown in Fig. 1B, CuD treatment led to a dose-dependent decrease in cell viability in CRC cell lines. Figure 1C shows representative images of cells treated with 5 μM CuD for 24 h. Colorectal cancer cells, when untreated, displayed a polygonal shape with a uniform size distribution. In contrast, CuD-treated cells exhibited noticeable morphological changes, including cell shrinkage, membrane blebbing, and an increased number of detached, round-shaped cells, indicating signs of cell death. Furthermore, the annexin V/7-AAD double staining apoptosis assay demonstrated an elevated population in both early (lower right quadrant) and late apoptosis (upper right quadrant) in CRC cell lines following CuD treatment (Fig. 2). These findings underscore CuD's robust potential in fighting CRC by inducing apoptotic cell death.

### Screening of potential targets of CuD in CRC

**Fig. 3:**
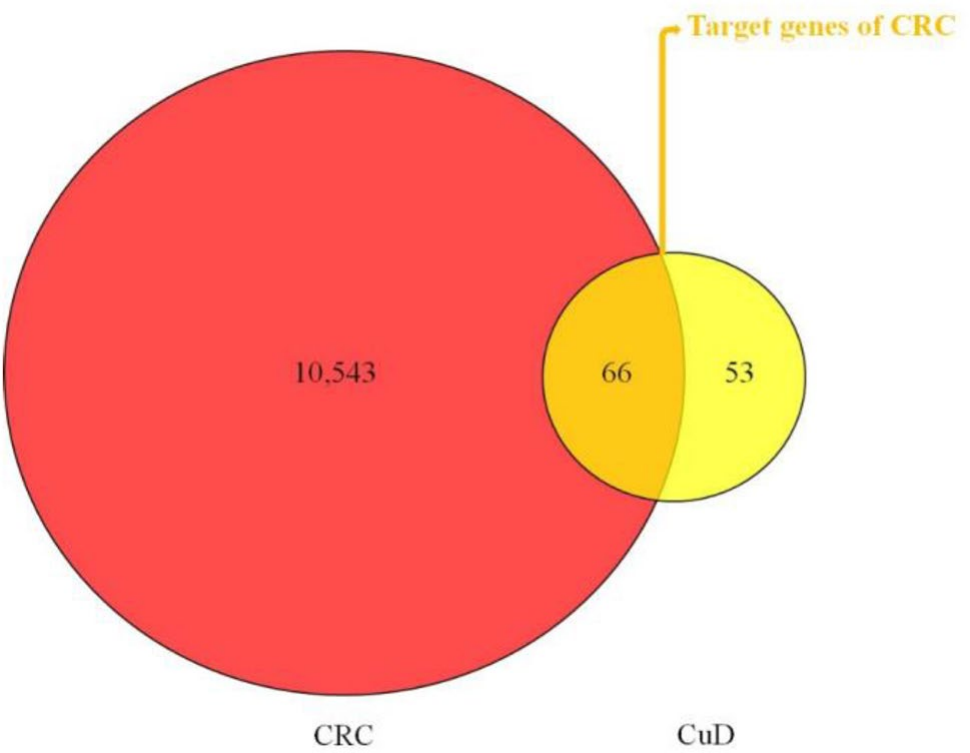
Venn diagram illustrating potential anti-CRC targets of CuD

Targets of CuD was collected from SwissTargetPrediction, PharmMapper and PubChem database. After deleting the duplicates, a total of 119 targets of CuD were found (Table S1). Next, CRC-related genes were gathered from DisGeNet and GeneCards database. After deleting the duplicates, 10,608 genes of CRC were identified (Table S2). The 66 intersecting common genes between targets of CuD and CRC-related genes were obtained as the potential anti-CRC targets of CuD (depicted in Fig. 3).

### Network construction and PPI network analysis

CuD-target network and CuD-anti-CRC target network were constructed. The network analysis results obtained using Cytoscape provided insights into the mechanism of action of CuD against CRC. The C-T network contained an compound and 119 targets. Further, we constructed a C-T network by linking the CuD and CRC-related 66 targets (Figure S1A and S1B). Constructed network showed the potential multitarget properties of CuD as an anti-CRC agent.

**Fig. 4:**
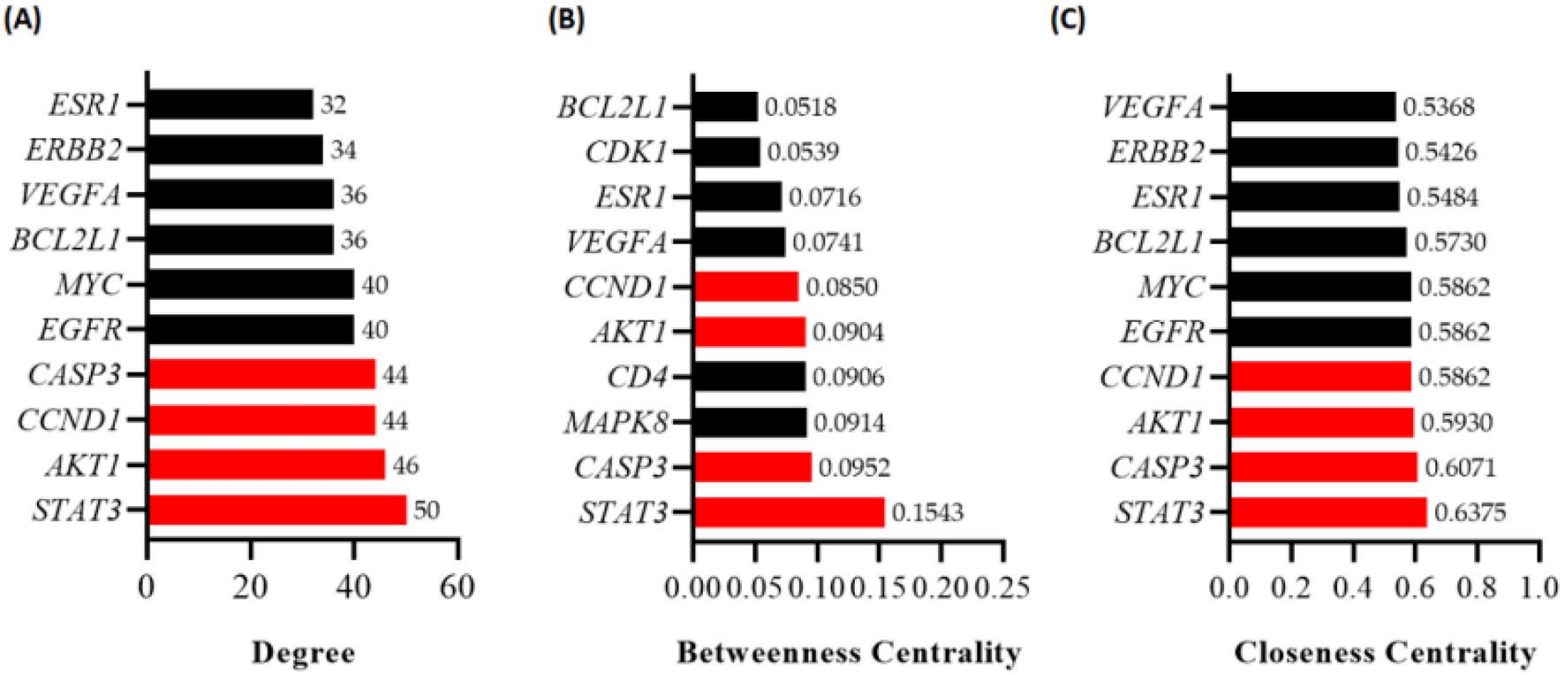
Protein–protein interaction (PPI) network analysis and identification of key targets for CuD in CRC. **A**–**C** The Degree, Betweenness Centrality, Closeness Centrality values of the identified targets in the PPI network was determined using Cytoscape software. Bar chart highlights the top 10 targets from each type of topological analysis. The bars colored red in the chart represents the core targets identified across all three centrality analyses

A total of 66 target genes were analyzed using STRING, with a high confidence score (> 0.7) and restricted to the 'Homo sapiens' species. The PPI network generated by the STRING analysis (Figure S2). Utilizing Cytoscape software, we analyzed the interaction network among these 66 targets, determining node values based on Degree, Betweenness Centrality, and Closeness Centrality. The outcomes, depicted in Fig. 4 (A-C), rank these targets according to their Degree, Betweenness Centrality, and Closeness Centrality values. Notably, STAT3, AKT1, CCND1, and CASP3 emerged as the top four key targets, possessing elevated degree values and securing positions within the top ten rankings for both Betweenness Centrality and Closeness Centrality algorithms.

### GO and KEGG enrichment analysis

**Fig. 5:**
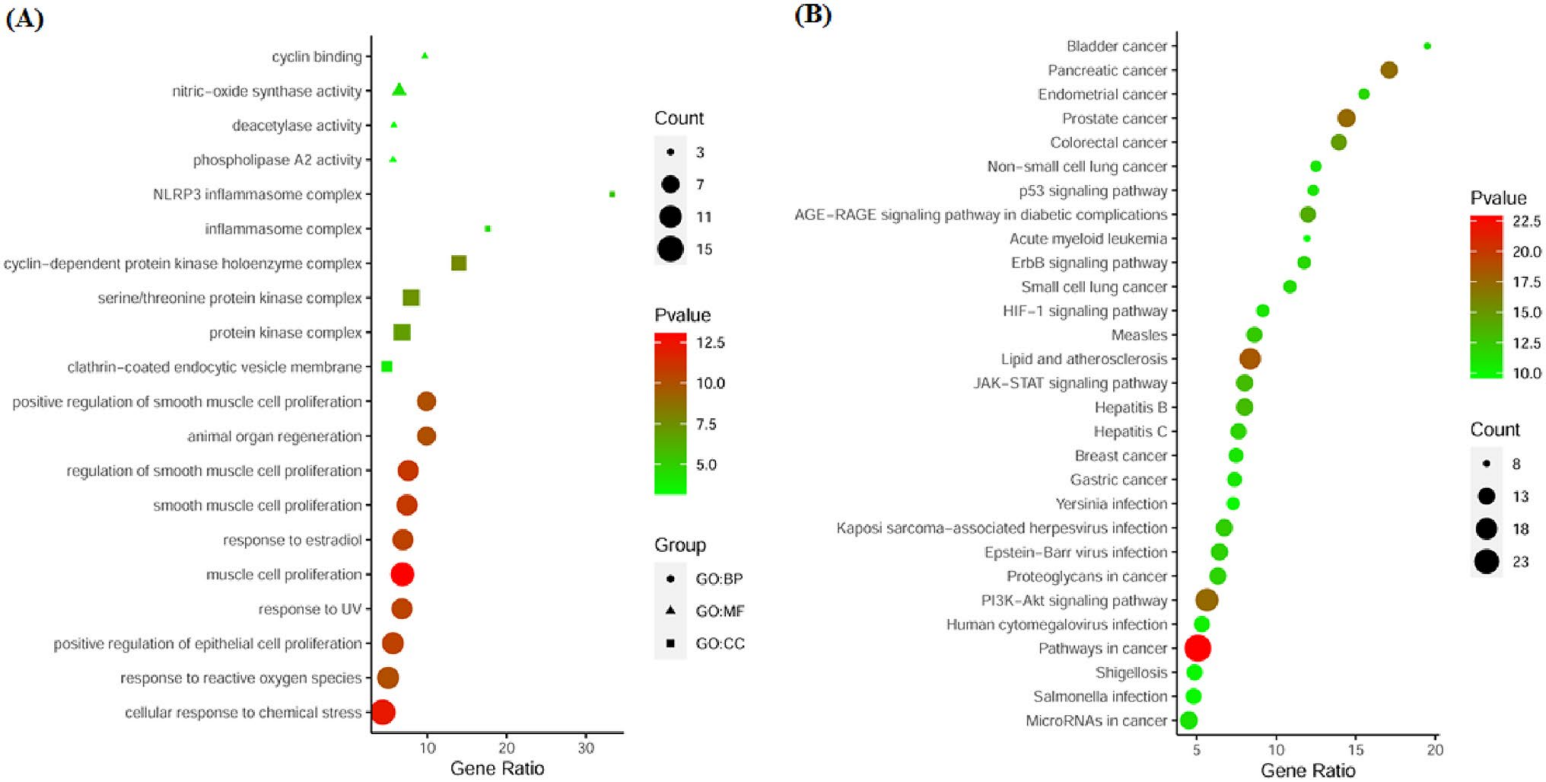
The GO and KEGG pathway analysis. **A** Biological process (GO), **B** KEGG pathway analysis. The Y-axis represents the enriched GO terms and KEGG pathways, while the X-axis denotes the GeneRatio, representing the percentage of genes associated with each GO or KEGG term. The size of the dots indicates the Count (number of genes associated with each term), and the color of the dots corresponds to the -Log10(p-value), corrected using the Benjamini–Hochberg method for multiple comparisons

To gain comprehensive insights into the biological functions of the CRC-related targets of CuD, we performed GO analysis that incorporated the analysis of BP, MF and CC. The analysis was conducted with a statistical significance threshold of p < 0.001. The top ten enriched results for BP, MF, and CC are presented in Fig. 5A and Table S3. The BP results included ‘muscle cell proliferation’, ‘cellular response to chemical stress’, ‘regulation of smooth muscle cell proliferation’, ‘smooth muscle cell proliferation’, ‘positive regulation of epithelial cell proliferation’, ‘response to estradiol’ and so on. The MF results contained ‘nitricoxide synthase activity’, ‘cyclin binding’, ‘deacetylase activity’ and ‘phospholipase A2 activity’. The CC results included ‘cyclin-dependent protein kinase holoenzyme complex’, ‘serine/threonine protein kinase complex’, ‘protein kinase complex’, ‘NLRP3 inflammasome complex’, ‘inflammasome complex’ and ‘clathrincoated endocytic vesicle membrane’. The KEGG pathway enrichment analysis revealed that a total of 80 pathways were significantly correlated with CRC-related targets of CuD at a significance threshold of FDR < 0.001 (Table S3). Top 30 KEGG pathways were presented in Fig. 5B. The cancer-related KEGG pathways included ‘Pathways in cancer’, ‘Lipid and atherosclerosis’, ‘Prostate cancer’, ‘PI3K-AKT signaling pathway’, ‘Pancreatic cancer’, ‘Colorectal cancer’, ‘JAK-STAT signaling pathway’, ‘Proteoglycans in cancer’, ‘ErbB signaling pathway’, etc. Together, the results of both GO and KEGG analyses collectively suggest that CuD has the potential to exhibit anti-CRC activity from a genetic perspective, possibly through multiple synergistic mechanisms.

### Molecular docking experiment

**Fig. 6:**
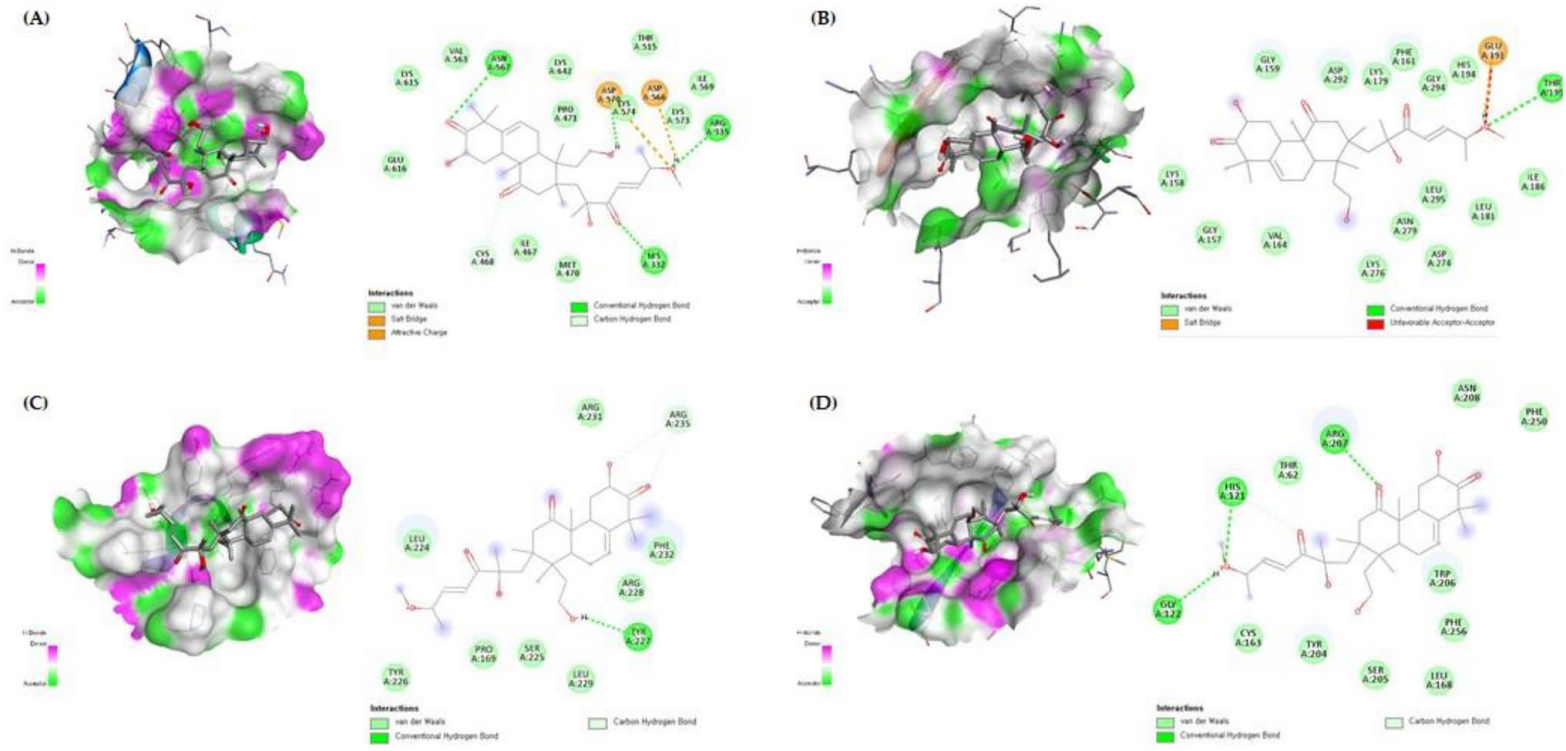
Molecular Docking Analysis of Cucurbitacin D with Target Proteins. Molecular docking of **A** STAT3 (PDB ID: 6NJS), **B** AKT1 (PDB ID: 3MVH), **C** cyclin D1 (PDB ID: 2W96) and **D** caspase-3 (PDB ID: 2J30) with Cucurbitacin D

The results of PPI analysis indicated that *STAT3*, *AKT1*, *CCND1* and *CASP3* are the hub targets of CuD in CRC (Fig. 5). Herein, we performed molecular docking on the CuD with STAT3, AKT, Cyclin D1 and Caspase-3 to reveal the potential binding affinity. Docking analysis showed that CuD can stably bind to four targets. Tables 1 and 2 give the detail about grid box and docking score, respectively. Figure 6 shows the 2D and 3D interaction diagrams of CuD in active sites of four targets with the lowest binding energy (Fig. 6).

**Table 1:** Grid box parameters

Targets	PDB ID	Grid box size			Grid center coordinate		
		x	x	y	x	y	z
STAT3	6NJS	37.193	44.328	41.753	3.410	55.679	1.204
AKT1	3MVH	30.133	30.166	30.103	18.369	−2.499	28.058
CYCLIN D1	2W96	60.584	59.952	60.042	11.430	8.978	41.081
CASPASE-3	2J30	23.088	17.835	19.430	37.981	10.511	67.466

**Table 2:** Binding energy of CuD

	Binding Energy (kcal/mol)			
	STAT3	AKT1	CYCLIN D1	CASPASE-3
Cucurbitacin D	−7.7	−8.8	−6.8	−7.7

### CuD’s impact on the top four hub targets was confirmed through western blot analysis

**Fig. 7:**
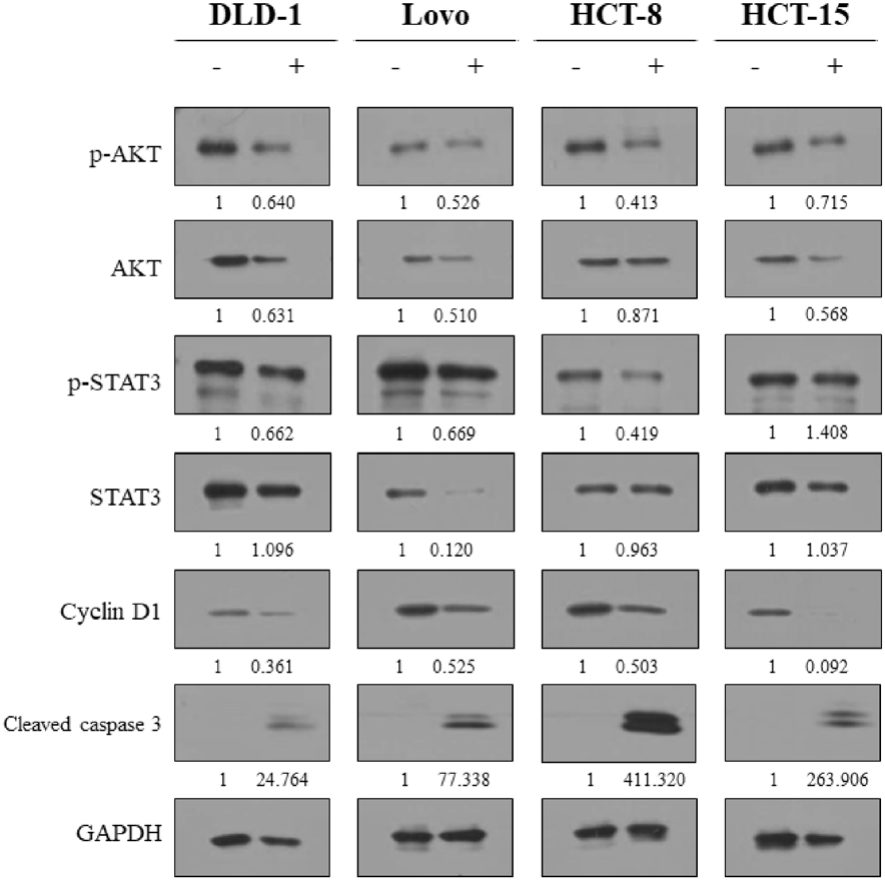
Western blot analysis for examining expression levels of targeted proteins in CRC cells treated with CuD. CRC cell lines were treated with the CuD (concentration of each IC 50; DLD-1: 4.476 μM, Lovo: 4.454 μM, HCT-8: 4.036 μM, HCT-15: 3.418 μM) for 24 h. Expression of p-AKT, AKT, p-STAT3, STAT3, Cyclin D1, Cleaved caspase 3 and GAPDH was determined by western blotting. Densitometry analysis was performed using ImageJ, and the protein expression levels were normalized to GAPDH. The ratios of protein expression in CuD-treated cells to the control group (untreated) are shown below each band. Original unprocessed Western blot images are provided in the supplementary materials

Treatment with CuD led to distinct alterations in the expression levels of the hub proteins, AKT, STAT3, Cyclin D1 and Cleaved caspase3 (Fig. 7). These changes in protein expression induced by CuD were overserved to be specific to the CRC cell lines, indicating a unique regulatory effect on these targets.

## Discussion

CRC is a pervasive global health concern associated with substantial morbidity and mortality. Existing therapeutic options are hampered by challenges such as chemoresistance and adverse effects, emphasizing the urgent need for innovative drug development. CuD, with its demonstrated anti-cancer effects in various malignancies, presents a promising candidate for cancer treatment, though its specific impact on CRC and the underlying mechanisms require further exploration.

Our analysis revealed that STAT3, AKT1, CCND1, and CASP3 is the hub targets in PPI network. The results of molecular docking study showed that CuD had good binding activities to these four key targets. STAT3 (Signal Transducer and Activator of Transcription 3) is a transcription factor that plays a crucial role in a variety of cellular processes, including cell proliferation, differentiation, and survival [59]. It is activated in response to various signals, including cytokines, growth factors, and oncogenic proteins, and has been implicated in the development and progression of several types of cancer, including CRC. In CRC, STAT3 is often constitutively activated, which contributes to the uncontrolled growth and survival of cancer cells. STAT3 activation has been linked to increased expression of genes involved in cell proliferation, angiogenesis, invasion, and immune evasion, and inhibition of STAT3 has been shown to decrease the growth and survival of CRC cells [60]. Several approaches have been explored for inhibiting STAT3 in the treatment of CRC, including small molecule inhibitors, peptides, and antibodies. Small molecule inhibitors that target STAT3 signaling, such as Static and S3I-201, have shown promise in preclinical studies [61] and have been shown to inhibit the growth of CRC cells. Peptide inhibitors that disrupt the interaction between STAT3 and other proteins have also been developed and tested in preclinical studies. Additionally, antibodies that target the extracellular domain of the IL-6 receptor, which is upstream of STAT3 activation, have been investigated in clinical trials for the treatment of CRC [62]. AKT1 (also known as protein kinase B) is a serine/threonine kinase that plays an important role in the regulation of cell survival, proliferation, and metabolism. AKT1 is frequently activated in CRC, and its activation has been associated with the development and progression of the disease (Activated AKT1 promotes cell survival and proliferation by regulating downstream targets involved in cell cycle regulation, apoptosis, and metabolism [63]. One of the downstream targets of AKT1 is Cyclin D1, which is a regulatory protein involved in the progression of cells through the cell cycle. Cyclin D1 is overexpressed in CRC, and its expression has been associated with poor clinical outcomes [64]. The overexpression of Cyclin D1 promotes cell cycle progression and cell proliferation, which contributes to the development and progression of CRC [65]. Several approaches have been explored for inhibiting AKT1 and Cyclin D1 in the treatment of CRC. Small molecule inhibitors that target AKT1, such as MK2206 and GSK2141795, have shown promise in preclinical studies and have been tested in clinical trials for the treatment of CRC [66, 67]. Additionally, inhibitors that target Cyclin D1, such as flavopiridol and dinaciclib, have also been tested in preclinical and clinical studies. These inhibitors can induce cell cycle arrest and inhibit cell proliferation and have been shown to be effective in reducing tumor growth in preclinical models of CRC [68]. Caspase-3, plays a pivotal role in apoptosis, regulating programmed cell death crucial for maintaining tissue balance and preventing cancerous growth. Dysregulation of Caspase-3 in cancer leads to the evasion of apoptosis, a hallmark of tumorigenesis [69]. A comprehensive understanding of its intricate involvement in cell death pathways is vital for developing precise cancer therapies. In CRC, inducing Caspase-3 cleavage through cytotoxic drug treatment initiates a series of events, resulting in selective cell demise and inhibiting migration, invasion, and metastasis of colorectal cancer cells. Therefore, targeting the four key components, STAT3, AKT1, CCND1, and CASP3, holds significant potential for CuD’s effectiveness in treating CRC.

The results of KEGG pathway enrichment analysis showed that anti-CRC effect of CuD is associated with diverse pathways. Among them, ‘PI3K-AKT signaling pathway’, ‘JAK-STAT signaling pathway’ and ‘ErbB signaling pathway’ are top three cancer-related pathways from our results. The PI3K-AKT signaling pathway is one of the key pathways in regulating cell survival and growth. Dysregulation of this pathway is commonly observed in many types of cancer, including CRC. In CRC, the PI3K-AKT pathway is activated by mutations in the PIK3CA, leading to increased proliferation and survival of cancer cells lines. The PI3K-AKT pathway also plays a pivotal role in regulating the expression of diverse genes involved in cell proliferation, apoptosis, and tumor angiogenesis [70]. The JAK-STAT signaling pathway is another important pathway involved in the development of CRC. This pathway is activated by cytokines and growth factors, which bind to cell surface receptors and activate Janus kinases (JAKs). Activated JAKs then phosphorylate and activate signal transducers and activators of transcription (STATs), leading to the transcription of genes involved in cell proliferation, differentiation, and survival. Dysregulation of the JAK-STAT pathway has been observed in many types of cancer, including CRC [70]. Accumulating studies have demonstrated that activation of the JAK-STAT pathway has been associated with increased proliferation and survival of CRC. The ErbB signaling pathway is a family of receptor tyrosine kinases involved in regulating cell proliferation, differentiation, and survival. Dysregulation of this pathway has been observed in many types of cancer, including CRC. Mutations in the EGFR gene and overexpression of ErbB2 have been observed in CRC, leading to the activation of downstream signaling pathways that promote cell proliferation, drug resistance and metastasis [71]. Therefore, targeting these pathways could be an attractive strategy for CRC treatment. The present study suggests that cucurbitacin D may exert its anti-cancer effect in CRC by targeting multiple pathways including ‘PI3K-Akt signaling pathway’, ‘JAK-STAT signaling pathway’, ‘ErbB signaling pathway’ and so on. Taken together, further research into the mechanisms that drive the anti-CRC activity of CuD could enable the development of novel treatments with multitarget properties for this malignancy. Establishing a firm understanding of the molecular pathways that contribute to the response to CuD treatment through further studies could provide a strong basis for the development of more effective interventions for CRC treatment.

CuD has shown promising anti-cancer properties in various studies. However, its therapeutic application requires careful consideration of its dosage and administration to avoid potential toxicity. While CuD has been observed to inhibit cancer cell proliferation at effective doses, further research is needed to fully assess its safety profile, particularly regarding its effects on normal tissues. In preclinical studies using animal models, it is essential to evaluate potential off-target effects and toxicity in normal tissues to determine the safe dosage range. The therapeutic window, which refers to the range between the effective dose and a potentially harmful dose, will be crucial in determining the clinical feasibility of CuD. In addition, due to its lipophilic nature and poor water solubility, CuD faces challenges in bioavailability. These limitations could be addressed by utilizing advanced drug delivery systems, such as nanoparticles or liposomes, which can improve CuD’s solubility and minimize potential side effects. Future research should focus on optimizing these delivery methods and conducting further preclinical and clinical trials to evaluate CuD's safety and therapeutic efficacy, ensuring its potential as a viable single-component natural medicine in clinical settings.

## Conclusion

Our study sheds light on the profound potential of CuD in combating CRC, revealing its intricate mechanism through a comprehensive approach. The integration of rigorous experimental validation with advanced network pharmacological analyses elucidates CuD's multi-faceted impact on CRC. Our findings not only underscore the strength of CuD in significantly diminishing CRC cell viability and triggering apoptosis but also emphasize the critical role of network pharmacological analysis. Unraveling pivotal targets like *STAT3*, *AKT1*, *CCND1*, and *CASP3*, coupled with robust docking interactions, accentuates CuD's potency. Importantly, our enrichment analysis uncovers the involvement of crucial cancer-related pathways, including 'PI3K-AKT signaling,' 'JAK-STAT signaling,' and 'ErbB signaling.' This holistic understanding highlights the significance of CuD's multitarget, multipathway approach in CRC treatment.

While our study provides valuable insights, certain limitations exist. The experimental focus on a specific cell line subset might restrict the generalizability of our findings. Additionally, the in-depth exploration of CuD's in vivo effects and toxicity profiles would enhance its translational potential. Despite these limitations, our integrated approach combining experimental assays and computational analyses enhances our understanding of CuD's anti-CRC properties. This research not only positions CuD as a promising CRC therapeutic candidate but also underscores the significance of a multi-faceted, targeted approach in cancer treatment. Moving forward, further preclinical studies addressing these limitations are vital to propel CuD towards clinical applications, potentially revolutionizing CRC therapy.

## Supplementary Information

Additional file 1: Figure S1A. Construction ofthe compound-targetnetwork andthe compound-colorectal cancer-related target network.depicts the C-T network of Cucurbitacin D, with its potential targets highlighted in yellow and orange. The orange nodes specifically represent CRC-related targets among the Cucurbitacin D targets.

Additional file 2: Figure S1B. B illustrates the C-CRC-related target network, which is constructed by retaining only CuD and its CRC-related targetsfrom the C-T network.

Additional file 3: Figure S2. Protein–protein interaction network of identified anti-CRC targets of CuD.

Additional file 4: Table S1. The potential targets of CuD.

Additional file 5: Table S2. CRC-related genes.

Additional file 6: Table S3. GO Terms and KEGG pathways associated with the key targets of CuD in CRC.

## Data Availability

All data generated or analyzed during this study are included in this published article and its supplementary information files.
