# Supplementary figures and images for: Uncovering the anti-cancer mechanism of cucurbitacin D against colorectal cancer through network pharmacology and molecular docking
Source: Discov Oncol. 2025 Apr 17;16:551. doi: 10.1007/s12672-025-02056-7 (PMC12006582; doi:10.1007/s12672-025-02056-7)

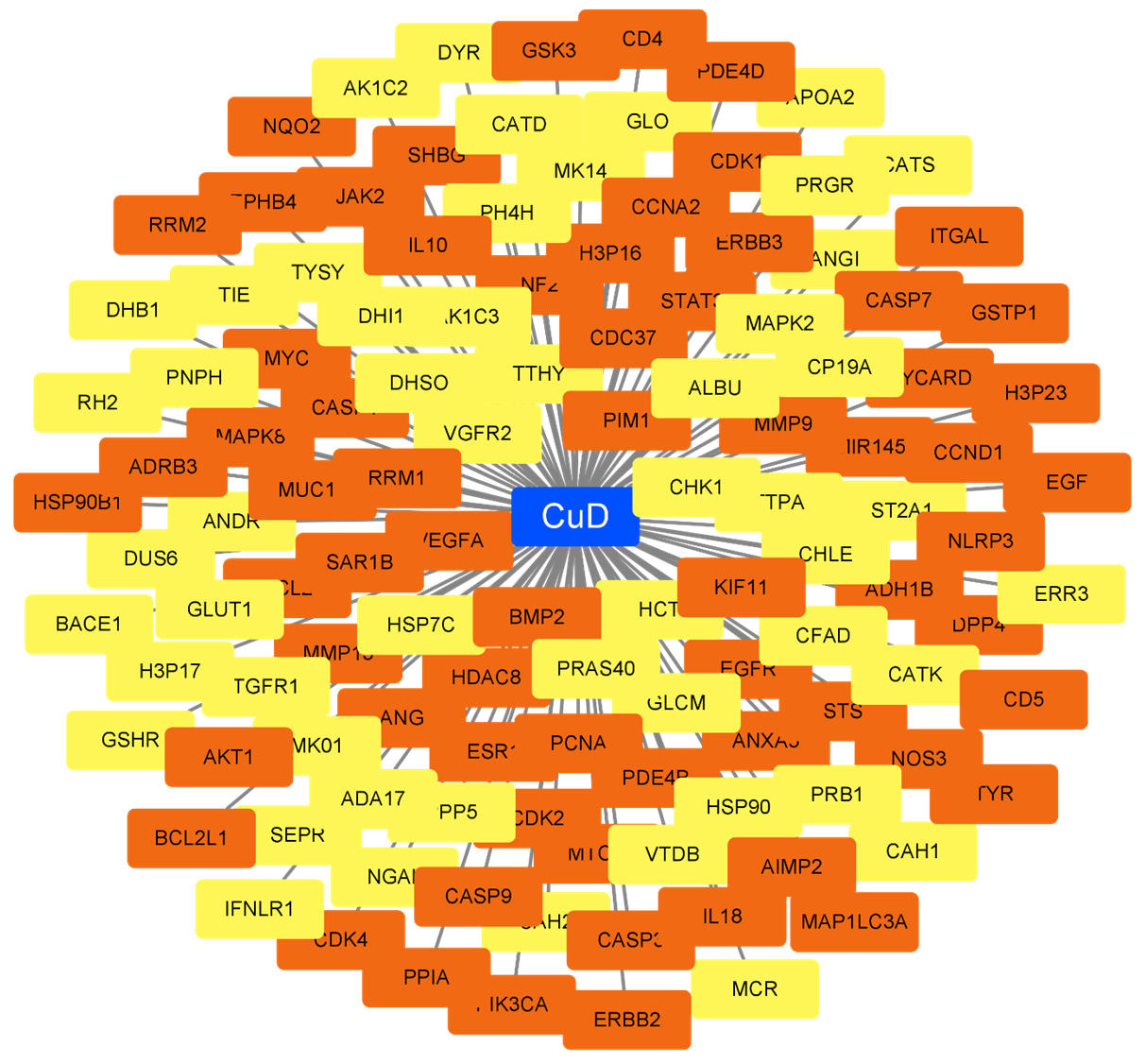

Supplement: Supplementary file 1 — Additional file 1: Figure S1A. Construction ofthe compound-targetnetwork andthe compound-colorectal cancer-related target network.depicts the C-T network of Cucurbitacin D, with its potential targets highlighted in yellow and orange. The orange nodes specifically represent CRC-related targets among the Cucurbitacin D targets. [file 12672_2025_2056_MOESM1_ESM.tif]

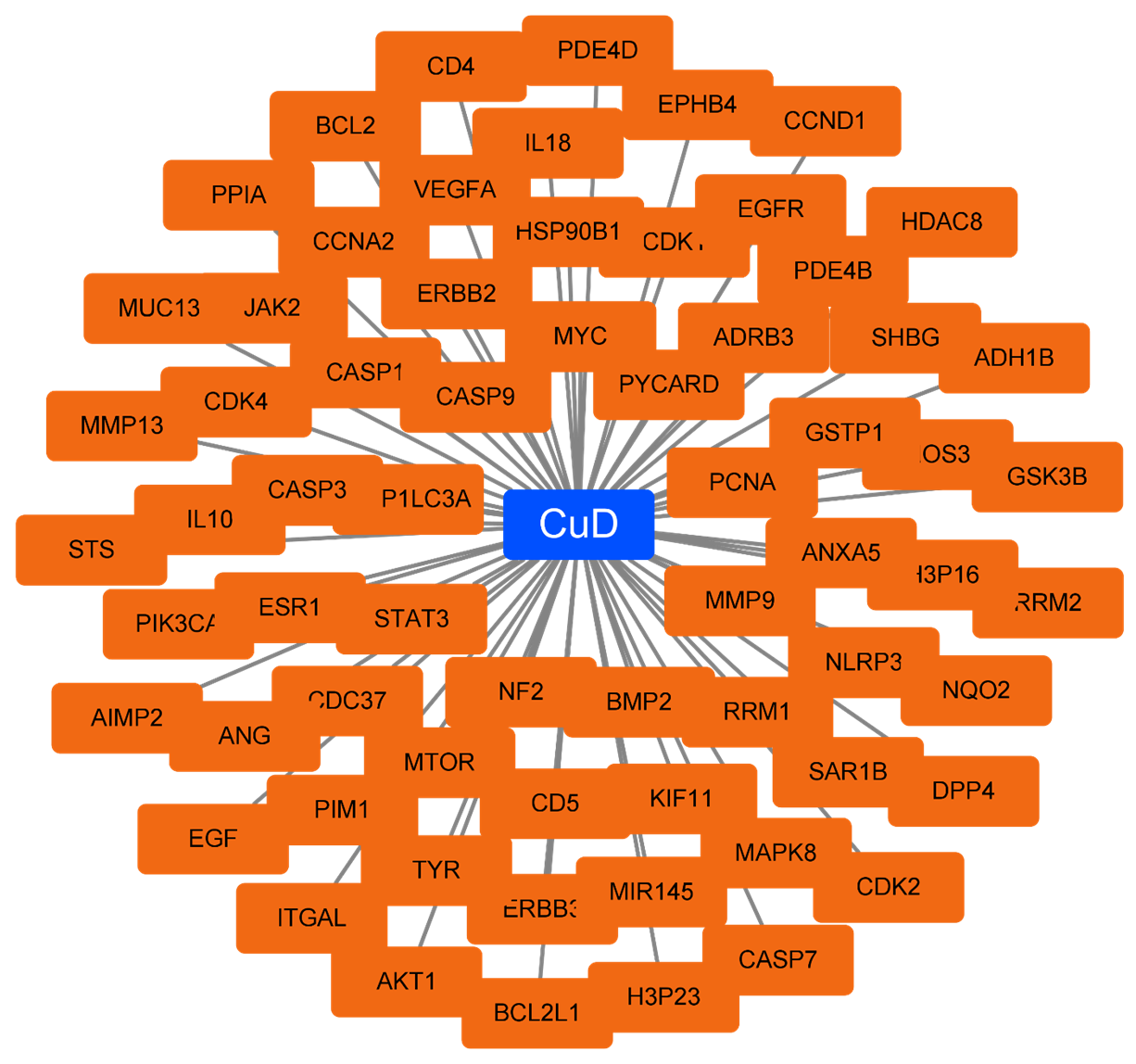

Supplement: Supplementary file 2 — Additional file 2: Figure S1B. B illustrates the C-CRC-related target network, which is constructed by retaining only CuD and its CRC-related targetsfrom the C-T network. [file 12672_2025_2056_MOESM2_ESM.tif]

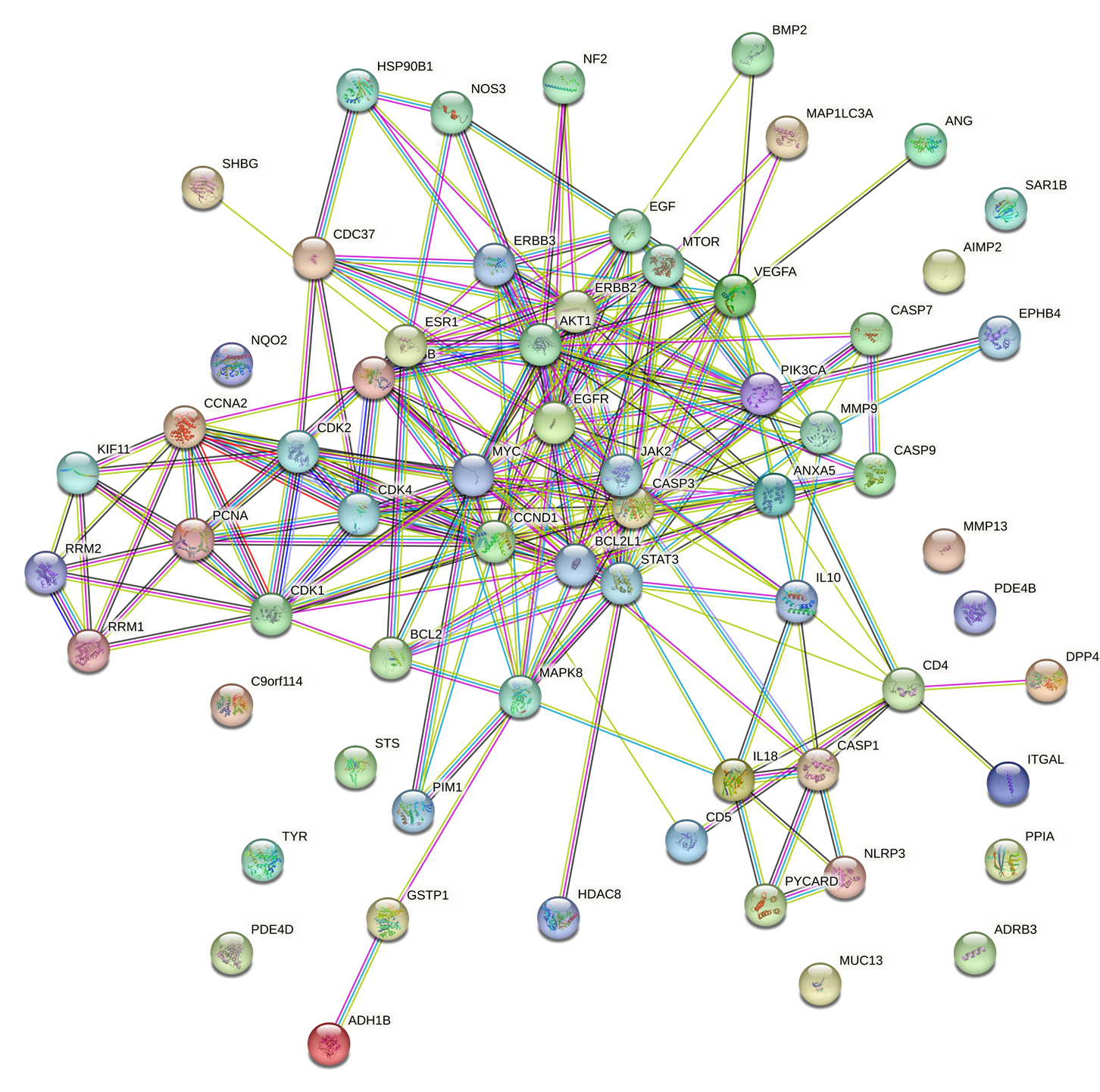

Supplement: Supplementary file 3 — Additional file 3: Figure S2. Protein–protein interaction network of identified anti-CRC targets of CuD. [file 12672_2025_2056_MOESM3_ESM.tif]
