# Supplementary material for: Uncovering the anti-cancer mechanism of cucurbitacin D against colorectal cancer through network pharmacology and molecular docking
Source: Discov Oncol. 2025 Apr 17;16:551. doi: 10.1007/s12672-025-02056-7 (PMC12006582; doi:10.1007/s12672-025-02056-7)
Supplement: Supplementary file 4 — Additional file 4: Table S1. The potential targets of CuD. [file 12672_2025_2056_MOESM4_ESM.docx]

**Table S1. The potential targets of CuD**

| **No.** | ***Gene*** |
| --- | --- |
| 1 | *ADA17* |
| 2 | *ADH1B* |
| 3 | *ADRB3* |
| 4 | *AIMP2* |
| 5 | *AK1C2* |
| 6 | *AK1C3* |
| 7 | *AKT1* |
| 8 | *ALBU* |
| 9 | *ANDR* |
| 10 | *ANG* |
| 11 | *ANGI* |
| 12 | *ANXA5* |
| 13 | *APOA2* |
| 14 | *BACE1* |
| 15 | *BCL2* |
| 16 | *BCL2L1* |
| 17 | *BMP2* |
| 18 | *CAH1* |
| 19 | *CAH2* |
| 20 | *CASP1* |
| 21 | *CASP3* |
| 22 | *CASP7* |
| 23 | *CASP9* |
| 24 | *CATD* |
| 25 | *CATK* |
| 26 | *CATS* |
| 27 | *CCNA2* |
| 28 | *CCND1* |
| 29 | *CD4* |
| 30 | *CD5* |
| 31 | *CDC37* |
| 32 | *CDK1* |
| 33 | *CDK2* |
| 34 | *CDK4* |
| 35 | *CFAD* |
| 36 | *CHK1* |
| 37 | *CHLE* |
| 38 | *CP19A* |
| 39 | *DHB1* |
| 40 | *DHI1* |
| 41 | *DHSO* |
| 42 | *DPP4* |
| 43 | *DUS6* |
| 44 | *DYR* |
| 45 | *EGF* |
| 46 | *EGFR* |
| 47 | *EPHB4* |
| 48 | *ERBB2* |
| 49 | *ERBB3* |
| 50 | *ERR3* |
| 51 | *ESR1* |
| 52 | *GLCM* |
| 53 | *GLO* |
| 54 | *GLUT1* |
| 55 | *GSHR* |
| 56 | *GSK3B* |
| 57 | *GSTP1* |
| 58 | *H3P16* |
| 59 | *H3P17* |
| 60 | *H3P23* |
| 61 | *HCT* |
| 62 | *HDAC8* |
| 63 | *HSP7C* |
| 64 | *HSP90* |
| 65 | *HSP90B1* |
| 66 | *IFNLR1* |
| 67 | *IL10* |
| 68 | *IL18* |
| 69 | *ITGAL* |
| 70 | *JAK2* |
| 71 | *KIF11* |
| 72 | *MAP1LC3A* |
| 73 | *MAPK2* |
| 74 | *MAPK8* |
| 75 | *MCR* |
| 76 | *MIR145* |
| 77 | *MK01* |
| 78 | *MK14* |
| 79 | *MMP13* |
| 80 | *MMP9* |
| 81 | *MTOR* |
| 82 | *MUC13* |
| 83 | *MYC* |
| 84 | *NF2* |
| 85 | *NGAL* |
| 86 | *NLRP3* |
| 87 | *NOS3* |
| 88 | *NQO2* |
| 89 | *PCNA* |
| 90 | *PDE4B* |
| 91 | *PDE4D* |
| 92 | *PH4H* |
| 93 | *PIK3CA* |
| 94 | *PIM1* |
| 95 | *PNPH* |
| 96 | *PPIA* |
| 97 | *PPP5* |
| 98 | *PRAS40* |
| 99 | *PRB1* |
| 100 | *PRGR* |
| 101 | *PYCARD* |
| 102 | *RH2* |
| 103 | *RRM1* |
| 104 | *RRM2* |
| 105 | *SAR1B* |
| 106 | *SEPR* |
| 107 | *SHBG* |
| 108 | *ST2A1* |
| 109 | *STAT3* |
| 110 | *STS* |
| 111 | *TGFR1* |
| 112 | *TIE* |
| 113 | *TTHY* |
| 114 | *TTPA* |
| 115 | *TYR* |
| 116 | *TYSY* |
| 117 | *VEGFA* |
| 118 | *VGFR2* |
| 119 | *VTDB* |
