# Supplementary material for: Uncovering the anti-cancer mechanism of cucurbitacin D against colorectal cancer through network pharmacology and molecular docking
Source: Discov Oncol. 2025 Apr 17;16:551. doi: 10.1007/s12672-025-02056-7 (PMC12006582; doi:10.1007/s12672-025-02056-7)
Supplement: Supplementary file 5 — Additional file 5: Table S2. CRC-related genes. [file 12672_2025_2056_MOESM5_ESM.docx]

**Table S2. CRC-related genes**

| **No.** | ***Gene*** |
| --- | --- |
| 1 | *A1CF* |
| 2 | *A2M* |
| 3 | *AADACL2* |
| 4 | *AAMP* |
| 5 | *AANAT* |
| 6 | *AARD* |
| 7 | *AARS1* |
| 8 | *AASDH* |
| 9 | *AASS* |
| 10 | *AATF* |
| 11 | *AATK* |
| 12 | *AAVS1* |
| 13 | *ABCA1* |
| 14 | *ABCA10* |
| 15 | *ABCA12* |
| 16 | *ABCA13* |
| 17 | *ABCA3* |
| 18 | *ABCA4* |
| 19 | *ABCA5* |
| 20 | *ABCA6* |
| 21 | *ABCA8* |
| 22 | *ABCA9* |
| 23 | *ABCB1* |
| 24 | *ABCB10* |
| 25 | *ABCB11* |
| 26 | *ABCB4* |
| 27 | *ABCB5* |
| 28 | *ABCB6* |
| 29 | *ABCB7* |
| 30 | *ABCB8* |
| 31 | *ABCB9* |
| 32 | *ABCC1* |
| 33 | *ABCC10* |
| 34 | *ABCC11* |
| 35 | *ABCC13* |
| 36 | *ABCC2* |
| 37 | *ABCC3* |
| 38 | *ABCC4* |
| 39 | *ABCC5* |
| 40 | *ABCC6* |
| 41 | *ABCC8* |
| 42 | *ABCD2* |
| 43 | *ABCD3* |
| 44 | *ABCD4* |
| 45 | *ABCE1* |
| 46 | *ABCF1* |
| 47 | *ABCF2* |
| 48 | *ABCF3* |
| 49 | *ABCG1* |
| 50 | *ABCG2* |
| 51 | *ABCG5* |
| 52 | *ABCG8* |
| 53 | *ABHD10* |
| 54 | *ABHD11* |
| 55 | *ABHD11-AS1* |
| 56 | *ABHD12B* |
| 57 | *ABHD14A-ACY1* |
| 58 | *ABHD14B* |
| 59 | *ABHD16A* |
| 60 | *ABHD5* |
| 61 | *ABI1* |
| 62 | *ABI2* |
| 63 | *ABL1* |
| 64 | *ABL2* |
| 65 | *ABLIM1* |
| 66 | *ABLIM3* |
| 67 | *ABO* |
| 68 | *ABR* |
| 69 | *ABRACL* |
| 70 | *ABRAXAS1* |
| 71 | *ABRAXAS2* |
| 72 | *ABTB1* |
| 73 | *ACAA1* |
| 74 | *ACAA2* |
| 75 | *ACACA* |
| 76 | *ACACB* |
| 77 | *ACAD10* |
| 78 | *ACAD8* |
| 79 | *ACAD9* |
| 80 | *ACADM* |
| 81 | *ACADS* |
| 82 | *ACADSB* |
| 83 | *ACADVL* |
| 84 | *ACAP1* |
| 85 | *ACAP2* |
| 86 | *ACAP3* |
| 87 | *ACAT1* |
| 88 | *ACCS* |
| 89 | *ACD* |
| 90 | *ACE* |
| 91 | *ACE2* |
| 92 | *ACER3* |
| 93 | *ACF* |
| 94 | *ACHE* |
| 95 | *ACIN1* |
| 96 | *ACKR1* |
| 97 | *ACKR2* |
| 98 | *ACKR3* |
| 99 | *ACKR4* |
| 100 | *ACKR4P1* |
| 101 | *ACLY* |
| 102 | *ACO2* |
| 103 | *ACOT7* |
| 104 | *ACOT9* |
| 105 | *ACOX1* |
| 106 | *ACOX2* |
| 107 | *ACOXL* |
| 108 | *ACP1* |
| 109 | *ACP2* |
| 110 | *ACP3* |
| 111 | *ACP5* |
| 112 | *ACP7* |
| 113 | *ACR* |
| 114 | *ACRBP* |
| 115 | *ACSF2* |
| 116 | *ACSL1* |
| 117 | *ACSL3* |
| 118 | *ACSL4* |
| 119 | *ACSL5* |
| 120 | *ACSS1* |
| 121 | *ACSS2* |
| 122 | *ACTA1* |
| 123 | *ACTA2* |
| 124 | *ACTB* |
| 125 | *ACTBL2* |
| 126 | *ACTC1* |
| 127 | *ACTG1* |
| 128 | *ACTG2* |
| 129 | *ACTL6A* |
| 130 | *ACTL7B* |
| 131 | *ACTL8* |
| 132 | *ACTL9* |
| 133 | *ACTN1* |
| 134 | *ACTN4* |
| 135 | *ACTR1A* |
| 136 | *ACTR1B* |
| 137 | *ACTR2* |
| 138 | *ACTR3* |
| 139 | *ACTR5* |
| 140 | *ACTR8* |
| 141 | *ACTRT1* |
| 142 | *ACTRT3* |
| 143 | *ACVR1* |
| 144 | *ACVR1B* |
| 145 | *ACVR2A* |
| 146 | *ACVR2B* |
| 147 | *ACVRL1* |
| 148 | *ACY1* |
| 149 | *ACYP1* |
| 150 | *ACYP2* |
| 151 | *ADA* |
| 152 | *ADAM10* |
| 153 | *ADAM11* |
| 154 | *ADAM12* |
| 155 | *ADAM15* |
| 156 | *ADAM17* |
| 157 | *ADAM19* |
| 158 | *ADAM1A* |
| 159 | *ADAM23* |
| 160 | *ADAM28* |
| 161 | *ADAM29* |
| 162 | *ADAM8* |
| 163 | *ADAM9* |
| 164 | *ADAMDEC1* |
| 165 | *ADAMTS1* |
| 166 | *ADAMTS10* |
| 167 | *ADAMTS12* |
| 168 | *ADAMTS13* |
| 169 | *ADAMTS14* |
| 170 | *ADAMTS15* |
| 171 | *ADAMTS16* |
| 172 | *ADAMTS17* |
| 173 | *ADAMTS18* |
| 174 | *ADAMTS19* |
| 175 | *ADAMTS20* |
| 176 | *ADAMTS3* |
| 177 | *ADAMTS4* |
| 178 | *ADAMTS5* |
| 179 | *ADAMTS8* |
| 180 | *ADAMTS9* |
| 181 | *ADAMTS9-AS2* |
| 182 | *ADAMTSL1* |
| 183 | *ADAMTSL3* |
| 184 | *ADAMTSL4* |
| 185 | *ADAR* |
| 186 | *ADARB1* |
| 187 | *ADARB2* |
| 188 | *ADAT1* |
| 189 | *ADAT3* |
| 190 | *ADCY1* |
| 191 | *ADCY10* |
| 192 | *ADCY10P1* |
| 193 | *ADCY2* |
| 194 | *ADCY3* |
| 195 | *ADCY4* |
| 196 | *ADCY5* |
| 197 | *ADCY6* |
| 198 | *ADCY7* |
| 199 | *ADCY8* |
| 200 | *ADCY9* |
| 201 | *ADCYAP1* |
| 202 | *ADD1* |
| 203 | *ADD2* |
| 204 | *ADGRA2* |
| 205 | *ADGRA3* |
| 206 | *ADGRB1* |
| 207 | *ADGRB2* |
| 208 | *ADGRB3* |
| 209 | *ADGRE2* |
| 210 | *ADGRE4P* |
| 211 | *ADGRE5* |
| 212 | *ADGRF4* |
| 213 | *ADGRF5* |
| 214 | *ADGRG1* |
| 215 | *ADGRG6* |
| 216 | *ADGRL1* |
| 217 | *ADGRL4* |
| 218 | *ADGRV1* |
| 219 | *ADH1A* |
| 220 | *ADH1B* |
| 221 | *ADH1C* |
| 222 | *ADH7* |
| 223 | *ADHFE1* |
| 224 | *ADIPOQ* |
| 225 | *ADIPOR1* |
| 226 | *ADIPOR2* |
| 227 | *ADK* |
| 228 | *ADM* |
| 229 | *ADM2* |
| 230 | *ADNP* |
| 231 | *ADORA1* |
| 232 | *ADORA2A* |
| 233 | *ADORA2B* |
| 234 | *ADORA3* |
| 235 | *ADPGK* |
| 236 | *ADPRM* |
| 237 | *ADRA1A* |
| 238 | *ADRA1B* |
| 239 | *ADRA2B* |
| 240 | *ADRB1* |
| 241 | *ADRB2* |
| 242 | *ADRB3* |
| 243 | *ADRM1* |
| 244 | *ADSL* |
| 245 | *ADTRP* |
| 246 | *AEBP1* |
| 247 | *AFA1* |
| 248 | *AFAP1* |
| 249 | *AFAP1-AS1* |
| 250 | *AFAP1L1* |
| 251 | *AFAP1L2* |
| 252 | *AFDN* |
| 253 | *AFF1* |
| 254 | *AFP* |
| 255 | *AGA* |
| 256 | *AGAP2* |
| 257 | *AGAP3* |
| 258 | *AGBL4* |
| 259 | *AGER* |
| 260 | *AGFG1* |
| 261 | *AGGF1* |
| 262 | *AGK* |
| 263 | *AGL* |
| 264 | *AGO1* |
| 265 | *AGO2* |
| 266 | *AGPAT1* |
| 267 | *AGPAT4* |
| 268 | *AGPAT5* |
| 269 | *AGPS* |
| 270 | *AGR2* |
| 271 | *AGR3* |
| 272 | *AGRN* |
| 273 | *AGRP* |
| 274 | *AGT* |
| 275 | *AGTPBP1* |
| 276 | *AGTR1* |
| 277 | *AGTR2* |
| 278 | *AGXT* |
| 279 | *AHCY* |
| 280 | *AHI1* |
| 281 | *AHNAK* |
| 282 | *AHNAK2* |
| 283 | *AHR* |
| 284 | *AHRR* |
| 285 | *AHSA1* |
| 286 | *AHSA2P* |
| 287 | *AHSG* |
| 288 | *AICDA* |
| 289 | *AIFM1* |
| 290 | *AIM2* |
| 291 | *AIMP1* |
| 292 | *AIMP2* |
| 293 | *AIP* |
| 294 | *AJUBA* |
| 295 | *AK2* |
| 296 | *AK3* |
| 297 | *AK5* |
| 298 | *AK6* |
| 299 | *AKAP1* |
| 300 | *AKAP10* |
| 301 | *AKAP11* |
| 302 | *AKAP12* |
| 303 | *AKAP13* |
| 304 | *AKAP3* |
| 305 | *AKAP4* |
| 306 | *AKAP6* |
| 307 | *AKAP8* |
| 308 | *AKAP8L* |
| 309 | *AKAP9* |
| 310 | *AKIP1* |
| 311 | *AKIRIN2* |
| 312 | *AKR1A1* |
| 313 | *AKR1B1* |
| 314 | *AKR1B10* |
| 315 | *AKR1B1P6* |
| 316 | *AKR1C1* |
| 317 | *AKR1C3* |
| 318 | *AKR7A2* |
| 319 | *AKR7A2P1* |
| 320 | *AKR7A3* |
| 321 | *AKR7L* |
| 322 | *AKT1* |
| 323 | *AKT1S1* |
| 324 | *AKT2* |
| 325 | *AKT3* |
| 326 | *ALAS1* |
| 327 | *ALB* |
| 328 | *ALCAM* |
| 329 | *ALDH16A1* |
| 330 | *ALDH18A1* |
| 331 | *ALDH1A1* |
| 332 | *ALDH1A3* |
| 333 | *ALDH1B1* |
| 334 | *ALDH1L1* |
| 335 | *ALDH2* |
| 336 | *ALDH3A1* |
| 337 | *ALDH3A2* |
| 338 | *ALDH7A1* |
| 339 | *ALDH9A1* |
| 340 | *ALDOA* |
| 341 | *ALDOB* |
| 342 | *ALDOC* |
| 343 | *ALG13* |
| 344 | *ALG6* |
| 345 | *ALG9* |
| 346 | *ALK* |
| 347 | *ALKAL2* |
| 348 | *ALKBH2* |
| 349 | *ALKBH5* |
| 350 | *ALKBH8* |
| 351 | *ALOX12* |
| 352 | *ALOX15* |
| 353 | *ALOX15B* |
| 354 | *ALOX5* |
| 355 | *ALOX5AP* |
| 356 | *ALOXE3* |
| 357 | *ALPG* |
| 358 | *ALPI* |
| 359 | *ALPK1* |
| 360 | *ALPK2* |
| 361 | *ALPL* |
| 362 | *ALPP* |
| 363 | *ALS2CL* |
| 364 | *ALX3* |
| 365 | *ALX4* |
| 366 | *ALYREF* |
| 367 | *AMACR* |
| 368 | *AMBP* |
| 369 | *AMBRA1* |
| 370 | *AMCN* |
| 371 | *AMD1* |
| 372 | *AMDHD2* |
| 373 | *AMELX* |
| 374 | *AMER1* |
| 375 | *AMER2* |
| 376 | *AMER3* |
| 377 | *AMFR* |
| 378 | *AMIGO2* |
| 379 | *AMOT* |
| 380 | *AMOTL2* |
| 381 | *AMPD1* |
| 382 | *AMPD2* |
| 383 | *AMT* |
| 384 | *AMTN* |
| 385 | *AMY1A* |
| 386 | *AMY2A* |
| 387 | *AMY2B* |
| 388 | *AMZ2P1* |
| 389 | *ANAPC1* |
| 390 | *ANAPC10* |
| 391 | *ANAPC11* |
| 392 | *ANAPC4* |
| 393 | *ANAPC5* |
| 394 | *ANAPC7* |
| 395 | *ANCR* |
| 396 | *ANG* |
| 397 | *ANGPT1* |
| 398 | *ANGPT2* |
| 399 | *ANGPTL1* |
| 400 | *ANGPTL2* |
| 401 | *ANGPTL4* |
| 402 | *ANGPTL6* |
| 403 | *ANGPTL7* |
| 404 | *ANIB1* |
| 405 | *ANK1* |
| 406 | *ANK2* |
| 407 | *ANKAR* |
| 408 | *ANKEF1* |
| 409 | *ANKFN1* |
| 410 | *ANKHD1* |
| 411 | *ANKHD1-EIF4EBP3* |
| 412 | *ANKLE1* |
| 413 | *ANKRD1* |
| 414 | *ANKRD10* |
| 415 | *ANKRD12* |
| 416 | *ANKRD13A* |
| 417 | *ANKRD17* |
| 418 | *ANKRD22* |
| 419 | *ANKRD26* |
| 420 | *ANKRD27* |
| 421 | *ANKRD28* |
| 422 | *ANKRD29* |
| 423 | *ANKRD30A* |
| 424 | *ANKRD36B* |
| 425 | *ANKRD40CL* |
| 426 | *ANKRD46* |
| 427 | *ANKRD55* |
| 428 | *ANKRD65* |
| 429 | *ANKS1A* |
| 430 | *ANKS1B* |
| 431 | *ANKZF1* |
| 432 | *ANLN* |
| 433 | *ANO1* |
| 434 | *ANO9* |
| 435 | *ANOS1* |
| 436 | *ANP32A* |
| 437 | *ANP32B* |
| 438 | *ANPEP* |
| 439 | *ANTXR1* |
| 440 | *ANXA1* |
| 441 | *ANXA10* |
| 442 | *ANXA11* |
| 443 | *ANXA13* |
| 444 | *ANXA2* |
| 445 | *ANXA2P2* |
| 446 | *ANXA3* |
| 447 | *ANXA4* |
| 448 | *ANXA5* |
| 449 | *ANXA6* |
| 450 | *ANXA7* |
| 451 | *ANXA9* |
| 452 | *AOC1* |
| 453 | *AOC3* |
| 454 | *AOC4P* |
| 455 | *AOPEP* |
| 456 | *AOX1* |
| 457 | *AP1B1* |
| 458 | *AP1G1* |
| 459 | *AP1G2* |
| 460 | *AP1M1* |
| 461 | *AP2A1* |
| 462 | *AP2A2* |
| 463 | *AP2B1* |
| 464 | *AP2M1* |
| 465 | *AP2S1* |
| 466 | *AP3B1* |
| 467 | *AP3B2* |
| 468 | *AP3D1* |
| 469 | *AP3M1* |
| 470 | *AP3S1* |
| 471 | *APAF1* |
| 472 | *APBA1* |
| 473 | *APBA2* |
| 474 | *APBB2* |
| 475 | *APC* |
| 476 | *APC2* |
| 477 | *APCDD1* |
| 478 | *APCS* |
| 479 | *APEH* |
| 480 | *APEX1* |
| 481 | *APH1A* |
| 482 | *APH1B* |
| 483 | *APIP* |
| 484 | *APLN* |
| 485 | *APLNR* |
| 486 | *APLP1* |
| 487 | *APLP2* |
| 488 | *APMAP* |
| 489 | *APOA1* |
| 490 | *APOA4* |
| 491 | *APOA5* |
| 492 | *APOB* |
| 493 | *APOBEC1* |
| 494 | *APOBEC2* |
| 495 | *APOBEC3A* |
| 496 | *APOBEC3C* |
| 497 | *APOBEC3F* |
| 498 | *APOBEC3G* |
| 499 | *APOC1* |
| 500 | *APOC3* |
| 501 | *APOC4* |
| 502 | *APOD* |
| 503 | *APOE* |
| 504 | *APOH* |
| 505 | *APOL1* |
| 506 | *APOL6* |
| 507 | *APOM* |
| 508 | *APP* |
| 509 | *APPL1* |
| 510 | *APPL2* |
| 511 | *APRT* |
| 512 | *APTX* |
| 513 | *AQP1* |
| 514 | *AQP2* |
| 515 | *AQP3* |
| 516 | *AQP5* |
| 517 | *AQP6* |
| 518 | *AQP8* |
| 519 | *AQP9* |
| 520 | *AQR* |
| 521 | *AR* |
| 522 | *ARAF* |
| 523 | *ARAP1* |
| 524 | *ARAP3* |
| 525 | *ARC* |
| 526 | *ARCN1* |
| 527 | *AREG* |
| 528 | *AREL1* |
| 529 | *ARF1* |
| 530 | *ARF3* |
| 531 | *ARF4* |
| 532 | *ARF5* |
| 533 | *ARF6* |
| 534 | *ARFGAP1* |
| 535 | *ARFGAP3* |
| 536 | *ARFGEF1* |
| 537 | *ARFGEF2* |
| 538 | *ARFRP1* |
| 539 | *ARG1* |
| 540 | *ARG2* |
| 541 | *ARGLU1* |
| 542 | *ARHGAP11A* |
| 543 | *ARHGAP12* |
| 544 | *ARHGAP15* |
| 545 | *ARHGAP17* |
| 546 | *ARHGAP24* |
| 547 | *ARHGAP25* |
| 548 | *ARHGAP29* |
| 549 | *ARHGAP30* |
| 550 | *ARHGAP32* |
| 551 | *ARHGAP35* |
| 552 | *ARHGAP4* |
| 553 | *ARHGAP42* |
| 554 | *ARHGAP45* |
| 555 | *ARHGAP5* |
| 556 | *ARHGAP6* |
| 557 | *ARHGAP8* |
| 558 | *ARHGAP9* |
| 559 | *ARHGDIA* |
| 560 | *ARHGDIB* |
| 561 | *ARHGEF1* |
| 562 | *ARHGEF10* |
| 563 | *ARHGEF10L* |
| 564 | *ARHGEF11* |
| 565 | *ARHGEF12* |
| 566 | *ARHGEF16* |
| 567 | *ARHGEF17* |
| 568 | *ARHGEF18* |
| 569 | *ARHGEF19* |
| 570 | *ARHGEF2* |
| 571 | *ARHGEF25* |
| 572 | *ARHGEF28* |
| 573 | *ARHGEF3* |
| 574 | *ARHGEF35* |
| 575 | *ARHGEF38* |
| 576 | *ARHGEF4* |
| 577 | *ARHGEF5* |
| 578 | *ARHGEF6* |
| 579 | *ARHGEF7* |
| 580 | *ARHGEF9* |
| 581 | *ARID1A* |
| 582 | *ARID1B* |
| 583 | *ARID2* |
| 584 | *ARID3A* |
| 585 | *ARID3B* |
| 586 | *ARID4A* |
| 587 | *ARID4B* |
| 588 | *ARID5B* |
| 589 | *ARIH1* |
| 590 | *ARL1* |
| 591 | *ARL11* |
| 592 | *ARL17A* |
| 593 | *ARL2* |
| 594 | *ARL3* |
| 595 | *ARL4C* |
| 596 | *ARL5A* |
| 597 | *ARL5B* |
| 598 | *ARL6IP1* |
| 599 | *ARL6IP4* |
| 600 | *ARL6IP5* |
| 601 | *ARL6IP6* |
| 602 | *ARL8A* |
| 603 | *ARMC10* |
| 604 | *ARMC12* |
| 605 | *ARMC3* |
| 606 | *ARMC5* |
| 607 | *ARMC6* |
| 608 | *ARMCX1* |
| 609 | *ARMCX5-GPRASP2* |
| 610 | *ARMCX6* |
| 611 | *ARMH1* |
| 612 | *ARNT* |
| 613 | *ARNTL* |
| 614 | *ARNTL2* |
| 615 | *ARPC1A* |
| 616 | *ARPC1B* |
| 617 | *ARPC2* |
| 618 | *ARPC3* |
| 619 | *ARPC4* |
| 620 | *ARPC5* |
| 621 | *ARPC5L* |
| 622 | *ARR3* |
| 623 | *ARRB1* |
| 624 | *ARRB2* |
| 625 | *ARRDC1* |
| 626 | *ARRDC3* |
| 627 | *ARSA* |
| 628 | *ARSB* |
| 629 | *ARSG* |
| 630 | *ARSH* |
| 631 | *ART1* |
| 632 | *ARTN* |
| 633 | *AS3MT* |
| 634 | *ASAH1* |
| 635 | *ASAH2* |
| 636 | *ASAP1* |
| 637 | *ASAP2* |
| 638 | *ASAP3* |
| 639 | *ASB1* |
| 640 | *ASB3* |
| 641 | *ASB4* |
| 642 | *ASB6* |
| 643 | *ASB9* |
| 644 | *ASCC1* |
| 645 | *ASCC2* |
| 646 | *ASCC3* |
| 647 | *ASCL2* |
| 648 | *ASF1A* |
| 649 | *ASGR1* |
| 650 | *ASGR2* |
| 651 | *ASH2L* |
| 652 | *ASIC2* |
| 653 | *ASIC3* |
| 654 | *ASIP* |
| 655 | *ASL* |
| 656 | *ASNS* |
| 657 | *ASNSD1* |
| 658 | *ASPA* |
| 659 | *ASPH* |
| 660 | *ASPM* |
| 661 | *ASPN* |
| 662 | *ASPRV1* |
| 663 | *ASRGL1* |
| 664 | *ASS1* |
| 665 | *ASTE1* |
| 666 | *ASTN2* |
| 667 | *ASXL1* |
| 668 | *ASXL2* |
| 669 | *ASXL3* |
| 670 | *ATAD1* |
| 671 | *ATAD2* |
| 672 | *ATAD3A* |
| 673 | *ATAD3B* |
| 674 | *ATAD3C* |
| 675 | *ATD* |
| 676 | *ATF1* |
| 677 | *ATF2* |
| 678 | *ATF3* |
| 679 | *ATF4* |
| 680 | *ATF6* |
| 681 | *ATF6B* |
| 682 | *ATF7* |
| 683 | *ATF7IP* |
| 684 | *ATG10* |
| 685 | *ATG12* |
| 686 | *ATG14* |
| 687 | *ATG16L1* |
| 688 | *ATG2B* |
| 689 | *ATG3* |
| 690 | *ATG4B* |
| 691 | *ATG4D* |
| 692 | *ATG5* |
| 693 | *ATG7* |
| 694 | *ATG9A* |
| 695 | *ATG9B* |
| 696 | *ATIC* |
| 697 | *ATM* |
| 698 | *ATMIN* |
| 699 | *ATN1* |
| 700 | *ATOH1* |
| 701 | *ATOH8* |
| 702 | *ATOX1* |
| 703 | *ATP11A* |
| 704 | *ATP11C* |
| 705 | *ATP12A* |
| 706 | *ATP13A1* |
| 707 | *ATP13A2* |
| 708 | *ATP1A1* |
| 709 | *ATP1B1* |
| 710 | *ATP1B3* |
| 711 | *ATP2A1* |
| 712 | *ATP2A2* |
| 713 | *ATP2A3* |
| 714 | *ATP2B1* |
| 715 | *ATP2B4* |
| 716 | *ATP2C1* |
| 717 | *ATP4A* |
| 718 | *ATP5F1A* |
| 719 | *ATP5F1B* |
| 720 | *ATP5F1C* |
| 721 | *ATP5F1CP1* |
| 722 | *ATP5F1D* |
| 723 | *ATP5F1E* |
| 724 | *ATP5F1EP2* |
| 725 | *ATP5IF1* |
| 726 | *ATP5ME* |
| 727 | *ATP5MF* |
| 728 | *ATP5MFP5* |
| 729 | *ATP5MF-PTCD1* |
| 730 | *ATP5MG* |
| 731 | *ATP5MJ* |
| 732 | *ATP5MK* |
| 733 | *ATP5PB* |
| 734 | *ATP5PBP4* |
| 735 | *ATP5PD* |
| 736 | *ATP5PF* |
| 737 | *ATP5PO* |
| 738 | *ATP6AP2* |
| 739 | *ATP6V0A1* |
| 740 | *ATP6V0B* |
| 741 | *ATP6V0C* |
| 742 | *ATP6V0D1* |
| 743 | *ATP6V1A* |
| 744 | *ATP6V1E1* |
| 745 | *ATP6V1G1* |
| 746 | *ATP7A* |
| 747 | *ATP7B* |
| 748 | *ATP8B1* |
| 749 | *ATP8B3* |
| 750 | *ATPSCKMT* |
| 751 | *ATR* |
| 752 | *ATRX* |
| 753 | *ATXN10* |
| 754 | *ATXN2* |
| 755 | *ATXN2L* |
| 756 | *ATXN3* |
| 757 | *ATXN7* |
| 758 | *ATXN7L3B* |
| 759 | *AURKA* |
| 760 | *AURKAIP1* |
| 761 | *AURKB* |
| 762 | *AURKC* |
| 763 | *AVP* |
| 764 | *AVPR2* |
| 765 | *AXDND1* |
| 766 | *AXIN1* |
| 767 | *AXIN2* |
| 768 | *AXL* |
| 769 | *AZF1* |
| 770 | *AZGP1* |
| 771 | *AZIN1* |
| 772 | *AZIN2* |
| 773 | *B2M* |
| 774 | *B3GALNT2* |
| 775 | *B3GALT4* |
| 776 | *B3GALT5* |
| 777 | *B3GAT1* |
| 778 | *B3GLCT* |
| 779 | *B3GNT2* |
| 780 | *B3GNT6* |
| 781 | *B3GNT8* |
| 782 | *B3GNTL1* |
| 783 | *B4GALNT1* |
| 784 | *B4GALNT2* |
| 785 | *B4GALNT3* |
| 786 | *B4GALT1* |
| 787 | *B4GALT2* |
| 788 | *B4GALT3* |
| 789 | *B4GALT4* |
| 790 | *B4GALT5* |
| 791 | *B4GALT6* |
| 792 | *B9D2* |
| 793 | *BAALC* |
| 794 | *BAALC-AS1* |
| 795 | *BAAT* |
| 796 | *BABAM1* |
| 797 | *BABAM2* |
| 798 | *BACE2* |
| 799 | *BACH1* |
| 800 | *BAD* |
| 801 | *BAG1* |
| 802 | *BAG2* |
| 803 | *BAG3* |
| 804 | *BAG4* |
| 805 | *BAG5* |
| 806 | *BAG6* |
| 807 | *BAGE* |
| 808 | *BAIAP2* |
| 809 | *BAIAP3* |
| 810 | *BAK1* |
| 811 | *BAMBI* |
| 812 | *BANCR* |
| 813 | *BANF1* |
| 814 | *BANP* |
| 815 | *BAP1* |
| 816 | *BARD1* |
| 817 | *BARHL2* |
| 818 | *BARX2* |
| 819 | *BASP1* |
| 820 | *BATF* |
| 821 | *BATF2* |
| 822 | *BATF3* |
| 823 | *BAX* |
| 824 | *BAZ1A* |
| 825 | *BBC3* |
| 826 | *BBS1* |
| 827 | *BBS2* |
| 828 | *BCAM* |
| 829 | *BCAP31* |
| 830 | *BCAR1* |
| 831 | *BCAR3* |
| 832 | *BCAR4* |
| 833 | *BCAS1* |
| 834 | *BCAS2* |
| 835 | *BCAS3* |
| 836 | *BCAS4* |
| 837 | *BCAT1* |
| 838 | *BCCIP* |
| 839 | *BCDIN3D* |
| 840 | *BCHE* |
| 841 | *BCKDHA* |
| 842 | *BCKDHB* |
| 843 | *BCKDK* |
| 844 | *BCL10* |
| 845 | *BCL11A* |
| 846 | *BCL11B* |
| 847 | *BCL2* |
| 848 | *BCL2A1* |
| 849 | *BCL2L1* |
| 850 | *BCL2L10* |
| 851 | *BCL2L11* |
| 852 | *BCL2L12* |
| 853 | *BCL2L14* |
| 854 | *BCL2L15* |
| 855 | *BCL2L2* |
| 856 | *BCL2L2-PABPN1* |
| 857 | *BCL3* |
| 858 | *BCL6* |
| 859 | *BCL6B* |
| 860 | *BCL9* |
| 861 | *BCL9L* |
| 862 | *BCLAF1* |
| 863 | *BCO1* |
| 864 | *BCORL1* |
| 865 | *BCR* |
| 866 | *BCYRN1* |
| 867 | *BDH1* |
| 868 | *BDKRB1* |
| 869 | *BDKRB2* |
| 870 | *BDNF* |
| 871 | *BDP1* |
| 872 | *BECN1* |
| 873 | *BEND4* |
| 874 | *BEND5* |
| 875 | *BEND7* |
| 876 | *BEST1* |
| 877 | *BEST4* |
| 878 | *BEX2* |
| 879 | *BEX3* |
| 880 | *BGN* |
| 881 | *BHD* |
| 882 | *BHLHE22* |
| 883 | *BHLHE23* |
| 884 | *BHLHE40* |
| 885 | *BHLHE41* |
| 886 | *BHMT* |
| 887 | *BICC1* |
| 888 | *BICRA* |
| 889 | *BID* |
| 890 | *BIK* |
| 891 | *BIN1* |
| 892 | *BIRC2* |
| 893 | *BIRC3* |
| 894 | *BIRC5* |
| 895 | *BIRC6* |
| 896 | *BIRC7* |
| 897 | *BLACAT1* |
| 898 | *BLCAP* |
| 899 | *BLID* |
| 900 | *BLK* |
| 901 | *BLM* |
| 902 | *BLNK* |
| 903 | *BLTP2* |
| 904 | *BLVRB* |
| 905 | *BLZF1* |
| 906 | *BMAL1* |
| 907 | *BMAL2* |
| 908 | *BMERB1* |
| 909 | *BMF* |
| 910 | *BMI1* |
| 911 | *BMP1* |
| 912 | *BMP2* |
| 913 | *BMP2K* |
| 914 | *BMP3* |
| 915 | *BMP4* |
| 916 | *BMP5* |
| 917 | *BMP6* |
| 918 | *BMP7* |
| 919 | *BMP8A* |
| 920 | *BMPR1A* |
| 921 | *BMPR1B* |
| 922 | *BMPR2* |
| 923 | *BMS1* |
| 924 | *BMX* |
| 925 | *BNC2* |
| 926 | *BNIP1* |
| 927 | *BNIP3* |
| 928 | *BNIP3L* |
| 929 | *BNIPL* |
| 930 | *BOC* |
| 931 | *BOD1L1* |
| 932 | *BOK* |
| 933 | *BOK-AS1* |
| 934 | *BOLA2* |
| 935 | *BOLA3P3* |
| 936 | *BOLL* |
| 937 | *BOP1* |
| 938 | *BORCS5* |
| 939 | *BORCS7* |
| 940 | *BORCS8-MEF2B* |
| 941 | *BPHL* |
| 942 | *BPIFA1* |
| 943 | *BPIFB2* |
| 944 | *BPTF* |
| 945 | *BRAF* |
| 946 | *BRAP* |
| 947 | *BRCA1* |
| 948 | *BRCA2* |
| 949 | *BRCA3* |
| 950 | *BRCC3* |
| 951 | *BRD1* |
| 952 | *BRD2* |
| 953 | *BRD3* |
| 954 | *BRD4* |
| 955 | *BRD7* |
| 956 | *BRD8* |
| 957 | *BRF1* |
| 958 | *BRI3* |
| 959 | *BRINP1* |
| 960 | *BRIP1* |
| 961 | *BRIX1* |
| 962 | *BRMS1* |
| 963 | *BRPF3* |
| 964 | *BRS3* |
| 965 | *BRSK1* |
| 966 | *BRWD1* |
| 967 | *BRWD3* |
| 968 | *BSG* |
| 969 | *BSN* |
| 970 | *BST2* |
| 971 | *BTAF1* |
| 972 | *BTBD10* |
| 973 | *BTBD7* |
| 974 | *BTBD9* |
| 975 | *BTC* |
| 976 | *BTF3* |
| 977 | *BTF3L4* |
| 978 | *BTF3P11* |
| 979 | *BTF3P7* |
| 980 | *BTG1* |
| 981 | *BTG2* |
| 982 | *BTG3* |
| 983 | *BTG4* |
| 984 | *BTK* |
| 985 | *BTLA* |
| 986 | *BTN3A1* |
| 987 | *BTRC* |
| 988 | *BUB1* |
| 989 | *BUB1B* |
| 990 | *BUB1B-PAK6* |
| 991 | *BUB3* |
| 992 | *BUD13* |
| 993 | *BUD23* |
| 994 | *BVES* |
| 995 | *BYSL* |
| 996 | *BZW1* |
| 997 | *BZW2* |
| 998 | *C10orf143* |
| 999 | *C11orf24* |
| 1000 | *C11orf53* |
| 1001 | *C11orf58* |
| 1002 | *C11orf65* |
| 1003 | *C12orf4* |
| 1004 | *C12orf75* |
| 1005 | *C12orf76* |
| 1006 | *C14orf28* |
| 1007 | *C16orf95* |
| 1008 | *C17orf58* |
| 1009 | *C17orf97* |
| 1010 | *C18orf32* |
| 1011 | *C19orf25* |
| 1012 | *C19orf33* |
| 1013 | *C19orf48* |
| 1014 | *C19orf53* |
| 1015 | *C1D* |
| 1016 | *C1GALT1* |
| 1017 | *C1GALT1C1* |
| 1018 | *C1orf109* |
| 1019 | *C1orf198* |
| 1020 | *C1orf21* |
| 1021 | *C1orf35* |
| 1022 | *C1orf43* |
| 1023 | *C1orf68* |
| 1024 | *C1orf87* |
| 1025 | *C1QB* |
| 1026 | *C1QBP* |
| 1027 | *C1QC* |
| 1028 | *C1QL4* |
| 1029 | *C1QTNF4* |
| 1030 | *C1R* |
| 1031 | *C20orf27* |
| 1032 | *C2CD2* |
| 1033 | *C2CD2L* |
| 1034 | *C2CD6* |
| 1035 | *C2orf68* |
| 1036 | *C2orf92* |
| 1037 | *C3* |
| 1038 | *C3orf38* |
| 1039 | *C3orf85* |
| 1040 | *C4A* |
| 1041 | *C4BPA* |
| 1042 | *C4orf50* |
| 1043 | *C4orf54* |
| 1044 | *C5orf15* |
| 1045 | *C5orf24* |
| 1046 | *C5orf34* |
| 1047 | *C5orf66* |
| 1048 | *C6orf136* |
| 1049 | *C8orf33* |
| 1050 | *C8orf34* |
| 1051 | *C8orf44-SGK3* |
| 1052 | *C8orf48* |
| 1053 | *C8orf82* |
| 1054 | *C9* |
| 1055 | *CA1* |
| 1056 | *CA10* |
| 1057 | *CA11* |
| 1058 | *CA12* |
| 1059 | *CA13* |
| 1060 | *CA2* |
| 1061 | *CA3-AS1* |
| 1062 | *CA4* |
| 1063 | *CA6* |
| 1064 | *CA7* |
| 1065 | *CA8* |
| 1066 | *CA9* |
| 1067 | *CAB39* |
| 1068 | *CAB39L* |
| 1069 | *CABLES1* |
| 1070 | *CABLES2* |
| 1071 | *CABYR* |
| 1072 | *CACNA1D* |
| 1073 | *CACNA1F* |
| 1074 | *CACNA1G* |
| 1075 | *CACNA2D1* |
| 1076 | *CACNA2D2* |
| 1077 | *CACNB1* |
| 1078 | *CACNB2* |
| 1079 | *CACUL1* |
| 1080 | *CACYBP* |
| 1081 | *CAD* |
| 1082 | *CADM1* |
| 1083 | *CADPS* |
| 1084 | *CAGE1* |
| 1085 | *CAHM* |
| 1086 | *CALB1* |
| 1087 | *CALB2* |
| 1088 | *CALCA* |
| 1089 | *CALCOCO1* |
| 1090 | *CALCOCO2* |
| 1091 | *CALCR* |
| 1092 | *CALCRL* |
| 1093 | *CALD1* |
| 1094 | *CALM1* |
| 1095 | *CALM2* |
| 1096 | *CALM3* |
| 1097 | *CALML3* |
| 1098 | *CALML4* |
| 1099 | *CALML5* |
| 1100 | *CALR* |
| 1101 | *CALU* |
| 1102 | *CAMK1D* |
| 1103 | *CAMK2A* |
| 1104 | *CAMK2B* |
| 1105 | *CAMK2D* |
| 1106 | *CAMK2G* |
| 1107 | *CAMK2N1* |
| 1108 | *CAMKK2* |
| 1109 | *CAMKMT* |
| 1110 | *CAMKV* |
| 1111 | *CAMLG* |
| 1112 | *CAMP* |
| 1113 | *CAMSAP1* |
| 1114 | *CAMSAP2* |
| 1115 | *CAMSAP3* |
| 1116 | *CAMTA1* |
| 1117 | *CAND1* |
| 1118 | *CANX* |
| 1119 | *CAP1* |
| 1120 | *CAP2* |
| 1121 | *CAPG* |
| 1122 | *CAPN1* |
| 1123 | *CAPN10* |
| 1124 | *CAPN13* |
| 1125 | *CAPN15* |
| 1126 | *CAPN2* |
| 1127 | *CAPN5* |
| 1128 | *CAPN6* |
| 1129 | *CAPN9* |
| 1130 | *CAPNS1* |
| 1131 | *CAPRIN1* |
| 1132 | *CAPRIN2* |
| 1133 | *CAPS* |
| 1134 | *CAPZA1* |
| 1135 | *CAPZA2* |
| 1136 | *CAPZB* |
| 1137 | *CARD10* |
| 1138 | *CARD11* |
| 1139 | *CARD6* |
| 1140 | *CARD8* |
| 1141 | *CARD9* |
| 1142 | *CARF* |
| 1143 | *CARM1* |
| 1144 | *CARMIL1* |
| 1145 | *CARNMT1* |
| 1146 | *CARS1* |
| 1147 | *CASC11* |
| 1148 | *CASC15* |
| 1149 | *CASC19* |
| 1150 | *CASC2* |
| 1151 | *CASC21* |
| 1152 | *CASC8* |
| 1153 | *CASC9* |
| 1154 | *CASD1* |
| 1155 | *CASK* |
| 1156 | *CASKIN2* |
| 1157 | *CASP1* |
| 1158 | *CASP10* |
| 1159 | *CASP14* |
| 1160 | *CASP2* |
| 1161 | *CASP3* |
| 1162 | *CASP3P1* |
| 1163 | *CASP4* |
| 1164 | *CASP5* |
| 1165 | *CASP6* |
| 1166 | *CASP7* |
| 1167 | *CASP8* |
| 1168 | *CASP8AP2* |
| 1169 | *CASP9* |
| 1170 | *CASR* |
| 1171 | *CAST* |
| 1172 | *CASTOR3P* |
| 1173 | *CASZ1* |
| 1174 | *CAT* |
| 1175 | *CATIP* |
| 1176 | *CATSPER3* |
| 1177 | *CATSPERE* |
| 1178 | *CAV1* |
| 1179 | *CAV2* |
| 1180 | *CAV3* |
| 1181 | *CAVIN1* |
| 1182 | *CAVIN3* |
| 1183 | *CBFA2T2* |
| 1184 | *CBFA2T3* |
| 1185 | *CBFB* |
| 1186 | *CBL* |
| 1187 | *CBLB* |
| 1188 | *CBLIF* |
| 1189 | *CBLL1* |
| 1190 | *CBLL2* |
| 1191 | *CBLN1* |
| 1192 | *CBLN2* |
| 1193 | *CBLN4* |
| 1194 | *CBR1* |
| 1195 | *CBR3* |
| 1196 | *CBR3-AS1* |
| 1197 | *CBS* |
| 1198 | *CBSL* |
| 1199 | *CBX1* |
| 1200 | *CBX2* |
| 1201 | *CBX3* |
| 1202 | *CBX4* |
| 1203 | *CBX5* |
| 1204 | *CBX7* |
| 1205 | *CBX8* |
| 1206 | *CBY1* |
| 1207 | *CBY2* |
| 1208 | *CC2D1A* |
| 1209 | *CC2D2A* |
| 1210 | *CCAR1* |
| 1211 | *CCAR2* |
| 1212 | *CCAT1* |
| 1213 | *CCAT2* |
| 1214 | *CCBE1* |
| 1215 | *CCDC107* |
| 1216 | *CCDC116* |
| 1217 | *CCDC12* |
| 1218 | *CCDC12P1* |
| 1219 | *CCDC137* |
| 1220 | *CCDC141* |
| 1221 | *CCDC153* |
| 1222 | *CCDC159* |
| 1223 | *CCDC174* |
| 1224 | *CCDC180* |
| 1225 | *CCDC186* |
| 1226 | *CCDC190* |
| 1227 | *CCDC195* |
| 1228 | *CCDC25* |
| 1229 | *CCDC28B* |
| 1230 | *CCDC34* |
| 1231 | *CCDC43* |
| 1232 | *CCDC6* |
| 1233 | *CCDC61* |
| 1234 | *CCDC62* |
| 1235 | *CCDC63* |
| 1236 | *CCDC66* |
| 1237 | *CCDC68* |
| 1238 | *CCDC69* |
| 1239 | *CCDC7* |
| 1240 | *CCDC80* |
| 1241 | *CCDC88A* |
| 1242 | *CCDC88C* |
| 1243 | *CCDC93* |
| 1244 | *CCDC97* |
| 1245 | *CCDC9B* |
| 1246 | *CCEPR* |
| 1247 | *CCK* |
| 1248 | *CCKAR* |
| 1249 | *CCKBR* |
| 1250 | *CCL1* |
| 1251 | *CCL11* |
| 1252 | *CCL14* |
| 1253 | *CCL15* |
| 1254 | *CCL17* |
| 1255 | *CCL18* |
| 1256 | *CCL19* |
| 1257 | *CCL2* |
| 1258 | *CCL20* |
| 1259 | *CCL21* |
| 1260 | *CCL22* |
| 1261 | *CCL24* |
| 1262 | *CCL25* |
| 1263 | *CCL26* |
| 1264 | *CCL28* |
| 1265 | *CCL3* |
| 1266 | *CCL4* |
| 1267 | *CCL4L1* |
| 1268 | *CCL4L2* |
| 1269 | *CCL5* |
| 1270 | *CCL7* |
| 1271 | *CCM2* |
| 1272 | *CCN1* |
| 1273 | *CCN2* |
| 1274 | *CCN3* |
| 1275 | *CCN4* |
| 1276 | *CCN5* |
| 1277 | *CCN6* |
| 1278 | *CCNA1* |
| 1279 | *CCNA2* |
| 1280 | *CCNB1* |
| 1281 | *CCNB2* |
| 1282 | *CCNB3* |
| 1283 | *CCNC* |
| 1284 | *CCND1* |
| 1285 | *CCND2* |
| 1286 | *CCND2-AS1* |
| 1287 | *CCND3* |
| 1288 | *CCNDBP1* |
| 1289 | *CCNE1* |
| 1290 | *CCNE2* |
| 1291 | *CCNG1* |
| 1292 | *CCNG2* |
| 1293 | *CCNH* |
| 1294 | *CCNI2* |
| 1295 | *CCNK* |
| 1296 | *CCNL2* |
| 1297 | *CCNO* |
| 1298 | *CCNP* |
| 1299 | *CCNY* |
| 1300 | *CCP110* |
| 1301 | *CCR1* |
| 1302 | *CCR2* |
| 1303 | *CCR3* |
| 1304 | *CCR4* |
| 1305 | *CCR5* |
| 1306 | *CCR6* |
| 1307 | *CCR7* |
| 1308 | *CCR8* |
| 1309 | *CCR9* |
| 1310 | *CCS* |
| 1311 | *CCSAP* |
| 1312 | *CCSER1* |
| 1313 | *CCT* |
| 1314 | *CCT2* |
| 1315 | *CCT3* |
| 1316 | *CCT4* |
| 1317 | *CCT5* |
| 1318 | *CCT6A* |
| 1319 | *CCT7* |
| 1320 | *CCT8* |
| 1321 | *CD109* |
| 1322 | *CD14* |
| 1323 | *CD151* |
| 1324 | *CD160* |
| 1325 | *CD163* |
| 1326 | *CD164* |
| 1327 | *CD177* |
| 1328 | *CD1A* |
| 1329 | *CD1D* |
| 1330 | *CD2* |
| 1331 | *CD200* |
| 1332 | *CD207* |
| 1333 | *CD209* |
| 1334 | *CD226* |
| 1335 | *CD24* |
| 1336 | *CD244* |
| 1337 | *CD247* |
| 1338 | *CD248* |
| 1339 | *CD27* |
| 1340 | *CD274* |
| 1341 | *CD276* |
| 1342 | *CD28* |
| 1343 | *CD2AP* |
| 1344 | *CD2BP2* |
| 1345 | *CD300E* |
| 1346 | *CD320* |
| 1347 | *CD33* |
| 1348 | *CD34* |
| 1349 | *CD36* |
| 1350 | *CD38* |
| 1351 | *CD3EAP* |
| 1352 | *CD4* |
| 1353 | *CD40* |
| 1354 | *CD40LG* |
| 1355 | *CD44* |
| 1356 | *CD46* |
| 1357 | *CD47* |
| 1358 | *CD5* |
| 1359 | *CD55* |
| 1360 | *CD58* |
| 1361 | *CD59* |
| 1362 | *CD63* |
| 1363 | *CD68* |
| 1364 | *CD69* |
| 1365 | *CD70* |
| 1366 | *CD74* |
| 1367 | *CD79A* |
| 1368 | *CD80* |
| 1369 | *CD81* |
| 1370 | *CD82* |
| 1371 | *CD83* |
| 1372 | *CD84* |
| 1373 | *CD86* |
| 1374 | *CD8A* |
| 1375 | *CD9* |
| 1376 | *CD93* |
| 1377 | *CD96* |
| 1378 | *CD99* |
| 1379 | *CDA* |
| 1380 | *CDADC1* |
| 1381 | *CDC14A* |
| 1382 | *CDC14B* |
| 1383 | *CDC16* |
| 1384 | *CDC20* |
| 1385 | *CDC23* |
| 1386 | *CDC25A* |
| 1387 | *CDC25B* |
| 1388 | *CDC25C* |
| 1389 | *CDC27* |
| 1390 | *CDC37* |
| 1391 | *CDC40* |
| 1392 | *CDC42* |
| 1393 | *CDC42BPA* |
| 1394 | *CDC42BPB* |
| 1395 | *CDC42BPG* |
| 1396 | *CDC42EP1* |
| 1397 | *CDC42SE2* |
| 1398 | *CDC45* |
| 1399 | *CDC5L* |
| 1400 | *CDC6* |
| 1401 | *CDC7* |
| 1402 | *CDC73* |
| 1403 | *CDCA2* |
| 1404 | *CDCA3* |
| 1405 | *CDCA5* |
| 1406 | *CDCA7* |
| 1407 | *CDCA8* |
| 1408 | *CDCP1* |
| 1409 | *CDH1* |
| 1410 | *CDH10* |
| 1411 | *CDH11* |
| 1412 | *CDH12* |
| 1413 | *CDH13* |
| 1414 | *CDH15* |
| 1415 | *CDH16* |
| 1416 | *CDH17* |
| 1417 | *CDH19* |
| 1418 | *CDH2* |
| 1419 | *CDH20* |
| 1420 | *CDH22* |
| 1421 | *CDH24* |
| 1422 | *CDH3* |
| 1423 | *CDH4* |
| 1424 | *CDH5* |
| 1425 | *CDH7* |
| 1426 | *CDH9* |
| 1427 | *CDHR2* |
| 1428 | *CDHR5* |
| 1429 | *CDIN1* |
| 1430 | *CDK1* |
| 1431 | *CDK10* |
| 1432 | *CDK11A* |
| 1433 | *CDK11B* |
| 1434 | *CDK12* |
| 1435 | *CDK13* |
| 1436 | *CDK14* |
| 1437 | *CDK15* |
| 1438 | *CDK18* |
| 1439 | *CDK19* |
| 1440 | *CDK2* |
| 1441 | *CDK20* |
| 1442 | *CDK2AP1* |
| 1443 | *CDK2AP2* |
| 1444 | *CDK3* |
| 1445 | *CDK4* |
| 1446 | *CDK5* |
| 1447 | *CDK5RAP1* |
| 1448 | *CDK5RAP3* |
| 1449 | *CDK6* |
| 1450 | *CDK7* |
| 1451 | *CDK8* |
| 1452 | *CDK9* |
| 1453 | *CDKAL1* |
| 1454 | *CDKL1* |
| 1455 | *CDKL2* |
| 1456 | *CDKL3* |
| 1457 | *CDKL4* |
| 1458 | *CDKL5* |
| 1459 | *CDKN1A* |
| 1460 | *CDKN1B* |
| 1461 | *CDKN1C* |
| 1462 | *CDKN2A* |
| 1463 | *CDKN2AIP* |
| 1464 | *CDKN2B* |
| 1465 | *CDKN2B-AS1* |
| 1466 | *CDKN2C* |
| 1467 | *CDKN2D* |
| 1468 | *CDKN3* |
| 1469 | *CDO1* |
| 1470 | *CDR1-AS* |
| 1471 | *CDR2* |
| 1472 | *CDR3* |
| 1473 | *CDS1* |
| 1474 | *CDT1* |
| 1475 | *CDV3* |
| 1476 | *CDX1* |
| 1477 | *CDX2* |
| 1478 | *CDX4* |
| 1479 | *CDYL* |
| 1480 | *CEACAM1* |
| 1481 | *CEACAM21* |
| 1482 | *CEACAM3* |
| 1483 | *CEACAM4* |
| 1484 | *CEACAM5* |
| 1485 | *CEACAM6* |
| 1486 | *CEACAM7* |
| 1487 | *CEACAM8* |
| 1488 | *CEBPA* |
| 1489 | *CEBPA-DT* |
| 1490 | *CEBPB* |
| 1491 | *CEBPD* |
| 1492 | *CEBPZ* |
| 1493 | *CELA1* |
| 1494 | *CELF1* |
| 1495 | *CELF2* |
| 1496 | *CELF3* |
| 1497 | *CELF4* |
| 1498 | *CELSR3* |
| 1499 | *CEMIP* |
| 1500 | *CENATAC* |
| 1501 | *CEND1* |
| 1502 | *CENPA* |
| 1503 | *CENPB* |
| 1504 | *CENPC* |
| 1505 | *CENPE* |
| 1506 | *CENPF* |
| 1507 | *CENPH* |
| 1508 | *CENPI* |
| 1509 | *CENPK* |
| 1510 | *CENPT* |
| 1511 | *CENPU* |
| 1512 | *CEP131* |
| 1513 | *CEP135* |
| 1514 | *CEP162* |
| 1515 | *CEP170* |
| 1516 | *CEP192* |
| 1517 | *CEP290* |
| 1518 | *CEP41* |
| 1519 | *CEP55* |
| 1520 | *CEP57L1* |
| 1521 | *CEP63* |
| 1522 | *CEP72* |
| 1523 | *CEP76* |
| 1524 | *CEP78* |
| 1525 | *CEP85L* |
| 1526 | *CEP89* |
| 1527 | *CEPT1* |
| 1528 | *CER1* |
| 1529 | *CERCAM* |
| 1530 | *CERNA3* |
| 1531 | *CERS4* |
| 1532 | *CERS5* |
| 1533 | *CERS6* |
| 1534 | *CES1* |
| 1535 | *CES2* |
| 1536 | *CES3* |
| 1537 | *CETN1* |
| 1538 | *CETN3* |
| 1539 | *CETP* |
| 1540 | *CFAP20DC* |
| 1541 | *CFAP251* |
| 1542 | *CFAP298* |
| 1543 | *CFAP44* |
| 1544 | *CFAP45* |
| 1545 | *CFAP65* |
| 1546 | *CFAP77* |
| 1547 | *CFD* |
| 1548 | *CFHR5* |
| 1549 | *CFI* |
| 1550 | *CFL1* |
| 1551 | *CFL2* |
| 1552 | *CFLAR* |
| 1553 | *CFP* |
| 1554 | *CFTR* |
| 1555 | *CGA* |
| 1556 | *CGAS* |
| 1557 | *CGB3* |
| 1558 | *CGB7* |
| 1559 | *CHAC2* |
| 1560 | *CHAF1A* |
| 1561 | *CHAF1B* |
| 1562 | *CHAT* |
| 1563 | *CHCHD3* |
| 1564 | *CHCHD3P1* |
| 1565 | *CHCHD4* |
| 1566 | *CHCHD7* |
| 1567 | *CHD1* |
| 1568 | *CHD1L* |
| 1569 | *CHD3* |
| 1570 | *CHD4* |
| 1571 | *CHD5* |
| 1572 | *CHD7* |
| 1573 | *CHD8* |
| 1574 | *CHD9* |
| 1575 | *CHD9NB* |
| 1576 | *CHDH* |
| 1577 | *CHEK1* |
| 1578 | *CHEK2* |
| 1579 | *CHERP* |
| 1580 | *CHFR* |
| 1581 | *CHGA* |
| 1582 | *CHGB* |
| 1583 | *CHI3L1* |
| 1584 | *CHIC2* |
| 1585 | *CHIT1* |
| 1586 | *CHKA* |
| 1587 | *CHL1* |
| 1588 | *CHM* |
| 1589 | *CHMP1A* |
| 1590 | *CHMP1B* |
| 1591 | *CHMP2A* |
| 1592 | *CHMP2B* |
| 1593 | *CHMP3* |
| 1594 | *CHMP4A* |
| 1595 | *CHMP5* |
| 1596 | *CHORDC1P5* |
| 1597 | *CHP1* |
| 1598 | *CHPF* |
| 1599 | *CHPF2* |
| 1600 | *CHPT1* |
| 1601 | *CHRDL2* |
| 1602 | *CHRM3* |
| 1603 | *CHRNA5* |
| 1604 | *CHRNA7* |
| 1605 | *CHRND* |
| 1606 | *CHRNE* |
| 1607 | *CHST11* |
| 1608 | *CHST15* |
| 1609 | *CHST2* |
| 1610 | *CHST3* |
| 1611 | *CHST7* |
| 1612 | *CHST8* |
| 1613 | *CHSY1* |
| 1614 | *CHSY3* |
| 1615 | *CHTF18* |
| 1616 | *CHTOP* |
| 1617 | *CHUK* |
| 1618 | *CIAO1* |
| 1619 | *CIAO2A* |
| 1620 | *CIAO3* |
| 1621 | *CIAPIN1* |
| 1622 | *CIB1* |
| 1623 | *CIB2* |
| 1624 | *CIC* |
| 1625 | *CICP17* |
| 1626 | *CIITA* |
| 1627 | *CINP* |
| 1628 | *CIP2A* |
| 1629 | *CIRBP* |
| 1630 | *CISD1* |
| 1631 | *CISD3* |
| 1632 | *CISH* |
| 1633 | *CITED1* |
| 1634 | *CITED2* |
| 1635 | *CITED4* |
| 1636 | *CIZ1* |
| 1637 | *CKAP2* |
| 1638 | *CKAP4* |
| 1639 | *CKAP5* |
| 1640 | *CKB* |
| 1641 | *CKLF* |
| 1642 | *CKM* |
| 1643 | *CKMT1A* |
| 1644 | *CKMT1B* |
| 1645 | *CKMT2* |
| 1646 | *CKS1B* |
| 1647 | *CKS1BP3* |
| 1648 | *CKS1BP7* |
| 1649 | *CKS2* |
| 1650 | *CLASP2* |
| 1651 | *CLASRP* |
| 1652 | *CLC* |
| 1653 | *CLCA1* |
| 1654 | *CLCA2* |
| 1655 | *CLCA4* |
| 1656 | *CLCC1* |
| 1657 | *CLCN1* |
| 1658 | *CLCN3* |
| 1659 | *CLDN1* |
| 1660 | *CLDN10* |
| 1661 | *CLDN11* |
| 1662 | *CLDN12* |
| 1663 | *CLDN14* |
| 1664 | *CLDN15* |
| 1665 | *CLDN16* |
| 1666 | *CLDN17* |
| 1667 | *CLDN18* |
| 1668 | *CLDN19* |
| 1669 | *CLDN2* |
| 1670 | *CLDN20* |
| 1671 | *CLDN22* |
| 1672 | *CLDN23* |
| 1673 | *CLDN24* |
| 1674 | *CLDN3* |
| 1675 | *CLDN4* |
| 1676 | *CLDN5* |
| 1677 | *CLDN6* |
| 1678 | *CLDN7* |
| 1679 | *CLDN8* |
| 1680 | *CLDN9* |
| 1681 | *CLDND1* |
| 1682 | *CLEC10A* |
| 1683 | *CLEC12A* |
| 1684 | *CLEC16A* |
| 1685 | *CLEC2B* |
| 1686 | *CLEC2D* |
| 1687 | *CLEC3A* |
| 1688 | *CLEC3B* |
| 1689 | *CLEC4D* |
| 1690 | *CLEC4G* |
| 1691 | *CLIC1* |
| 1692 | *CLIC4* |
| 1693 | *CLIC5* |
| 1694 | *CLINT1* |
| 1695 | *CLIP1* |
| 1696 | *CLIP2* |
| 1697 | *CLK2* |
| 1698 | *CLK3* |
| 1699 | *CLMAT3* |
| 1700 | *CLMP* |
| 1701 | *CLN3* |
| 1702 | *CLN5* |
| 1703 | *CLN6* |
| 1704 | *CLNS1A* |
| 1705 | *CLOCK* |
| 1706 | *CLP1* |
| 1707 | *CLPB* |
| 1708 | *CLPTM1* |
| 1709 | *CLPTM1L* |
| 1710 | *CLSPN* |
| 1711 | *CLSTN2* |
| 1712 | *CLSTN3* |
| 1713 | *CLTA* |
| 1714 | *CLTC* |
| 1715 | *CLTCL1* |
| 1716 | *CLU* |
| 1717 | *CLUAP1* |
| 1718 | *CLUH* |
| 1719 | *CLXN* |
| 1720 | *CMA1* |
| 1721 | *CMAS* |
| 1722 | *CMC1* |
| 1723 | *CMIP* |
| 1724 | *CMKLR1* |
| 1725 | *CMPK1* |
| 1726 | *CMPK2* |
| 1727 | *CMTM2* |
| 1728 | *CMTM3* |
| 1729 | *CMTM4* |
| 1730 | *CMTM6* |
| 1731 | *CNBP* |
| 1732 | *CNC2* |
| 1733 | *CNGA2* |
| 1734 | *CNGB1* |
| 1735 | *CNGB3* |
| 1736 | *CNKSR1* |
| 1737 | *CNKSR2* |
| 1738 | *CNN1* |
| 1739 | *CNN2* |
| 1740 | *CNN3* |
| 1741 | *CNNM1* |
| 1742 | *CNNM3* |
| 1743 | *CNNM4* |
| 1744 | *CNOT2* |
| 1745 | *CNOT3* |
| 1746 | *CNOT4* |
| 1747 | *CNOT7* |
| 1748 | *CNOT9* |
| 1749 | *CNP* |
| 1750 | *CNPPD1* |
| 1751 | *CNPY1* |
| 1752 | *CNPY2* |
| 1753 | *CNR1* |
| 1754 | *CNR2* |
| 1755 | *CNRIP1* |
| 1756 | *CNTD1* |
| 1757 | *CNTD2* |
| 1758 | *CNTN1* |
| 1759 | *CNTN3* |
| 1760 | *CNTN4* |
| 1761 | *CNTN5* |
| 1762 | *CNTN6* |
| 1763 | *CNTNAP1* |
| 1764 | *CNTROB* |
| 1765 | *COA1* |
| 1766 | *COA3* |
| 1767 | *COASY* |
| 1768 | *COBL* |
| 1769 | *COG2* |
| 1770 | *COG3* |
| 1771 | *COG6* |
| 1772 | *COG8* |
| 1773 | *COIL* |
| 1774 | *COL10A1* |
| 1775 | *COL11A1* |
| 1776 | *COL11A2* |
| 1777 | *COL12A1* |
| 1778 | *COL13A1* |
| 1779 | *COL14A1* |
| 1780 | *COL15A1* |
| 1781 | *COL16A1* |
| 1782 | *COL17A1* |
| 1783 | *COL18A1* |
| 1784 | *COL19A1* |
| 1785 | *COL1A1* |
| 1786 | *COL1A2* |
| 1787 | *COL20A1* |
| 1788 | *COL21A1* |
| 1789 | *COL23A1* |
| 1790 | *COL24A1* |
| 1791 | *COL25A1* |
| 1792 | *COL27A1* |
| 1793 | *COL28A1* |
| 1794 | *COL2A1* |
| 1795 | *COL3A1* |
| 1796 | *COL4A1* |
| 1797 | *COL4A2* |
| 1798 | *COL4A3* |
| 1799 | *COL4A4* |
| 1800 | *COL4A5* |
| 1801 | *COL4A6* |
| 1802 | *COL5A1* |
| 1803 | *COL5A2* |
| 1804 | *COL6A1* |
| 1805 | *COL6A2* |
| 1806 | *COL6A3* |
| 1807 | *COL6A6* |
| 1808 | *COL7A1* |
| 1809 | *COL8A1* |
| 1810 | *COL8A2* |
| 1811 | *COL9A1* |
| 1812 | *COL9A2* |
| 1813 | *COL9A3* |
| 1814 | *COLCA1* |
| 1815 | *COLCA2* |
| 1816 | *COLEC12* |
| 1817 | *COLGALT2* |
| 1818 | *COLQ* |
| 1819 | *COMETT* |
| 1820 | *COMMD10* |
| 1821 | *COMMD3-BMI1* |
| 1822 | *COMP* |
| 1823 | *COMT* |
| 1824 | *COMTD1* |
| 1825 | *COP1* |
| 1826 | *COPA* |
| 1827 | *COPB1* |
| 1828 | *COPB2* |
| 1829 | *COPD* |
| 1830 | *COPE* |
| 1831 | *COPG1* |
| 1832 | *COPG2* |
| 1833 | *COPRS* |
| 1834 | *COPS2* |
| 1835 | *COPS3* |
| 1836 | *COPS4* |
| 1837 | *COPS5* |
| 1838 | *COPS6* |
| 1839 | *COPS7B* |
| 1840 | *COPS8* |
| 1841 | *COPZ1* |
| 1842 | *COQ2* |
| 1843 | *COQ6* |
| 1844 | *COQ7* |
| 1845 | *COQ8A* |
| 1846 | *COQ8B* |
| 1847 | *CORO1A* |
| 1848 | *CORO1B* |
| 1849 | *CORO1C* |
| 1850 | *CORO2A* |
| 1851 | *CORO2B* |
| 1852 | *CORO6* |
| 1853 | *CORO7* |
| 1854 | *COTL1* |
| 1855 | *COX1* |
| 1856 | *COX10* |
| 1857 | *COX15* |
| 1858 | *COX2* |
| 1859 | *COX4I1* |
| 1860 | *COX4I2* |
| 1861 | *COX5A* |
| 1862 | *COX6B1* |
| 1863 | *COX6B1P1* |
| 1864 | *COX6C* |
| 1865 | *COX6CP2* |
| 1866 | *COX7A2* |
| 1867 | *COX7A2L* |
| 1868 | *COX8A* |
| 1869 | *CP* |
| 1870 | *CPA4* |
| 1871 | *CPA5* |
| 1872 | *CPAMD8* |
| 1873 | *CPD* |
| 1874 | *CPE* |
| 1875 | *CPEB1* |
| 1876 | *CPEB2* |
| 1877 | *CPEB3* |
| 1878 | *CPEB4* |
| 1879 | *CPLANE1* |
| 1880 | *CPM* |
| 1881 | *CPNE1* |
| 1882 | *CPNE3* |
| 1883 | *CPNE8* |
| 1884 | *CPO* |
| 1885 | *CPOX* |
| 1886 | *CPQ* |
| 1887 | *CPS1* |
| 1888 | *CPS1-IT1* |
| 1889 | *CPSF1* |
| 1890 | *CPSF2* |
| 1891 | *CPSF3* |
| 1892 | *CPSF4* |
| 1893 | *CPSF6* |
| 1894 | *CPSF7* |
| 1895 | *CPT1A* |
| 1896 | *CPT1B* |
| 1897 | *CPT2* |
| 1898 | *CPVL* |
| 1899 | *CR1* |
| 1900 | *CR2* |
| 1901 | *CRABP1* |
| 1902 | *CRABP2* |
| 1903 | *CRACD* |
| 1904 | *CRACDL* |
| 1905 | *CRAT* |
| 1906 | *CRB1* |
| 1907 | *CRB3* |
| 1908 | *CRBN* |
| 1909 | *CRCS10* |
| 1910 | *CRCS11* |
| 1911 | *CRCS2* |
| 1912 | *CRCS5* |
| 1913 | *CRCS6* |
| 1914 | *CRCS7* |
| 1915 | *CRCS8* |
| 1916 | *CRCS9* |
| 1917 | *CREB1* |
| 1918 | *CREB5* |
| 1919 | *CREBBP* |
| 1920 | *CREBZF* |
| 1921 | *CRH* |
| 1922 | *CRHR1* |
| 1923 | *CRHR2* |
| 1924 | *CRIM1* |
| 1925 | *CRIP1* |
| 1926 | *CRIP2* |
| 1927 | *CRIPT* |
| 1928 | *CRISP2* |
| 1929 | *CRISPLD2* |
| 1930 | *CRK* |
| 1931 | *CRKL* |
| 1932 | *CRLF2* |
| 1933 | *CRLS1* |
| 1934 | *CRMP1* |
| 1935 | *CRNDE* |
| 1936 | *CRP* |
| 1937 | *CRTAP* |
| 1938 | *CRTC1* |
| 1939 | *CRTC1P1* |
| 1940 | *CRTC2* |
| 1941 | *CRTC3* |
| 1942 | *CRTC3-AS1* |
| 1943 | *CRX* |
| 1944 | *CRY1* |
| 1945 | *CRY2* |
| 1946 | *CRYAA* |
| 1947 | *CRYAB* |
| 1948 | *CRYBG2* |
| 1949 | *CRYGC* |
| 1950 | *CRYGEP* |
| 1951 | *CRYZ* |
| 1952 | *CRYZL2P* |
| 1953 | *CS* |
| 1954 | *CSAG2* |
| 1955 | *CSAG3* |
| 1956 | *CSDE1* |
| 1957 | *CSE1L* |
| 1958 | *CSF1* |
| 1959 | *CSF1R* |
| 1960 | *CSF2* |
| 1961 | *CSF2RB* |
| 1962 | *CSF3* |
| 1963 | *CSF3R* |
| 1964 | *CSK* |
| 1965 | *CSMD1* |
| 1966 | *CSMD2* |
| 1967 | *CSMD3* |
| 1968 | *CSN1S1* |
| 1969 | *CSN2* |
| 1970 | *CSNK1A1* |
| 1971 | *CSNK1A1L* |
| 1972 | *CSNK1D* |
| 1973 | *CSNK1E* |
| 1974 | *CSNK1G2* |
| 1975 | *CSNK2A1* |
| 1976 | *CSNK2A2* |
| 1977 | *CSNK2A3* |
| 1978 | *CSNK2B* |
| 1979 | *CSPG4* |
| 1980 | *CSPG4P10* |
| 1981 | *CSRNP1* |
| 1982 | *CSRNP3* |
| 1983 | *CSRP1* |
| 1984 | *CSRP2* |
| 1985 | *CST1* |
| 1986 | *CST3* |
| 1987 | *CST4* |
| 1988 | *CST5* |
| 1989 | *CST7* |
| 1990 | *CSTA* |
| 1991 | *CSTB* |
| 1992 | *CT45A1* |
| 1993 | *CT47A1* |
| 1994 | *CT47A10* |
| 1995 | *CT47A11* |
| 1996 | *CT47A12* |
| 1997 | *CT47A2* |
| 1998 | *CT47A3* |
| 1999 | *CT47A4* |
| 2000 | *CT47A5* |
| 2001 | *CT47A6* |
| 2002 | *CT47A7* |
| 2003 | *CT47A8* |
| 2004 | *CT47A9* |
| 2005 | *CT47B1* |
| 2006 | *CT55* |
| 2007 | *CTAG1A* |
| 2008 | *CTAG1B* |
| 2009 | *CTAG2* |
| 2010 | *CTAGE4* |
| 2011 | *CTBP1* |
| 2012 | *CTBP2* |
| 2013 | *CTCF* |
| 2014 | *CTCFL* |
| 2015 | *CTDNEP1* |
| 2016 | *CTDP1* |
| 2017 | *CTDSP1* |
| 2018 | *CTDSP2* |
| 2019 | *CTDSPL2* |
| 2020 | *CTHRC1* |
| 2021 | *CTIF* |
| 2022 | *CTLA4* |
| 2023 | *CTNNA1* |
| 2024 | *CTNNA1P1* |
| 2025 | *CTNNA2* |
| 2026 | *CTNNA3* |
| 2027 | *CTNNB1* |
| 2028 | *CTNNBIP1* |
| 2029 | *CTNNBL1* |
| 2030 | *CTNND1* |
| 2031 | *CTNND2* |
| 2032 | *CTPS1* |
| 2033 | *CTPS2* |
| 2034 | *CTR9* |
| 2035 | *CTSA* |
| 2036 | *CTSB* |
| 2037 | *CTSC* |
| 2038 | *CTSD* |
| 2039 | *CTSE* |
| 2040 | *CTSF* |
| 2041 | *CTSG* |
| 2042 | *CTSH* |
| 2043 | *CTSK* |
| 2044 | *CTSL* |
| 2045 | *CTSS* |
| 2046 | *CTSV* |
| 2047 | *CTSZ* |
| 2048 | *CTTN* |
| 2049 | *CUBN* |
| 2050 | *CUEDC2* |
| 2051 | *CUL1* |
| 2052 | *CUL2* |
| 2053 | *CUL3* |
| 2054 | *CUL4A* |
| 2055 | *CUL4B* |
| 2056 | *CUL5* |
| 2057 | *CUL7* |
| 2058 | *CUL9* |
| 2059 | *CUTC* |
| 2060 | *CUX1* |
| 2061 | *CUX2* |
| 2062 | *CUZD1* |
| 2063 | *CWC25* |
| 2064 | *CWH43* |
| 2065 | *CX3CL1* |
| 2066 | *CX3CR1* |
| 2067 | *CXADR* |
| 2068 | *CXADRP1* |
| 2069 | *CXCL1* |
| 2070 | *CXCL10* |
| 2071 | *CXCL11* |
| 2072 | *CXCL12* |
| 2073 | *CXCL13* |
| 2074 | *CXCL14* |
| 2075 | *CXCL16* |
| 2076 | *CXCL17* |
| 2077 | *CXCL2* |
| 2078 | *CXCL3* |
| 2079 | *CXCL5* |
| 2080 | *CXCL6* |
| 2081 | *CXCL8* |
| 2082 | *CXCL9* |
| 2083 | *CXCR1* |
| 2084 | *CXCR2* |
| 2085 | *CXCR3* |
| 2086 | *CXCR4* |
| 2087 | *CXCR5* |
| 2088 | *CXCR6* |
| 2089 | *CXXC1* |
| 2090 | *CXXC4* |
| 2091 | *CYB561A3* |
| 2092 | *CYB5A* |
| 2093 | *CYB5B* |
| 2094 | *CYB5D2* |
| 2095 | *CYB5R1* |
| 2096 | *CYB5R2* |
| 2097 | *CYB5R4* |
| 2098 | *CYBA* |
| 2099 | *CYBB* |
| 2100 | *CYBRD1* |
| 2101 | *CYC1* |
| 2102 | *CYCS* |
| 2103 | *CYCSP51* |
| 2104 | *CYFIP1* |
| 2105 | *CYFIP2* |
| 2106 | *CYGB* |
| 2107 | *CYLC2* |
| 2108 | *CYLD* |
| 2109 | *CYP11A1* |
| 2110 | *CYP17A1* |
| 2111 | *CYP19A1* |
| 2112 | *CYP1A1* |
| 2113 | *CYP1A2* |
| 2114 | *CYP1B1* |
| 2115 | *CYP21A1P* |
| 2116 | *CYP24A1* |
| 2117 | *CYP26B1* |
| 2118 | *CYP27A1* |
| 2119 | *CYP27B1* |
| 2120 | *CYP2A6* |
| 2121 | *CYP2B6* |
| 2122 | *CYP2C18* |
| 2123 | *CYP2C19* |
| 2124 | *CYP2C8* |
| 2125 | *CYP2C9* |
| 2126 | *CYP2D6* |
| 2127 | *CYP2E1* |
| 2128 | *CYP2F1* |
| 2129 | *CYP2G1P* |
| 2130 | *CYP2R1* |
| 2131 | *CYP2S1* |
| 2132 | *CYP2T1P* |
| 2133 | *CYP2W1* |
| 2134 | *CYP3A4* |
| 2135 | *CYP3A5* |
| 2136 | *CYP3A7* |
| 2137 | *CYP46A1* |
| 2138 | *CYP4A11* |
| 2139 | *CYP4F3* |
| 2140 | *CYP51A1* |
| 2141 | *CYP7A1* |
| 2142 | *CYP7B1* |
| 2143 | *CYREN* |
| 2144 | *CYRIB* |
| 2145 | *CYSLTR1* |
| 2146 | *CYSLTR2* |
| 2147 | *CYTB* |
| 2148 | *CYTH1* |
| 2149 | *CYTH2* |
| 2150 | *CYTH3* |
| 2151 | *CYTIP* |
| 2152 | *CYTL1* |
| 2153 | *CYTOR* |
| 2154 | *DAAM1* |
| 2155 | *DAAM2* |
| 2156 | *DAB1* |
| 2157 | *DAB2* |
| 2158 | *DAB2IP* |
| 2159 | *DACH1* |
| 2160 | *DACT1* |
| 2161 | *DACT2* |
| 2162 | *DACT3* |
| 2163 | *DAD1* |
| 2164 | *DAG1* |
| 2165 | *DALRD3* |
| 2166 | *DANCR* |
| 2167 | *DAND5* |
| 2168 | *DAP* |
| 2169 | *DAP3* |
| 2170 | *DAPK1* |
| 2171 | *DAPK2* |
| 2172 | *DAPK3* |
| 2173 | *DARS1* |
| 2174 | *DAW1* |
| 2175 | *DAXX* |
| 2176 | *DAZAP1* |
| 2177 | *DBF4B* |
| 2178 | *DBH* |
| 2179 | *DBN1* |
| 2180 | *DBNDD2* |
| 2181 | *DBNL* |
| 2182 | *DBP* |
| 2183 | *DCAF1* |
| 2184 | *DCAF12* |
| 2185 | *DCAF13* |
| 2186 | *DCAF15* |
| 2187 | *DCAF4* |
| 2188 | *DCAF4L2* |
| 2189 | *DCAF7* |
| 2190 | *DCAF8* |
| 2191 | *DCANP1* |
| 2192 | *DCBLD1* |
| 2193 | *DCBLD2* |
| 2194 | *DCC* |
| 2195 | *DCD* |
| 2196 | *DCHS1* |
| 2197 | *DCHS2* |
| 2198 | *DCK* |
| 2199 | *DCLK1* |
| 2200 | *DCLK2* |
| 2201 | *DCLK3* |
| 2202 | *DCLRE1A* |
| 2203 | *DCLRE1B* |
| 2204 | *DCLRE1C* |
| 2205 | *DCN* |
| 2206 | *DCP1A* |
| 2207 | *DCP2* |
| 2208 | *DCT* |
| 2209 | *DCTN1* |
| 2210 | *DCTN2* |
| 2211 | *DCTN4* |
| 2212 | *DCTN6* |
| 2213 | *DCTPP1* |
| 2214 | *DCUN1D1* |
| 2215 | *DCUN1D5* |
| 2216 | *DCXR* |
| 2217 | *DDA1* |
| 2218 | *DDAH1* |
| 2219 | *DDB1* |
| 2220 | *DDB2* |
| 2221 | *DDC* |
| 2222 | *DDHD1* |
| 2223 | *DDI1* |
| 2224 | *DDIAS* |
| 2225 | *DDIT3* |
| 2226 | *DDIT4* |
| 2227 | *DDO* |
| 2228 | *DDR1* |
| 2229 | *DDR2* |
| 2230 | *DDT* |
| 2231 | *DDX1* |
| 2232 | *DDX10* |
| 2233 | *DDX11* |
| 2234 | *DDX11-AS1* |
| 2235 | *DDX11L8* |
| 2236 | *DDX12P* |
| 2237 | *DDX17* |
| 2238 | *DDX18* |
| 2239 | *DDX19A* |
| 2240 | *DDX19B* |
| 2241 | *DDX20* |
| 2242 | *DDX21* |
| 2243 | *DDX23* |
| 2244 | *DDX24* |
| 2245 | *DDX25* |
| 2246 | *DDX27* |
| 2247 | *DDX28* |
| 2248 | *DDX31* |
| 2249 | *DDX39A* |
| 2250 | *DDX39B* |
| 2251 | *DDX3X* |
| 2252 | *DDX41* |
| 2253 | *DDX42* |
| 2254 | *DDX46* |
| 2255 | *DDX47* |
| 2256 | *DDX5* |
| 2257 | *DDX50* |
| 2258 | *DDX52* |
| 2259 | *DDX53* |
| 2260 | *DDX56* |
| 2261 | *DDX58* |
| 2262 | *DDX59* |
| 2263 | *DDX6* |
| 2264 | *DEAF1* |
| 2265 | *DECR1* |
| 2266 | *DEDD2* |
| 2267 | *DEF6* |
| 2268 | *DEFA1* |
| 2269 | *DEFA1B* |
| 2270 | *DEFA3* |
| 2271 | *DEFA4* |
| 2272 | *DEFA5* |
| 2273 | *DEFA6* |
| 2274 | *DEFB1* |
| 2275 | *DEFB109B* |
| 2276 | *DEFB126* |
| 2277 | *DEFB4A* |
| 2278 | *DEGS1* |
| 2279 | *DEGS2* |
| 2280 | *DEK* |
| 2281 | *DENND10* |
| 2282 | *DENND4A* |
| 2283 | *DENND4C* |
| 2284 | *DENND5B* |
| 2285 | *DENR* |
| 2286 | *DEPDC1* |
| 2287 | *DEPP1* |
| 2288 | *DEPTOR* |
| 2289 | *DERL1* |
| 2290 | *DERL2* |
| 2291 | *DES* |
| 2292 | *DESI1* |
| 2293 | *DESI2* |
| 2294 | *DEXI* |
| 2295 | *DFFA* |
| 2296 | *DFFB* |
| 2297 | *DGCR2* |
| 2298 | *DGCR5* |
| 2299 | *DGCR8* |
| 2300 | *DGKB* |
| 2301 | *DGKD* |
| 2302 | *DGKE* |
| 2303 | *DGKG* |
| 2304 | *DGKH* |
| 2305 | *DGKZ* |
| 2306 | *DGUOK* |
| 2307 | *DHCR24* |
| 2308 | *DHCR7* |
| 2309 | *DHDDS* |
| 2310 | *DHFR* |
| 2311 | *DHODH* |
| 2312 | *DHPS* |
| 2313 | *DHRS11* |
| 2314 | *DHRS2* |
| 2315 | *DHRS9* |
| 2316 | *DHX15* |
| 2317 | *DHX16* |
| 2318 | *DHX29* |
| 2319 | *DHX30* |
| 2320 | *DHX32* |
| 2321 | *DHX33* |
| 2322 | *DHX35* |
| 2323 | *DHX36* |
| 2324 | *DHX38* |
| 2325 | *DHX58* |
| 2326 | *DHX9* |
| 2327 | *DIABLO* |
| 2328 | *DIAPH1* |
| 2329 | *DIAPH3* |
| 2330 | *DICER1* |
| 2331 | *DIDO1* |
| 2332 | *DIMT1* |
| 2333 | *DIO2* |
| 2334 | *DIP* |
| 2335 | *DIP2A* |
| 2336 | *DIP2B* |
| 2337 | *DIP2C* |
| 2338 | *DIRAS1* |
| 2339 | *DIRAS2* |
| 2340 | *DIS3* |
| 2341 | *DIS3L* |
| 2342 | *DIS3L2* |
| 2343 | *DIXDC1* |
| 2344 | *DKC1* |
| 2345 | *DKK1* |
| 2346 | *DKK2* |
| 2347 | *DKK3* |
| 2348 | *DKK4* |
| 2349 | *DKKL1* |
| 2350 | *DLC1* |
| 2351 | *DLD* |
| 2352 | *DLEC1* |
| 2353 | *DLEU1* |
| 2354 | *DLEU2* |
| 2355 | *DLEU7-AS1* |
| 2356 | *DLG1* |
| 2357 | *DLG2* |
| 2358 | *DLG3* |
| 2359 | *DLG4* |
| 2360 | *DLG5* |
| 2361 | *DLGAP2* |
| 2362 | *DLGAP5* |
| 2363 | *DLK1* |
| 2364 | *DLL1* |
| 2365 | *DLL3* |
| 2366 | *DLL4* |
| 2367 | *DLST* |
| 2368 | *DLX6* |
| 2369 | *DLX6-AS1* |
| 2370 | *DMBT1* |
| 2371 | *DMD* |
| 2372 | *DMKN* |
| 2373 | *DMPK* |
| 2374 | *DMRT2* |
| 2375 | *DMRTA1* |
| 2376 | *DMTF1* |
| 2377 | *DMTN* |
| 2378 | *DMWD* |
| 2379 | *DNAAF4* |
| 2380 | *DNAAF5* |
| 2381 | *DNAAF6* |
| 2382 | *DNAH14* |
| 2383 | *DNAH17-AS1* |
| 2384 | *DNAH3* |
| 2385 | *DNAH8* |
| 2386 | *DNAH9* |
| 2387 | *DNAJA1* |
| 2388 | *DNAJA2* |
| 2389 | *DNAJA3* |
| 2390 | *DNAJB1* |
| 2391 | *DNAJB11* |
| 2392 | *DNAJB12* |
| 2393 | *DNAJB1P1* |
| 2394 | *DNAJB2* |
| 2395 | *DNAJB4* |
| 2396 | *DNAJB6* |
| 2397 | *DNAJB8* |
| 2398 | *DNAJC10* |
| 2399 | *DNAJC13* |
| 2400 | *DNAJC2* |
| 2401 | *DNAJC21* |
| 2402 | *DNAJC24* |
| 2403 | *DNAJC25* |
| 2404 | *DNAJC27* |
| 2405 | *DNAJC3* |
| 2406 | *DNAJC4* |
| 2407 | *DNAJC6* |
| 2408 | *DNAJC7* |
| 2409 | *DNALI1* |
| 2410 | *DNASE1* |
| 2411 | *DNASE1L3* |
| 2412 | *DNASE2* |
| 2413 | *DND1* |
| 2414 | *DNER* |
| 2415 | *DNM1* |
| 2416 | *DNM1L* |
| 2417 | *DNM2* |
| 2418 | *DNM3* |
| 2419 | *DNMBP* |
| 2420 | *DNMT1* |
| 2421 | *DNMT3A* |
| 2422 | *DNMT3AP1* |
| 2423 | *DNMT3B* |
| 2424 | *DNPEP* |
| 2425 | *DNTT* |
| 2426 | *DOCK1* |
| 2427 | *DOCK2* |
| 2428 | *DOCK3* |
| 2429 | *DOCK4* |
| 2430 | *DOCK5* |
| 2431 | *DOCK7* |
| 2432 | *DOCK9* |
| 2433 | *DOK1* |
| 2434 | *DOK2* |
| 2435 | *DOK3* |
| 2436 | *DOLK* |
| 2437 | *DOLPP1* |
| 2438 | *DONSON* |
| 2439 | *DOP1A* |
| 2440 | *DOT1L* |
| 2441 | *DPAGT1* |
| 2442 | *DPEP1* |
| 2443 | *DPF2* |
| 2444 | *DPH1* |
| 2445 | *DPM1* |
| 2446 | *DPP10* |
| 2447 | *DPP10-AS1* |
| 2448 | *DPP3* |
| 2449 | *DPP4* |
| 2450 | *DPP6* |
| 2451 | *DPP7* |
| 2452 | *DPP8* |
| 2453 | *DPP9* |
| 2454 | *DPPA2* |
| 2455 | *DPPA4* |
| 2456 | *DPT* |
| 2457 | *DPYD* |
| 2458 | *DPYS* |
| 2459 | *DPYSL2* |
| 2460 | *DPYSL5* |
| 2461 | *DR1* |
| 2462 | *DRAM2* |
| 2463 | *DRAP1* |
| 2464 | *DRC3* |
| 2465 | *DRD2* |
| 2466 | *DRD3* |
| 2467 | *DRD4* |
| 2468 | *DRG1* |
| 2469 | *DROSHA* |
| 2470 | *DSC1* |
| 2471 | *DSC2* |
| 2472 | *DSC3* |
| 2473 | *DSCAM* |
| 2474 | *DSCAM-AS1* |
| 2475 | *DSCAML1* |
| 2476 | *DSCC1* |
| 2477 | *DSE* |
| 2478 | *DSEL* |
| 2479 | *DSG1* |
| 2480 | *DSG2* |
| 2481 | *DSN1* |
| 2482 | *DSP* |
| 2483 | *DSPP* |
| 2484 | *DST* |
| 2485 | *DSTN* |
| 2486 | *DTL* |
| 2487 | *DTWD1* |
| 2488 | *DTWD2* |
| 2489 | *DTX2P1-UPK3BP1-PMS2P11* |
| 2490 | *DTX3* |
| 2491 | *DTX3L* |
| 2492 | *DUOX2* |
| 2493 | *DUS2* |
| 2494 | *DUSP1* |
| 2495 | *DUSP10* |
| 2496 | *DUSP13* |
| 2497 | *DUSP14* |
| 2498 | *DUSP16* |
| 2499 | *DUSP19* |
| 2500 | *DUSP2* |
| 2501 | *DUSP21* |
| 2502 | *DUSP22* |
| 2503 | *DUSP23* |
| 2504 | *DUSP28* |
| 2505 | *DUSP4* |
| 2506 | *DUSP5* |
| 2507 | *DUSP6* |
| 2508 | *DUSP8* |
| 2509 | *DUSP9* |
| 2510 | *DUT* |
| 2511 | *DUXAP10* |
| 2512 | *DUXAP8* |
| 2513 | *DUXAP9* |
| 2514 | *DVL1* |
| 2515 | *DVL1P1* |
| 2516 | *DVL2* |
| 2517 | *DVL3* |
| 2518 | *DXO* |
| 2519 | *DYNC1H1* |
| 2520 | *DYNC1I2* |
| 2521 | *DYNC1LI1* |
| 2522 | *DYNC2H1* |
| 2523 | *DYNC2I1* |
| 2524 | *DYNLL1* |
| 2525 | *DYNLRB1* |
| 2526 | *DYNLRB2* |
| 2527 | *DYRK1A* |
| 2528 | *DYRK1B* |
| 2529 | *DYRK2* |
| 2530 | *DYSF* |
| 2531 | *E2F1* |
| 2532 | *E2F2* |
| 2533 | *E2F3* |
| 2534 | *E2F4* |
| 2535 | *E2F5* |
| 2536 | *E2F7* |
| 2537 | *E2F8* |
| 2538 | *EAF2* |
| 2539 | *EARS2* |
| 2540 | *EBAG9* |
| 2541 | *EBAG9P1* |
| 2542 | *EBF1* |
| 2543 | *EBF3* |
| 2544 | *EBI3* |
| 2545 | *EBNA1BP2* |
| 2546 | *EBP* |
| 2547 | *ECE1* |
| 2548 | *ECH1* |
| 2549 | *ECHS1* |
| 2550 | *ECM1* |
| 2551 | *ECPAS* |
| 2552 | *ECRG4* |
| 2553 | *ECSIT* |
| 2554 | *ECT2* |
| 2555 | *ECT2L* |
| 2556 | *EDA* |
| 2557 | *EDA2R* |
| 2558 | *EDAR* |
| 2559 | *EDC3* |
| 2560 | *EDC4* |
| 2561 | *EDEM3* |
| 2562 | *EDN1* |
| 2563 | *EDN2* |
| 2564 | *EDN3* |
| 2565 | *EDNRA* |
| 2566 | *EDNRB* |
| 2567 | *EDRF1* |
| 2568 | *EED* |
| 2569 | *EEF1A1* |
| 2570 | *EEF1A1P5* |
| 2571 | *EEF1A2* |
| 2572 | *EEF1B2* |
| 2573 | *EEF1B2P2* |
| 2574 | *EEF1D* |
| 2575 | *EEF1E1* |
| 2576 | *EEF1G* |
| 2577 | *EEF2* |
| 2578 | *EEF2K* |
| 2579 | *EFCAB13* |
| 2580 | *EFCAB2* |
| 2581 | *EFCAB9* |
| 2582 | *EFEMP1* |
| 2583 | *EFEMP2* |
| 2584 | *EFHC1* |
| 2585 | *EFHD1* |
| 2586 | *EFHD2* |
| 2587 | *EFNA1* |
| 2588 | *EFNA2* |
| 2589 | *EFNA3* |
| 2590 | *EFNA4* |
| 2591 | *EFNA5* |
| 2592 | *EFNB1* |
| 2593 | *EFNB2* |
| 2594 | *EFNB3* |
| 2595 | *EFS* |
| 2596 | *EFTUD2* |
| 2597 | *EGF* |
| 2598 | *EGFL6* |
| 2599 | *EGFL7* |
| 2600 | *EGFL8* |
| 2601 | *EGFR* |
| 2602 | *EGLN1* |
| 2603 | *EGLN2* |
| 2604 | *EGLN3* |
| 2605 | *EGR1* |
| 2606 | *EGR2* |
| 2607 | *EHBP1* |
| 2608 | *EHD1* |
| 2609 | *EHD2* |
| 2610 | *EHD4* |
| 2611 | *EHHADH* |
| 2612 | *EHMT1* |
| 2613 | *EHMT2* |
| 2614 | *EI24* |
| 2615 | *EIF1* |
| 2616 | *EIF1AX* |
| 2617 | *EIF1AY* |
| 2618 | *EIF2A* |
| 2619 | *EIF2AK2* |
| 2620 | *EIF2AK3* |
| 2621 | *EIF2AK4* |
| 2622 | *EIF2B2* |
| 2623 | *EIF2B4* |
| 2624 | *EIF2B5* |
| 2625 | *EIF2S1* |
| 2626 | *EIF2S2* |
| 2627 | *EIF2S3* |
| 2628 | *EIF3A* |
| 2629 | *EIF3B* |
| 2630 | *EIF3C* |
| 2631 | *EIF3CL* |
| 2632 | *EIF3D* |
| 2633 | *EIF3E* |
| 2634 | *EIF3F* |
| 2635 | *EIF3G* |
| 2636 | *EIF3H* |
| 2637 | *EIF3I* |
| 2638 | *EIF3J* |
| 2639 | *EIF3J-DT* |
| 2640 | *EIF3K* |
| 2641 | *EIF3L* |
| 2642 | *EIF3M* |
| 2643 | *EIF4A1* |
| 2644 | *EIF4A2* |
| 2645 | *EIF4A3* |
| 2646 | *EIF4B* |
| 2647 | *EIF4BP2* |
| 2648 | *EIF4E* |
| 2649 | *EIF4E3* |
| 2650 | *EIF4EBP1* |
| 2651 | *EIF4EBP2* |
| 2652 | *EIF4EBP3* |
| 2653 | *EIF4ENIF1* |
| 2654 | *EIF4G1* |
| 2655 | *EIF4G2* |
| 2656 | *EIF4G3* |
| 2657 | *EIF4H* |
| 2658 | *EIF5* |
| 2659 | *EIF5A* |
| 2660 | *EIF5A2* |
| 2661 | *EIF5AL1* |
| 2662 | *EIF5B* |
| 2663 | *EIF6* |
| 2664 | *ELAC2* |
| 2665 | *ELANE* |
| 2666 | *ELAPOR1* |
| 2667 | *ELAVL1* |
| 2668 | *ELAVL2* |
| 2669 | *ELF2* |
| 2670 | *ELF3* |
| 2671 | *ELFN1-AS1* |
| 2672 | *ELK1* |
| 2673 | *ELK3* |
| 2674 | *ELK4* |
| 2675 | *ELMO2* |
| 2676 | *ELMO3* |
| 2677 | *ELMOD3* |
| 2678 | *ELN* |
| 2679 | *ELOA* |
| 2680 | *ELOA2* |
| 2681 | *ELOB* |
| 2682 | *ELOC* |
| 2683 | *ELOCP20* |
| 2684 | *ELOF1* |
| 2685 | *ELOVL1* |
| 2686 | *ELOVL5* |
| 2687 | *ELP1* |
| 2688 | *ELP2* |
| 2689 | *ELP3* |
| 2690 | *ELP6* |
| 2691 | *EMC2* |
| 2692 | *EMC4* |
| 2693 | *EMD* |
| 2694 | *EMG1* |
| 2695 | *EMILIN1* |
| 2696 | *EMILIN2* |
| 2697 | *EMILIN3* |
| 2698 | *EML1* |
| 2699 | *EML2* |
| 2700 | *EML3* |
| 2701 | *EML4* |
| 2702 | *EMP1* |
| 2703 | *EMSY* |
| 2704 | *EMX2* |
| 2705 | *EN1* |
| 2706 | *EN2* |
| 2707 | *ENAH* |
| 2708 | *ENC1* |
| 2709 | *ENDOG* |
| 2710 | *ENDOV* |
| 2711 | *ENG* |
| 2712 | *ENKD1* |
| 2713 | *ENKUR* |
| 2714 | *ENO1* |
| 2715 | *ENO2* |
| 2716 | *ENO3* |
| 2717 | *ENOPH1* |
| 2718 | *ENOSF1* |
| 2719 | *ENPEP* |
| 2720 | *ENPP1* |
| 2721 | *ENPP2* |
| 2722 | *ENPP7* |
| 2723 | *ENSG00000124593* |
| 2724 | *ENSG00000213587* |
| 2725 | *ENSG00000225507* |
| 2726 | *ENSG00000225513* |
| 2727 | *ENSG00000225864* |
| 2728 | *ENSG00000226570* |
| 2729 | *ENSG00000227766* |
| 2730 | *ENSG00000227775* |
| 2731 | *ENSG00000229399* |
| 2732 | *ENSG00000230092* |
| 2733 | *ENSG00000230521* |
| 2734 | *ENSG00000234017* |
| 2735 | *ENSG00000235007* |
| 2736 | *ENSG00000235286* |
| 2737 | *ENSG00000237669* |
| 2738 | *ENSG00000251473* |
| 2739 | *ENSG00000255730* |
| 2740 | *ENSG00000255836* |
| 2741 | *ENSG00000265136* |
| 2742 | *ENSG00000269737* |
| 2743 | *ENSG00000279024* |
| 2744 | *ENSG00000279378* |
| 2745 | *ENSG00000280173* |
| 2746 | *ENSG00000280183* |
| 2747 | *ENSG00000282218* |
| 2748 | *ENSG00000285713* |
| 2749 | *ENSG00000285868* |
| 2750 | *ENSG00000285901* |
| 2751 | *ENSG00000290180* |
| 2752 | *ENTPD1* |
| 2753 | *ENTPD5* |
| 2754 | *ENTPD7* |
| 2755 | *ENTREP3* |
| 2756 | *ENY2* |
| 2757 | *EOMES* |
| 2758 | *EP300* |
| 2759 | *EP300-AS1* |
| 2760 | *EP400* |
| 2761 | *EPAS1* |
| 2762 | *EPB41* |
| 2763 | *EPB41L2* |
| 2764 | *EPB41L3* |
| 2765 | *EPB41L4B* |
| 2766 | *EPB41L5* |
| 2767 | *EPCAM* |
| 2768 | *EPDR1* |
| 2769 | *EPG5* |
| 2770 | *EPGN* |
| 2771 | *EPHA1* |
| 2772 | *EPHA2* |
| 2773 | *EPHA3* |
| 2774 | *EPHA4* |
| 2775 | *EPHA5* |
| 2776 | *EPHA6* |
| 2777 | *EPHA7* |
| 2778 | *EPHA8* |
| 2779 | *EPHB1* |
| 2780 | *EPHB2* |
| 2781 | *EPHB3* |
| 2782 | *EPHB4* |
| 2783 | *EPHB6* |
| 2784 | *EPHX1* |
| 2785 | *EPHX2* |
| 2786 | *EPM2AIP1* |
| 2787 | *EPN3* |
| 2788 | *EPO* |
| 2789 | *EPOR* |
| 2790 | *EPS15* |
| 2791 | *EPS15L1* |
| 2792 | *EPS15P1* |
| 2793 | *EPS8* |
| 2794 | *EPS8L2* |
| 2795 | *EPS8L3* |
| 2796 | *EPX* |
| 2797 | *ERAL1* |
| 2798 | *ERAP1* |
| 2799 | *ERAS* |
| 2800 | *ERBB2* |
| 2801 | *ERBB3* |
| 2802 | *ERBB4* |
| 2803 | *ERBIN* |
| 2804 | *ERC1* |
| 2805 | *ERCC1* |
| 2806 | *ERCC2* |
| 2807 | *ERCC3* |
| 2808 | *ERCC4* |
| 2809 | *ERCC5* |
| 2810 | *ERCC6* |
| 2811 | *ERCC6L* |
| 2812 | *ERCC8* |
| 2813 | *EREG* |
| 2814 | *ERF* |
| 2815 | *ERG* |
| 2816 | *ERG28* |
| 2817 | *ERGIC2* |
| 2818 | *ERGIC3* |
| 2819 | *ERH* |
| 2820 | *ERI2* |
| 2821 | *ERICH1* |
| 2822 | *ERLIN1* |
| 2823 | *ERLIN2* |
| 2824 | *ERN1* |
| 2825 | *ERN2* |
| 2826 | *ERO1A* |
| 2827 | *ERP29* |
| 2828 | *ERRFI1* |
| 2829 | *ERV3-1* |
| 2830 | *ERVW-1* |
| 2831 | *ESCO2* |
| 2832 | *ESD* |
| 2833 | *ESM1* |
| 2834 | *ESPL1* |
| 2835 | *ESR1* |
| 2836 | *ESR2* |
| 2837 | *ESRP1* |
| 2838 | *ESRP2* |
| 2839 | *ESRRA* |
| 2840 | *ESRRB* |
| 2841 | *ESRRG* |
| 2842 | *ESS2* |
| 2843 | *ETAA1* |
| 2844 | *ETF1* |
| 2845 | *ETF1P1* |
| 2846 | *ETFA* |
| 2847 | *ETFB* |
| 2848 | *ETFDH* |
| 2849 | *ETHE1* |
| 2850 | *ETS1* |
| 2851 | *ETS2* |
| 2852 | *ETV1* |
| 2853 | *ETV3* |
| 2854 | *ETV4* |
| 2855 | *ETV5* |
| 2856 | *ETV6* |
| 2857 | *ETV7* |
| 2858 | *EVC2* |
| 2859 | *EVL* |
| 2860 | *EWSAT1* |
| 2861 | *EWSR1* |
| 2862 | *EXD2* |
| 2863 | *EXO1* |
| 2864 | *EXOC2* |
| 2865 | *EXOC3* |
| 2866 | *EXOC3L1* |
| 2867 | *EXOC4* |
| 2868 | *EXOC7* |
| 2869 | *EXOC8* |
| 2870 | *EXOSC1* |
| 2871 | *EXOSC10* |
| 2872 | *EXOSC3* |
| 2873 | *EXOSC4* |
| 2874 | *EXOSC5* |
| 2875 | *EXOSC8* |
| 2876 | *EXOSC9* |
| 2877 | *EXPH5* |
| 2878 | *EXT1* |
| 2879 | *EXT2* |
| 2880 | *EXTL1* |
| 2881 | *EXTL3* |
| 2882 | *EYA1* |
| 2883 | *EYA2* |
| 2884 | *EYA4* |
| 2885 | *EYS* |
| 2886 | *EZH2* |
| 2887 | *EZR* |
| 2888 | *EZR-AS1* |
| 2889 | *F10* |
| 2890 | *F11* |
| 2891 | *F11R* |
| 2892 | *F12* |
| 2893 | *F13A1* |
| 2894 | *F2* |
| 2895 | *F2R* |
| 2896 | *F2RL1* |
| 2897 | *F2RL2* |
| 2898 | *F2RL3* |
| 2899 | *F3* |
| 2900 | *F5* |
| 2901 | *F7* |
| 2902 | *F8* |
| 2903 | *F9* |
| 2904 | *FA2H* |
| 2905 | *FAAH* |
| 2906 | *FAAP24* |
| 2907 | *FABP1* |
| 2908 | *FABP12* |
| 2909 | *FABP2* |
| 2910 | *FABP4* |
| 2911 | *FABP5* |
| 2912 | *FABP6* |
| 2913 | *FADD* |
| 2914 | *FADS1* |
| 2915 | *FADS2* |
| 2916 | *FADS3* |
| 2917 | *FAF1* |
| 2918 | *FAF2* |
| 2919 | *FAH* |
| 2920 | *FAIM2* |
| 2921 | *FALEC* |
| 2922 | *FAM107A* |
| 2923 | *FAM110B* |
| 2924 | *FAM110C* |
| 2925 | *FAM117B* |
| 2926 | *FAM118A* |
| 2927 | *FAM120A* |
| 2928 | *FAM120AOS* |
| 2929 | *FAM120C* |
| 2930 | *FAM126A* |
| 2931 | *FAM131A* |
| 2932 | *FAM156A* |
| 2933 | *FAM156B* |
| 2934 | *FAM161A* |
| 2935 | *FAM162A* |
| 2936 | *FAM171B* |
| 2937 | *FAM172A* |
| 2938 | *FAM177A1* |
| 2939 | *FAM182A* |
| 2940 | *FAM183BP* |
| 2941 | *FAM184A* |
| 2942 | *FAM185A* |
| 2943 | *FAM186A* |
| 2944 | *FAM193A* |
| 2945 | *FAM210A* |
| 2946 | *FAM217B* |
| 2947 | *FAM241B* |
| 2948 | *FAM27E5* |
| 2949 | *FAM30A* |
| 2950 | *FAM32A* |
| 2951 | *FAM3A* |
| 2952 | *FAM3B* |
| 2953 | *FAM3C* |
| 2954 | *FAM3D* |
| 2955 | *FAM3D-AS1* |
| 2956 | *FAM72A* |
| 2957 | *FAM83B* |
| 2958 | *FAM83D* |
| 2959 | *FAM83E* |
| 2960 | *FAM83F* |
| 2961 | *FAM83G* |
| 2962 | *FAM83H* |
| 2963 | *FAM83H-AS1* |
| 2964 | *FAM86JP* |
| 2965 | *FAM98A* |
| 2966 | *FAM98B* |
| 2967 | *FAN1* |
| 2968 | *FANCA* |
| 2969 | *FANCC* |
| 2970 | *FANCD2* |
| 2971 | *FANCE* |
| 2972 | *FANCF* |
| 2973 | *FANCG* |
| 2974 | *FANCI* |
| 2975 | *FANCL* |
| 2976 | *FANCM* |
| 2977 | *FAP* |
| 2978 | *FAR1* |
| 2979 | *FARP1* |
| 2980 | *FARP2* |
| 2981 | *FARSA* |
| 2982 | *FARSB* |
| 2983 | *FAS* |
| 2984 | *FASLG* |
| 2985 | *FASN* |
| 2986 | *FASTK* |
| 2987 | *FASTKD2* |
| 2988 | *FASTKD3* |
| 2989 | *FAT1* |
| 2990 | *FAT2* |
| 2991 | *FAT3* |
| 2992 | *FAT4* |
| 2993 | *FATE1* |
| 2994 | *FAU* |
| 2995 | *FBH1* |
| 2996 | *FBL* |
| 2997 | *FBLIM1* |
| 2998 | *FBLN1* |
| 2999 | *FBLN2* |
| 3000 | *FBLN5* |
| 3001 | *FBN1* |
| 3002 | *FBN2* |
| 3003 | *FBP1* |
| 3004 | *FBP2* |
| 3005 | *FBXL13* |
| 3006 | *FBXL19-AS1* |
| 3007 | *FBXL2* |
| 3008 | *FBXL20* |
| 3009 | *FBXL3* |
| 3010 | *FBXL6* |
| 3011 | *FBXO11* |
| 3012 | *FBXO2* |
| 3013 | *FBXO21* |
| 3014 | *FBXO22* |
| 3015 | *FBXO24* |
| 3016 | *FBXO30* |
| 3017 | *FBXO32* |
| 3018 | *FBXO39* |
| 3019 | *FBXO42* |
| 3020 | *FBXO45* |
| 3021 | *FBXO46* |
| 3022 | *FBXO6* |
| 3023 | *FBXO7* |
| 3024 | *FBXO8* |
| 3025 | *FBXO9* |
| 3026 | *FBXW11* |
| 3027 | *FBXW7* |
| 3028 | *FCAR* |
| 3029 | *FCF1P10* |
| 3030 | *FCGBP* |
| 3031 | *FCGR1A* |
| 3032 | *FCGR2A* |
| 3033 | *FCGR2B* |
| 3034 | *FCGR2C* |
| 3035 | *FCGR3A* |
| 3036 | *FCGR3B* |
| 3037 | *FCGRT* |
| 3038 | *FCHO1* |
| 3039 | *FCN1* |
| 3040 | *FCN2* |
| 3041 | *FCRL3* |
| 3042 | *FCRL5* |
| 3043 | *FDFT1* |
| 3044 | *FDPS* |
| 3045 | *FDXR* |
| 3046 | *FECH* |
| 3047 | *FEM1B* |
| 3048 | *FEM1C* |
| 3049 | *FEN1* |
| 3050 | *FENDRR* |
| 3051 | *FER* |
| 3052 | *FER1L4* |
| 3053 | *FERD3L* |
| 3054 | *FERMT1* |
| 3055 | *FERMT2* |
| 3056 | *FERMT3* |
| 3057 | *FES* |
| 3058 | *FEV* |
| 3059 | *FEZF1* |
| 3060 | *FEZF1-AS1* |
| 3061 | *FEZF2* |
| 3062 | *FFAR1* |
| 3063 | *FFAR2* |
| 3064 | *FFAR4* |
| 3065 | *FGA* |
| 3066 | *FGB* |
| 3067 | *FGD1* |
| 3068 | *FGD2* |
| 3069 | *FGD3* |
| 3070 | *FGD4* |
| 3071 | *FGF1* |
| 3072 | *FGF10* |
| 3073 | *FGF12* |
| 3074 | *FGF13* |
| 3075 | *FGF14* |
| 3076 | *FGF18* |
| 3077 | *FGF19* |
| 3078 | *FGF2* |
| 3079 | *FGF20* |
| 3080 | *FGF21* |
| 3081 | *FGF23* |
| 3082 | *FGF3* |
| 3083 | *FGF4* |
| 3084 | *FGF6* |
| 3085 | *FGF7* |
| 3086 | *FGF7P5* |
| 3087 | *FGF8* |
| 3088 | *FGF9* |
| 3089 | *FGFBP1* |
| 3090 | *FGFR1* |
| 3091 | *FGFR1OP* |
| 3092 | *FGFR2* |
| 3093 | *FGFR3* |
| 3094 | *FGFR3P3* |
| 3095 | *FGFR4* |
| 3096 | *FGG* |
| 3097 | *FGL1* |
| 3098 | *FGL2* |
| 3099 | *FGR* |
| 3100 | *FH* |
| 3101 | *FHIP2B* |
| 3102 | *FHIT* |
| 3103 | *FHL1* |
| 3104 | *FHL2* |
| 3105 | *FHL3* |
| 3106 | *FIBCD1* |
| 3107 | *FIBP* |
| 3108 | *FILIP1L* |
| 3109 | *FIP1L1* |
| 3110 | *FIRRE* |
| 3111 | *FITM1* |
| 3112 | *FJX1* |
| 3113 | *FKBP10* |
| 3114 | *FKBP15* |
| 3115 | *FKBP1A* |
| 3116 | *FKBP3* |
| 3117 | *FKBP4* |
| 3118 | *FKBP5* |
| 3119 | *FKBP8* |
| 3120 | *FKBPL* |
| 3121 | *FKTN* |
| 3122 | *FLAD1* |
| 3123 | *FLCN* |
| 3124 | *FLG* |
| 3125 | *FLG2* |
| 3126 | *FLI1* |
| 3127 | *FLII* |
| 3128 | *FLNA* |
| 3129 | *FLNB* |
| 3130 | *FLNC* |
| 3131 | *FLOT1* |
| 3132 | *FLOT2* |
| 3133 | *FLRT3* |
| 3134 | *FLT1* |
| 3135 | *FLT1P1* |
| 3136 | *FLT3* |
| 3137 | *FLT3LG* |
| 3138 | *FLT4* |
| 3139 | *FLVCR2* |
| 3140 | *FLYWCH1* |
| 3141 | *FLYWCH2* |
| 3142 | *FMN1* |
| 3143 | *FMN2* |
| 3144 | *FMNL2* |
| 3145 | *FMNL3* |
| 3146 | *FMO3* |
| 3147 | *FMO5* |
| 3148 | *FMR1* |
| 3149 | *FN1* |
| 3150 | *FN3K* |
| 3151 | *FNDC1* |
| 3152 | *FNDC11* |
| 3153 | *FNDC3B* |
| 3154 | *FNDC5* |
| 3155 | *FNIP1* |
| 3156 | *FNIP2* |
| 3157 | *FNTA* |
| 3158 | *FNTB* |
| 3159 | *FOCAD* |
| 3160 | *FOLH1* |
| 3161 | *FOLR1* |
| 3162 | *FOLR2* |
| 3163 | *FOS* |
| 3164 | *FOSB* |
| 3165 | *FOSL1* |
| 3166 | *FOSL2* |
| 3167 | *FOXA1* |
| 3168 | *FOXA2* |
| 3169 | *FOXC1* |
| 3170 | *FOXC2* |
| 3171 | *FOXD1* |
| 3172 | *FOXD2-AS1* |
| 3173 | *FOXD3* |
| 3174 | *FOXD3-AS1* |
| 3175 | *FOXD4* |
| 3176 | *FOXE1* |
| 3177 | *FOXF1* |
| 3178 | *FOXF2* |
| 3179 | *FOXH1* |
| 3180 | *FOXJ1* |
| 3181 | *FOXJ2* |
| 3182 | *FOXJ3* |
| 3183 | *FOXK1* |
| 3184 | *FOXK2* |
| 3185 | *FOXL1* |
| 3186 | *FOXL2* |
| 3187 | *FOXM1* |
| 3188 | *FOXN2* |
| 3189 | *FOXN3* |
| 3190 | *FOXO1* |
| 3191 | *FOXO3* |
| 3192 | *FOXO4* |
| 3193 | *FOXO6* |
| 3194 | *FOXP1* |
| 3195 | *FOXP2* |
| 3196 | *FOXP3* |
| 3197 | *FOXP4* |
| 3198 | *FOXP4-AS1* |
| 3199 | *FOXQ1* |
| 3200 | *FOXR1* |
| 3201 | *FOXR2* |
| 3202 | *FOXS1* |
| 3203 | *FPGS* |
| 3204 | *FPR1* |
| 3205 | *FPR2* |
| 3206 | *FRA3B* |
| 3207 | *FRAT1* |
| 3208 | *FRAT2* |
| 3209 | *FRAXA* |
| 3210 | *FRMD3* |
| 3211 | *FRMD5* |
| 3212 | *FRMD6* |
| 3213 | *FRMPD1* |
| 3214 | *FRMPD2* |
| 3215 | *FRS2* |
| 3216 | *FRTS1* |
| 3217 | *FRY* |
| 3218 | *FRZB* |
| 3219 | *FSBP* |
| 3220 | *FSCB* |
| 3221 | *FSCN1* |
| 3222 | *FSCN2* |
| 3223 | *FSD1* |
| 3224 | *FSD1L* |
| 3225 | *FSD2* |
| 3226 | *FSHR* |
| 3227 | *FSIP1* |
| 3228 | *FSTL1* |
| 3229 | *FSTL3* |
| 3230 | *FSTL4* |
| 3231 | *FSTL5* |
| 3232 | *FTH1* |
| 3233 | *FTH1P1* |
| 3234 | *FTH1P6* |
| 3235 | *FTL* |
| 3236 | *FTLP14* |
| 3237 | *FTLP15* |
| 3238 | *FTO* |
| 3239 | *FTSJ1* |
| 3240 | *FTSJ3* |
| 3241 | *FTX* |
| 3242 | *FUBP1* |
| 3243 | *FUBP3* |
| 3244 | *FUCA1* |
| 3245 | *FURIN* |
| 3246 | *FUS* |
| 3247 | *FUT1* |
| 3248 | *FUT2* |
| 3249 | *FUT3* |
| 3250 | *FUT4* |
| 3251 | *FUT5* |
| 3252 | *FUT6* |
| 3253 | *FUT7* |
| 3254 | *FUT8* |
| 3255 | *FUT9* |
| 3256 | *FUZ* |
| 3257 | *FXR1* |
| 3258 | *FXR2* |
| 3259 | *FXYD1* |
| 3260 | *FXYD3* |
| 3261 | *FXYD5* |
| 3262 | *FXYD6* |
| 3263 | *FYN* |
| 3264 | *FYTTD1* |
| 3265 | *FZD1* |
| 3266 | *FZD10* |
| 3267 | *FZD2* |
| 3268 | *FZD3* |
| 3269 | *FZD4* |
| 3270 | *FZD5* |
| 3271 | *FZD6* |
| 3272 | *FZD7* |
| 3273 | *FZD8* |
| 3274 | *FZD9* |
| 3275 | *FZR1* |
| 3276 | *G3BP1* |
| 3277 | *G3BP2* |
| 3278 | *G6PC1* |
| 3279 | *G6PD* |
| 3280 | *GAA* |
| 3281 | *GAB1* |
| 3282 | *GAB2* |
| 3283 | *GAB3* |
| 3284 | *GABARAP* |
| 3285 | *GABARAPL1* |
| 3286 | *GABARAPL2* |
| 3287 | *GABARAPL3* |
| 3288 | *GABBR1* |
| 3289 | *GABPA* |
| 3290 | *GABPB1* |
| 3291 | *GABRA1* |
| 3292 | *GABRA4* |
| 3293 | *GABRA6* |
| 3294 | *GABRP* |
| 3295 | *GABRR3* |
| 3296 | *GACAT3* |
| 3297 | *GAD2* |
| 3298 | *GADD45A* |
| 3299 | *GADD45B* |
| 3300 | *GADD45G* |
| 3301 | *GADL1* |
| 3302 | *GAEC1* |
| 3303 | *GAGE13* |
| 3304 | *GAGE7* |
| 3305 | *GAK* |
| 3306 | *GAL* |
| 3307 | *GAL3ST2* |
| 3308 | *GALK1* |
| 3309 | *GALM* |
| 3310 | *GALNS* |
| 3311 | *GALNT1* |
| 3312 | *GALNT11* |
| 3313 | *GALNT12* |
| 3314 | *GALNT13* |
| 3315 | *GALNT14* |
| 3316 | *GALNT15* |
| 3317 | *GALNT16* |
| 3318 | *GALNT3* |
| 3319 | *GALNT4* |
| 3320 | *GALNT5* |
| 3321 | *GALNT6* |
| 3322 | *GALNT7* |
| 3323 | *GALNT8* |
| 3324 | *GALP* |
| 3325 | *GALR1* |
| 3326 | *GALR2* |
| 3327 | *GALR3* |
| 3328 | *GALT* |
| 3329 | *GAMT* |
| 3330 | *GAMTP1* |
| 3331 | *GAP43* |
| 3332 | *GAPDH* |
| 3333 | *GAPDHS* |
| 3334 | *GAPLINC* |
| 3335 | *GAPVD1* |
| 3336 | *GAR1* |
| 3337 | *GAREM1* |
| 3338 | *GARRE1* |
| 3339 | *GART* |
| 3340 | *GAS1* |
| 3341 | *GAS2* |
| 3342 | *GAS2L3* |
| 3343 | *GAS5* |
| 3344 | *GAS6* |
| 3345 | *GAS7* |
| 3346 | *GAS8* |
| 3347 | *GAS8-AS1* |
| 3348 | *GAST* |
| 3349 | *GATA1* |
| 3350 | *GATA2* |
| 3351 | *GATA3* |
| 3352 | *GATA4* |
| 3353 | *GATA5* |
| 3354 | *GATA6* |
| 3355 | *GATAD1* |
| 3356 | *GATAD2A* |
| 3357 | *GATAD2B* |
| 3358 | *GATC* |
| 3359 | *GATD1* |
| 3360 | *GATD3* |
| 3361 | *GBA1* |
| 3362 | *GBAP1* |
| 3363 | *GBE1* |
| 3364 | *GBF1* |
| 3365 | *GBP1* |
| 3366 | *GBP2* |
| 3367 | *GC* |
| 3368 | *GCA* |
| 3369 | *GCAT* |
| 3370 | *GCC1* |
| 3371 | *GCC2* |
| 3372 | *GCDH* |
| 3373 | *GCFC2* |
| 3374 | *GCG* |
| 3375 | *GCH1* |
| 3376 | *GCK* |
| 3377 | *GCLC* |
| 3378 | *GCLM* |
| 3379 | *GCM1* |
| 3380 | *GCM2* |
| 3381 | *GCN1* |
| 3382 | *GCNT1* |
| 3383 | *GCNT2* |
| 3384 | *GCNT3* |
| 3385 | *GCNT7* |
| 3386 | *GDAP1L1* |
| 3387 | *GDE1* |
| 3388 | *GDF1* |
| 3389 | *GDF11* |
| 3390 | *GDF15* |
| 3391 | *GDF2* |
| 3392 | *GDF5* |
| 3393 | *GDI1* |
| 3394 | *GDI2* |
| 3395 | *GDNF* |
| 3396 | *GDPD2* |
| 3397 | *GDPD5* |
| 3398 | *GEM* |
| 3399 | *GEMIN4* |
| 3400 | *GEMIN5* |
| 3401 | *GEN1* |
| 3402 | *GET4* |
| 3403 | *GFAP* |
| 3404 | *GFER* |
| 3405 | *GFI1* |
| 3406 | *GFI1B* |
| 3407 | *GFPT1* |
| 3408 | *GFRA1* |
| 3409 | *GFRA2* |
| 3410 | *GFRA3* |
| 3411 | *GFRAL* |
| 3412 | *GGA1* |
| 3413 | *GGA2* |
| 3414 | *GGA3* |
| 3415 | *GGCT* |
| 3416 | *GGCTP3* |
| 3417 | *GGH* |
| 3418 | *GGT1* |
| 3419 | *GGT2* |
| 3420 | *GGTLC3* |
| 3421 | *GGTLC4P* |
| 3422 | *GGTLC5P* |
| 3423 | *GH1* |
| 3424 | *GHET1* |
| 3425 | *GHR* |
| 3426 | *GHRH* |
| 3427 | *GHRHR* |
| 3428 | *GHRL* |
| 3429 | *GHSR* |
| 3430 | *GID4* |
| 3431 | *GID8* |
| 3432 | *GIGYF2* |
| 3433 | *GIHCG* |
| 3434 | *GIMAP1* |
| 3435 | *GINS1* |
| 3436 | *GINS2* |
| 3437 | *GINS3* |
| 3438 | *GINS4* |
| 3439 | *GIP* |
| 3440 | *GIPC1* |
| 3441 | *GIPC2* |
| 3442 | *GIT1* |
| 3443 | *GIT2* |
| 3444 | *GJA1* |
| 3445 | *GJA10* |
| 3446 | *GJA3* |
| 3447 | *GJB1* |
| 3448 | *GJB2* |
| 3449 | *GJC1* |
| 3450 | *GJD2* |
| 3451 | *GJD3* |
| 3452 | *GJD4* |
| 3453 | *GKN1* |
| 3454 | *GLB1* |
| 3455 | *GLB1L2* |
| 3456 | *GLCCI1* |
| 3457 | *GLDC* |
| 3458 | *GLE1* |
| 3459 | *GLG1* |
| 3460 | *GLI1* |
| 3461 | *GLI2* |
| 3462 | *GLI3* |
| 3463 | *GLIS2* |
| 3464 | *GLIS3* |
| 3465 | *GLO1* |
| 3466 | *GLOD4* |
| 3467 | *GLP1R* |
| 3468 | *GLRX3* |
| 3469 | *GLS* |
| 3470 | *GLS2* |
| 3471 | *GLTP* |
| 3472 | *GLUD1* |
| 3473 | *GLUD2* |
| 3474 | *GLUL* |
| 3475 | *GLYR1* |
| 3476 | *GM2A* |
| 3477 | *GMCL1* |
| 3478 | *GMCL2* |
| 3479 | *GMDS* |
| 3480 | *GMFG* |
| 3481 | *GML* |
| 3482 | *GMNN* |
| 3483 | *GMPPA* |
| 3484 | *GMPR2* |
| 3485 | *GNA12* |
| 3486 | *GNA13* |
| 3487 | *GNAI1* |
| 3488 | *GNAI2* |
| 3489 | *GNAI3* |
| 3490 | *GNAQ* |
| 3491 | *GNAS* |
| 3492 | *GNAT1* |
| 3493 | *GNB1* |
| 3494 | *GNB1L* |
| 3495 | *GNB2* |
| 3496 | *GNB3* |
| 3497 | *GNB4* |
| 3498 | *GNB5* |
| 3499 | *GNE* |
| 3500 | *GNG10* |
| 3501 | *GNG11* |
| 3502 | *GNG12* |
| 3503 | *GNG13* |
| 3504 | *GNG2* |
| 3505 | *GNG3* |
| 3506 | *GNG4* |
| 3507 | *GNG5* |
| 3508 | *GNG5P1* |
| 3509 | *GNG7* |
| 3510 | *GNG8* |
| 3511 | *GNGT1* |
| 3512 | *GNGT2* |
| 3513 | *GNL2* |
| 3514 | *GNL3* |
| 3515 | *GNLY* |
| 3516 | *GNPDA2* |
| 3517 | *GNPTAB* |
| 3518 | *GNRH1* |
| 3519 | *GOLGA2* |
| 3520 | *GOLGA2P10* |
| 3521 | *GOLGA4* |
| 3522 | *GOLGA7* |
| 3523 | *GOLGA8B* |
| 3524 | *GOLGB1* |
| 3525 | *GOLIM4* |
| 3526 | *GOLM1* |
| 3527 | *GOLM2* |
| 3528 | *GOLPH3* |
| 3529 | *GOLT1B* |
| 3530 | *GOPC* |
| 3531 | *GORASP1* |
| 3532 | *GOSR1* |
| 3533 | *GOT1* |
| 3534 | *GOT2* |
| 3535 | *GP1BA* |
| 3536 | *GP2* |
| 3537 | *GP6* |
| 3538 | *GPA33* |
| 3539 | *GPAA1* |
| 3540 | *GPAT4* |
| 3541 | *GPATCH1* |
| 3542 | *GPATCH4* |
| 3543 | *GPBAR1* |
| 3544 | *GPBP1L1* |
| 3545 | *GPC1* |
| 3546 | *GPC2* |
| 3547 | *GPC3* |
| 3548 | *GPC4* |
| 3549 | *GPC5* |
| 3550 | *GPC6* |
| 3551 | *GPD1* |
| 3552 | *GPER1* |
| 3553 | *GPI* |
| 3554 | *GPKOW* |
| 3555 | *GPLD1* |
| 3556 | *GPM6B* |
| 3557 | *GPNMB* |
| 3558 | *GPR101* |
| 3559 | *GPR119* |
| 3560 | *GPR137* |
| 3561 | *GPR143* |
| 3562 | *GPR15* |
| 3563 | *GPR151* |
| 3564 | *GPR15LG* |
| 3565 | *GPR162* |
| 3566 | *GPR166P* |
| 3567 | *GPR180* |
| 3568 | *GPR31* |
| 3569 | *GPR34* |
| 3570 | *GPR35* |
| 3571 | *GPR39* |
| 3572 | *GPR4* |
| 3573 | *GPR42* |
| 3574 | *GPR45* |
| 3575 | *GPR55* |
| 3576 | *GPR68* |
| 3577 | *GPR87* |
| 3578 | *GPR89A* |
| 3579 | *GPRASP1* |
| 3580 | *GPRASP3* |
| 3581 | *GPRC5A* |
| 3582 | *GPRC5C* |
| 3583 | *GPRC5D* |
| 3584 | *GPRC6A* |
| 3585 | *GPS1* |
| 3586 | *GPS2* |
| 3587 | *GPSM3* |
| 3588 | *GPT* |
| 3589 | *GPT2* |
| 3590 | *GPX1* |
| 3591 | *GPX2* |
| 3592 | *GPX3* |
| 3593 | *GPX4* |
| 3594 | *GPX8* |
| 3595 | *GRAMD1B* |
| 3596 | *GRAMD4* |
| 3597 | *GRAP2* |
| 3598 | *GRASP* |
| 3599 | *GRB10* |
| 3600 | *GRB14* |
| 3601 | *GRB2* |
| 3602 | *GRB7* |
| 3603 | *GREB1* |
| 3604 | *GREB1L* |
| 3605 | *GREM1* |
| 3606 | *GREM2* |
| 3607 | *GRHL1* |
| 3608 | *GRHL2* |
| 3609 | *GRHL3* |
| 3610 | *GRHPR* |
| 3611 | *GRIA1* |
| 3612 | *GRIA4* |
| 3613 | *GRID1* |
| 3614 | *GRID2* |
| 3615 | *GRIK2* |
| 3616 | *GRIK3* |
| 3617 | *GRIN1* |
| 3618 | *GRIN2A* |
| 3619 | *GRIN2B* |
| 3620 | *GRIN2D* |
| 3621 | *GRINA* |
| 3622 | *GRK1* |
| 3623 | *GRK2* |
| 3624 | *GRK3* |
| 3625 | *GRK4* |
| 3626 | *GRK5* |
| 3627 | *GRK6* |
| 3628 | *GRK7* |
| 3629 | *GRM1* |
| 3630 | *GRM2* |
| 3631 | *GRM3* |
| 3632 | *GRM4* |
| 3633 | *GRM5* |
| 3634 | *GRM6* |
| 3635 | *GRM7* |
| 3636 | *GRN* |
| 3637 | *GRP* |
| 3638 | *GRPEL1* |
| 3639 | *GRPR* |
| 3640 | *GRWD1* |
| 3641 | *GSDMA* |
| 3642 | *GSDMB* |
| 3643 | *GSDMC* |
| 3644 | *GSDMD* |
| 3645 | *GSDME* |
| 3646 | *GSE1* |
| 3647 | *GSEC* |
| 3648 | *GSK3A* |
| 3649 | *GSK3B* |
| 3650 | *GSN* |
| 3651 | *GSPT1* |
| 3652 | *GSPT2* |
| 3653 | *GSR* |
| 3654 | *GSS* |
| 3655 | *GSTA1* |
| 3656 | *GSTA2* |
| 3657 | *GSTA3* |
| 3658 | *GSTA4* |
| 3659 | *GSTK1* |
| 3660 | *GSTM1* |
| 3661 | *GSTM2* |
| 3662 | *GSTM3* |
| 3663 | *GSTO1* |
| 3664 | *GSTO2* |
| 3665 | *GSTP1* |
| 3666 | *GSTT1* |
| 3667 | *GSTT2* |
| 3668 | *GTF2A1* |
| 3669 | *GTF2B* |
| 3670 | *GTF2E1* |
| 3671 | *GTF2E2* |
| 3672 | *GTF2F1* |
| 3673 | *GTF2H1* |
| 3674 | *GTF2H2* |
| 3675 | *GTF2H5* |
| 3676 | *GTF2I* |
| 3677 | *GTF2IRD1* |
| 3678 | *GTF2IRD2B* |
| 3679 | *GTF3A* |
| 3680 | *GTF3C1* |
| 3681 | *GTF3C4* |
| 3682 | *GTF3C5* |
| 3683 | *GTPBP3* |
| 3684 | *GTPBP4* |
| 3685 | *GTSF1L* |
| 3686 | *GUCA2A* |
| 3687 | *GUCA2B* |
| 3688 | *GUCD1* |
| 3689 | *GUCY1A2* |
| 3690 | *GUCY2C* |
| 3691 | *GUCY2F* |
| 3692 | *GUSB* |
| 3693 | *GXYLT1* |
| 3694 | *GYPA* |
| 3695 | *GYS1* |
| 3696 | *GZMA* |
| 3697 | *GZMB* |
| 3698 | *H1-0* |
| 3699 | *H1-1* |
| 3700 | *H1-10* |
| 3701 | *H1-2* |
| 3702 | *H1-3* |
| 3703 | *H1-4* |
| 3704 | *H1-5* |
| 3705 | *H1-6* |
| 3706 | *H19* |
| 3707 | *H19-ICR* |
| 3708 | *H2AC12* |
| 3709 | *H2AC13* |
| 3710 | *H2AC14* |
| 3711 | *H2AC18* |
| 3712 | *H2AC19* |
| 3713 | *H2AC20* |
| 3714 | *H2AC21* |
| 3715 | *H2AC25* |
| 3716 | *H2AC4* |
| 3717 | *H2AC6* |
| 3718 | *H2AC7* |
| 3719 | *H2AC8* |
| 3720 | *H2AC9P* |
| 3721 | *H2AJ* |
| 3722 | *H2AX* |
| 3723 | *H2AZ1* |
| 3724 | *H2AZ2* |
| 3725 | *H2BC1* |
| 3726 | *H2BC11* |
| 3727 | *H2BC12* |
| 3728 | *H2BC12L* |
| 3729 | *H2BC13* |
| 3730 | *H2BC14* |
| 3731 | *H2BC15* |
| 3732 | *H2BC17* |
| 3733 | *H2BC18* |
| 3734 | *H2BC21* |
| 3735 | *H2BC3* |
| 3736 | *H2BC4* |
| 3737 | *H2BC5* |
| 3738 | *H2BC9* |
| 3739 | *H3-3A* |
| 3740 | *H3-3B* |
| 3741 | *H3-4* |
| 3742 | *H3-5* |
| 3743 | *H3-7* |
| 3744 | *H3C1* |
| 3745 | *H3C10* |
| 3746 | *H3C11* |
| 3747 | *H3C12* |
| 3748 | *H3C14* |
| 3749 | *H3C15* |
| 3750 | *H3C2* |
| 3751 | *H3C3* |
| 3752 | *H3C4* |
| 3753 | *H3C6* |
| 3754 | *H3C7* |
| 3755 | *H3C8* |
| 3756 | *H3P10* |
| 3757 | *H3P13* |
| 3758 | *H3P16* |
| 3759 | *H3P23* |
| 3760 | *H3P28* |
| 3761 | *H3P40* |
| 3762 | *H3P47* |
| 3763 | *H3P8* |
| 3764 | *H3P9* |
| 3765 | *H4-16* |
| 3766 | *H4C1* |
| 3767 | *H4C11* |
| 3768 | *H4C12* |
| 3769 | *H4C13* |
| 3770 | *H4C14* |
| 3771 | *H4C15* |
| 3772 | *H4C16* |
| 3773 | *H4C2* |
| 3774 | *H4C3* |
| 3775 | *H4C4* |
| 3776 | *H4C5* |
| 3777 | *H4C6* |
| 3778 | *H4C8* |
| 3779 | *H4C9* |
| 3780 | *H6PD* |
| 3781 | *HAAO* |
| 3782 | *HABP2* |
| 3783 | *HABP4* |
| 3784 | *HACD3* |
| 3785 | *HACD4* |
| 3786 | *HACE1* |
| 3787 | *HACL1* |
| 3788 | *HADHA* |
| 3789 | *HADHB* |
| 3790 | *HAGLR* |
| 3791 | *HAMP* |
| 3792 | *HAND2* |
| 3793 | *HAND2-AS1* |
| 3794 | *HAO1* |
| 3795 | *HAPLN1* |
| 3796 | *HAPSTR1* |
| 3797 | *HAS1* |
| 3798 | *HAS2* |
| 3799 | *HAS3* |
| 3800 | *HASPIN* |
| 3801 | *HAT1* |
| 3802 | *HAUS8* |
| 3803 | *HAVCR1* |
| 3804 | *HAVCR2* |
| 3805 | *HAX1* |
| 3806 | *HBA1* |
| 3807 | *HBA2* |
| 3808 | *HBB* |
| 3809 | *HBD* |
| 3810 | *HBEGF* |
| 3811 | *HBP1* |
| 3812 | *HBS1L* |
| 3813 | *HCA1* |
| 3814 | *HCAR2* |
| 3815 | *HCC* |
| 3816 | *HCFC1* |
| 3817 | *HCFC1R1* |
| 3818 | *HCG22* |
| 3819 | *HCG9P5* |
| 3820 | *HCK* |
| 3821 | *HCLS1* |
| 3822 | *HCN1* |
| 3823 | *HCRTR2* |
| 3824 | *HDAC1* |
| 3825 | *HDAC10* |
| 3826 | *HDAC2* |
| 3827 | *HDAC3* |
| 3828 | *HDAC4* |
| 3829 | *HDAC5* |
| 3830 | *HDAC6* |
| 3831 | *HDAC7* |
| 3832 | *HDAC8* |
| 3833 | *HDAC9* |
| 3834 | *HDC* |
| 3835 | *HDDC3* |
| 3836 | *HDGF* |
| 3837 | *HDLBP* |
| 3838 | *HEATR1* |
| 3839 | *HEATR6* |
| 3840 | *HEATR9* |
| 3841 | *HECA* |
| 3842 | *HECTD1* |
| 3843 | *HECTD2* |
| 3844 | *HECTD4* |
| 3845 | *HECW1* |
| 3846 | *HEIH* |
| 3847 | *HELLS* |
| 3848 | *HELQ* |
| 3849 | *HELZ* |
| 3850 | *HELZ2* |
| 3851 | *HEMGN* |
| 3852 | *HEPACAM* |
| 3853 | *HEPACAM2* |
| 3854 | *HEPH* |
| 3855 | *HERC2* |
| 3856 | *HERC2P2* |
| 3857 | *HERC3* |
| 3858 | *HERC4* |
| 3859 | *HERC5* |
| 3860 | *HES1* |
| 3861 | *HES2* |
| 3862 | *HES3* |
| 3863 | *HES4* |
| 3864 | *HES5* |
| 3865 | *HES6* |
| 3866 | *HES7* |
| 3867 | *HEXA* |
| 3868 | *HEXB* |
| 3869 | *HEXD* |
| 3870 | *HEXIM1* |
| 3871 | *HEY1* |
| 3872 | *HEY2* |
| 3873 | *HEYL* |
| 3874 | *HFE* |
| 3875 | *HFM1* |
| 3876 | *HGD* |
| 3877 | *HGF* |
| 3878 | *HGFAC* |
| 3879 | *HGS* |
| 3880 | *HGSNAT* |
| 3881 | *HHAT* |
| 3882 | *HHATL* |
| 3883 | *HHEX* |
| 3884 | *HHIP* |
| 3885 | *HHIPL2* |
| 3886 | *HHLA2* |
| 3887 | *HIBCH* |
| 3888 | *HIC1* |
| 3889 | *HIC2* |
| 3890 | *HIF1A* |
| 3891 | *HIF1A-AS1* |
| 3892 | *HIF1A-AS2* |
| 3893 | *HIF1AN* |
| 3894 | *HIGD1A* |
| 3895 | *HIGD1C* |
| 3896 | *HILPDA* |
| 3897 | *HINFP* |
| 3898 | *HINT1* |
| 3899 | *HINT2* |
| 3900 | *HIP1* |
| 3901 | *HIPK1* |
| 3902 | *HIPK2* |
| 3903 | *HIPK3* |
| 3904 | *HIRA* |
| 3905 | *HIVEP1* |
| 3906 | *HIVEP3* |
| 3907 | *HJURP* |
| 3908 | *HK1* |
| 3909 | *HK2* |
| 3910 | *HK3* |
| 3911 | *HKDC1* |
| 3912 | *HLA-A* |
| 3913 | *HLA-B* |
| 3914 | *HLA-C* |
| 3915 | *HLA-DMA* |
| 3916 | *HLA-DMB* |
| 3917 | *HLA-DOA* |
| 3918 | *HLA-DOB* |
| 3919 | *HLA-DPA1* |
| 3920 | *HLA-DPB1* |
| 3921 | *HLA-DQA1* |
| 3922 | *HLA-DQA2* |
| 3923 | *HLA-DQB1* |
| 3924 | *HLA-DQB2* |
| 3925 | *HLA-DRA* |
| 3926 | *HLA-DRB1* |
| 3927 | *HLA-DRB4* |
| 3928 | *HLA-DRB5* |
| 3929 | *HLA-DRB6* |
| 3930 | *HLA-DRB9* |
| 3931 | *HLA-E* |
| 3932 | *HLA-F* |
| 3933 | *HLA-G* |
| 3934 | *HLA-H* |
| 3935 | *HLA-J* |
| 3936 | *HLA-K* |
| 3937 | *HLA-S* |
| 3938 | *HLA-U* |
| 3939 | *HLA-V* |
| 3940 | *HLA-W* |
| 3941 | *HLCS* |
| 3942 | *HLTF* |
| 3943 | *HLX* |
| 3944 | *HM13* |
| 3945 | *HMBS* |
| 3946 | *HMCN1* |
| 3947 | *HMGA1* |
| 3948 | *HMGA2* |
| 3949 | *HMGB1* |
| 3950 | *HMGB1P1* |
| 3951 | *HMGB1P14* |
| 3952 | *HMGB2* |
| 3953 | *HMGB3* |
| 3954 | *HMGCR* |
| 3955 | *HMGCS2* |
| 3956 | *HMGN1* |
| 3957 | *HMGN2* |
| 3958 | *HMGN2P24* |
| 3959 | *HMGN3* |
| 3960 | *HMGN5* |
| 3961 | *HMGXB3* |
| 3962 | *HMMR* |
| 3963 | *HMOX1* |
| 3964 | *HMOX2* |
| 3965 | *HNF1A* |
| 3966 | *HNF1A-AS1* |
| 3967 | *HNF1B* |
| 3968 | *HNF4A* |
| 3969 | *HNF4G* |
| 3970 | *HNMT* |
| 3971 | *HNP1* |
| 3972 | *HNRNPA0* |
| 3973 | *HNRNPA1* |
| 3974 | *HNRNPA1L2* |
| 3975 | *HNRNPA1P10* |
| 3976 | *HNRNPA2B1* |
| 3977 | *HNRNPA3* |
| 3978 | *HNRNPAB* |
| 3979 | *HNRNPC* |
| 3980 | *HNRNPCL1* |
| 3981 | *HNRNPCL2* |
| 3982 | *HNRNPD* |
| 3983 | *HNRNPDL* |
| 3984 | *HNRNPF* |
| 3985 | *HNRNPH1* |
| 3986 | *HNRNPH2* |
| 3987 | *HNRNPH3* |
| 3988 | *HNRNPK* |
| 3989 | *HNRNPKP2* |
| 3990 | *HNRNPL* |
| 3991 | *HNRNPLL* |
| 3992 | *HNRNPM* |
| 3993 | *HNRNPR* |
| 3994 | *HNRNPU* |
| 3995 | *HNRNPUL1* |
| 3996 | *HNRNPUL2* |
| 3997 | *HOMER2* |
| 3998 | *HOMER3* |
| 3999 | *HOOK1* |
| 4000 | *HOOK2* |
| 4001 | *HOOK3* |
| 4002 | *HOPX* |
| 4003 | *HOTAIR* |
| 4004 | *HOTAIRM1* |
| 4005 | *HOTTIP* |
| 4006 | *HOXA@* |
| 4007 | *HOXA1* |
| 4008 | *HOXA10* |
| 4009 | *HOXA11-AS* |
| 4010 | *HOXA13* |
| 4011 | *HOXA2* |
| 4012 | *HOXA3* |
| 4013 | *HOXA4* |
| 4014 | *HOXA5* |
| 4015 | *HOXA6* |
| 4016 | *HOXA9* |
| 4017 | *HOXA-AS2* |
| 4018 | *HOXB@* |
| 4019 | *HOXB1* |
| 4020 | *HOXB13* |
| 4021 | *HOXB2* |
| 4022 | *HOXB5* |
| 4023 | *HOXB6* |
| 4024 | *HOXB7* |
| 4025 | *HOXB8* |
| 4026 | *HOXB9* |
| 4027 | *HOXB-AS3* |
| 4028 | *HOXC10* |
| 4029 | *HOXC6* |
| 4030 | *HOXC9* |
| 4031 | *HOXD@* |
| 4032 | *HOXD1* |
| 4033 | *HOXD10* |
| 4034 | *HOXD13* |
| 4035 | *HOXD3* |
| 4036 | *HOXD8* |
| 4037 | *HOXD9* |
| 4038 | *HP* |
| 4039 | *HP1BP3* |
| 4040 | *HPC14* |
| 4041 | *HPCA* |
| 4042 | *HPF1* |
| 4043 | *HPGD* |
| 4044 | *HPGDS* |
| 4045 | *HPP1* |
| 4046 | *HPRT1* |
| 4047 | *HPS1* |
| 4048 | *HPS3* |
| 4049 | *HPS5* |
| 4050 | *HPSE* |
| 4051 | *HPSE2* |
| 4052 | *HPX* |
| 4053 | *HR* |
| 4054 | *HRAS* |
| 4055 | *HRG* |
| 4056 | *HRH1* |
| 4057 | *HRH2* |
| 4058 | *HRH4* |
| 4059 | *HRK* |
| 4060 | *HRNR* |
| 4061 | *HS3ST2* |
| 4062 | *HS3ST3B1* |
| 4063 | *HSD11B1* |
| 4064 | *HSD11B2* |
| 4065 | *HSD17B1* |
| 4066 | *HSD17B10* |
| 4067 | *HSD17B11* |
| 4068 | *HSD17B12* |
| 4069 | *HSD17B13* |
| 4070 | *HSD17B2* |
| 4071 | *HSD17B3* |
| 4072 | *HSD17B4* |
| 4073 | *HSD17B7* |
| 4074 | *HSD17B8* |
| 4075 | *HSD3B1* |
| 4076 | *HSD3B2* |
| 4077 | *HSDL2* |
| 4078 | *HSF1* |
| 4079 | *HSF2* |
| 4080 | *HSF4* |
| 4081 | *HSP90AA1* |
| 4082 | *HSP90AA2P* |
| 4083 | *HSP90AB1* |
| 4084 | *HSP90AB2P* |
| 4085 | *HSP90AB3P* |
| 4086 | *HSP90B1* |
| 4087 | *HSP90B2P* |
| 4088 | *HSPA12A* |
| 4089 | *HSPA14* |
| 4090 | *HSPA1A* |
| 4091 | *HSPA1B* |
| 4092 | *HSPA1L* |
| 4093 | *HSPA2* |
| 4094 | *HSPA4* |
| 4095 | *HSPA4L* |
| 4096 | *HSPA5* |
| 4097 | *HSPA6* |
| 4098 | *HSPA7* |
| 4099 | *HSPA8* |
| 4100 | *HSPA8P14* |
| 4101 | *HSPA9* |
| 4102 | *HSPB1* |
| 4103 | *HSPB2* |
| 4104 | *HSPB3* |
| 4105 | *HSPB6* |
| 4106 | *HSPBP1* |
| 4107 | *HSPD1* |
| 4108 | *HSPD1P4* |
| 4109 | *HSPE1* |
| 4110 | *HSPG2* |
| 4111 | *HSPH1* |
| 4112 | *HTATIP2* |
| 4113 | *HTC2* |
| 4114 | *HTD2* |
| 4115 | *HTN3* |
| 4116 | *HTR1D* |
| 4117 | *HTR1E* |
| 4118 | *HTR2B* |
| 4119 | *HTR3A* |
| 4120 | *HTR3C* |
| 4121 | *HTR5A* |
| 4122 | *HTR7* |
| 4123 | *HTRA1* |
| 4124 | *HTRA2* |
| 4125 | *HTRA3* |
| 4126 | *HULC* |
| 4127 | *HUS1* |
| 4128 | *HUWE1* |
| 4129 | *HVCN1* |
| 4130 | *HYAL1* |
| 4131 | *HYAL2* |
| 4132 | *HYAL3* |
| 4133 | *HYCC1* |
| 4134 | *HYDIN* |
| 4135 | *HYOU1* |
| 4136 | *IAPP* |
| 4137 | *IARS1* |
| 4138 | *IARS2* |
| 4139 | *IATPR* |
| 4140 | *IBD27* |
| 4141 | *IBSP* |
| 4142 | *ICA1* |
| 4143 | *ICAM1* |
| 4144 | *ICAM5* |
| 4145 | *ICMT* |
| 4146 | *ICOS* |
| 4147 | *ICOSLG* |
| 4148 | *ID1* |
| 4149 | *ID2* |
| 4150 | *ID3* |
| 4151 | *ID4* |
| 4152 | *IDH1* |
| 4153 | *IDH2* |
| 4154 | *IDH3B* |
| 4155 | *IDH3G* |
| 4156 | *IDO1* |
| 4157 | *IDO2* |
| 4158 | *IDUA* |
| 4159 | *IER2* |
| 4160 | *IER3* |
| 4161 | *IER3IP1* |
| 4162 | *IER5L* |
| 4163 | *IFFO1* |
| 4164 | *IFI27* |
| 4165 | *IFI6* |
| 4166 | *IFIH1* |
| 4167 | *IFIT2* |
| 4168 | *IFITM1* |
| 4169 | *IFITM2* |
| 4170 | *IFITM3* |
| 4171 | *IFITM4P* |
| 4172 | *IFNA1* |
| 4173 | *IFNA13* |
| 4174 | *IFNA2* |
| 4175 | *IFNAR1* |
| 4176 | *IFNAR2* |
| 4177 | *IFNB1* |
| 4178 | *IFNG* |
| 4179 | *IFNGR1* |
| 4180 | *IFNGR2* |
| 4181 | *IFNL1* |
| 4182 | *IFNL2* |
| 4183 | *IFNL3* |
| 4184 | *IFRD1* |
| 4185 | *IFT172* |
| 4186 | *IFT20* |
| 4187 | *IFT57* |
| 4188 | *IFT74* |
| 4189 | *IFT80* |
| 4190 | *IGDCC3* |
| 4191 | *IGDCC4* |
| 4192 | *IGF1* |
| 4193 | *IGF1R* |
| 4194 | *IGF2* |
| 4195 | *IGF2-AS* |
| 4196 | *IGF2BP1* |
| 4197 | *IGF2BP2* |
| 4198 | *IGF2BP3* |
| 4199 | *IGF2R* |
| 4200 | *IGFBP1* |
| 4201 | *IGFBP2* |
| 4202 | *IGFBP3* |
| 4203 | *IGFBP4* |
| 4204 | *IGFBP5* |
| 4205 | *IGFBP6* |
| 4206 | *IGFBP7* |
| 4207 | *IGHA1* |
| 4208 | *IGHA2* |
| 4209 | *IGHD1-14* |
| 4210 | *IGHG1* |
| 4211 | *IGHV3-7* |
| 4212 | *IGK* |
| 4213 | *IGKC* |
| 4214 | *IGKV2-28* |
| 4215 | *IGKV4-1* |
| 4216 | *IGLC2* |
| 4217 | *IGSF11* |
| 4218 | *IGSF21* |
| 4219 | *IGSF9* |
| 4220 | *IHH* |
| 4221 | *IKBKB* |
| 4222 | *IKBKE* |
| 4223 | *IKBKG* |
| 4224 | *IKZF1* |
| 4225 | *IKZF2* |
| 4226 | *IKZF3* |
| 4227 | *IL10* |
| 4228 | *IL10RA* |
| 4229 | *IL10RB* |
| 4230 | *IL11* |
| 4231 | *IL11RA* |
| 4232 | *IL12A* |
| 4233 | *IL12B* |
| 4234 | *IL12RB1* |
| 4235 | *IL13* |
| 4236 | *IL13RA1* |
| 4237 | *IL13RA2* |
| 4238 | *IL15* |
| 4239 | *IL15RA* |
| 4240 | *IL16* |
| 4241 | *IL17A* |
| 4242 | *IL17B* |
| 4243 | *IL17C* |
| 4244 | *IL17D* |
| 4245 | *IL17F* |
| 4246 | *IL17RA* |
| 4247 | *IL17RB* |
| 4248 | *IL17RC* |
| 4249 | *IL17RD* |
| 4250 | *IL18* |
| 4251 | *IL18R1* |
| 4252 | *IL1A* |
| 4253 | *IL1B* |
| 4254 | *IL1F10* |
| 4255 | *IL1R1* |
| 4256 | *IL1R2* |
| 4257 | *IL1RAPL2* |
| 4258 | *IL1RL1* |
| 4259 | *IL1RN* |
| 4260 | *IL2* |
| 4261 | *IL20RA* |
| 4262 | *IL21* |
| 4263 | *IL21R* |
| 4264 | *IL22* |
| 4265 | *IL22RA1* |
| 4266 | *IL22RA2* |
| 4267 | *IL23A* |
| 4268 | *IL23R* |
| 4269 | *IL24* |
| 4270 | *IL27* |
| 4271 | *IL2RA* |
| 4272 | *IL2RB* |
| 4273 | *IL2RG* |
| 4274 | *IL3* |
| 4275 | *IL31* |
| 4276 | *IL31RA* |
| 4277 | *IL32* |
| 4278 | *IL33* |
| 4279 | *IL34* |
| 4280 | *IL36A* |
| 4281 | *IL36B* |
| 4282 | *IL36G* |
| 4283 | *IL37* |
| 4284 | *IL4* |
| 4285 | *IL4R* |
| 4286 | *IL5* |
| 4287 | *IL6* |
| 4288 | *IL6R* |
| 4289 | *IL6ST* |
| 4290 | *IL7* |
| 4291 | *IL7R* |
| 4292 | *IL9* |
| 4293 | *ILF2* |
| 4294 | *ILF3* |
| 4295 | *ILK* |
| 4296 | *ILKAP* |
| 4297 | *IMMP1L* |
| 4298 | *IMMT* |
| 4299 | *IMP3* |
| 4300 | *IMP4* |
| 4301 | *IMPA2* |
| 4302 | *IMPACT* |
| 4303 | *IMPDH2* |
| 4304 | *INA* |
| 4305 | *INAVA* |
| 4306 | *INCENP* |
| 4307 | *INF2* |
| 4308 | *ING1* |
| 4309 | *ING2* |
| 4310 | *ING3* |
| 4311 | *ING4* |
| 4312 | *ING5* |
| 4313 | *INHA* |
| 4314 | *INHBA* |
| 4315 | *INHBB* |
| 4316 | *INHBE* |
| 4317 | *INO80* |
| 4318 | *INO80C* |
| 4319 | *INPP1* |
| 4320 | *INPP4A* |
| 4321 | *INPP4B* |
| 4322 | *INPP5B* |
| 4323 | *INPP5D* |
| 4324 | *INPP5E* |
| 4325 | *INPP5K* |
| 4326 | *INPPL1* |
| 4327 | *INS* |
| 4328 | *INSIG2* |
| 4329 | *INS-IGF2* |
| 4330 | *INSL5* |
| 4331 | *INSL6* |
| 4332 | *INSR* |
| 4333 | *INSRR* |
| 4334 | *INSYN2A* |
| 4335 | *INTS1* |
| 4336 | *INTS11* |
| 4337 | *INTS13* |
| 4338 | *INTS2* |
| 4339 | *INTS3* |
| 4340 | *INTS5* |
| 4341 | *INTS6* |
| 4342 | *INTS9* |
| 4343 | *INVS* |
| 4344 | *IP6K2* |
| 4345 | *IPMK* |
| 4346 | *IPO11* |
| 4347 | *IPO4* |
| 4348 | *IPO5* |
| 4349 | *IPO7* |
| 4350 | *IPO8* |
| 4351 | *IPO8P1* |
| 4352 | *IPO9* |
| 4353 | *IPP* |
| 4354 | *IQANK1* |
| 4355 | *IQCB1* |
| 4356 | *IQCH* |
| 4357 | *IQCJ-SCHIP1* |
| 4358 | *IQCJ-SCHIP1-AS1* |
| 4359 | *IQCN* |
| 4360 | *IQGAP1* |
| 4361 | *IQGAP2* |
| 4362 | *IQGAP3* |
| 4363 | *IQSEC2* |
| 4364 | *IQUB* |
| 4365 | *IRAG1* |
| 4366 | *IRAK1* |
| 4367 | *IRAK2* |
| 4368 | *IRAK3* |
| 4369 | *IRAK4* |
| 4370 | *IREB2* |
| 4371 | *IRF1* |
| 4372 | *IRF2* |
| 4373 | *IRF2BP2* |
| 4374 | *IRF3* |
| 4375 | *IRF4* |
| 4376 | *IRF5* |
| 4377 | *IRF6* |
| 4378 | *IRF7* |
| 4379 | *IRF8* |
| 4380 | *IRF9* |
| 4381 | *IRS1* |
| 4382 | *IRS2* |
| 4383 | *IRS4* |
| 4384 | *IRX5* |
| 4385 | *ISG15* |
| 4386 | *ISL1* |
| 4387 | *ISLR* |
| 4388 | *ISOC1* |
| 4389 | *IST1* |
| 4390 | *ISX* |
| 4391 | *ISY1-RAB43* |
| 4392 | *ISYNA1* |
| 4393 | *ITCH* |
| 4394 | *ITGA1* |
| 4395 | *ITGA2* |
| 4396 | *ITGA2B* |
| 4397 | *ITGA3* |
| 4398 | *ITGA4* |
| 4399 | *ITGA5* |
| 4400 | *ITGA6* |
| 4401 | *ITGA7* |
| 4402 | *ITGA9* |
| 4403 | *ITGAE* |
| 4404 | *ITGAL* |
| 4405 | *ITGAM* |
| 4406 | *ITGAV* |
| 4407 | *ITGAX* |
| 4408 | *ITGB1* |
| 4409 | *ITGB1BP2* |
| 4410 | *ITGB2* |
| 4411 | *ITGB3* |
| 4412 | *ITGB3BP* |
| 4413 | *ITGB4* |
| 4414 | *ITGB5* |
| 4415 | *ITGB6* |
| 4416 | *ITGB7* |
| 4417 | *ITGB8* |
| 4418 | *ITGBL1* |
| 4419 | *ITIH1* |
| 4420 | *ITIH2* |
| 4421 | *ITIH4* |
| 4422 | *ITIH4-AS1* |
| 4423 | *ITIH5* |
| 4424 | *ITK* |
| 4425 | *ITLN1* |
| 4426 | *ITPA* |
| 4427 | *ITPKA* |
| 4428 | *ITPKC* |
| 4429 | *ITPR1* |
| 4430 | *ITPR2* |
| 4431 | *ITPR3* |
| 4432 | *ITPRID1* |
| 4433 | *ITPRID2* |
| 4434 | *ITSN1* |
| 4435 | *ITSN2* |
| 4436 | *IVD* |
| 4437 | *IVNS1ABP* |
| 4438 | *IWS1* |
| 4439 | *IZUMO1* |
| 4440 | *JADE2* |
| 4441 | *JADE3* |
| 4442 | *JAG1* |
| 4443 | *JAG2* |
| 4444 | *JAK1* |
| 4445 | *JAK2* |
| 4446 | *JAK3* |
| 4447 | *JAKMIP1* |
| 4448 | *JAKMIP2* |
| 4449 | *JAKMIP3* |
| 4450 | *JAM2* |
| 4451 | *JAM3* |
| 4452 | *JARID2* |
| 4453 | *JAZF1* |
| 4454 | *JMJD1C* |
| 4455 | *JMJD4* |
| 4456 | *JMJD8* |
| 4457 | *JMY* |
| 4458 | *JPH1* |
| 4459 | *JPH3* |
| 4460 | *JPH4* |
| 4461 | *JPT1* |
| 4462 | *JRK* |
| 4463 | *JTB* |
| 4464 | *JUN* |
| 4465 | *JUNB* |
| 4466 | *JUND* |
| 4467 | *JUP* |
| 4468 | *KALRN* |
| 4469 | *KANK1* |
| 4470 | *KANK2* |
| 4471 | *KANSL2* |
| 4472 | *KARS1* |
| 4473 | *KAT2A* |
| 4474 | *KAT2B* |
| 4475 | *KAT5* |
| 4476 | *KAT6A* |
| 4477 | *KAT6B* |
| 4478 | *KAT7* |
| 4479 | *KAT8* |
| 4480 | *KATNAL1* |
| 4481 | *KAZN* |
| 4482 | *KBTBD11* |
| 4483 | *KBTBD8* |
| 4484 | *KCNA1* |
| 4485 | *KCNA10* |
| 4486 | *KCNA3* |
| 4487 | *KCNA5* |
| 4488 | *KCNAB2* |
| 4489 | *KCNB1* |
| 4490 | *KCNB2* |
| 4491 | *KCNC2* |
| 4492 | *KCNC4* |
| 4493 | *KCND3* |
| 4494 | *KCNH1* |
| 4495 | *KCNH2* |
| 4496 | *KCNH4* |
| 4497 | *KCNIP3* |
| 4498 | *KCNIP4* |
| 4499 | *KCNJ1* |
| 4500 | *KCNJ14* |
| 4501 | *KCNJ15* |
| 4502 | *KCNK12* |
| 4503 | *KCNK9* |
| 4504 | *KCNMA1* |
| 4505 | *KCNN3* |
| 4506 | *KCNN4* |
| 4507 | *KCNQ1* |
| 4508 | *KCNQ1OT1* |
| 4509 | *KCNQ5* |
| 4510 | *KCNS3* |
| 4511 | *KCNT1* |
| 4512 | *KCNT2* |
| 4513 | *KCTD12* |
| 4514 | *KCTD17* |
| 4515 | *KCTD2* |
| 4516 | *KCTD5* |
| 4517 | *KCTD6* |
| 4518 | *KDELR1* |
| 4519 | *KDF1* |
| 4520 | *KDM1A* |
| 4521 | *KDM1B* |
| 4522 | *KDM2A* |
| 4523 | *KDM2B* |
| 4524 | *KDM3A* |
| 4525 | *KDM3B* |
| 4526 | *KDM4A* |
| 4527 | *KDM4B* |
| 4528 | *KDM4C* |
| 4529 | *KDM4D* |
| 4530 | *KDM5A* |
| 4531 | *KDM5B* |
| 4532 | *KDM5D* |
| 4533 | *KDM6A* |
| 4534 | *KDM6B* |
| 4535 | *KDR* |
| 4536 | *KEAP1* |
| 4537 | *KHDC4* |
| 4538 | *KHDRBS1* |
| 4539 | *KHDRBS3* |
| 4540 | *KHK* |
| 4541 | *KHSRP* |
| 4542 | *KIAA0040* |
| 4543 | *KIAA0319* |
| 4544 | *KIAA0355* |
| 4545 | *KIAA0825* |
| 4546 | *KIAA1191* |
| 4547 | *KIAA1217* |
| 4548 | *KIAA1522* |
| 4549 | *KIAA1614* |
| 4550 | *KIAA1671* |
| 4551 | *KIAA2013* |
| 4552 | *KIDINS220* |
| 4553 | *KIF11* |
| 4554 | *KIF13A* |
| 4555 | *KIF14* |
| 4556 | *KIF15* |
| 4557 | *KIF16B* |
| 4558 | *KIF18A* |
| 4559 | *KIF18B* |
| 4560 | *KIF20A* |
| 4561 | *KIF20B* |
| 4562 | *KIF21B* |
| 4563 | *KIF22* |
| 4564 | *KIF23* |
| 4565 | *KIF24* |
| 4566 | *KIF26B* |
| 4567 | *KIF2A* |
| 4568 | *KIF2C* |
| 4569 | *KIF3A* |
| 4570 | *KIF3B* |
| 4571 | *KIF4A* |
| 4572 | *KIF4B* |
| 4573 | *KIF6* |
| 4574 | *KIF7* |
| 4575 | *KIF9* |
| 4576 | *KIFAP3* |
| 4577 | *KIFC3* |
| 4578 | *KIN* |
| 4579 | *KIR2DL1* |
| 4580 | *KIR2DL2* |
| 4581 | *KIR2DL3* |
| 4582 | *KIR2DS1* |
| 4583 | *KIR2DS2* |
| 4584 | *KIR2DS4* |
| 4585 | *KIR3DL1* |
| 4586 | *KIR3DL2* |
| 4587 | *KIR3DL3* |
| 4588 | *KIR3DX1* |
| 4589 | *KISS1* |
| 4590 | *KISS1R* |
| 4591 | *KIT* |
| 4592 | *KITLG* |
| 4593 | *KL* |
| 4594 | *KLC1* |
| 4595 | *KLC2* |
| 4596 | *KLC4* |
| 4597 | *KLF10* |
| 4598 | *KLF11* |
| 4599 | *KLF12* |
| 4600 | *KLF13* |
| 4601 | *KLF14* |
| 4602 | *KLF15* |
| 4603 | *KLF16* |
| 4604 | *KLF17* |
| 4605 | *KLF2* |
| 4606 | *KLF3* |
| 4607 | *KLF4* |
| 4608 | *KLF5* |
| 4609 | *KLF6* |
| 4610 | *KLF8* |
| 4611 | *KLF9* |
| 4612 | *KLHDC10* |
| 4613 | *KLHDC2* |
| 4614 | *KLHL18* |
| 4615 | *KLHL2* |
| 4616 | *KLHL22* |
| 4617 | *KLHL40* |
| 4618 | *KLHL8* |
| 4619 | *KLK1* |
| 4620 | *KLK10* |
| 4621 | *KLK11* |
| 4622 | *KLK12* |
| 4623 | *KLK15* |
| 4624 | *KLK3* |
| 4625 | *KLK4* |
| 4626 | *KLK5* |
| 4627 | *KLK6* |
| 4628 | *KLK7* |
| 4629 | *KLK8* |
| 4630 | *KLLN* |
| 4631 | *KLRB1* |
| 4632 | *KLRC1* |
| 4633 | *KLRC3* |
| 4634 | *KLRC4-KLRK1* |
| 4635 | *KLRD1* |
| 4636 | *KLRK1* |
| 4637 | *KMT2A* |
| 4638 | *KMT2C* |
| 4639 | *KMT2D* |
| 4640 | *KMT5A* |
| 4641 | *KNG1* |
| 4642 | *KNL1* |
| 4643 | *KPNA1* |
| 4644 | *KPNA2* |
| 4645 | *KPNA3* |
| 4646 | *KPNA5* |
| 4647 | *KPNB1* |
| 4648 | *KRAS* |
| 4649 | *KRASP1* |
| 4650 | *KRBOX4* |
| 4651 | *KREMEN1* |
| 4652 | *KREMEN2* |
| 4653 | *KRIT1* |
| 4654 | *KRR1* |
| 4655 | *KRT1* |
| 4656 | *KRT10* |
| 4657 | *KRT13* |
| 4658 | *KRT14* |
| 4659 | *KRT15* |
| 4660 | *KRT16* |
| 4661 | *KRT17* |
| 4662 | *KRT18* |
| 4663 | *KRT18P56* |
| 4664 | *KRT18P8* |
| 4665 | *KRT19* |
| 4666 | *KRT2* |
| 4667 | *KRT20* |
| 4668 | *KRT23* |
| 4669 | *KRT24* |
| 4670 | *KRT3* |
| 4671 | *KRT33A* |
| 4672 | *KRT33B* |
| 4673 | *KRT35* |
| 4674 | *KRT36* |
| 4675 | *KRT5* |
| 4676 | *KRT6A* |
| 4677 | *KRT6B* |
| 4678 | *KRT7* |
| 4679 | *KRT71* |
| 4680 | *KRT73* |
| 4681 | *KRT76* |
| 4682 | *KRT77* |
| 4683 | *KRT78* |
| 4684 | *KRT79* |
| 4685 | *KRT8* |
| 4686 | *KRT80* |
| 4687 | *KRT82* |
| 4688 | *KRT83* |
| 4689 | *KRT84* |
| 4690 | *KRT85* |
| 4691 | *KRT8P16* |
| 4692 | *KRT9* |
| 4693 | *KRTAP10-1* |
| 4694 | *KRTAP10-8* |
| 4695 | *KRTAP20-1* |
| 4696 | *KRTAP21-1* |
| 4697 | *KRTCAP2* |
| 4698 | *KSR1* |
| 4699 | *KTN1* |
| 4700 | *KTN1-AS1* |
| 4701 | *KYNU* |
| 4702 | *L1CAM* |
| 4703 | *L1TD1* |
| 4704 | *L3HYPDH* |
| 4705 | *L3MBTL1* |
| 4706 | *L3MBTL3* |
| 4707 | *LACTB* |
| 4708 | *LACTB2* |
| 4709 | *LAD1* |
| 4710 | *LAG3* |
| 4711 | *LAGE3* |
| 4712 | *LAIR1* |
| 4713 | *LAIR2* |
| 4714 | *LALBA* |
| 4715 | *LAMA1* |
| 4716 | *LAMA2* |
| 4717 | *LAMA3* |
| 4718 | *LAMA4* |
| 4719 | *LAMA5* |
| 4720 | *LAMB1* |
| 4721 | *LAMB2* |
| 4722 | *LAMB3* |
| 4723 | *LAMB4* |
| 4724 | *LAMC1* |
| 4725 | *LAMC2* |
| 4726 | *LAMC3* |
| 4727 | *LAMP1* |
| 4728 | *LAMP2* |
| 4729 | *LAMP3* |
| 4730 | *LAMTOR2* |
| 4731 | *LAMTOR3* |
| 4732 | *LAMTOR5* |
| 4733 | *LANCL1* |
| 4734 | *LAP* |
| 4735 | *LAP3* |
| 4736 | *LAPTM4B* |
| 4737 | *LARGE2* |
| 4738 | *LARP1* |
| 4739 | *LARP4* |
| 4740 | *LARP4B* |
| 4741 | *LARS1* |
| 4742 | *LARS2* |
| 4743 | *LAS1L* |
| 4744 | *LASP1* |
| 4745 | *LAT* |
| 4746 | *LAT2* |
| 4747 | *LATS1* |
| 4748 | *LATS2* |
| 4749 | *LAYN* |
| 4750 | *LBHD1* |
| 4751 | *LBP* |
| 4752 | *LBR* |
| 4753 | *LBX1* |
| 4754 | *LCAT* |
| 4755 | *LCE1B* |
| 4756 | *LCK* |
| 4757 | *LCN1* |
| 4758 | *LCN1P1* |
| 4759 | *LCN2* |
| 4760 | *LCOR* |
| 4761 | *LCORL* |
| 4762 | *LCP1* |
| 4763 | *LCP2* |
| 4764 | *LCS1* |
| 4765 | *LCT* |
| 4766 | *LCTL* |
| 4767 | *LDB1* |
| 4768 | *LDB2* |
| 4769 | *LDHA* |
| 4770 | *LDHB* |
| 4771 | *LDHC* |
| 4772 | *LDLR* |
| 4773 | *LDLRAD4* |
| 4774 | *LDLRAP1* |
| 4775 | *LDOC1* |
| 4776 | *LECT2* |
| 4777 | *LEF1* |
| 4778 | *LEF1-AS1* |
| 4779 | *LEFTY2* |
| 4780 | *LEMD1* |
| 4781 | *LEMD2* |
| 4782 | *LEMD3* |
| 4783 | *LENG8* |
| 4784 | *LEO1* |
| 4785 | *LEP* |
| 4786 | *LEPQTL1* |
| 4787 | *LEPR* |
| 4788 | *LETM1* |
| 4789 | *LETM2* |
| 4790 | *LETMD1* |
| 4791 | *LFNG* |
| 4792 | *LFS3* |
| 4793 | *LGALS1* |
| 4794 | *LGALS12* |
| 4795 | *LGALS2* |
| 4796 | *LGALS3* |
| 4797 | *LGALS3BP* |
| 4798 | *LGALS4* |
| 4799 | *LGALS7* |
| 4800 | *LGALS8* |
| 4801 | *LGALS9* |
| 4802 | *LGI1* |
| 4803 | *LGI3* |
| 4804 | *LGMN* |
| 4805 | *LGR4* |
| 4806 | *LGR5* |
| 4807 | *LGR6* |
| 4808 | *LGSN* |
| 4809 | *LHCGR* |
| 4810 | *LHFPL6* |
| 4811 | *LHPP* |
| 4812 | *LHX3* |
| 4813 | *LHX4* |
| 4814 | *LHX6* |
| 4815 | *LIF* |
| 4816 | *LIFR* |
| 4817 | *LIFR-AS1* |
| 4818 | *LIG1* |
| 4819 | *LIG3* |
| 4820 | *LIG4* |
| 4821 | *LILRA1* |
| 4822 | *LILRA2* |
| 4823 | *LILRA5* |
| 4824 | *LILRA6* |
| 4825 | *LILRB1* |
| 4826 | *LILRB2* |
| 4827 | *LILRB3* |
| 4828 | *LILRB4* |
| 4829 | *LIMA1* |
| 4830 | *LIMCH1* |
| 4831 | *LIME1* |
| 4832 | *LIMK1* |
| 4833 | *LIMK2* |
| 4834 | *LIMS1* |
| 4835 | *LIMS3* |
| 4836 | *LIMS4* |
| 4837 | *LIN28A* |
| 4838 | *LIN28B* |
| 4839 | *LIN54* |
| 4840 | *LIN7C* |
| 4841 | *LIN9* |
| 4842 | *LINC00052* |
| 4843 | *LINC00174* |
| 4844 | *LINC00265* |
| 4845 | *LINC00273* |
| 4846 | *LINC00312* |
| 4847 | *LINC00328* |
| 4848 | *LINC00355* |
| 4849 | *LINC00365* |
| 4850 | *LINC00400* |
| 4851 | *LINC00446* |
| 4852 | *LINC00460* |
| 4853 | *LINC00467* |
| 4854 | *LINC00472* |
| 4855 | *LINC00473* |
| 4856 | *LINC00488* |
| 4857 | *LINC00511* |
| 4858 | *LINC00649* |
| 4859 | *LINC00654* |
| 4860 | *LINC00659* |
| 4861 | *LINC00668* |
| 4862 | *LINC00673* |
| 4863 | *LINC00707* |
| 4864 | *LINC00858* |
| 4865 | *LINC00909* |
| 4866 | *LINC00957* |
| 4867 | *LINC00958* |
| 4868 | *LINC00996* |
| 4869 | *LINC01121* |
| 4870 | *LINC01133* |
| 4871 | *LINC01173* |
| 4872 | *LINC01194* |
| 4873 | *LINC01234* |
| 4874 | *LINC01354* |
| 4875 | *LINC01475* |
| 4876 | *LINC01500* |
| 4877 | *LINC01503* |
| 4878 | *LINC01555* |
| 4879 | *LINC01630* |
| 4880 | *LINC01672* |
| 4881 | *LINC01708* |
| 4882 | *LINC01917* |
| 4883 | *LINC02264* |
| 4884 | *LINC02418* |
| 4885 | *LINC02605* |
| 4886 | *LINC03057* |
| 4887 | *LINC-ROR* |
| 4888 | *LINS1* |
| 4889 | *LIPA* |
| 4890 | *LIPC* |
| 4891 | *LIPE* |
| 4892 | *LIPF* |
| 4893 | *LIPG* |
| 4894 | *LIPJ* |
| 4895 | *LIPM* |
| 4896 | *LIPN* |
| 4897 | *LITAF* |
| 4898 | *LLGL1* |
| 4899 | *LLGL2* |
| 4900 | *LLPHP3* |
| 4901 | *LMAN1* |
| 4902 | *LMAN2* |
| 4903 | *LMAN2L* |
| 4904 | *LMBR1* |
| 4905 | *LMF2* |
| 4906 | *LMLN* |
| 4907 | *LMNA* |
| 4908 | *LMNB1* |
| 4909 | *LMNB2* |
| 4910 | *LMO1* |
| 4911 | *LMO2* |
| 4912 | *LMO3* |
| 4913 | *LMO4* |
| 4914 | *LMO7* |
| 4915 | *LMOD1* |
| 4916 | *LMTK3* |
| 4917 | *LNPK* |
| 4918 | *LNX1* |
| 4919 | *LNX2* |
| 4920 | *LOC100288966* |
| 4921 | *LOC102724334* |
| 4922 | *LOC102724594* |
| 4923 | *LOC106096416* |
| 4924 | *LOC106099062* |
| 4925 | *LOC106128905* |
| 4926 | *LOC107133510* |
| 4927 | *LOC107303338* |
| 4928 | *LOC107303340* |
| 4929 | *LOC107325936* |
| 4930 | *LOC107372315* |
| 4931 | *LOC107982234* |
| 4932 | *LOC108353817* |
| 4933 | *LOC109029530* |
| 4934 | *LOC109433679* |
| 4935 | *LOC109611593* |
| 4936 | *LOC110006319* |
| 4937 | *LOC110283621* |
| 4938 | *LOC110467515* |
| 4939 | *LOC110594336* |
| 4940 | *LOC110599580* |
| 4941 | *LOC110806262* |
| 4942 | *LOC110806263* |
| 4943 | *LOC110806306* |
| 4944 | *LOC111162620* |
| 4945 | *LOC111188161* |
| 4946 | *LOC111240475* |
| 4947 | *LOC111242783* |
| 4948 | *LOC111255642* |
| 4949 | *LOC111258501* |
| 4950 | *LOC111365141* |
| 4951 | *LOC111365171* |
| 4952 | *LOC111365177* |
| 4953 | *LOC111674464* |
| 4954 | *LOC111674477* |
| 4955 | *LOC112543491* |
| 4956 | *LOC113687181* |
| 4957 | *LOC122455342* |
| 4958 | *LOC122539214* |
| 4959 | *LOC124900868* |
| 4960 | *LOC124901872* |
| 4961 | *LOC124902713* |
| 4962 | *LOC124903151* |
| 4963 | *LOC124903317* |
| 4964 | *LOC124903570* |
| 4965 | *LOC124904805* |
| 4966 | *LONP1* |
| 4967 | *LONRF1* |
| 4968 | *LONRF2* |
| 4969 | *LONRF3* |
| 4970 | *LOX* |
| 4971 | *LOXL1* |
| 4972 | *LOXL2* |
| 4973 | *LOXL3* |
| 4974 | *LOXL4* |
| 4975 | *LPA* |
| 4976 | *LPAR1* |
| 4977 | *LPAR2* |
| 4978 | *LPAR3* |
| 4979 | *LPCAT1* |
| 4980 | *LPCAT2* |
| 4981 | *LPCAT4* |
| 4982 | *LPIN1* |
| 4983 | *LPIN2* |
| 4984 | *LPL* |
| 4985 | *LPP* |
| 4986 | *LRAT* |
| 4987 | *LRATD1* |
| 4988 | *LRATD2* |
| 4989 | *LRBA* |
| 4990 | *LRCH1* |
| 4991 | *LRFN2* |
| 4992 | *LRFN4* |
| 4993 | *LRFN5* |
| 4994 | *LRG1* |
| 4995 | *LRIG1* |
| 4996 | *LRIG3* |
| 4997 | *LRIT1* |
| 4998 | *LRP1* |
| 4999 | *LRP1-AS* |
| 5000 | *LRP2* |
| 5001 | *LRP5* |
| 5002 | *LRP6* |
| 5003 | *LRPAP1* |
| 5004 | *LRPPRC* |
| 5005 | *LRRC17* |
| 5006 | *LRRC23* |
| 5007 | *LRRC28* |
| 5008 | *LRRC32* |
| 5009 | *LRRC34* |
| 5010 | *LRRC37A3* |
| 5011 | *LRRC3B* |
| 5012 | *LRRC4* |
| 5013 | *LRRC41* |
| 5014 | *LRRC47* |
| 5015 | *LRRC4C* |
| 5016 | *LRRC56* |
| 5017 | *LRRC59* |
| 5018 | *LRRC7* |
| 5019 | *LRRC8A* |
| 5020 | *LRRFIP1* |
| 5021 | *LRRFIP2* |
| 5022 | *LRRK1* |
| 5023 | *LRRTM1* |
| 5024 | *LRWD1* |
| 5025 | *LSAMP* |
| 5026 | *LSINCT5* |
| 5027 | *LSM12* |
| 5028 | *LSM14A* |
| 5029 | *LSP1* |
| 5030 | *LSR* |
| 5031 | *LTA* |
| 5032 | *LTA4H* |
| 5033 | *LTB4R* |
| 5034 | *LTB4R2* |
| 5035 | *LTBP1* |
| 5036 | *LTBP2* |
| 5037 | *LTBP3* |
| 5038 | *LTBP4* |
| 5039 | *LTBR* |
| 5040 | *LTC4S* |
| 5041 | *LTF* |
| 5042 | *LUADT1* |
| 5043 | *LUC7L* |
| 5044 | *LUC7L2* |
| 5045 | *LUC7L3* |
| 5046 | *LUCAT1* |
| 5047 | *LUM* |
| 5048 | *LUZP1* |
| 5049 | *LY6E* |
| 5050 | *LY6G6C* |
| 5051 | *LY6G6D* |
| 5052 | *LY6G6F* |
| 5053 | *LY86* |
| 5054 | *LY96* |
| 5055 | *LYAR* |
| 5056 | *LYG1* |
| 5057 | *LYN* |
| 5058 | *LYPD3* |
| 5059 | *LYPD4* |
| 5060 | *LYPD5* |
| 5061 | *LYPD8* |
| 5062 | *LYPLAL1* |
| 5063 | *LYRM2* |
| 5064 | *LYSMD3* |
| 5065 | *LYVE1* |
| 5066 | *LYZ* |
| 5067 | *LZTFL1* |
| 5068 | *LZTS1* |
| 5069 | *LZTS2* |
| 5070 | *MACC1* |
| 5071 | *MACF1* |
| 5072 | *MACROD1* |
| 5073 | *MACROD2* |
| 5074 | *MACROH2A1* |
| 5075 | *MACROH2A2* |
| 5076 | *MAD1L1* |
| 5077 | *MAD2L1* |
| 5078 | *MAD2L1P1* |
| 5079 | *MAD2L2* |
| 5080 | *MADCAM1* |
| 5081 | *MADD* |
| 5082 | *MAEL* |
| 5083 | *MAF* |
| 5084 | *MAF1* |
| 5085 | *MAFB* |
| 5086 | *MAFF* |
| 5087 | *MAFG* |
| 5088 | *MAFG-DT* |
| 5089 | *MAFK* |
| 5090 | *MAGEA1* |
| 5091 | *MAGEA3* |
| 5092 | *MAGEA4* |
| 5093 | *MAGEA6* |
| 5094 | *MAGEB2* |
| 5095 | *MAGEC1* |
| 5096 | *MAGEC2* |
| 5097 | *MAGED1* |
| 5098 | *MAGED2* |
| 5099 | *MAGED4* |
| 5100 | *MAGEE1* |
| 5101 | *MAGI1* |
| 5102 | *MAGI2* |
| 5103 | *MAGI2-AS3* |
| 5104 | *MAGI3* |
| 5105 | *MAGOHB* |
| 5106 | *MAGT1* |
| 5107 | *MAIP1* |
| 5108 | *MAK16* |
| 5109 | *MAL* |
| 5110 | *MAL2* |
| 5111 | *MALAT1* |
| 5112 | *MALL* |
| 5113 | *MALRD1* |
| 5114 | *MALT1* |
| 5115 | *MAMDC4* |
| 5116 | *MAMSTR* |
| 5117 | *MAN2A2* |
| 5118 | *MANBA* |
| 5119 | *MANBAL* |
| 5120 | *MANEA* |
| 5121 | *MANF* |
| 5122 | *MAOA* |
| 5123 | *MAOB* |
| 5124 | *MAP1B* |
| 5125 | *MAP1LC3A* |
| 5126 | *MAP1LC3B* |
| 5127 | *MAP1LC3C* |
| 5128 | *MAP1S* |
| 5129 | *MAP2* |
| 5130 | *MAP2K1* |
| 5131 | *MAP2K2* |
| 5132 | *MAP2K3* |
| 5133 | *MAP2K4* |
| 5134 | *MAP2K5* |
| 5135 | *MAP2K6* |
| 5136 | *MAP2K7* |
| 5137 | *MAP3K1* |
| 5138 | *MAP3K11* |
| 5139 | *MAP3K12* |
| 5140 | *MAP3K14* |
| 5141 | *MAP3K14-AS1* |
| 5142 | *MAP3K15* |
| 5143 | *MAP3K19* |
| 5144 | *MAP3K2* |
| 5145 | *MAP3K20* |
| 5146 | *MAP3K20-AS1* |
| 5147 | *MAP3K21* |
| 5148 | *MAP3K5* |
| 5149 | *MAP3K6* |
| 5150 | *MAP3K7* |
| 5151 | *MAP3K8* |
| 5152 | *MAP4* |
| 5153 | *MAP4K1* |
| 5154 | *MAP4K4* |
| 5155 | *MAP6* |
| 5156 | *MAP7* |
| 5157 | *MAP7D1* |
| 5158 | *MAP7D2* |
| 5159 | *MAP9* |
| 5160 | *MAPK1* |
| 5161 | *MAPK10* |
| 5162 | *MAPK11* |
| 5163 | *MAPK12* |
| 5164 | *MAPK13* |
| 5165 | *MAPK14* |
| 5166 | *MAPK15* |
| 5167 | *MAPK3* |
| 5168 | *MAPK4* |
| 5169 | *MAPK6* |
| 5170 | *MAPK7* |
| 5171 | *MAPK8* |
| 5172 | *MAPK8IP1* |
| 5173 | *MAPK8IP2* |
| 5174 | *MAPK9* |
| 5175 | *MAPKAP1* |
| 5176 | *MAPKAPK2* |
| 5177 | *MAPKAPK3* |
| 5178 | *MAPKAPK5* |
| 5179 | *MAPKAPK5-AS1* |
| 5180 | *MAPRE1* |
| 5181 | *MAPRE2* |
| 5182 | *MAPRE3* |
| 5183 | *MAPT* |
| 5184 | *MARCHF1* |
| 5185 | *MARCHF7* |
| 5186 | *MARCHF8* |
| 5187 | *MARCKS* |
| 5188 | *MARCKSL1* |
| 5189 | *MARK1* |
| 5190 | *MARK2* |
| 5191 | *MARK2P12* |
| 5192 | *MARK3* |
| 5193 | *MARK4* |
| 5194 | *MARS1* |
| 5195 | *MARVELD1* |
| 5196 | *MARVELD2* |
| 5197 | *MAS1* |
| 5198 | *MASP1* |
| 5199 | *MASP2* |
| 5200 | *MAST1* |
| 5201 | *MAST2* |
| 5202 | *MASTL* |
| 5203 | *MAT1A* |
| 5204 | *MAT2A* |
| 5205 | *MAT2B* |
| 5206 | *MATK* |
| 5207 | *MATN1* |
| 5208 | *MATN2* |
| 5209 | *MATN3* |
| 5210 | *MATR3* |
| 5211 | *MAU2* |
| 5212 | *MAX* |
| 5213 | *MAZ* |
| 5214 | *MB* |
| 5215 | *MBD1* |
| 5216 | *MBD2* |
| 5217 | *MBD4* |
| 5218 | *MBD6* |
| 5219 | *MBIP* |
| 5220 | *MBL2* |
| 5221 | *MBLAC2* |
| 5222 | *MBNL1* |
| 5223 | *MBOAT7* |
| 5224 | *MBP* |
| 5225 | *MBTPS1* |
| 5226 | *MC1R* |
| 5227 | *MC2R* |
| 5228 | *MC4R* |
| 5229 | *MCAM* |
| 5230 | *MCAT* |
| 5231 | *MCC* |
| 5232 | *MCCC1* |
| 5233 | *MCCC2* |
| 5234 | *MCF2* |
| 5235 | *MCF2L* |
| 5236 | *MCF2L2* |
| 5237 | *MCL1* |
| 5238 | *MCM2* |
| 5239 | *MCM3* |
| 5240 | *MCM3AP* |
| 5241 | *MCM4* |
| 5242 | *MCM5* |
| 5243 | *MCM6* |
| 5244 | *MCM7* |
| 5245 | *MCM8* |
| 5246 | *MCM9* |
| 5247 | *MCMBP* |
| 5248 | *MCOLN1* |
| 5249 | *MCPH1* |
| 5250 | *MCRS1* |
| 5251 | *MCTS1* |
| 5252 | *MCU* |
| 5253 | *MDC1* |
| 5254 | *MDFI* |
| 5255 | *MDFIC* |
| 5256 | *MDFIC2* |
| 5257 | *MDH1* |
| 5258 | *MDH2* |
| 5259 | *MDK* |
| 5260 | *MDM2* |
| 5261 | *MDM4* |
| 5262 | *ME1* |
| 5263 | *MEAF6* |
| 5264 | *MECOM* |
| 5265 | *MECP2* |
| 5266 | *MECR* |
| 5267 | *MED1* |
| 5268 | *MED10* |
| 5269 | *MED12* |
| 5270 | *MED13* |
| 5271 | *MED13L* |
| 5272 | *MED14* |
| 5273 | *MED15* |
| 5274 | *MED19* |
| 5275 | *MED22* |
| 5276 | *MED23* |
| 5277 | *MED24* |
| 5278 | *MED25* |
| 5279 | *MED26* |
| 5280 | *MED28* |
| 5281 | *MED31* |
| 5282 | *MED4* |
| 5283 | *MED8* |
| 5284 | *MEF2A* |
| 5285 | *MEF2C* |
| 5286 | *MEF2D* |
| 5287 | *MEG3* |
| 5288 | *MEG8* |
| 5289 | *MEGF6* |
| 5290 | *MEGF8* |
| 5291 | *MEIS1* |
| 5292 | *MEIS2* |
| 5293 | *MEIS3* |
| 5294 | *MELK* |
| 5295 | *MELTF* |
| 5296 | *MELTF-AS1* |
| 5297 | *MEMO1* |
| 5298 | *MEMO1P1* |
| 5299 | *MEN1* |
| 5300 | *MEP1A* |
| 5301 | *MEP1B* |
| 5302 | *MEPCE* |
| 5303 | *MERTK* |
| 5304 | *MESD* |
| 5305 | *MET* |
| 5306 | *METAP1* |
| 5307 | *METAP2* |
| 5308 | *METRN* |
| 5309 | *METRNL* |
| 5310 | *METTL14* |
| 5311 | *METTL15* |
| 5312 | *METTL16* |
| 5313 | *METTL17* |
| 5314 | *METTL3* |
| 5315 | *METTL7B* |
| 5316 | *MEX3A* |
| 5317 | *MEX3B* |
| 5318 | *MEX3C* |
| 5319 | *MFAP1* |
| 5320 | *MFAP2* |
| 5321 | *MFAP3* |
| 5322 | *MFAP3L* |
| 5323 | *MFAP4* |
| 5324 | *MFAP5* |
| 5325 | *MFF* |
| 5326 | *MFGE8* |
| 5327 | *MFHAS1* |
| 5328 | *MFN1* |
| 5329 | *MFN2* |
| 5330 | *MFNG* |
| 5331 | *MFSD1* |
| 5332 | *MFSD11* |
| 5333 | *MFSD13A* |
| 5334 | *MFSD14B* |
| 5335 | *MFSD4B* |
| 5336 | *MFSD9* |
| 5337 | *MGA* |
| 5338 | *MGAM* |
| 5339 | *MGAT4A* |
| 5340 | *MGAT5* |
| 5341 | *MGAT5B* |
| 5342 | *MGLL* |
| 5343 | *MGMT* |
| 5344 | *MGP* |
| 5345 | *MGRN1* |
| 5346 | *MGST1* |
| 5347 | *MGST3* |
| 5348 | *MHS2* |
| 5349 | *MIA* |
| 5350 | *MIA2* |
| 5351 | *MIA3* |
| 5352 | *MIAT* |
| 5353 | *MIB1* |
| 5354 | *MIB2* |
| 5355 | *MICA* |
| 5356 | *MICAL1* |
| 5357 | *MICAL2* |
| 5358 | *MICALL1* |
| 5359 | *MICB* |
| 5360 | *MICC* |
| 5361 | *MICE* |
| 5362 | *MICU1* |
| 5363 | *MICU2* |
| 5364 | *MID1* |
| 5365 | *MID1IP1* |
| 5366 | *MIEF1* |
| 5367 | *MIEN1* |
| 5368 | *MIER1* |
| 5369 | *MIER2* |
| 5370 | *MIER3* |
| 5371 | *MIF* |
| 5372 | *MIF4GD* |
| 5373 | *MIGA2* |
| 5374 | *MIIP* |
| 5375 | *MINAR1* |
| 5376 | *MINDY4* |
| 5377 | *MINK1* |
| 5378 | *MIOS* |
| 5379 | *MIP* |
| 5380 | *MIPEP* |
| 5381 | *MIPEPP2* |
| 5382 | *MIR100* |
| 5383 | *MIR100HG* |
| 5384 | *MIR103A2* |
| 5385 | *MIR106A* |
| 5386 | *MIR106B* |
| 5387 | *MIR107* |
| 5388 | *MIR10A* |
| 5389 | *MIR10B* |
| 5390 | *MIR1-1* |
| 5391 | *MIR1179* |
| 5392 | *MIR1182* |
| 5393 | *MIR122* |
| 5394 | *MIR1229* |
| 5395 | *MIR1236* |
| 5396 | *MIR124-1* |
| 5397 | *MIR1246* |
| 5398 | *MIR1247* |
| 5399 | *MIR1249* |
| 5400 | *MIR1256* |
| 5401 | *MIR1258* |
| 5402 | *MIR125A* |
| 5403 | *MIR125B1* |
| 5404 | *MIR126* |
| 5405 | *MIR1260B* |
| 5406 | *MIR1266* |
| 5407 | *MIR1271* |
| 5408 | *MIR1273C* |
| 5409 | *MIR1275* |
| 5410 | *MIR1288* |
| 5411 | *MIR1290* |
| 5412 | *MIR1295A* |
| 5413 | *MIR1296* |
| 5414 | *MIR1297* |
| 5415 | *MIR1307* |
| 5416 | *MIR130A* |
| 5417 | *MIR130B* |
| 5418 | *MIR132* |
| 5419 | *MIR133A2* |
| 5420 | *MIR133B* |
| 5421 | *MIR134* |
| 5422 | *MIR135B* |
| 5423 | *MIR136* |
| 5424 | *MIR137* |
| 5425 | *MIR139* |
| 5426 | *MIR140* |
| 5427 | *MIR141* |
| 5428 | *MIR142* |
| 5429 | *MIR143* |
| 5430 | *MIR144* |
| 5431 | *MIR145* |
| 5432 | *MIR146A* |
| 5433 | *MIR146B* |
| 5434 | *MIR147B* |
| 5435 | *MIR148A* |
| 5436 | *MIR148B* |
| 5437 | *MIR149* |
| 5438 | *MIR150* |
| 5439 | *MIR152* |
| 5440 | *MIR153-1* |
| 5441 | *MIR154* |
| 5442 | *MIR155* |
| 5443 | *MIR155HG* |
| 5444 | *MIR15A* |
| 5445 | *MIR15B* |
| 5446 | *MIR17* |
| 5447 | *MIR17HG* |
| 5448 | *MIR181A2* |
| 5449 | *MIR181C* |
| 5450 | *MIR181D* |
| 5451 | *MIR182* |
| 5452 | *MIR1827* |
| 5453 | *MIR183* |
| 5454 | *MIR184* |
| 5455 | *MIR185* |
| 5456 | *MIR186* |
| 5457 | *MIR187* |
| 5458 | *MIR188* |
| 5459 | *MIR18A* |
| 5460 | *MIR18B* |
| 5461 | *MIR190A* |
| 5462 | *MIR191* |
| 5463 | *MIR1910* |
| 5464 | *MIR1914* |
| 5465 | *MIR192* |
| 5466 | *MIR193A* |
| 5467 | *MIR193B* |
| 5468 | *MIR195* |
| 5469 | *MIR196A1* |
| 5470 | *MIR196A2* |
| 5471 | *MIR196B* |
| 5472 | *MIR197* |
| 5473 | *MIR1976* |
| 5474 | *MIR198* |
| 5475 | *MIR199A1* |
| 5476 | *MIR199A2* |
| 5477 | *MIR199B* |
| 5478 | *MIR19A* |
| 5479 | *MIR19B1* |
| 5480 | *MIR200A* |
| 5481 | *MIR200B* |
| 5482 | *MIR200C* |
| 5483 | *MIR202* |
| 5484 | *MIR203A* |
| 5485 | *MIR204* |
| 5486 | *MIR205* |
| 5487 | *MIR206* |
| 5488 | *MIR20A* |
| 5489 | *MIR20B* |
| 5490 | *MIR21* |
| 5491 | *MIR210* |
| 5492 | *MIR211* |
| 5493 | *MIR212* |
| 5494 | *MIR214* |
| 5495 | *MIR215* |
| 5496 | *MIR216A* |
| 5497 | *MIR216B* |
| 5498 | *MIR217* |
| 5499 | *MIR219A1* |
| 5500 | *MIR22* |
| 5501 | *MIR221* |
| 5502 | *MIR222* |
| 5503 | *MIR223* |
| 5504 | *MIR224* |
| 5505 | *MIR23A* |
| 5506 | *MIR23B* |
| 5507 | *MIR24-1* |
| 5508 | *MIR24-2* |
| 5509 | *MIR2467* |
| 5510 | *MIR25* |
| 5511 | *MIR26A1* |
| 5512 | *MIR26B* |
| 5513 | *MIR27A* |
| 5514 | *MIR27B* |
| 5515 | *MIR28* |
| 5516 | *MIR296* |
| 5517 | *MIR297* |
| 5518 | *MIR298* |
| 5519 | *MIR29A* |
| 5520 | *MIR29B1* |
| 5521 | *MIR29B2* |
| 5522 | *MIR29C* |
| 5523 | *MIR301A* |
| 5524 | *MIR302A* |
| 5525 | *MIR302C* |
| 5526 | *MIR30A* |
| 5527 | *MIR30B* |
| 5528 | *MIR30C1* |
| 5529 | *MIR30C2* |
| 5530 | *MIR30D* |
| 5531 | *MIR30E* |
| 5532 | *MIR31* |
| 5533 | *MIR3150B* |
| 5534 | *MIR3163* |
| 5535 | *MIR3191* |
| 5536 | *MIR31HG* |
| 5537 | *MIR32* |
| 5538 | *MIR320A* |
| 5539 | *MIR323A* |
| 5540 | *MIR324* |
| 5541 | *MIR326* |
| 5542 | *MIR328* |
| 5543 | *MIR330* |
| 5544 | *MIR331* |
| 5545 | *MIR335* |
| 5546 | *MIR337* |
| 5547 | *MIR338* |
| 5548 | *MIR339* |
| 5549 | *MIR33A* |
| 5550 | *MIR33B* |
| 5551 | *MIR340* |
| 5552 | *MIR342* |
| 5553 | *MIR345* |
| 5554 | *MIR34A* |
| 5555 | *MIR34B* |
| 5556 | *MIR34C* |
| 5557 | *MIR361* |
| 5558 | *MIR362* |
| 5559 | *MIR3621* |
| 5560 | *MIR363* |
| 5561 | *MIR365B* |
| 5562 | *MIR3666* |
| 5563 | *MIR367* |
| 5564 | *MIR3678* |
| 5565 | *MIR3679* |
| 5566 | *MIR369* |
| 5567 | *MIR370* |
| 5568 | *MIR372* |
| 5569 | *MIR373* |
| 5570 | *MIR374A* |
| 5571 | *MIR374B* |
| 5572 | *MIR375* |
| 5573 | *MIR376A1* |
| 5574 | *MIR378A* |
| 5575 | *MIR378F* |
| 5576 | *MIR382* |
| 5577 | *MIR383* |
| 5578 | *MIR384* |
| 5579 | *MIR3940* |
| 5580 | *MIR409* |
| 5581 | *MIR410* |
| 5582 | *MIR411* |
| 5583 | *MIR421* |
| 5584 | *MIR422A* |
| 5585 | *MIR423* |
| 5586 | *MIR424* |
| 5587 | *MIR425* |
| 5588 | *MIR4260* |
| 5589 | *MIR4261* |
| 5590 | *MIR4282* |
| 5591 | *MIR429* |
| 5592 | *MIR431* |
| 5593 | *MIR4316* |
| 5594 | *MIR4319* |
| 5595 | *MIR433* |
| 5596 | *MIR4435-2HG* |
| 5597 | *MIR4474* |
| 5598 | *MIR4478* |
| 5599 | *MIR448* |
| 5600 | *MIR4487* |
| 5601 | *MIR4492* |
| 5602 | *MIR449A* |
| 5603 | *MIR4500* |
| 5604 | *MIR450B* |
| 5605 | *MIR451A* |
| 5606 | *MIR452* |
| 5607 | *MIR454* |
| 5608 | *MIR455* |
| 5609 | *MIR4658* |
| 5610 | *MIR466* |
| 5611 | *MIR4666A* |
| 5612 | *MIR4717* |
| 5613 | *MIR4775* |
| 5614 | *MIR483* |
| 5615 | *MIR484* |
| 5616 | *MIR485* |
| 5617 | *MIR486-1* |
| 5618 | *MIR487B* |
| 5619 | *MIR488* |
| 5620 | *MIR489* |
| 5621 | *MIR490* |
| 5622 | *MIR491* |
| 5623 | *MIR492* |
| 5624 | *MIR493* |
| 5625 | *MIR494* |
| 5626 | *MIR495* |
| 5627 | *MIR496* |
| 5628 | *MIR497* |
| 5629 | *MIR498* |
| 5630 | *MIR499A* |
| 5631 | *MIR500A* |
| 5632 | *MIR501* |
| 5633 | *MIR502* |
| 5634 | *MIR503* |
| 5635 | *MIR503HG* |
| 5636 | *MIR505* |
| 5637 | *MIR506* |
| 5638 | *MIR508* |
| 5639 | *MIR511* |
| 5640 | *MIR514B* |
| 5641 | *MIR5191* |
| 5642 | *MIR519B* |
| 5643 | *MIR519C* |
| 5644 | *MIR519D* |
| 5645 | *MIR520B* |
| 5646 | *MIR520E* |
| 5647 | *MIR520G* |
| 5648 | *MIR522* |
| 5649 | *MIR524* |
| 5650 | *MIR532* |
| 5651 | *MIR539* |
| 5652 | *MIR542* |
| 5653 | *MIR543* |
| 5654 | *MIR544A* |
| 5655 | *MIR545* |
| 5656 | *MIR551B* |
| 5657 | *MIR552* |
| 5658 | *MIR567* |
| 5659 | *MIR568* |
| 5660 | *MIR572* |
| 5661 | *MIR574* |
| 5662 | *MIR577* |
| 5663 | *MIR579* |
| 5664 | *MIR582* |
| 5665 | *MIR584* |
| 5666 | *MIR585* |
| 5667 | *MIR587* |
| 5668 | *MIR590* |
| 5669 | *MIR592* |
| 5670 | *MIR595* |
| 5671 | *MIR597* |
| 5672 | *MIR598* |
| 5673 | *MIR600* |
| 5674 | *MIR601* |
| 5675 | *MIR603* |
| 5676 | *MIR605* |
| 5677 | *MIR608* |
| 5678 | *MIR612* |
| 5679 | *MIR6165* |
| 5680 | *MIR618* |
| 5681 | *MIR622* |
| 5682 | *MIR625* |
| 5683 | *MIR627* |
| 5684 | *MIR629* |
| 5685 | *MIR630* |
| 5686 | *MIR637* |
| 5687 | *MIR638* |
| 5688 | *MIR642B* |
| 5689 | *MIR646* |
| 5690 | *MIR647* |
| 5691 | *MIR650* |
| 5692 | *MIR663A* |
| 5693 | *MIR663B* |
| 5694 | *MIR6716* |
| 5695 | *MIR675* |
| 5696 | *MIR6778* |
| 5697 | *MIR6787* |
| 5698 | *MIR6803* |
| 5699 | *MIR6852* |
| 5700 | *MIR6868* |
| 5701 | *MIR6869* |
| 5702 | *MIR708* |
| 5703 | *MIR7-1* |
| 5704 | *MIR7-2* |
| 5705 | *MIR7-3* |
| 5706 | *MIR744* |
| 5707 | *MIR760* |
| 5708 | *MIR761* |
| 5709 | *MIR766* |
| 5710 | *MIR769* |
| 5711 | *MIR7702* |
| 5712 | *MIR8064* |
| 5713 | *MIR8075* |
| 5714 | *MIR873* |
| 5715 | *MIR874* |
| 5716 | *MIR875* |
| 5717 | *MIR877* |
| 5718 | *MIR885* |
| 5719 | *MIR888* |
| 5720 | *MIR889* |
| 5721 | *MIR92B* |
| 5722 | *MIR93* |
| 5723 | *MIR935* |
| 5724 | *MIR938* |
| 5725 | *MIR939* |
| 5726 | *MIR940* |
| 5727 | *MIR942* |
| 5728 | *MIR944* |
| 5729 | *MIR95* |
| 5730 | *MIR96* |
| 5731 | *MIR98* |
| 5732 | *MIR99A* |
| 5733 | *MIR99B* |
| 5734 | *MIRLET7B* |
| 5735 | *MIRLET7C* |
| 5736 | *MIRLET7D* |
| 5737 | *MIRLET7E* |
| 5738 | *MIRLET7I* |
| 5739 | *MIS18A* |
| 5740 | *MISP* |
| 5741 | *MITF* |
| 5742 | *MIX23P2* |
| 5743 | *MKI67* |
| 5744 | *MKLN1* |
| 5745 | *MKNK1* |
| 5746 | *MKRN1* |
| 5747 | *MKRN2* |
| 5748 | *MKRN3* |
| 5749 | *MLANA* |
| 5750 | *MLC1* |
| 5751 | *MLF2* |
| 5752 | *MLH1* |
| 5753 | *MLH3* |
| 5754 | *MLKL* |
| 5755 | *MLLT10* |
| 5756 | *MLLT11* |
| 5757 | *MLLT6* |
| 5758 | *MLN* |
| 5759 | *MLNR* |
| 5760 | *MLRL* |
| 5761 | *MLST8* |
| 5762 | *MLX* |
| 5763 | *MLXIP* |
| 5764 | *MLXIPL* |
| 5765 | *MMAA* |
| 5766 | *MME* |
| 5767 | *MMP1* |
| 5768 | *MMP10* |
| 5769 | *MMP11* |
| 5770 | *MMP12* |
| 5771 | *MMP13* |
| 5772 | *MMP14* |
| 5773 | *MMP15* |
| 5774 | *MMP16* |
| 5775 | *MMP19* |
| 5776 | *MMP2* |
| 5777 | *MMP21* |
| 5778 | *MMP23A* |
| 5779 | *MMP23B* |
| 5780 | *MMP25* |
| 5781 | *MMP26* |
| 5782 | *MMP28* |
| 5783 | *MMP3* |
| 5784 | *MMP7* |
| 5785 | *MMP8* |
| 5786 | *MMP9* |
| 5787 | *MMRN1* |
| 5788 | *MMRN2* |
| 5789 | *MMS19* |
| 5790 | *MMUT* |
| 5791 | *MN1* |
| 5792 | *MNAT1* |
| 5793 | *MNS1* |
| 5794 | *MNS16A* |
| 5795 | *MNX1* |
| 5796 | *MNX1-AS1* |
| 5797 | *MOAP1* |
| 5798 | *MOB1A* |
| 5799 | *MOB2* |
| 5800 | *MOB4* |
| 5801 | *MOCS1* |
| 5802 | *MOG* |
| 5803 | *MOGS* |
| 5804 | *MOK* |
| 5805 | *MON1A* |
| 5806 | *MON2* |
| 5807 | *MORC1* |
| 5808 | *MORC2* |
| 5809 | *MORC4* |
| 5810 | *MORF4L2* |
| 5811 | *MOSPD1* |
| 5812 | *MOSPD3* |
| 5813 | *MOV10* |
| 5814 | *MPC1* |
| 5815 | *MPC2* |
| 5816 | *MPEG1* |
| 5817 | *MPG* |
| 5818 | *MPHOSPH8* |
| 5819 | *MPHOSPH9* |
| 5820 | *MPL* |
| 5821 | *MPLKIP* |
| 5822 | *MPO* |
| 5823 | *MPP3* |
| 5824 | *MPP7* |
| 5825 | *MPPE1* |
| 5826 | *MPPED1* |
| 5827 | *MPPED2* |
| 5828 | *MPRIP* |
| 5829 | *MPST* |
| 5830 | *MPV17* |
| 5831 | *MPZ* |
| 5832 | *MPZL1* |
| 5833 | *MRAS* |
| 5834 | *MRC1* |
| 5835 | *MRE11* |
| 5836 | *MRGBP* |
| 5837 | *MRGPRX1* |
| 5838 | *MRGPRX3* |
| 5839 | *MRGPRX4* |
| 5840 | *MRO* |
| 5841 | *MROH7* |
| 5842 | *MRPL10* |
| 5843 | *MRPL11* |
| 5844 | *MRPL12* |
| 5845 | *MRPL13* |
| 5846 | *MRPL15* |
| 5847 | *MRPL16* |
| 5848 | *MRPL17* |
| 5849 | *MRPL18* |
| 5850 | *MRPL2* |
| 5851 | *MRPL20* |
| 5852 | *MRPL22* |
| 5853 | *MRPL23* |
| 5854 | *MRPL24* |
| 5855 | *MRPL28* |
| 5856 | *MRPL3* |
| 5857 | *MRPL32* |
| 5858 | *MRPL33* |
| 5859 | *MRPL35* |
| 5860 | *MRPL38* |
| 5861 | *MRPL39* |
| 5862 | *MRPL4* |
| 5863 | *MRPL41* |
| 5864 | *MRPL42* |
| 5865 | *MRPL42P4* |
| 5866 | *MRPL43* |
| 5867 | *MRPL44* |
| 5868 | *MRPL45* |
| 5869 | *MRPL47* |
| 5870 | *MRPL49* |
| 5871 | *MRPL50* |
| 5872 | *MRPL53* |
| 5873 | *MRPL55* |
| 5874 | *MRPL57* |
| 5875 | *MRPL57P9* |
| 5876 | *MRPL58* |
| 5877 | *MRPS10* |
| 5878 | *MRPS12* |
| 5879 | *MRPS14* |
| 5880 | *MRPS15* |
| 5881 | *MRPS17* |
| 5882 | *MRPS18A* |
| 5883 | *MRPS18C* |
| 5884 | *MRPS2* |
| 5885 | *MRPS21* |
| 5886 | *MRPS22* |
| 5887 | *MRPS23* |
| 5888 | *MRPS24* |
| 5889 | *MRPS25* |
| 5890 | *MRPS26* |
| 5891 | *MRPS27* |
| 5892 | *MRPS28* |
| 5893 | *MRPS30* |
| 5894 | *MRPS31* |
| 5895 | *MRPS31P1* |
| 5896 | *MRPS31P5* |
| 5897 | *MRPS33* |
| 5898 | *MRPS34* |
| 5899 | *MRPS35* |
| 5900 | *MRPS5* |
| 5901 | *MRPS6* |
| 5902 | *MRPS7* |
| 5903 | *MRPS9* |
| 5904 | *MRTFA* |
| 5905 | *MRTFB* |
| 5906 | *MRTO4* |
| 5907 | *MS* |
| 5908 | *MS4A1* |
| 5909 | *MS4A12* |
| 5910 | *MS4A5* |
| 5911 | *MSANTD3* |
| 5912 | *MSBP1* |
| 5913 | *MSC* |
| 5914 | *MSGN1* |
| 5915 | *MSH2* |
| 5916 | *MSH3* |
| 5917 | *MSH4* |
| 5918 | *MSH5* |
| 5919 | *MSH6* |
| 5920 | *MSI1* |
| 5921 | *MSI2* |
| 5922 | *MSL3* |
| 5923 | *MSLN* |
| 5924 | *MSMB* |
| 5925 | *MSN* |
| 5926 | *MSR1* |
| 5927 | *MSRB3* |
| 5928 | *MST1* |
| 5929 | *MST1R* |
| 5930 | *MSTN* |
| 5931 | *MSTO2P* |
| 5932 | *MSX1* |
| 5933 | *MSX2* |
| 5934 | *MT1A* |
| 5935 | *MT1F* |
| 5936 | *MT1G* |
| 5937 | *MT1X* |
| 5938 | *MT2A* |
| 5939 | *MT3* |
| 5940 | *MTA1* |
| 5941 | *MTA2* |
| 5942 | *MTA3* |
| 5943 | *MTAP* |
| 5944 | *MT-ATP6* |
| 5945 | *MTCH1* |
| 5946 | *MTCH2* |
| 5947 | *MT-CO1* |
| 5948 | *MT-CO2* |
| 5949 | *MTCO2P12* |
| 5950 | *MT-CO3* |
| 5951 | *MTCO3P1* |
| 5952 | *MT-CYB* |
| 5953 | *MTDH* |
| 5954 | *MTERF3* |
| 5955 | *MTERF4* |
| 5956 | *MTF1* |
| 5957 | *MTFMT* |
| 5958 | *MTFR2* |
| 5959 | *MTG1* |
| 5960 | *MTG2* |
| 5961 | *MTHFD1* |
| 5962 | *MTHFD1L* |
| 5963 | *MTHFD2* |
| 5964 | *MTHFD2P4* |
| 5965 | *MTHFR* |
| 5966 | *MTHFS* |
| 5967 | *MTHFSD* |
| 5968 | *MTM1* |
| 5969 | *MTMR10* |
| 5970 | *MTMR11* |
| 5971 | *MTMR14* |
| 5972 | *MTMR2* |
| 5973 | *MTMR3* |
| 5974 | *MTMR7* |
| 5975 | *MTMR8* |
| 5976 | *MT-ND1* |
| 5977 | *MT-ND2* |
| 5978 | *MT-ND3* |
| 5979 | *MT-ND4* |
| 5980 | *MT-ND4L* |
| 5981 | *MTND4P1* |
| 5982 | *MT-ND5* |
| 5983 | *MTND5P42* |
| 5984 | *MT-ND6* |
| 5985 | *MTNR1A* |
| 5986 | *MTNR1B* |
| 5987 | *MTO1* |
| 5988 | *MTOR* |
| 5989 | *MTPAP* |
| 5990 | *MTR* |
| 5991 | *MTRR* |
| 5992 | *MTSS1* |
| 5993 | *MTTP* |
| 5994 | *MTUS1* |
| 5995 | *MTUS2* |
| 5996 | *MTX1* |
| 5997 | *MUC1* |
| 5998 | *MUC12* |
| 5999 | *MUC13* |
| 6000 | *MUC15* |
| 6001 | *MUC16* |
| 6002 | *MUC17* |
| 6003 | *MUC2* |
| 6004 | *MUC20* |
| 6005 | *MUC21* |
| 6006 | *MUC22* |
| 6007 | *MUC3A* |
| 6008 | *MUC3B* |
| 6009 | *MUC4* |
| 6010 | *MUC5AC* |
| 6011 | *MUC5B* |
| 6012 | *MUC6* |
| 6013 | *MUC7* |
| 6014 | *MUCL1* |
| 6015 | *MUCL3* |
| 6016 | *MUL1* |
| 6017 | *MUS81* |
| 6018 | *MUTYH* |
| 6019 | *MVB12A* |
| 6020 | *MVD* |
| 6021 | *MVK* |
| 6022 | *MVP* |
| 6023 | *MX1* |
| 6024 | *MXD1* |
| 6025 | *MXD3* |
| 6026 | *MXD4* |
| 6027 | *MXI1* |
| 6028 | *MXRA5* |
| 6029 | *MXRA8* |
| 6030 | *MYB* |
| 6031 | *MYBBP1A* |
| 6032 | *MYBL1* |
| 6033 | *MYBL2* |
| 6034 | *MYBPC3* |
| 6035 | *MYC* |
| 6036 | *MYCBP* |
| 6037 | *MYCBP2* |
| 6038 | *MYCL* |
| 6039 | *MYCN* |
| 6040 | *MYD88* |
| 6041 | *MYDGF* |
| 6042 | *MYEOV* |
| 6043 | *MYH1* |
| 6044 | *MYH10* |
| 6045 | *MYH11* |
| 6046 | *MYH14* |
| 6047 | *MYH2* |
| 6048 | *MYH3* |
| 6049 | *MYH6* |
| 6050 | *MYH7* |
| 6051 | *MYH7B* |
| 6052 | *MYH9* |
| 6053 | *MYL1* |
| 6054 | *MYL10* |
| 6055 | *MYL11* |
| 6056 | *MYL12B* |
| 6057 | *MYL2* |
| 6058 | *MYL6* |
| 6059 | *MYL6B* |
| 6060 | *MYL9* |
| 6061 | *MYLK* |
| 6062 | *MYLK2* |
| 6063 | *MYLK3* |
| 6064 | *MYLKP1* |
| 6065 | *MYMX* |
| 6066 | *MYNN* |
| 6067 | *MYO10* |
| 6068 | *MYO15A* |
| 6069 | *MYO16* |
| 6070 | *MYO18A* |
| 6071 | *MYO18B* |
| 6072 | *MYO1A* |
| 6073 | *MYO1B* |
| 6074 | *MYO1C* |
| 6075 | *MYO1D* |
| 6076 | *MYO1E* |
| 6077 | *MYO1G* |
| 6078 | *MYO3A* |
| 6079 | *MYO5A* |
| 6080 | *MYO5B* |
| 6081 | *MYO5C* |
| 6082 | *MYO6* |
| 6083 | *MYO7A* |
| 6084 | *MYO9A* |
| 6085 | *MYO9B* |
| 6086 | *MYOD1* |
| 6087 | *MYOG* |
| 6088 | *MYOM2* |
| 6089 | *MYOT* |
| 6090 | *MYRF* |
| 6091 | *MYSM1* |
| 6092 | *MZB1* |
| 6093 | *MZF1* |
| 6094 | *MZF1-AS1* |
| 6095 | *MZT1* |
| 6096 | *MZT2B* |
| 6097 | *N4BP2* |
| 6098 | *N4BP2L1* |
| 6099 | *N6AMT1* |
| 6100 | *NAA10* |
| 6101 | *NAA15* |
| 6102 | *NAA20* |
| 6103 | *NAA25* |
| 6104 | *NAA40* |
| 6105 | *NAA60* |
| 6106 | *NAAA* |
| 6107 | *NAB2* |
| 6108 | *NABP1* |
| 6109 | *NABP2* |
| 6110 | *NACA* |
| 6111 | *NACAD* |
| 6112 | *NACC1* |
| 6113 | *NADK* |
| 6114 | *NAF1* |
| 6115 | *NAGLU* |
| 6116 | *NAMPT* |
| 6117 | *NANOG* |
| 6118 | *NANOGP1* |
| 6119 | *NANOGP8* |
| 6120 | *NANOS1* |
| 6121 | *NANP* |
| 6122 | *NANS* |
| 6123 | *NAP1L1* |
| 6124 | *NAP1L2* |
| 6125 | *NAP1L4* |
| 6126 | *NAPB* |
| 6127 | *NAPG* |
| 6128 | *NAPRT* |
| 6129 | *NAPSA* |
| 6130 | *NARF* |
| 6131 | *NARS1* |
| 6132 | *NASP* |
| 6133 | *NAT1* |
| 6134 | *NAT10* |
| 6135 | *NAT2* |
| 6136 | *NAT8B* |
| 6137 | *NATD1* |
| 6138 | *NAV2* |
| 6139 | *NAV3* |
| 6140 | *NAXE* |
| 6141 | *NBEAL2* |
| 6142 | *NBN* |
| 6143 | *NBPF1* |
| 6144 | *NBPF26* |
| 6145 | *NBPF4* |
| 6146 | *NBPF6* |
| 6147 | *NBR2* |
| 6148 | *NCAM1* |
| 6149 | *NCAM2* |
| 6150 | *NCAPD2* |
| 6151 | *NCAPG* |
| 6152 | *NCAPH* |
| 6153 | *NCBP1* |
| 6154 | *NCDN* |
| 6155 | *NCF2* |
| 6156 | *NCF4* |
| 6157 | *NCK1* |
| 6158 | *NCK2* |
| 6159 | *NCKAP1* |
| 6160 | *NCKIPSD* |
| 6161 | *NCL* |
| 6162 | *NCLN* |
| 6163 | *NCOA1* |
| 6164 | *NCOA2* |
| 6165 | *NCOA3* |
| 6166 | *NCOA4* |
| 6167 | *NCOA5* |
| 6168 | *NCOA6* |
| 6169 | *NCOR1* |
| 6170 | *NCOR2* |
| 6171 | *NCR1* |
| 6172 | *NCR2* |
| 6173 | *NCR3* |
| 6174 | *NCRUPAR* |
| 6175 | *NCSTN* |
| 6176 | *ND1* |
| 6177 | *ND2* |
| 6178 | *ND4L* |
| 6179 | *NDC80* |
| 6180 | *NDE1* |
| 6181 | *NDN* |
| 6182 | *NDOR1* |
| 6183 | *NDP* |
| 6184 | *NDRG1* |
| 6185 | *NDRG2* |
| 6186 | *NDRG3* |
| 6187 | *NDRG4* |
| 6188 | *NDST1* |
| 6189 | *NDST3* |
| 6190 | *NDST4* |
| 6191 | *NDUFA1* |
| 6192 | *NDUFA10* |
| 6193 | *NDUFA13* |
| 6194 | *NDUFA2* |
| 6195 | *NDUFA3* |
| 6196 | *NDUFA4* |
| 6197 | *NDUFA4L2* |
| 6198 | *NDUFA5* |
| 6199 | *NDUFA5P10* |
| 6200 | *NDUFA8* |
| 6201 | *NDUFA9* |
| 6202 | *NDUFAB1* |
| 6203 | *NDUFAF3* |
| 6204 | *NDUFAF4P3* |
| 6205 | *NDUFB2* |
| 6206 | *NDUFB9* |
| 6207 | *NDUFC2* |
| 6208 | *NDUFC2-KCTD14* |
| 6209 | *NDUFS1* |
| 6210 | *NDUFS2* |
| 6211 | *NDUFS3* |
| 6212 | *NDUFS7* |
| 6213 | *NEAT1* |
| 6214 | *NEB* |
| 6215 | *NEBL* |
| 6216 | *NECAB3* |
| 6217 | *NECTIN1* |
| 6218 | *NECTIN2* |
| 6219 | *NECTIN3* |
| 6220 | *NECTIN4* |
| 6221 | *NEDD4* |
| 6222 | *NEDD4L* |
| 6223 | *NEDD8* |
| 6224 | *NEDD9* |
| 6225 | *NEFH* |
| 6226 | *NEFM* |
| 6227 | *NEIL1* |
| 6228 | *NEIL2* |
| 6229 | *NEIL3* |
| 6230 | *NEK1* |
| 6231 | *NEK10* |
| 6232 | *NEK11* |
| 6233 | *NEK2* |
| 6234 | *NEK4* |
| 6235 | *NEK6* |
| 6236 | *NELFA* |
| 6237 | *NELFCD* |
| 6238 | *NELFE* |
| 6239 | *NELL1* |
| 6240 | *NELL2* |
| 6241 | *NEMF* |
| 6242 | *NEMP1* |
| 6243 | *NEO1* |
| 6244 | *NES* |
| 6245 | *NET1* |
| 6246 | *NETO2* |
| 6247 | *NEU1* |
| 6248 | *NEU3* |
| 6249 | *NEURL1* |
| 6250 | *NEURL4* |
| 6251 | *NEUROD1* |
| 6252 | *NEUROG1* |
| 6253 | *NF1* |
| 6254 | *NF2* |
| 6255 | *NFASC* |
| 6256 | *NFAT5* |
| 6257 | *NFATC1* |
| 6258 | *NFATC2* |
| 6259 | *NFATC3* |
| 6260 | *NFATC4* |
| 6261 | *NFE2L1* |
| 6262 | *NFE2L2* |
| 6263 | *NFE2L3* |
| 6264 | *NFIB* |
| 6265 | *NFIC* |
| 6266 | *NFIL3* |
| 6267 | *NFIX* |
| 6268 | *NFKB1* |
| 6269 | *NFKB2* |
| 6270 | *NFKBIA* |
| 6271 | *NFKBIZ* |
| 6272 | *NFS1* |
| 6273 | *NFX1* |
| 6274 | *NFXL1* |
| 6275 | *NFYA* |
| 6276 | *NFYB* |
| 6277 | *NFYC* |
| 6278 | *NGB* |
| 6279 | *NGEF* |
| 6280 | *NGF* |
| 6281 | *NGFR* |
| 6282 | *NGRN* |
| 6283 | *NHEJ1* |
| 6284 | *NHLRC2* |
| 6285 | *NHP2* |
| 6286 | *NHS* |
| 6287 | *NHSL1* |
| 6288 | *NID1* |
| 6289 | *NID2* |
| 6290 | *NIF3L1* |
| 6291 | *NIFK* |
| 6292 | *NIN* |
| 6293 | *NINJ2* |
| 6294 | *NINL* |
| 6295 | *NIP7* |
| 6296 | *NIPBL* |
| 6297 | *NIPSNAP1* |
| 6298 | *NIPSNAP2* |
| 6299 | *NISCH* |
| 6300 | *NIT1* |
| 6301 | *NIT2* |
| 6302 | *NKAIN2* |
| 6303 | *NKAP* |
| 6304 | *NKAPL* |
| 6305 | *NKD1* |
| 6306 | *NKD2* |
| 6307 | *NKIRAS1* |
| 6308 | *NKIRAS2* |
| 6309 | *NKRF* |
| 6310 | *NKX2-1* |
| 6311 | *NKX2-3* |
| 6312 | *NKX2-8* |
| 6313 | *NKX3-1* |
| 6314 | *NKX6-1* |
| 6315 | *NKX6-3* |
| 6316 | *NLE1* |
| 6317 | *NLGN1* |
| 6318 | *NLGN3* |
| 6319 | *NLGN4X* |
| 6320 | *NLK* |
| 6321 | *NLRC3* |
| 6322 | *NLRC4* |
| 6323 | *NLRC5* |
| 6324 | *NLRP1* |
| 6325 | *NLRP14* |
| 6326 | *NLRP2* |
| 6327 | *NLRP3* |
| 6328 | *NLRP6* |
| 6329 | *NLRP7* |
| 6330 | *NLRP8* |
| 6331 | *NLRP9* |
| 6332 | *NM* |
| 6333 | *NMB* |
| 6334 | *NMBR* |
| 6335 | *NME1* |
| 6336 | *NME1-NME2* |
| 6337 | *NME2* |
| 6338 | *NME2P1* |
| 6339 | *NME2P3* |
| 6340 | *NME3* |
| 6341 | *NME4* |
| 6342 | *NME6* |
| 6343 | *NME8* |
| 6344 | *NME9* |
| 6345 | *NMI* |
| 6346 | *NMNAT2* |
| 6347 | *NMRK1* |
| 6348 | *NMT1* |
| 6349 | *NMT2* |
| 6350 | *NMU* |
| 6351 | *NNMT* |
| 6352 | *NNT-AS1* |
| 6353 | *NOA1* |
| 6354 | *NOB1* |
| 6355 | *NOC2L* |
| 6356 | *NOC3L* |
| 6357 | *NOD1* |
| 6358 | *NOD2* |
| 6359 | *NODAL* |
| 6360 | *NOG* |
| 6361 | *NOL3* |
| 6362 | *NOL6* |
| 6363 | *NOLC1* |
| 6364 | *NOMO1* |
| 6365 | *NONO* |
| 6366 | *NOP10* |
| 6367 | *NOP14* |
| 6368 | *NOP16* |
| 6369 | *NOP2* |
| 6370 | *NOP56* |
| 6371 | *NOP56P3* |
| 6372 | *NOP58* |
| 6373 | *NOP9* |
| 6374 | *NORAD* |
| 6375 | *NOS1* |
| 6376 | *NOS2* |
| 6377 | *NOS3* |
| 6378 | *NOTCH1* |
| 6379 | *NOTCH2* |
| 6380 | *NOTCH3* |
| 6381 | *NOTCH4* |
| 6382 | *NOTUM* |
| 6383 | *NOVA1* |
| 6384 | *NOX1* |
| 6385 | *NOX4* |
| 6386 | *NOX5* |
| 6387 | *NOXA1* |
| 6388 | *NPAP1* |
| 6389 | *NPAS2* |
| 6390 | *NPAS3* |
| 6391 | *NPAT* |
| 6392 | *NPB* |
| 6393 | *NPBWR1* |
| 6394 | *NPBWR2* |
| 6395 | *NPC1* |
| 6396 | *NPC1L1* |
| 6397 | *NPEPL1* |
| 6398 | *NPEPPS* |
| 6399 | *NPHP1* |
| 6400 | *NPHS1* |
| 6401 | *NPHS2* |
| 6402 | *NPIPB5* |
| 6403 | *NPLOC4* |
| 6404 | *NPM1* |
| 6405 | *NPM1P28* |
| 6406 | *NPM1P5* |
| 6407 | *NPM3* |
| 6408 | *NPNT* |
| 6409 | *NPR1* |
| 6410 | *NPR3* |
| 6411 | *NPRL2* |
| 6412 | *NPTX1* |
| 6413 | *NPTX2* |
| 6414 | *NPW* |
| 6415 | *NPY* |
| 6416 | *NPY4R* |
| 6417 | *NPY4R2* |
| 6418 | *NQO1* |
| 6419 | *NQO2* |
| 6420 | *NR0B1* |
| 6421 | *NR0B2* |
| 6422 | *NR1H2* |
| 6423 | *NR1H3* |
| 6424 | *NR1H4* |
| 6425 | *NR1I2* |
| 6426 | *NR1I3* |
| 6427 | *NR2C2* |
| 6428 | *NR2E3* |
| 6429 | *NR2F2* |
| 6430 | *NR2F2-AS1* |
| 6431 | *NR2F6* |
| 6432 | *NR3C1* |
| 6433 | *NR3C2* |
| 6434 | *NR4A1* |
| 6435 | *NR4A1AS* |
| 6436 | *NR4A2* |
| 6437 | *NR5A2* |
| 6438 | *NRAS* |
| 6439 | *NRBP1* |
| 6440 | *NRCAM* |
| 6441 | *NRDC* |
| 6442 | *NRF1* |
| 6443 | *NRG1* |
| 6444 | *NRG2* |
| 6445 | *NRIP1* |
| 6446 | *NRIP2* |
| 6447 | *NRIP3* |
| 6448 | *NRK* |
| 6449 | *NRL* |
| 6450 | *NRM* |
| 6451 | *NRP1* |
| 6452 | *NRP2* |
| 6453 | *NRXN1* |
| 6454 | *NRXN3* |
| 6455 | *NSD1* |
| 6456 | *NSD2* |
| 6457 | *NSD3* |
| 6458 | *NSDHL* |
| 6459 | *NSF* |
| 6460 | *NSG1* |
| 6461 | *NSMAF* |
| 6462 | *NSMCE1* |
| 6463 | *NSMCE2* |
| 6464 | *NSRP1* |
| 6465 | *NSUN2* |
| 6466 | *NT5C* |
| 6467 | *NT5C2* |
| 6468 | *NT5C3B* |
| 6469 | *NT5DC2* |
| 6470 | *NT5E* |
| 6471 | *NTAQ1* |
| 6472 | *NTF3* |
| 6473 | *NTF4* |
| 6474 | *NTHL1* |
| 6475 | *NTMT1* |
| 6476 | *NTN1* |
| 6477 | *NTN3* |
| 6478 | *NTN4* |
| 6479 | *NTN5* |
| 6480 | *NTNG1* |
| 6481 | *NTPCR* |
| 6482 | *NTRK1* |
| 6483 | *NTRK2* |
| 6484 | *NTRK3* |
| 6485 | *NTS* |
| 6486 | *NTSR1* |
| 6487 | *NTSR2* |
| 6488 | *NTT* |
| 6489 | *NUAK1* |
| 6490 | *NUBP1* |
| 6491 | *NUBP2* |
| 6492 | *NUBPL* |
| 6493 | *NUCB1* |
| 6494 | *NUCB2* |
| 6495 | *NUCKS1* |
| 6496 | *NUDC* |
| 6497 | *NUDCD1* |
| 6498 | *NUDT1* |
| 6499 | *NUDT12* |
| 6500 | *NUDT16L1* |
| 6501 | *NUDT19* |
| 6502 | *NUDT21* |
| 6503 | *NUDT4B* |
| 6504 | *NUDT5* |
| 6505 | *NUDT6* |
| 6506 | *NUF2* |
| 6507 | *NUFIP2* |
| 6508 | *NUMA1* |
| 6509 | *NUMB* |
| 6510 | *NUMBL* |
| 6511 | *NUP107* |
| 6512 | *NUP133* |
| 6513 | *NUP153* |
| 6514 | *NUP160* |
| 6515 | *NUP188* |
| 6516 | *NUP210* |
| 6517 | *NUP214* |
| 6518 | *NUP50* |
| 6519 | *NUP62* |
| 6520 | *NUP85* |
| 6521 | *NUP88* |
| 6522 | *NUP93* |
| 6523 | *NUP98* |
| 6524 | *NUPR1* |
| 6525 | *NUSAP1* |
| 6526 | *NUTM1* |
| 6527 | *NUTM2D* |
| 6528 | *NWD1* |
| 6529 | *NXF1* |
| 6530 | *NXF2* |
| 6531 | *NXF2B* |
| 6532 | *NXN* |
| 6533 | *NXPE4* |
| 6534 | *NXPH4* |
| 6535 | *NXT1* |
| 6536 | *OARD1* |
| 6537 | *OAS2* |
| 6538 | *OAT* |
| 6539 | *OAZ2* |
| 6540 | *OBI1* |
| 6541 | *OBI1-AS1* |
| 6542 | *OBP2A* |
| 6543 | *OBSCN* |
| 6544 | *OCA2* |
| 6545 | *OCEL1* |
| 6546 | *OCIAD1* |
| 6547 | *OCLN* |
| 6548 | *OCM2* |
| 6549 | *ODAM* |
| 6550 | *ODC1* |
| 6551 | *ODF1* |
| 6552 | *ODF2* |
| 6553 | *ODF2L* |
| 6554 | *OGA* |
| 6555 | *OGDH* |
| 6556 | *OGDHL* |
| 6557 | *OGFR* |
| 6558 | *OGG1* |
| 6559 | *OGN* |
| 6560 | *OGT* |
| 6561 | *OIP5* |
| 6562 | *OIP5-AS1* |
| 6563 | *OLA1* |
| 6564 | *OLA1P2* |
| 6565 | *OLFM1* |
| 6566 | *OLFM2* |
| 6567 | *OLFM4* |
| 6568 | *OLFML3* |
| 6569 | *OLR1* |
| 6570 | *OMA1* |
| 6571 | *ONECUT1* |
| 6572 | *ONECUT2* |
| 6573 | *OPA1* |
| 6574 | *OPCML* |
| 6575 | *OPHN1* |
| 6576 | *OPN1LW* |
| 6577 | *OPN1MW* |
| 6578 | *OPN1MW2* |
| 6579 | *OPN1MW3* |
| 6580 | *OPN3* |
| 6581 | *OPRD1* |
| 6582 | *OPRM1* |
| 6583 | *OR10AH1P* |
| 6584 | *OR12D3* |
| 6585 | *OR1E2* |
| 6586 | *OR1J2* |
| 6587 | *OR1N1* |
| 6588 | *OR2H2* |
| 6589 | *OR4A5* |
| 6590 | *OR51V1* |
| 6591 | *OR7C1* |
| 6592 | *ORAI1* |
| 6593 | *ORAI3* |
| 6594 | *ORC3* |
| 6595 | *ORC6* |
| 6596 | *ORM1* |
| 6597 | *ORM2* |
| 6598 | *ORMDL1* |
| 6599 | *OSBP* |
| 6600 | *OSBP2* |
| 6601 | *OSBPL11* |
| 6602 | *OSBPL3* |
| 6603 | *OSBPL7* |
| 6604 | *OSBPL8* |
| 6605 | *OSER1* |
| 6606 | *OSGIN2* |
| 6607 | *OSM* |
| 6608 | *OSMR* |
| 6609 | *OSR2* |
| 6610 | *OSTC* |
| 6611 | *OSTF1* |
| 6612 | *OTC* |
| 6613 | *OTOF* |
| 6614 | *OTOGL* |
| 6615 | *OTOP2* |
| 6616 | *OTUB1* |
| 6617 | *OTUB2* |
| 6618 | *OTUD1* |
| 6619 | *OTUD3* |
| 6620 | *OTUD4* |
| 6621 | *OTUD5* |
| 6622 | *OTUD6A* |
| 6623 | *OTUD7A* |
| 6624 | *OTUD7B* |
| 6625 | *OTX1* |
| 6626 | *OVGP1* |
| 6627 | *OVOL2* |
| 6628 | *OXA1L* |
| 6629 | *OXER1* |
| 6630 | *OXSM* |
| 6631 | *P2RX5* |
| 6632 | *P2RX7* |
| 6633 | *P2RY13* |
| 6634 | *P2RY14* |
| 6635 | *P2RY2* |
| 6636 | *P2RY6* |
| 6637 | *P3H2* |
| 6638 | *P3H3* |
| 6639 | *P3H4* |
| 6640 | *P4HA1* |
| 6641 | *P4HB* |
| 6642 | *P4HTM* |
| 6643 | *PA2G4* |
| 6644 | *PAAF1* |
| 6645 | *PABIR2* |
| 6646 | *PABPC1* |
| 6647 | *PABPC1L* |
| 6648 | *PABPC1L2A* |
| 6649 | *PABPC3* |
| 6650 | *PABPC4* |
| 6651 | *PABPC4L* |
| 6652 | *PABPN1* |
| 6653 | *PACC1* |
| 6654 | *PACRGL* |
| 6655 | *PACS2* |
| 6656 | *PADI2* |
| 6657 | *PADI3* |
| 6658 | *PADI4* |
| 6659 | *PAEP* |
| 6660 | *PAF1* |
| 6661 | *PAFAH1B1* |
| 6662 | *PAFAH1B2* |
| 6663 | *PAFAH1B3* |
| 6664 | *PAG1* |
| 6665 | *PAGE2* |
| 6666 | *PAGE4* |
| 6667 | *PAH* |
| 6668 | *PAICS* |
| 6669 | *PAIP1* |
| 6670 | *PAIP1P1* |
| 6671 | *PAIP2* |
| 6672 | *PAIP2B* |
| 6673 | *PAK1* |
| 6674 | *PAK2* |
| 6675 | *PAK3* |
| 6676 | *PAK4* |
| 6677 | *PAK5* |
| 6678 | *PAK6* |
| 6679 | *PALB2* |
| 6680 | *PALD1* |
| 6681 | *PALLD* |
| 6682 | *PALM2AKAP2* |
| 6683 | *PALS1* |
| 6684 | *PAM* |
| 6685 | *PAMR1* |
| 6686 | *PAN2* |
| 6687 | *PANDAR* |
| 6688 | *PANK1* |
| 6689 | *PANK2* |
| 6690 | *PANK4* |
| 6691 | *PANTR1* |
| 6692 | *PANX1* |
| 6693 | *PANX2* |
| 6694 | *PAOX* |
| 6695 | *PAPOLA* |
| 6696 | *PAPPA* |
| 6697 | *PAPSS1* |
| 6698 | *PAPSS2* |
| 6699 | *PAQR3* |
| 6700 | *PAQR6* |
| 6701 | *PARD3* |
| 6702 | *PARD3B* |
| 6703 | *PARD6B* |
| 6704 | *PARD6G* |
| 6705 | *PARK7* |
| 6706 | *PARM1* |
| 6707 | *PARP1* |
| 6708 | *PARP10* |
| 6709 | *PARP11* |
| 6710 | *PARP12* |
| 6711 | *PARP14* |
| 6712 | *PARP2* |
| 6713 | *PARP4* |
| 6714 | *PARP6* |
| 6715 | *PARP8* |
| 6716 | *PARS2* |
| 6717 | *PART1* |
| 6718 | *PARVA* |
| 6719 | *PARVB* |
| 6720 | *PARVG* |
| 6721 | *PASD1* |
| 6722 | *PATZ1* |
| 6723 | *PAWR* |
| 6724 | *PAX2* |
| 6725 | *PAX3* |
| 6726 | *PAX4* |
| 6727 | *PAX5* |
| 6728 | *PAX6* |
| 6729 | *PAX6DRR* |
| 6730 | *PAX8* |
| 6731 | *PAX9* |
| 6732 | *PBK* |
| 6733 | *PBRM1* |
| 6734 | *PBX3* |
| 6735 | *PBX4* |
| 6736 | *PC* |
| 6737 | *PCAT1* |
| 6738 | *PCAT6* |
| 6739 | *PCBD1* |
| 6740 | *PCBD2* |
| 6741 | *PCBP1* |
| 6742 | *PCBP2* |
| 6743 | *PCBP3* |
| 6744 | *PCBP4* |
| 6745 | *PCCA* |
| 6746 | *PCCB* |
| 6747 | *PCDH10* |
| 6748 | *PCDH11X* |
| 6749 | *PCDH15* |
| 6750 | *PCDH17* |
| 6751 | *PCDH18* |
| 6752 | *PCDH20* |
| 6753 | *PCDH7* |
| 6754 | *PCDH8* |
| 6755 | *PCDHA@* |
| 6756 | *PCDHB15* |
| 6757 | *PCDHB3* |
| 6758 | *PCDHG@* |
| 6759 | *PCDHGA7* |
| 6760 | *PCDHGC3* |
| 6761 | *PCED1B* |
| 6762 | *PCGF1* |
| 6763 | *PCGF2* |
| 6764 | *PCID2* |
| 6765 | *PCK1* |
| 6766 | *PCK2* |
| 6767 | *PCLAF* |
| 6768 | *PCLO* |
| 6769 | *PCM1* |
| 6770 | *PCNA* |
| 6771 | *PCNAP1* |
| 6772 | *PCNPP1* |
| 6773 | *PCNT* |
| 6774 | *PCNX1* |
| 6775 | *PCNX2* |
| 6776 | *PCNX3* |
| 6777 | *PCOLCE* |
| 6778 | *PCSK1* |
| 6779 | *PCSK2* |
| 6780 | *PCSK5* |
| 6781 | *PCSK6* |
| 6782 | *PCSK7* |
| 6783 | *PCSK9* |
| 6784 | *PCTP* |
| 6785 | *PCYT1A* |
| 6786 | *PDAP1* |
| 6787 | *PDC* |
| 6788 | *PDCD1* |
| 6789 | *PDCD10* |
| 6790 | *PDCD11* |
| 6791 | *PDCD1LG2* |
| 6792 | *PDCD2L* |
| 6793 | *PDCD4* |
| 6794 | *PDCD5* |
| 6795 | *PDCD6* |
| 6796 | *PDCD6IP* |
| 6797 | *PDCL3* |
| 6798 | *PDCL3P4* |
| 6799 | *PDE10A* |
| 6800 | *PDE1C* |
| 6801 | *PDE2A* |
| 6802 | *PDE4A* |
| 6803 | *PDE4B* |
| 6804 | *PDE4D* |
| 6805 | *PDE4DIP* |
| 6806 | *PDE5A* |
| 6807 | *PDE6B* |
| 6808 | *PDE7A* |
| 6809 | *PDE9A* |
| 6810 | *PDGFA* |
| 6811 | *PDGFB* |
| 6812 | *PDGFC* |
| 6813 | *PDGFD* |
| 6814 | *PDGFRA* |
| 6815 | *PDGFRB* |
| 6816 | *PDGFRL* |
| 6817 | *PDHA1* |
| 6818 | *PDHB* |
| 6819 | *PDHX* |
| 6820 | *PDIA2* |
| 6821 | *PDIA3* |
| 6822 | *PDIA6* |
| 6823 | *PDIK1L* |
| 6824 | *PDILT* |
| 6825 | *PDK1* |
| 6826 | *PDK3* |
| 6827 | *PDK4* |
| 6828 | *PDLIM1* |
| 6829 | *PDLIM2* |
| 6830 | *PDLIM5* |
| 6831 | *PDLIM7* |
| 6832 | *PDP1* |
| 6833 | *PDPK1* |
| 6834 | *PDPN* |
| 6835 | *PDRG1* |
| 6836 | *PDS5A* |
| 6837 | *PDS5B* |
| 6838 | *PDSS1* |
| 6839 | *PDX1* |
| 6840 | *PDXDC1* |
| 6841 | *PDXP* |
| 6842 | *PDZD2* |
| 6843 | *PDZD4* |
| 6844 | *PDZK1* |
| 6845 | *PDZK1IP1* |
| 6846 | *PDZRN3* |
| 6847 | *PDZRN4* |
| 6848 | *PEA15* |
| 6849 | *PEAK1* |
| 6850 | *PEBP1* |
| 6851 | *PEBP4* |
| 6852 | *PECAM1* |
| 6853 | *PEDS1* |
| 6854 | *PEDS1-UBE2V1* |
| 6855 | *PEG10* |
| 6856 | *PEG3* |
| 6857 | *PELI1* |
| 6858 | *PELO* |
| 6859 | *PELP1* |
| 6860 | *PENK* |
| 6861 | *PEPD* |
| 6862 | *PER1* |
| 6863 | *PER2* |
| 6864 | *PER3* |
| 6865 | *PERCC1* |
| 6866 | *PES1* |
| 6867 | *PEX1* |
| 6868 | *PEX10* |
| 6869 | *PEX12* |
| 6870 | *PEX5* |
| 6871 | *PEX5L* |
| 6872 | *PEX6* |
| 6873 | *PF4* |
| 6874 | *PFAS* |
| 6875 | *PFDN1* |
| 6876 | *PFDN2* |
| 6877 | *PFDN4* |
| 6878 | *PFDN5* |
| 6879 | *PFDN6* |
| 6880 | *PFKFB2* |
| 6881 | *PFKFB3* |
| 6882 | *PFKFB4* |
| 6883 | *PFKL* |
| 6884 | *PFKM* |
| 6885 | *PFKP* |
| 6886 | *PFN1* |
| 6887 | *PFN2* |
| 6888 | *PGA3* |
| 6889 | *PGA4* |
| 6890 | *PGA5* |
| 6891 | *PGAM1* |
| 6892 | *PGAM1P5* |
| 6893 | *PGAM2* |
| 6894 | *PGAM5* |
| 6895 | *PGAP6* |
| 6896 | *PGBD3* |
| 6897 | *PGC* |
| 6898 | *PGD* |
| 6899 | *PGF* |
| 6900 | *PGGT1B* |
| 6901 | *PGK1* |
| 6902 | *PGM1* |
| 6903 | *PGM3* |
| 6904 | *PGM5* |
| 6905 | *PGM5-AS1* |
| 6906 | *PGP* |
| 6907 | *PGPEP1* |
| 6908 | *PGR* |
| 6909 | *PGS1* |
| 6910 | *PHACTR1* |
| 6911 | *PHACTR2* |
| 6912 | *PHACTR3* |
| 6913 | *PHACTR4* |
| 6914 | *PHAX* |
| 6915 | *PHB* |
| 6916 | *PHB1* |
| 6917 | *PHB1P9* |
| 6918 | *PHB2* |
| 6919 | *PHB2P1* |
| 6920 | *PHC1* |
| 6921 | *PHC2* |
| 6922 | *PHC3* |
| 6923 | *PHETA1* |
| 6924 | *PHEX* |
| 6925 | *PHF1* |
| 6926 | *PHF14* |
| 6927 | *PHF2* |
| 6928 | *PHF20* |
| 6929 | *PHF20L1* |
| 6930 | *PHF21A* |
| 6931 | *PHF5A* |
| 6932 | *PHF6* |
| 6933 | *PHF7* |
| 6934 | *PHF8* |
| 6935 | *PHGDH* |
| 6936 | *PHGR1* |
| 6937 | *PHIP* |
| 6938 | *PHKA2* |
| 6939 | *PHKB* |
| 6940 | *PHKG1* |
| 6941 | *PHLDA1* |
| 6942 | *PHLDA2* |
| 6943 | *PHLDA3* |
| 6944 | *PHLDB1* |
| 6945 | *PHLDB2* |
| 6946 | *PHLDB3* |
| 6947 | *PHLPP1* |
| 6948 | *PHLPP2* |
| 6949 | *PHOX2B* |
| 6950 | *PHPT1* |
| 6951 | *PHRF1* |
| 6952 | *PHYHD1* |
| 6953 | *PI3* |
| 6954 | *PI4K2A* |
| 6955 | *PI4KB* |
| 6956 | *PIAS1* |
| 6957 | *PIAS3* |
| 6958 | *PICALM* |
| 6959 | *PICK1* |
| 6960 | *PIEZO1* |
| 6961 | *PIF1* |
| 6962 | *PIGB* |
| 6963 | *PIGF* |
| 6964 | *PIGK* |
| 6965 | *PIGN* |
| 6966 | *PIGO* |
| 6967 | *PIGP* |
| 6968 | *PIGR* |
| 6969 | *PIGS* |
| 6970 | *PIGU* |
| 6971 | *PIH1D1* |
| 6972 | *PIK3AP1* |
| 6973 | *PIK3C2A* |
| 6974 | *PIK3C2B* |
| 6975 | *PIK3C2G* |
| 6976 | *PIK3C3* |
| 6977 | *PIK3CA* |
| 6978 | *PIK3CB* |
| 6979 | *PIK3CD* |
| 6980 | *PIK3CG* |
| 6981 | *PIK3R1* |
| 6982 | *PIK3R2* |
| 6983 | *PIK3R3* |
| 6984 | *PIK3R4* |
| 6985 | *PIK3R5* |
| 6986 | *PIM1* |
| 6987 | *PIM2* |
| 6988 | *PIM3* |
| 6989 | *PIMREG* |
| 6990 | *PIN1* |
| 6991 | *PIN4* |
| 6992 | *PINK1* |
| 6993 | *PINX1* |
| 6994 | *PIP* |
| 6995 | *PIP4K2A* |
| 6996 | *PIP4K2B* |
| 6997 | *PIP5K1C* |
| 6998 | *PIPOX* |
| 6999 | *PIR* |
| 7000 | *PIRT* |
| 7001 | *PISD* |
| 7002 | *PITPNA* |
| 7003 | *PITPNB* |
| 7004 | *PITPNM1* |
| 7005 | *PITPNM3* |
| 7006 | *PITX1* |
| 7007 | *PITX2* |
| 7008 | *PIWIL1* |
| 7009 | *PIWIL2* |
| 7010 | *PIWIL4* |
| 7011 | *PJA2* |
| 7012 | *PKD1* |
| 7013 | *PKD1L1* |
| 7014 | *PKD1L2* |
| 7015 | *PKD2* |
| 7016 | *PKD2L1* |
| 7017 | *PKDREJ* |
| 7018 | *PKHD1* |
| 7019 | *PKLR* |
| 7020 | *PKM* |
| 7021 | *PKMYT1* |
| 7022 | *PKN1* |
| 7023 | *PKNOX1* |
| 7024 | *PKP1* |
| 7025 | *PKP2* |
| 7026 | *PKP3* |
| 7027 | *PKP4* |
| 7028 | *PLA1A* |
| 7029 | *PLA2G10* |
| 7030 | *PLA2G12B* |
| 7031 | *PLA2G15* |
| 7032 | *PLA2G1B* |
| 7033 | *PLA2G2A* |
| 7034 | *PLA2G2D* |
| 7035 | *PLA2G2E* |
| 7036 | *PLA2G3* |
| 7037 | *PLA2G4A* |
| 7038 | *PLA2G4C* |
| 7039 | *PLA2G6* |
| 7040 | *PLA2G7* |
| 7041 | *PLA2R1* |
| 7042 | *PLAAT3* |
| 7043 | *PLAAT4* |
| 7044 | *PLAC1* |
| 7045 | *PLAC8* |
| 7046 | *PLAG1* |
| 7047 | *PLAGL1* |
| 7048 | *PLAGL2* |
| 7049 | *PLAT* |
| 7050 | *PLAU* |
| 7051 | *PLAUR* |
| 7052 | *PLB1* |
| 7053 | *PLBD2* |
| 7054 | *PLCB1* |
| 7055 | *PLCB3* |
| 7056 | *PLCB4* |
| 7057 | *PLCD1* |
| 7058 | *PLCD4* |
| 7059 | *PLCE1* |
| 7060 | *PLCG1* |
| 7061 | *PLCG2* |
| 7062 | *PLCH1* |
| 7063 | *PLCL1* |
| 7064 | *PLD1* |
| 7065 | *PLD2* |
| 7066 | *PLEC* |
| 7067 | *PLEK* |
| 7068 | *PLEK2* |
| 7069 | *PLEKHA7* |
| 7070 | *PLEKHA8* |
| 7071 | *PLEKHA8P1* |
| 7072 | *PLEKHG2* |
| 7073 | *PLEKHG4* |
| 7074 | *PLEKHG5* |
| 7075 | *PLEKHG6* |
| 7076 | *PLEKHM1* |
| 7077 | *PLEKHO2* |
| 7078 | *PLG* |
| 7079 | *PLIN2* |
| 7080 | *PLIN3* |
| 7081 | *PLK1* |
| 7082 | *PLK2* |
| 7083 | *PLK3* |
| 7084 | *PLK4* |
| 7085 | *PLOD1* |
| 7086 | *PLOD2* |
| 7087 | *PLOD3* |
| 7088 | *PLP2* |
| 7089 | *PLPBP* |
| 7090 | *PLPPR2* |
| 7091 | *PLRG1* |
| 7092 | *PLS1* |
| 7093 | *PLS3* |
| 7094 | *PLSCR1* |
| 7095 | *PLSCR4* |
| 7096 | *PLXDC1* |
| 7097 | *PLXDC2* |
| 7098 | *PLXNA1* |
| 7099 | *PLXNA2* |
| 7100 | *PLXNB1* |
| 7101 | *PLXNB2* |
| 7102 | *PLXND1* |
| 7103 | *PMAIP1* |
| 7104 | *PMEL* |
| 7105 | *PMEPA1* |
| 7106 | *PML* |
| 7107 | *PMM2* |
| 7108 | *PMP2* |
| 7109 | *PMP22* |
| 7110 | *PMS1* |
| 7111 | *PMS2* |
| 7112 | *PMS2CL* |
| 7113 | *PMS2P1* |
| 7114 | *PMS2P10* |
| 7115 | *PMS2P11* |
| 7116 | *PMS2P2* |
| 7117 | *PMS2P3* |
| 7118 | *PMS2P4* |
| 7119 | *PMS2P5* |
| 7120 | *PMS2P6* |
| 7121 | *PMS2P7* |
| 7122 | *PMS2P8* |
| 7123 | *PMS2P9* |
| 7124 | *PNKD* |
| 7125 | *PNLIPRP1* |
| 7126 | *PNMA8A* |
| 7127 | *PNMA8C* |
| 7128 | *PNMT* |
| 7129 | *PNN* |
| 7130 | *PNO1* |
| 7131 | *PNP* |
| 7132 | *PNPLA2* |
| 7133 | *PNPLA6* |
| 7134 | *PNPLA8* |
| 7135 | *PNPT1* |
| 7136 | *POC1B-GALNT4* |
| 7137 | *PODXL* |
| 7138 | *POF1B* |
| 7139 | *POFUT1* |
| 7140 | *POGLUT1* |
| 7141 | *POGLUT2* |
| 7142 | *POLA1* |
| 7143 | *POLA2* |
| 7144 | *POLB* |
| 7145 | *POLD1* |
| 7146 | *POLD3* |
| 7147 | *POLDIP2* |
| 7148 | *POLDIP3* |
| 7149 | *POLE* |
| 7150 | *POLE2* |
| 7151 | *POLG* |
| 7152 | *POLH* |
| 7153 | *POLI* |
| 7154 | *POLK* |
| 7155 | *POLL* |
| 7156 | *POLQ* |
| 7157 | *POLR1B* |
| 7158 | *POLR1D* |
| 7159 | *POLR1G* |
| 7160 | *POLR1H* |
| 7161 | *POLR1HASP* |
| 7162 | *POLR2A* |
| 7163 | *POLR2B* |
| 7164 | *POLR2C* |
| 7165 | *POLR2F* |
| 7166 | *POLR2G* |
| 7167 | *POLR2J2* |
| 7168 | *POLR2J3* |
| 7169 | *POLR2J4* |
| 7170 | *POLR2K* |
| 7171 | *POLR2L* |
| 7172 | *POLR3A* |
| 7173 | *POLR3G* |
| 7174 | *POLR3K* |
| 7175 | *POLRMT* |
| 7176 | *POM121* |
| 7177 | *POMC* |
| 7178 | *POMGNT1* |
| 7179 | *POMGNT2* |
| 7180 | *POMP* |
| 7181 | *POMT1* |
| 7182 | *POMT2* |
| 7183 | *PON1* |
| 7184 | *PON2* |
| 7185 | *PON3* |
| 7186 | *POP1* |
| 7187 | *POP4* |
| 7188 | *POP7* |
| 7189 | *POR* |
| 7190 | *PORCN* |
| 7191 | *POSTN* |
| 7192 | *POT1* |
| 7193 | *POTEA* |
| 7194 | *POTED* |
| 7195 | *POTEE* |
| 7196 | *POTEF* |
| 7197 | *POU2AF1* |
| 7198 | *POU2AF2* |
| 7199 | *POU2AF3* |
| 7200 | *POU2F1* |
| 7201 | *POU2F2* |
| 7202 | *POU3F3* |
| 7203 | *POU5F1* |
| 7204 | *POU5F1B* |
| 7205 | *POU5F1P3* |
| 7206 | *POU5F1P4* |
| 7207 | *POU6F1* |
| 7208 | *POU6F2-AS2* |
| 7209 | *PPA1* |
| 7210 | *PPA2* |
| 7211 | *PPARA* |
| 7212 | *PPARD* |
| 7213 | *PPARG* |
| 7214 | *PPARGC1A* |
| 7215 | *PPARGC1B* |
| 7216 | *PPAT* |
| 7217 | *PPBP* |
| 7218 | *PPFIA1* |
| 7219 | *PPFIBP1* |
| 7220 | *PPHLN1* |
| 7221 | *PPIA* |
| 7222 | *PPIAP33* |
| 7223 | *PPIAP43* |
| 7224 | *PPIAP44* |
| 7225 | *PPIAP45* |
| 7226 | *PPIB* |
| 7227 | *PPID* |
| 7228 | *PPIF* |
| 7229 | *PPIG* |
| 7230 | *PPIL1* |
| 7231 | *PPIP5K2* |
| 7232 | *PPL* |
| 7233 | *PPM1A* |
| 7234 | *PPM1B* |
| 7235 | *PPM1D* |
| 7236 | *PPM1E* |
| 7237 | *PPM1F* |
| 7238 | *PPM1G* |
| 7239 | *PPM1H* |
| 7240 | *PPM1L* |
| 7241 | *PPM1M* |
| 7242 | *PPOX* |
| 7243 | *PPP1CA* |
| 7244 | *PPP1CB* |
| 7245 | *PPP1CC* |
| 7246 | *PPP1R10* |
| 7247 | *PPP1R11* |
| 7248 | *PPP1R12A* |
| 7249 | *PPP1R12B* |
| 7250 | *PPP1R12C* |
| 7251 | *PPP1R13B* |
| 7252 | *PPP1R13L* |
| 7253 | *PPP1R14A* |
| 7254 | *PPP1R15A* |
| 7255 | *PPP1R1A* |
| 7256 | *PPP1R1B* |
| 7257 | *PPP1R21* |
| 7258 | *PPP1R2C* |
| 7259 | *PPP1R3A* |
| 7260 | *PPP1R3C* |
| 7261 | *PPP1R3D* |
| 7262 | *PPP1R3F* |
| 7263 | *PPP1R8* |
| 7264 | *PPP1R9B* |
| 7265 | *PPP2CA* |
| 7266 | *PPP2CB* |
| 7267 | *PPP2R1A* |
| 7268 | *PPP2R1B* |
| 7269 | *PPP2R2A* |
| 7270 | *PPP2R2B* |
| 7271 | *PPP2R2D* |
| 7272 | *PPP2R3C* |
| 7273 | *PPP2R5C* |
| 7274 | *PPP2R5E* |
| 7275 | *PPP3CA* |
| 7276 | *PPP3CB* |
| 7277 | *PPP3R2* |
| 7278 | *PPP4C* |
| 7279 | *PPP4R1* |
| 7280 | *PPP4R3A* |
| 7281 | *PPP5C* |
| 7282 | *PPP6C* |
| 7283 | *PPP6R1* |
| 7284 | *PPP6R2* |
| 7285 | *PPP6R3* |
| 7286 | *PPRC1* |
| 7287 | *PPT1* |
| 7288 | *PPY* |
| 7289 | *PQBP1* |
| 7290 | *PRAC2* |
| 7291 | *PRAG1* |
| 7292 | *PRC1* |
| 7293 | *PRCP* |
| 7294 | *PRDM1* |
| 7295 | *PRDM14* |
| 7296 | *PRDM15* |
| 7297 | *PRDM2* |
| 7298 | *PRDM4* |
| 7299 | *PRDM5* |
| 7300 | *PRDX1* |
| 7301 | *PRDX2* |
| 7302 | *PRDX3* |
| 7303 | *PRDX4* |
| 7304 | *PRDX5* |
| 7305 | *PRDX6* |
| 7306 | *PRELP* |
| 7307 | *PREP* |
| 7308 | *PREX1* |
| 7309 | *PREX2* |
| 7310 | *PRF1* |
| 7311 | *PRG2* |
| 7312 | *PRG3* |
| 7313 | *PRG4* |
| 7314 | *PRH2* |
| 7315 | *PRICKLE1* |
| 7316 | *PRICKLE2* |
| 7317 | *PRICKLE3* |
| 7318 | *PRIM1* |
| 7319 | *PRIM2* |
| 7320 | *PRIMA1* |
| 7321 | *PRKAA1* |
| 7322 | *PRKAA2* |
| 7323 | *PRKAB1* |
| 7324 | *PRKACA* |
| 7325 | *PRKACB* |
| 7326 | *PRKACG* |
| 7327 | *PRKAG1* |
| 7328 | *PRKAG2* |
| 7329 | *PRKAR1A* |
| 7330 | *PRKAR1B* |
| 7331 | *PRKAR2A* |
| 7332 | *PRKAR2B* |
| 7333 | *PRKCA* |
| 7334 | *PRKCB* |
| 7335 | *PRKCD* |
| 7336 | *PRKCE* |
| 7337 | *PRKCG* |
| 7338 | *PRKCH* |
| 7339 | *PRKCI* |
| 7340 | *PRKCQ* |
| 7341 | *PRKCQ-AS1* |
| 7342 | *PRKCZ* |
| 7343 | *PRKD1* |
| 7344 | *PRKD2* |
| 7345 | *PRKDC* |
| 7346 | *PRKG1* |
| 7347 | *PRKG2* |
| 7348 | *PRKN* |
| 7349 | *PRKRA* |
| 7350 | *PRKRIP1* |
| 7351 | *PRL* |
| 7352 | *PRLHR* |
| 7353 | *PRLR* |
| 7354 | *PRMT1* |
| 7355 | *PRMT3* |
| 7356 | *PRMT5* |
| 7357 | *PRMT6* |
| 7358 | *PRMT7* |
| 7359 | *PRNCR1* |
| 7360 | *PRNP* |
| 7361 | *PROCR* |
| 7362 | *PRODH* |
| 7363 | *PROK1* |
| 7364 | *PROK2* |
| 7365 | *PROKR2* |
| 7366 | *PROM1* |
| 7367 | *PROM2* |
| 7368 | *PROS1* |
| 7369 | *PROSER2* |
| 7370 | *PROX1* |
| 7371 | *PROZ* |
| 7372 | *PRPF19* |
| 7373 | *PRPF31* |
| 7374 | *PRPF4* |
| 7375 | *PRPF4B* |
| 7376 | *PRPF6* |
| 7377 | *PRPF8* |
| 7378 | *PRPH* |
| 7379 | *PRPS1* |
| 7380 | *PRPS2* |
| 7381 | *PRPSAP1* |
| 7382 | *PRPSAP2* |
| 7383 | *PRR11* |
| 7384 | *PRR13* |
| 7385 | *PRR15* |
| 7386 | *PRR18* |
| 7387 | *PRR5* |
| 7388 | *PRR5-ARHGAP8* |
| 7389 | *PRR5L* |
| 7390 | *PRRC2A* |
| 7391 | *PRRC2C* |
| 7392 | *PRRG1* |
| 7393 | *PRRG4* |
| 7394 | *PRRT1* |
| 7395 | *PRRT2* |
| 7396 | *PRRX1* |
| 7397 | *PRRX2* |
| 7398 | *PRS* |
| 7399 | *PRSS1* |
| 7400 | *PRSS2* |
| 7401 | *PRSS3* |
| 7402 | *PRSS3P2* |
| 7403 | *PRSS50* |
| 7404 | *PRSS55* |
| 7405 | *PRSS58* |
| 7406 | *PRSS8* |
| 7407 | *PRTN3* |
| 7408 | *PRUNE1* |
| 7409 | *PRUNE2* |
| 7410 | *PSAP* |
| 7411 | *PSAT1* |
| 7412 | *PSC* |
| 7413 | *PSCA* |
| 7414 | *PSD* |
| 7415 | *PSD4* |
| 7416 | *PSEN1* |
| 7417 | *PSEN2* |
| 7418 | *PSENEN* |
| 7419 | *PSG2* |
| 7420 | *PSG9* |
| 7421 | *PSMA1* |
| 7422 | *PSMA2* |
| 7423 | *PSMA3* |
| 7424 | *PSMA5* |
| 7425 | *PSMA6* |
| 7426 | *PSMA7* |
| 7427 | *PSMA8* |
| 7428 | *PSMB6* |
| 7429 | *PSMB7* |
| 7430 | *PSMB8* |
| 7431 | *PSMB9* |
| 7432 | *PSMC1* |
| 7433 | *PSMC1P8* |
| 7434 | *PSMC2* |
| 7435 | *PSMC3* |
| 7436 | *PSMC3IP* |
| 7437 | *PSMC4* |
| 7438 | *PSMC5* |
| 7439 | *PSMC6* |
| 7440 | *PSMD10* |
| 7441 | *PSMD11* |
| 7442 | *PSMD13* |
| 7443 | *PSMD14* |
| 7444 | *PSMD2* |
| 7445 | *PSMD3* |
| 7446 | *PSMD4* |
| 7447 | *PSMD5* |
| 7448 | *PSMD7* |
| 7449 | *PSMD8* |
| 7450 | *PSMD9* |
| 7451 | *PSME1* |
| 7452 | *PSME2* |
| 7453 | *PSME3* |
| 7454 | *PSMF1* |
| 7455 | *PSMG1* |
| 7456 | *PSMG2* |
| 7457 | *PSPC1* |
| 7458 | *PSPH* |
| 7459 | *PSRC1* |
| 7460 | *PSTPIP1* |
| 7461 | *PSTPIP2* |
| 7462 | *PTAFR* |
| 7463 | *PTBP1* |
| 7464 | *PTBP2* |
| 7465 | *PTBP3* |
| 7466 | *PTCD1* |
| 7467 | *PTCD2* |
| 7468 | *PTCD3* |
| 7469 | *PTCH1* |
| 7470 | *PTCHD4* |
| 7471 | *PTDSS1* |
| 7472 | *PTEN* |
| 7473 | *PTENP1* |
| 7474 | *PTGDR* |
| 7475 | *PTGDR2* |
| 7476 | *PTGDS* |
| 7477 | *PTGER1* |
| 7478 | *PTGER2* |
| 7479 | *PTGER3* |
| 7480 | *PTGER4* |
| 7481 | *PTGES* |
| 7482 | *PTGES2* |
| 7483 | *PTGES3* |
| 7484 | *PTGES3L-AARSD1* |
| 7485 | *PTGFR* |
| 7486 | *PTGIR* |
| 7487 | *PTGIS* |
| 7488 | *PTGS1* |
| 7489 | *PTGS2* |
| 7490 | *PTH* |
| 7491 | *PTHLH* |
| 7492 | *PTK2* |
| 7493 | *PTK2B* |
| 7494 | *PTK6* |
| 7495 | *PTK7* |
| 7496 | *PTMA* |
| 7497 | *PTN* |
| 7498 | *PTOV1* |
| 7499 | *PTP4A1* |
| 7500 | *PTP4A2* |
| 7501 | *PTP4A3* |
| 7502 | *PTPA* |
| 7503 | *PTPDC1* |
| 7504 | *PTPN1* |
| 7505 | *PTPN11* |
| 7506 | *PTPN12* |
| 7507 | *PTPN13* |
| 7508 | *PTPN14* |
| 7509 | *PTPN2* |
| 7510 | *PTPN21* |
| 7511 | *PTPN22* |
| 7512 | *PTPN23* |
| 7513 | *PTPN3* |
| 7514 | *PTPN4* |
| 7515 | *PTPN5* |
| 7516 | *PTPN6* |
| 7517 | *PTPN9* |
| 7518 | *PTPRA* |
| 7519 | *PTPRB* |
| 7520 | *PTPRC* |
| 7521 | *PTPRD* |
| 7522 | *PTPRF* |
| 7523 | *PTPRG* |
| 7524 | *PTPRH* |
| 7525 | *PTPRJ* |
| 7526 | *PTPRK* |
| 7527 | *PTPRN* |
| 7528 | *PTPRN2* |
| 7529 | *PTPRO* |
| 7530 | *PTPRQ* |
| 7531 | *PTPRR* |
| 7532 | *PTPRS* |
| 7533 | *PTPRT* |
| 7534 | *PTPRU* |
| 7535 | *PTPRZ1* |
| 7536 | *PTS* |
| 7537 | *PTTG1* |
| 7538 | *PTTG1IP* |
| 7539 | *PTTG3P* |
| 7540 | *PTX3* |
| 7541 | *PUDP* |
| 7542 | *PUF60* |
| 7543 | *PUM1* |
| 7544 | *PUM3* |
| 7545 | *PURA* |
| 7546 | *PURB* |
| 7547 | *PURG* |
| 7548 | *PURPL* |
| 7549 | *PUS1* |
| 7550 | *PUS10* |
| 7551 | *PUS7* |
| 7552 | *PVR* |
| 7553 | *PVRIG* |
| 7554 | *PVT1* |
| 7555 | *PWAR4* |
| 7556 | *PWP1* |
| 7557 | *PWP2* |
| 7558 | *PXDC1* |
| 7559 | *PXDN* |
| 7560 | *PXK* |
| 7561 | *PXN* |
| 7562 | *PYCARD* |
| 7563 | *PYCR1* |
| 7564 | *PYCR2* |
| 7565 | *PYGB* |
| 7566 | *PYGL* |
| 7567 | *PYGO1* |
| 7568 | *PYGO2* |
| 7569 | *PYROXD1* |
| 7570 | *PYY* |
| 7571 | *PZP* |
| 7572 | *QARS1* |
| 7573 | *QKI* |
| 7574 | *QPCT* |
| 7575 | *QPCTL* |
| 7576 | *QPRT* |
| 7577 | *QSOX2* |
| 7578 | *QTRT1* |
| 7579 | *R3HCC1* |
| 7580 | *R3HDM1* |
| 7581 | *R3HDM4* |
| 7582 | *RAB10* |
| 7583 | *RAB11A* |
| 7584 | *RAB11B* |
| 7585 | *RAB11FIP2* |
| 7586 | *RAB11FIP4* |
| 7587 | *RAB11FIP5* |
| 7588 | *RAB12* |
| 7589 | *RAB13* |
| 7590 | *RAB14* |
| 7591 | *RAB18* |
| 7592 | *RAB1A* |
| 7593 | *RAB1B* |
| 7594 | *RAB20* |
| 7595 | *RAB21* |
| 7596 | *RAB22A* |
| 7597 | *RAB23* |
| 7598 | *RAB25* |
| 7599 | *RAB27A* |
| 7600 | *RAB27B* |
| 7601 | *RAB31* |
| 7602 | *RAB32* |
| 7603 | *RAB35* |
| 7604 | *RAB38* |
| 7605 | *RAB3C* |
| 7606 | *RAB3D* |
| 7607 | *RAB3GAP1* |
| 7608 | *RAB40B* |
| 7609 | *RAB40C* |
| 7610 | *RAB43* |
| 7611 | *RAB44* |
| 7612 | *RAB4A* |
| 7613 | *RAB4B* |
| 7614 | *RAB5A* |
| 7615 | *RAB5C* |
| 7616 | *RAB5IF* |
| 7617 | *RAB6A* |
| 7618 | *RAB6B* |
| 7619 | *RAB7A* |
| 7620 | *RAB8A* |
| 7621 | *RABAC1* |
| 7622 | *RABEPK* |
| 7623 | *RABGEF1* |
| 7624 | *RABGGTA* |
| 7625 | *RABGGTB* |
| 7626 | *RAC1* |
| 7627 | *RAC2* |
| 7628 | *RAC3* |
| 7629 | *RACGAP1* |
| 7630 | *RACK1* |
| 7631 | *RAD1* |
| 7632 | *RAD17* |
| 7633 | *RAD18* |
| 7634 | *RAD21* |
| 7635 | *RAD23B* |
| 7636 | *RAD50* |
| 7637 | *RAD51* |
| 7638 | *RAD51AP1* |
| 7639 | *RAD51B* |
| 7640 | *RAD51C* |
| 7641 | *RAD51D* |
| 7642 | *RAD52* |
| 7643 | *RAD54B* |
| 7644 | *RAD54L* |
| 7645 | *RAD54L2P1* |
| 7646 | *RAD9A* |
| 7647 | *RAD9B* |
| 7648 | *RAE1* |
| 7649 | *RAET1E* |
| 7650 | *RAET1G* |
| 7651 | *RAF1* |
| 7652 | *RAG1* |
| 7653 | *RAG2* |
| 7654 | *RAI1* |
| 7655 | *RAI14* |
| 7656 | *RAI2* |
| 7657 | *RALA* |
| 7658 | *RALB* |
| 7659 | *RALBP1* |
| 7660 | *RALGAPA1* |
| 7661 | *RALGDS* |
| 7662 | *RALY* |
| 7663 | *RALYL* |
| 7664 | *RAMP3* |
| 7665 | *RAN* |
| 7666 | *RANBP1* |
| 7667 | *RANBP10* |
| 7668 | *RANBP2* |
| 7669 | *RANBP6* |
| 7670 | *RANBP9* |
| 7671 | *RANGAP1* |
| 7672 | *RAP1A* |
| 7673 | *RAP1B* |
| 7674 | *RAP1BP2* |
| 7675 | *RAP1GAP* |
| 7676 | *RAP1GDS1* |
| 7677 | *RAP2A* |
| 7678 | *RAP2B* |
| 7679 | *RAPGEF1* |
| 7680 | *RAPGEF3* |
| 7681 | *RAPGEF6* |
| 7682 | *RAPGEFL1* |
| 7683 | *RAPH1* |
| 7684 | *RARA* |
| 7685 | *RARB* |
| 7686 | *RARG* |
| 7687 | *RARRES1* |
| 7688 | *RARRES2* |
| 7689 | *RARS1* |
| 7690 | *RASA1* |
| 7691 | *RASA2* |
| 7692 | *RASAL1* |
| 7693 | *RASAL2* |
| 7694 | *RASD1* |
| 7695 | *RASD2* |
| 7696 | *RASGEF1B* |
| 7697 | *RASGRF1* |
| 7698 | *RASGRF2* |
| 7699 | *RASGRP1* |
| 7700 | *RASIP1* |
| 7701 | *RASL10B* |
| 7702 | *RASL11A* |
| 7703 | *RASSF1* |
| 7704 | *RASSF10* |
| 7705 | *RASSF2* |
| 7706 | *RASSF5* |
| 7707 | *RASSF6* |
| 7708 | *RASSF7* |
| 7709 | *RASSF8* |
| 7710 | *RAVER1* |
| 7711 | *RAVER2* |
| 7712 | *RB1* |
| 7713 | *RB1CC1* |
| 7714 | *RBBP4* |
| 7715 | *RBBP5* |
| 7716 | *RBBP6* |
| 7717 | *RBBP7* |
| 7718 | *RBBP8* |
| 7719 | *RBBP8NL* |
| 7720 | *RBCK1* |
| 7721 | *RBFOX1* |
| 7722 | *RBFOX2* |
| 7723 | *RBFOX3* |
| 7724 | *RBKS* |
| 7725 | *RBL1* |
| 7726 | *RBL2* |
| 7727 | *RBM10* |
| 7728 | *RBM12B* |
| 7729 | *RBM14* |
| 7730 | *RBM15B* |
| 7731 | *RBM17* |
| 7732 | *RBM19* |
| 7733 | *RBM24* |
| 7734 | *RBM25* |
| 7735 | *RBM28* |
| 7736 | *RBM3* |
| 7737 | *RBM33* |
| 7738 | *RBM34* |
| 7739 | *RBM38* |
| 7740 | *RBM39* |
| 7741 | *RBM4* |
| 7742 | *RBM45* |
| 7743 | *RBM47* |
| 7744 | *RBM8A* |
| 7745 | *RBMS2* |
| 7746 | *RBMS3* |
| 7747 | *RBMX* |
| 7748 | *RBMXL1* |
| 7749 | *RBMXL2* |
| 7750 | *RBP1* |
| 7751 | *RBP2* |
| 7752 | *RBP3* |
| 7753 | *RBP4* |
| 7754 | *RBPJ* |
| 7755 | *RBPMS* |
| 7756 | *RBPMS2* |
| 7757 | *RBX1* |
| 7758 | *RCAN1* |
| 7759 | *RCC1* |
| 7760 | *RCC2* |
| 7761 | *RCCD1* |
| 7762 | *RCE1* |
| 7763 | *RCHY1* |
| 7764 | *RCL1* |
| 7765 | *RCN1* |
| 7766 | *RCN2* |
| 7767 | *RCOR1* |
| 7768 | *RDH11* |
| 7769 | *RDH14* |
| 7770 | *RDX* |
| 7771 | *RECK* |
| 7772 | *RECQL* |
| 7773 | *RECQL4* |
| 7774 | *RECQL5* |
| 7775 | *REEP4* |
| 7776 | *REEP5* |
| 7777 | *REG1A* |
| 7778 | *REG1B* |
| 7779 | *REG3A* |
| 7780 | *REG4* |
| 7781 | *REL* |
| 7782 | *RELA* |
| 7783 | *RELB* |
| 7784 | *RELCH* |
| 7785 | *RELN* |
| 7786 | *REN* |
| 7787 | *RENBP* |
| 7788 | *REPIN1* |
| 7789 | *REPS1* |
| 7790 | *RERE* |
| 7791 | *RERG* |
| 7792 | *RERGL* |
| 7793 | *REST* |
| 7794 | *RET* |
| 7795 | *RETN* |
| 7796 | *RETNLB* |
| 7797 | *RETREG1* |
| 7798 | *RETREG3* |
| 7799 | *REV1* |
| 7800 | *REV3L* |
| 7801 | *REXO4* |
| 7802 | *RFC1* |
| 7803 | *RFC2* |
| 7804 | *RFC3* |
| 7805 | *RFC4* |
| 7806 | *RFC5* |
| 7807 | *RFLNA* |
| 7808 | *RFPL4A* |
| 7809 | *RFWD3* |
| 7810 | *RFX1* |
| 7811 | *RFX2* |
| 7812 | *RFX5* |
| 7813 | *RFX7* |
| 7814 | *RFXANK* |
| 7815 | *RGCC* |
| 7816 | *RGL1* |
| 7817 | *RGMA* |
| 7818 | *RGMB* |
| 7819 | *RGN* |
| 7820 | *RGPD2* |
| 7821 | *RGPD3* |
| 7822 | *RGPD4* |
| 7823 | *RGPD5* |
| 7824 | *RGPD8* |
| 7825 | *RGS1* |
| 7826 | *RGS10* |
| 7827 | *RGS12* |
| 7828 | *RGS16* |
| 7829 | *RGS17* |
| 7830 | *RGS19* |
| 7831 | *RGS2* |
| 7832 | *RGS6* |
| 7833 | *RHBDD1* |
| 7834 | *RHBDD2* |
| 7835 | *RHBDD3* |
| 7836 | *RHBDF1* |
| 7837 | *RHBDF2* |
| 7838 | *RHD* |
| 7839 | *RHEB* |
| 7840 | *RHEBL1* |
| 7841 | *RHO* |
| 7842 | *RHOA* |
| 7843 | *RHOB* |
| 7844 | *RHOBTB1* |
| 7845 | *RHOC* |
| 7846 | *RHOD* |
| 7847 | *RHOH* |
| 7848 | *RHOQ* |
| 7849 | *RHOT1* |
| 7850 | *RHOT2* |
| 7851 | *RHOU* |
| 7852 | *RHOV* |
| 7853 | *RHPN1* |
| 7854 | *RHPN2* |
| 7855 | *RIC3* |
| 7856 | *RIC8A* |
| 7857 | *RICTOR* |
| 7858 | *RIF1* |
| 7859 | *RIGI* |
| 7860 | *RILPL1* |
| 7861 | *RIMBP2* |
| 7862 | *RIMKLB* |
| 7863 | *RIMOC1* |
| 7864 | *RIN1* |
| 7865 | *RIN2* |
| 7866 | *RIN3* |
| 7867 | *RING1* |
| 7868 | *RINT1* |
| 7869 | *RIOK1* |
| 7870 | *RIOK2* |
| 7871 | *RIOK3* |
| 7872 | *RIOX1* |
| 7873 | *RIOX2* |
| 7874 | *RIPK1* |
| 7875 | *RIPK2* |
| 7876 | *RIPK3* |
| 7877 | *RIPOR3* |
| 7878 | *RLIM* |
| 7879 | *RMC1* |
| 7880 | *RMI1* |
| 7881 | *RN7SKP242* |
| 7882 | *RN7SKP293* |
| 7883 | *RN7SKP297* |
| 7884 | *RN7SL222P* |
| 7885 | *RN7SL381P* |
| 7886 | *RN7SL519P* |
| 7887 | *RN7SL547P* |
| 7888 | *RN7SL553P* |
| 7889 | *RN7SL569P* |
| 7890 | *RN7SL636P* |
| 7891 | *RN7SL643P* |
| 7892 | *RN7SL688P* |
| 7893 | *RN7SL804P* |
| 7894 | *RNA5SP299* |
| 7895 | *RNA5SP45* |
| 7896 | *RNA5SP79* |
| 7897 | *RNASE1* |
| 7898 | *RNASE3* |
| 7899 | *RNASEH2C* |
| 7900 | *RNASEL* |
| 7901 | *RNASET2* |
| 7902 | *RND3* |
| 7903 | *RNF10* |
| 7904 | *RNF111* |
| 7905 | *RNF114* |
| 7906 | *RNF121* |
| 7907 | *RNF125* |
| 7908 | *RNF126* |
| 7909 | *RNF128* |
| 7910 | *RNF138* |
| 7911 | *RNF141* |
| 7912 | *RNF146* |
| 7913 | *RNF149* |
| 7914 | *RNF152* |
| 7915 | *RNF167* |
| 7916 | *RNF168* |
| 7917 | *RNF182* |
| 7918 | *RNF183* |
| 7919 | *RNF186* |
| 7920 | *RNF19A* |
| 7921 | *RNF2* |
| 7922 | *RNF20* |
| 7923 | *RNF213* |
| 7924 | *RNF216* |
| 7925 | *RNF220* |
| 7926 | *RNF26* |
| 7927 | *RNF31* |
| 7928 | *RNF34* |
| 7929 | *RNF38* |
| 7930 | *RNF4* |
| 7931 | *RNF40* |
| 7932 | *RNF41* |
| 7933 | *RNF43* |
| 7934 | *RNF5* |
| 7935 | *RNF6* |
| 7936 | *RNF7* |
| 7937 | *RNGTT* |
| 7938 | *RNH1* |
| 7939 | *RNLS* |
| 7940 | *RNMT* |
| 7941 | *RNPC3* |
| 7942 | *RNPEP* |
| 7943 | *RNPEPL1* |
| 7944 | *RNPS1* |
| 7945 | *RNU1-103P* |
| 7946 | *RNU1-150P* |
| 7947 | *RNU1-76P* |
| 7948 | *RNU1-88P* |
| 7949 | *RNU4-10P* |
| 7950 | *RNU4-36P* |
| 7951 | *RNU4-50P* |
| 7952 | *RNU4-84P* |
| 7953 | *RNU6-1010P* |
| 7954 | *RNU6-1065P* |
| 7955 | *RNU6-1213P* |
| 7956 | *RNU6-185P* |
| 7957 | *RNU6-238P* |
| 7958 | *RNU6-253P* |
| 7959 | *RNU6-282P* |
| 7960 | *RNU6-351P* |
| 7961 | *RNU6-403P* |
| 7962 | *RNU6-41P* |
| 7963 | *RNU6-510P* |
| 7964 | *RNU6-636P* |
| 7965 | *RNU6-650P* |
| 7966 | *RNU6-669P* |
| 7967 | *RNU6-6P* |
| 7968 | *RNU6-72P* |
| 7969 | *RNU6-839P* |
| 7970 | *RNU7-11P* |
| 7971 | *RNU7-147P* |
| 7972 | *RNU7-76P* |
| 7973 | *RNY1P8* |
| 7974 | *ROBO1* |
| 7975 | *ROBO2* |
| 7976 | *ROBO3* |
| 7977 | *ROBO4* |
| 7978 | *ROCK1* |
| 7979 | *ROCK2* |
| 7980 | *ROMO1* |
| 7981 | *ROPN1L* |
| 7982 | *ROR1* |
| 7983 | *ROR1-AS1* |
| 7984 | *ROR2* |
| 7985 | *RORA* |
| 7986 | *RORB* |
| 7987 | *RORC* |
| 7988 | *ROS1* |
| 7989 | *RP1* |
| 7990 | *RP9* |
| 7991 | *RPA1* |
| 7992 | *RPA2* |
| 7993 | *RPA3* |
| 7994 | *RPAIN* |
| 7995 | *RPAP1* |
| 7996 | *RPE* |
| 7997 | *RPE65* |
| 7998 | *RPF2* |
| 7999 | *RPGR* |
| 8000 | *RPGRIP1* |
| 8001 | *RPH3A* |
| 8002 | *RPH3AL* |
| 8003 | *RPIA* |
| 8004 | *RPL10* |
| 8005 | *RPL10A* |
| 8006 | *RPL10L* |
| 8007 | *RPL11* |
| 8008 | *RPL12* |
| 8009 | *RPL13* |
| 8010 | *RPL13A* |
| 8011 | *RPL13AP20* |
| 8012 | *RPL13P5* |
| 8013 | *RPL14* |
| 8014 | *RPL15* |
| 8015 | *RPL15P21* |
| 8016 | *RPL17* |
| 8017 | *RPL17-C18orf32* |
| 8018 | *RPL17P25* |
| 8019 | *RPL17P39* |
| 8020 | *RPL17P46* |
| 8021 | *RPL18* |
| 8022 | *RPL18A* |
| 8023 | *RPL19* |
| 8024 | *RPL21* |
| 8025 | *RPL21P119* |
| 8026 | *RPL21P46* |
| 8027 | *RPL22* |
| 8028 | *RPL22L1* |
| 8029 | *RPL22P1* |
| 8030 | *RPL23* |
| 8031 | *RPL23A* |
| 8032 | *RPL23AP1* |
| 8033 | *RPL23AP12* |
| 8034 | *RPL23AP87* |
| 8035 | *RPL23P5* |
| 8036 | *RPL24* |
| 8037 | *RPL24P7* |
| 8038 | *RPL26* |
| 8039 | *RPL27* |
| 8040 | *RPL27A* |
| 8041 | *RPL27AP8* |
| 8042 | *RPL28* |
| 8043 | *RPL29* |
| 8044 | *RPL3* |
| 8045 | *RPL30* |
| 8046 | *RPL31* |
| 8047 | *RPL31P28* |
| 8048 | *RPL31P46* |
| 8049 | *RPL32* |
| 8050 | *RPL34* |
| 8051 | *RPL34-AS1* |
| 8052 | *RPL35* |
| 8053 | *RPL35A* |
| 8054 | *RPL36* |
| 8055 | *RPL36A* |
| 8056 | *RPL36AL* |
| 8057 | *RPL37* |
| 8058 | *RPL37A* |
| 8059 | *RPL38* |
| 8060 | *RPL39* |
| 8061 | *RPL39P5* |
| 8062 | *RPL4* |
| 8063 | *RPL5* |
| 8064 | *RPL6* |
| 8065 | *RPL7* |
| 8066 | *RPL7A* |
| 8067 | *RPL7AP69* |
| 8068 | *RPL7P1* |
| 8069 | *RPL8* |
| 8070 | *RPL9* |
| 8071 | *RPLP0* |
| 8072 | *RPLP0P2* |
| 8073 | *RPLP0P6* |
| 8074 | *RPLP1* |
| 8075 | *RPLP2* |
| 8076 | *RPN1* |
| 8077 | *RPN2* |
| 8078 | *RPP14* |
| 8079 | *RPP21* |
| 8080 | *RPP25L* |
| 8081 | *RPPH1* |
| 8082 | *RPRD1A* |
| 8083 | *RPRD1B* |
| 8084 | *RPRD2* |
| 8085 | *RPRM* |
| 8086 | *RPRML* |
| 8087 | *RPS10* |
| 8088 | *RPS11* |
| 8089 | *RPS12* |
| 8090 | *RPS13* |
| 8091 | *RPS14* |
| 8092 | *RPS15* |
| 8093 | *RPS15A* |
| 8094 | *RPS16* |
| 8095 | *RPS16P4* |
| 8096 | *RPS16P5* |
| 8097 | *RPS17* |
| 8098 | *RPS18* |
| 8099 | *RPS18P7* |
| 8100 | *RPS19* |
| 8101 | *RPS2* |
| 8102 | *RPS20* |
| 8103 | *RPS21* |
| 8104 | *RPS23* |
| 8105 | *RPS24* |
| 8106 | *RPS25* |
| 8107 | *RPS26* |
| 8108 | *RPS26P11* |
| 8109 | *RPS26P45* |
| 8110 | *RPS27* |
| 8111 | *RPS27A* |
| 8112 | *RPS27L* |
| 8113 | *RPS29* |
| 8114 | *RPS2P1* |
| 8115 | *RPS3* |
| 8116 | *RPS3A* |
| 8117 | *RPS3AP21* |
| 8118 | *RPS3AP46* |
| 8119 | *RPS3AP49* |
| 8120 | *RPS4X* |
| 8121 | *RPS5* |
| 8122 | *RPS6* |
| 8123 | *RPS6KA1* |
| 8124 | *RPS6KA2* |
| 8125 | *RPS6KA3* |
| 8126 | *RPS6KA4* |
| 8127 | *RPS6KA5* |
| 8128 | *RPS6KA6* |
| 8129 | *RPS6KB1* |
| 8130 | *RPS6KB2* |
| 8131 | *RPS6P16* |
| 8132 | *RPS7* |
| 8133 | *RPS7P5* |
| 8134 | *RPS8* |
| 8135 | *RPS9* |
| 8136 | *RPSA* |
| 8137 | *RPSAP72* |
| 8138 | *RPTOR* |
| 8139 | *RPUSD1* |
| 8140 | *RPUSD3* |
| 8141 | *RPUSD4* |
| 8142 | *RRAD* |
| 8143 | *RRAGA* |
| 8144 | *RRAGB* |
| 8145 | *RRAGC* |
| 8146 | *RRAGD* |
| 8147 | *RRAS* |
| 8148 | *RRAS2* |
| 8149 | *RRBP1* |
| 8150 | *RREB1* |
| 8151 | *RRM1* |
| 8152 | *RRM2* |
| 8153 | *RRM2B* |
| 8154 | *RRN3* |
| 8155 | *RRP1* |
| 8156 | *RRP15* |
| 8157 | *RRP9* |
| 8158 | *RRS1* |
| 8159 | *RS1* |
| 8160 | *RSBN1* |
| 8161 | *RSBN1L* |
| 8162 | *RSF1* |
| 8163 | *RSL1D1* |
| 8164 | *RSPH6A* |
| 8165 | *RSPO1* |
| 8166 | *RSPO2* |
| 8167 | *RSPO3* |
| 8168 | *RSPRY1* |
| 8169 | *RSRP1* |
| 8170 | *RSU1* |
| 8171 | *RTCA* |
| 8172 | *RTCB* |
| 8173 | *RTEL1* |
| 8174 | *RTEL1-TNFRSF6B* |
| 8175 | *RTF1* |
| 8176 | *RTKN* |
| 8177 | *RTKN2* |
| 8178 | *RTL10* |
| 8179 | *RTL8C* |
| 8180 | *RTN1* |
| 8181 | *RTN2* |
| 8182 | *RTN3P1* |
| 8183 | *RTN4* |
| 8184 | *RTN4R* |
| 8185 | *RTN4RL2* |
| 8186 | *RTP1* |
| 8187 | *RTRAF* |
| 8188 | *RUBCN* |
| 8189 | *RUFY1* |
| 8190 | *RUFY3* |
| 8191 | *RUNX1* |
| 8192 | *RUNX1-IT1* |
| 8193 | *RUNX1T1* |
| 8194 | *RUNX2* |
| 8195 | *RUNX3* |
| 8196 | *RUSC1* |
| 8197 | *RUSF1* |
| 8198 | *RUVBL1* |
| 8199 | *RUVBL2* |
| 8200 | *RWDD2A* |
| 8201 | *RXFP2* |
| 8202 | *RXFP4* |
| 8203 | *RXRA* |
| 8204 | *RXRB* |
| 8205 | *RYK* |
| 8206 | *RYR2* |
| 8207 | *RYR3* |
| 8208 | *S100A1* |
| 8209 | *S100A10* |
| 8210 | *S100A11* |
| 8211 | *S100A12* |
| 8212 | *S100A14* |
| 8213 | *S100A16* |
| 8214 | *S100A2* |
| 8215 | *S100A4* |
| 8216 | *S100A5* |
| 8217 | *S100A6* |
| 8218 | *S100A7* |
| 8219 | *S100A7A* |
| 8220 | *S100A8* |
| 8221 | *S100A9* |
| 8222 | *S100B* |
| 8223 | *S100G* |
| 8224 | *S100P* |
| 8225 | *S100PBP* |
| 8226 | *S1PR1* |
| 8227 | *S1PR2* |
| 8228 | *SAA1* |
| 8229 | *SAA2* |
| 8230 | *SAA3P* |
| 8231 | *SAA4* |
| 8232 | *SACS* |
| 8233 | *SAFB* |
| 8234 | *SAFB2* |
| 8235 | *SAGE1* |
| 8236 | *SAI1* |
| 8237 | *SALL1* |
| 8238 | *SALL3* |
| 8239 | *SALL4* |
| 8240 | *SALL4P5* |
| 8241 | *SAMD14* |
| 8242 | *SAMD3* |
| 8243 | *SAMD4B* |
| 8244 | *SAMHD1* |
| 8245 | *SANBR* |
| 8246 | *SAP130* |
| 8247 | *SAP18* |
| 8248 | *SAP30BP* |
| 8249 | *SAPCD1* |
| 8250 | *SAPCD2* |
| 8251 | *SAR1A* |
| 8252 | *SAR1B* |
| 8253 | *SARDH* |
| 8254 | *SARS1* |
| 8255 | *SARS2* |
| 8256 | *SART1* |
| 8257 | *SART3* |
| 8258 | *SASH1* |
| 8259 | *SASS6* |
| 8260 | *SAT1* |
| 8261 | *SAT2* |
| 8262 | *SATB1* |
| 8263 | *SATB1-AS1* |
| 8264 | *SATB2* |
| 8265 | *SATB2-AS1* |
| 8266 | *SAV1* |
| 8267 | *SBDS* |
| 8268 | *SBDSP1* |
| 8269 | *SBF1* |
| 8270 | *SBF2* |
| 8271 | *SBF2-AS1* |
| 8272 | *SBNO1* |
| 8273 | *SBNO2* |
| 8274 | *SBSN* |
| 8275 | *SBSPON* |
| 8276 | *SC5D* |
| 8277 | *SCAF1* |
| 8278 | *SCAF8* |
| 8279 | *SCAI* |
| 8280 | *SCAMP3* |
| 8281 | *SCAP* |
| 8282 | *SCARA5* |
| 8283 | *SCARB2* |
| 8284 | *SCARF2* |
| 8285 | *SCARNA2* |
| 8286 | *SCD* |
| 8287 | *SCEL* |
| 8288 | *SCFD1* |
| 8289 | *SCFD2* |
| 8290 | *SCG2* |
| 8291 | *SCG5* |
| 8292 | *SCGB2A1* |
| 8293 | *SCGB2A2* |
| 8294 | *SCGB3A1* |
| 8295 | *SCGB3A2* |
| 8296 | *SCGN* |
| 8297 | *SCIN* |
| 8298 | *SCLC1* |
| 8299 | *SCN10A* |
| 8300 | *SCN1A* |
| 8301 | *SCN2A* |
| 8302 | *SCN3B* |
| 8303 | *SCN5A* |
| 8304 | *SCN7A* |
| 8305 | *SCN8A* |
| 8306 | *SCNN1B* |
| 8307 | *SCNN1G* |
| 8308 | *SCO1* |
| 8309 | *SCO2* |
| 8310 | *SCP2* |
| 8311 | *SCPEP1* |
| 8312 | *SCRIB* |
| 8313 | *SCRN1* |
| 8314 | *SCRN3* |
| 8315 | *SCT* |
| 8316 | *SCTR* |
| 8317 | *SCUBE2* |
| 8318 | *SCYL1* |
| 8319 | *SCYL2* |
| 8320 | *SCYL3* |
| 8321 | *SDC1* |
| 8322 | *SDC2* |
| 8323 | *SDC3* |
| 8324 | *SDC4* |
| 8325 | *SDCBP* |
| 8326 | *SDCBP2* |
| 8327 | *SDF2L1* |
| 8328 | *SDF4* |
| 8329 | *SDHA* |
| 8330 | *SDHAF2* |
| 8331 | *SDHB* |
| 8332 | *SDHC* |
| 8333 | *SDHD* |
| 8334 | *SDHDP3* |
| 8335 | *SDK2* |
| 8336 | *SDR16C5* |
| 8337 | *SDR39U1* |
| 8338 | *SEC11A* |
| 8339 | *SEC13* |
| 8340 | *SEC14L2* |
| 8341 | *SEC16A* |
| 8342 | *SEC1P* |
| 8343 | *SEC23A* |
| 8344 | *SEC23B* |
| 8345 | *SEC24A* |
| 8346 | *SEC24C* |
| 8347 | *SEC31A* |
| 8348 | *SEC61A1* |
| 8349 | *SEC61A2* |
| 8350 | *SEC61B* |
| 8351 | *SEC61G* |
| 8352 | *SEC62* |
| 8353 | *SEC63* |
| 8354 | *SEC63P1* |
| 8355 | *SECISBP2* |
| 8356 | *SECISBP2L* |
| 8357 | *SEL1L* |
| 8358 | *SELE* |
| 8359 | *SELENBP1* |
| 8360 | *SELENOF* |
| 8361 | *SELENOH* |
| 8362 | *SELENOK* |
| 8363 | *SELENOP* |
| 8364 | *SELENOS* |
| 8365 | *SELENOW* |
| 8366 | *SELL* |
| 8367 | *SELP* |
| 8368 | *SEMA3A* |
| 8369 | *SEMA3B* |
| 8370 | *SEMA3C* |
| 8371 | *SEMA3D* |
| 8372 | *SEMA3E* |
| 8373 | *SEMA3F* |
| 8374 | *SEMA4A* |
| 8375 | *SEMA4C* |
| 8376 | *SEMA4D* |
| 8377 | *SEMA4F* |
| 8378 | *SEMA4G* |
| 8379 | *SEMA5A* |
| 8380 | *SEMA5B* |
| 8381 | *SEMA6A* |
| 8382 | *SEMA6D* |
| 8383 | *SEMG1* |
| 8384 | *SENP1* |
| 8385 | *SENP2* |
| 8386 | *SENP7* |
| 8387 | *SENP8* |
| 8388 | *SEPHS1* |
| 8389 | *SEPTIN1* |
| 8390 | *SEPTIN10* |
| 8391 | *SEPTIN14P20* |
| 8392 | *SEPTIN2* |
| 8393 | *SEPTIN4* |
| 8394 | *SEPTIN6* |
| 8395 | *SEPTIN7* |
| 8396 | *SEPTIN8* |
| 8397 | *SEPTIN9* |
| 8398 | *SERBP1* |
| 8399 | *SERBP1P3* |
| 8400 | *SERF1A* |
| 8401 | *SERF2* |
| 8402 | *SERPINA1* |
| 8403 | *SERPINA10* |
| 8404 | *SERPINA12* |
| 8405 | *SERPINA2* |
| 8406 | *SERPINA3* |
| 8407 | *SERPINA4* |
| 8408 | *SERPINA7* |
| 8409 | *SERPINB1* |
| 8410 | *SERPINB11* |
| 8411 | *SERPINB12* |
| 8412 | *SERPINB2* |
| 8413 | *SERPINB3* |
| 8414 | *SERPINB5* |
| 8415 | *SERPINB6* |
| 8416 | *SERPINB8* |
| 8417 | *SERPINB9* |
| 8418 | *SERPINC1* |
| 8419 | *SERPINE1* |
| 8420 | *SERPINE2* |
| 8421 | *SERPINF1* |
| 8422 | *SERPING1* |
| 8423 | *SERPINH1* |
| 8424 | *SERPINI1* |
| 8425 | *SERPINI2* |
| 8426 | *SERTAD1* |
| 8427 | *SERTAD2* |
| 8428 | *SERTAD3* |
| 8429 | *SESN1* |
| 8430 | *SESN2* |
| 8431 | *SET* |
| 8432 | *SETBP1* |
| 8433 | *SETD1A* |
| 8434 | *SETD1B* |
| 8435 | *SETD2* |
| 8436 | *SETD3* |
| 8437 | *SETD4* |
| 8438 | *SETD6* |
| 8439 | *SETD7* |
| 8440 | *SETD9* |
| 8441 | *SETDB1* |
| 8442 | *SETDB2* |
| 8443 | *SETMAR* |
| 8444 | *SEZ6* |
| 8445 | *SEZ6L* |
| 8446 | *SEZ6L2* |
| 8447 | *SF1* |
| 8448 | *SF3A1* |
| 8449 | *SF3A2* |
| 8450 | *SF3A3* |
| 8451 | *SF3B1* |
| 8452 | *SF3B2* |
| 8453 | *SF3B3* |
| 8454 | *SF3B4* |
| 8455 | *SF3B6* |
| 8456 | *SFMBT1* |
| 8457 | *SFN* |
| 8458 | *SFPQ* |
| 8459 | *SFRP1* |
| 8460 | *SFRP2* |
| 8461 | *SFRP4* |
| 8462 | *SFRP5* |
| 8463 | *SFT2D1* |
| 8464 | *SFT2D3* |
| 8465 | *SFTPB* |
| 8466 | *SFTPC* |
| 8467 | *SFTPD* |
| 8468 | *SFXN1* |
| 8469 | *SFXN2* |
| 8470 | *SGCB* |
| 8471 | *SGCE* |
| 8472 | *SGK1* |
| 8473 | *SGK2* |
| 8474 | *SGK3* |
| 8475 | *SGMS1* |
| 8476 | *SGMS2* |
| 8477 | *SGO1* |
| 8478 | *SGPL1* |
| 8479 | *SGPP1* |
| 8480 | *SGSM3* |
| 8481 | *SGTA* |
| 8482 | *SH2B1* |
| 8483 | *SH2B3* |
| 8484 | *SH2D3A* |
| 8485 | *SH2D3C* |
| 8486 | *SH2D4A* |
| 8487 | *SH2D4B* |
| 8488 | *SH3BGRL* |
| 8489 | *SH3BGRL2* |
| 8490 | *SH3BP1* |
| 8491 | *SH3BP2* |
| 8492 | *SH3GL1* |
| 8493 | *SH3GL3* |
| 8494 | *SH3GLB1* |
| 8495 | *SH3GLB2* |
| 8496 | *SH3KBP1* |
| 8497 | *SH3PXD2A* |
| 8498 | *SH3PXD2A-AS1* |
| 8499 | *SH3TC1* |
| 8500 | *SH3TC2* |
| 8501 | *SHANK1* |
| 8502 | *SHANK3* |
| 8503 | *SHARPIN* |
| 8504 | *SHBG* |
| 8505 | *SHC1* |
| 8506 | *SHC3* |
| 8507 | *SHC4* |
| 8508 | *SHCBP1* |
| 8509 | *SHCBP1L* |
| 8510 | *SHH* |
| 8511 | *SHISA3* |
| 8512 | *SHKBP1* |
| 8513 | *SHMT1* |
| 8514 | *SHMT2* |
| 8515 | *SHOC2* |
| 8516 | *SHOX2* |
| 8517 | *SHROOM2* |
| 8518 | *SHROOM3* |
| 8519 | *SHROOM4* |
| 8520 | *SHTN1* |
| 8521 | *SI* |
| 8522 | *SIAH1* |
| 8523 | *SIAH2* |
| 8524 | *SIGIRR* |
| 8525 | *SIGLEC1* |
| 8526 | *SIGLEC10* |
| 8527 | *SIGLEC11* |
| 8528 | *SIGLEC5* |
| 8529 | *SIGLEC6* |
| 8530 | *SIGLEC7* |
| 8531 | *SIGLEC9* |
| 8532 | *SIGMAR1* |
| 8533 | *SIK1* |
| 8534 | *SIK1B* |
| 8535 | *SIK2* |
| 8536 | *SIK3* |
| 8537 | *SIKE1* |
| 8538 | *SIM2* |
| 8539 | *SIPA1* |
| 8540 | *SIPA1L1* |
| 8541 | *SIPA1L3* |
| 8542 | *SIRPA* |
| 8543 | *SIRT1* |
| 8544 | *SIRT2* |
| 8545 | *SIRT3* |
| 8546 | *SIRT4* |
| 8547 | *SIRT5* |
| 8548 | *SIRT6* |
| 8549 | *SIRT7* |
| 8550 | *SIVA1* |
| 8551 | *SIX1* |
| 8552 | *SIX2* |
| 8553 | *SIX4* |
| 8554 | *SKA1* |
| 8555 | *SKA3* |
| 8556 | *SKAP1* |
| 8557 | *SKI* |
| 8558 | *SKIC2* |
| 8559 | *SKIC8* |
| 8560 | *SKIDA1* |
| 8561 | *SKIL* |
| 8562 | *SKIV2L* |
| 8563 | *SKOR1* |
| 8564 | *SKP1* |
| 8565 | *SKP2* |
| 8566 | *SLA* |
| 8567 | *SLAIN2* |
| 8568 | *SLAMF1* |
| 8569 | *SLAMF6* |
| 8570 | *SLAMF7* |
| 8571 | *SLC10A2* |
| 8572 | *SLC10A3* |
| 8573 | *SLC11A1* |
| 8574 | *SLC11A2* |
| 8575 | *SLC12A1* |
| 8576 | *SLC12A3* |
| 8577 | *SLC12A4* |
| 8578 | *SLC12A5* |
| 8579 | *SLC12A6* |
| 8580 | *SLC12A9* |
| 8581 | *SLC13A5* |
| 8582 | *SLC14A1* |
| 8583 | *SLC14A2* |
| 8584 | *SLC15A1* |
| 8585 | *SLC15A4* |
| 8586 | *SLC16A1* |
| 8587 | *SLC16A3* |
| 8588 | *SLC16A4* |
| 8589 | *SLC16A7* |
| 8590 | *SLC16A9* |
| 8591 | *SLC17A1* |
| 8592 | *SLC17A5* |
| 8593 | *SLC17A6* |
| 8594 | *SLC17A9* |
| 8595 | *SLC18A1* |
| 8596 | *SLC18A2* |
| 8597 | *SLC19A1* |
| 8598 | *SLC19A2* |
| 8599 | *SLC1A1* |
| 8600 | *SLC1A2* |
| 8601 | *SLC1A3* |
| 8602 | *SLC1A4* |
| 8603 | *SLC1A5* |
| 8604 | *SLC1A7* |
| 8605 | *SLC22A1* |
| 8606 | *SLC22A15* |
| 8607 | *SLC22A16* |
| 8608 | *SLC22A18* |
| 8609 | *SLC22A2* |
| 8610 | *SLC22A3* |
| 8611 | *SLC22A4* |
| 8612 | *SLC22A5* |
| 8613 | *SLC22A7* |
| 8614 | *SLC22A9* |
| 8615 | *SLC23A1* |
| 8616 | *SLC23A2* |
| 8617 | *SLC23A3* |
| 8618 | *SLC24A3* |
| 8619 | *SLC25A1* |
| 8620 | *SLC25A10* |
| 8621 | *SLC25A11* |
| 8622 | *SLC25A12* |
| 8623 | *SLC25A13* |
| 8624 | *SLC25A18* |
| 8625 | *SLC25A19* |
| 8626 | *SLC25A20* |
| 8627 | *SLC25A21* |
| 8628 | *SLC25A22* |
| 8629 | *SLC25A24* |
| 8630 | *SLC25A25-AS1* |
| 8631 | *SLC25A26* |
| 8632 | *SLC25A28* |
| 8633 | *SLC25A3* |
| 8634 | *SLC25A4* |
| 8635 | *SLC25A40* |
| 8636 | *SLC25A46* |
| 8637 | *SLC25A47P1* |
| 8638 | *SLC25A5* |
| 8639 | *SLC25A51* |
| 8640 | *SLC25A6* |
| 8641 | *SLC26A10* |
| 8642 | *SLC26A10P* |
| 8643 | *SLC26A2* |
| 8644 | *SLC26A3* |
| 8645 | *SLC26A4* |
| 8646 | *SLC26A8* |
| 8647 | *SLC27A4* |
| 8648 | *SLC28A2* |
| 8649 | *SLC29A1* |
| 8650 | *SLC29A2* |
| 8651 | *SLC2A1* |
| 8652 | *SLC2A2* |
| 8653 | *SLC2A3* |
| 8654 | *SLC2A4* |
| 8655 | *SLC2A4RG* |
| 8656 | *SLC2A5* |
| 8657 | *SLC30A10* |
| 8658 | *SLC30A9* |
| 8659 | *SLC31A1* |
| 8660 | *SLC33A1* |
| 8661 | *SLC34A2* |
| 8662 | *SLC35A1* |
| 8663 | *SLC35A2* |
| 8664 | *SLC35A3* |
| 8665 | *SLC35B2* |
| 8666 | *SLC35B3* |
| 8667 | *SLC35C1* |
| 8668 | *SLC35E2A* |
| 8669 | *SLC35E2B* |
| 8670 | *SLC35F2* |
| 8671 | *SLC35F5* |
| 8672 | *SLC35G1* |
| 8673 | *SLC36A1* |
| 8674 | *SLC36A4* |
| 8675 | *SLC37A1* |
| 8676 | *SLC37A4* |
| 8677 | *SLC38A1* |
| 8678 | *SLC38A7* |
| 8679 | *SLC38A9* |
| 8680 | *SLC39A10* |
| 8681 | *SLC39A11* |
| 8682 | *SLC39A12* |
| 8683 | *SLC39A14* |
| 8684 | *SLC39A7* |
| 8685 | *SLC39A8* |
| 8686 | *SLC3A2* |
| 8687 | *SLC41A2* |
| 8688 | *SLC43A3* |
| 8689 | *SLC44A4* |
| 8690 | *SLC44A5* |
| 8691 | *SLC45A3* |
| 8692 | *SLC46A1* |
| 8693 | *SLC47A1* |
| 8694 | *SLC4A1* |
| 8695 | *SLC4A1AP* |
| 8696 | *SLC4A2* |
| 8697 | *SLC4A4* |
| 8698 | *SLC4A7* |
| 8699 | *SLC51B* |
| 8700 | *SLC52A1* |
| 8701 | *SLC52A2* |
| 8702 | *SLC52A3* |
| 8703 | *SLC5A1* |
| 8704 | *SLC5A2* |
| 8705 | *SLC5A5* |
| 8706 | *SLC5A7* |
| 8707 | *SLC5A8* |
| 8708 | *SLC66A1* |
| 8709 | *SLC66A2* |
| 8710 | *SLC6A1* |
| 8711 | *SLC6A12* |
| 8712 | *SLC6A14* |
| 8713 | *SLC6A18* |
| 8714 | *SLC6A2* |
| 8715 | *SLC6A3* |
| 8716 | *SLC6A4* |
| 8717 | *SLC6A5* |
| 8718 | *SLC6A6* |
| 8719 | *SLC6A7* |
| 8720 | *SLC6A8* |
| 8721 | *SLC7A1* |
| 8722 | *SLC7A11* |
| 8723 | *SLC7A2* |
| 8724 | *SLC7A4* |
| 8725 | *SLC7A5* |
| 8726 | *SLC7A6* |
| 8727 | *SLC7A7* |
| 8728 | *SLC8A1* |
| 8729 | *SLC8A3* |
| 8730 | *SLC8B1* |
| 8731 | *SLC9A1* |
| 8732 | *SLC9A2* |
| 8733 | *SLC9A3R1* |
| 8734 | *SLC9A3R2* |
| 8735 | *SLC9A9* |
| 8736 | *SLCO1A2* |
| 8737 | *SLCO1B1* |
| 8738 | *SLCO1B3* |
| 8739 | *SLCO2A1* |
| 8740 | *SLCO2B1* |
| 8741 | *SLCO3A1* |
| 8742 | *SLCO4A1* |
| 8743 | *SLCO4A1-AS1* |
| 8744 | *SLCO6A1* |
| 8745 | *SLFN11* |
| 8746 | *SLFN5* |
| 8747 | *SLIRP* |
| 8748 | *SLIT1* |
| 8749 | *SLIT2* |
| 8750 | *SLIT3* |
| 8751 | *SLITRK1* |
| 8752 | *SLITRK4* |
| 8753 | *SLITRK6* |
| 8754 | *SLMAP* |
| 8755 | *SLPI* |
| 8756 | *SLURP1* |
| 8757 | *SLX4* |
| 8758 | *SMAD1* |
| 8759 | *SMAD2* |
| 8760 | *SMAD3* |
| 8761 | *SMAD4* |
| 8762 | *SMAD5* |
| 8763 | *SMAD6* |
| 8764 | *SMAD7* |
| 8765 | *SMAD9* |
| 8766 | *SMAGP* |
| 8767 | *SMAP1* |
| 8768 | *SMARCA1* |
| 8769 | *SMARCA2* |
| 8770 | *SMARCA4* |
| 8771 | *SMARCA5* |
| 8772 | *SMARCAD1* |
| 8773 | *SMARCAL1* |
| 8774 | *SMARCB1* |
| 8775 | *SMARCC1* |
| 8776 | *SMARCC2* |
| 8777 | *SMARCD1* |
| 8778 | *SMARCD3* |
| 8779 | *SMARCE1* |
| 8780 | *SMC1A* |
| 8781 | *SMC1B* |
| 8782 | *SMC2* |
| 8783 | *SMC3* |
| 8784 | *SMC4* |
| 8785 | *SMC6* |
| 8786 | *SMCO1* |
| 8787 | *SMCR8* |
| 8788 | *SMG1* |
| 8789 | *SMG7* |
| 8790 | *SMIM14* |
| 8791 | *SMIM20* |
| 8792 | *SMIM3* |
| 8793 | *SMIM38* |
| 8794 | *SMLR1* |
| 8795 | *SMN1* |
| 8796 | *SMN2* |
| 8797 | *SMO* |
| 8798 | *SMOC2* |
| 8799 | *SMOX* |
| 8800 | *SMPD1* |
| 8801 | *SMPD3* |
| 8802 | *SMPD4* |
| 8803 | *SMPDL3B* |
| 8804 | *SMR3B* |
| 8805 | *SMS* |
| 8806 | *SMTN* |
| 8807 | *SMUG1* |
| 8808 | *SMURF1* |
| 8809 | *SMURF2* |
| 8810 | *SMYD2* |
| 8811 | *SMYD3* |
| 8812 | *SNAI1* |
| 8813 | *SNAI2* |
| 8814 | *SNAI3* |
| 8815 | *SNAP23* |
| 8816 | *SNAP25* |
| 8817 | *SNAP47* |
| 8818 | *SNAP91* |
| 8819 | *SNCA* |
| 8820 | *SNCB* |
| 8821 | *SNCG* |
| 8822 | *SND1* |
| 8823 | *SNED1* |
| 8824 | *SNF8* |
| 8825 | *SNHG1* |
| 8826 | *SNHG11* |
| 8827 | *SNHG12* |
| 8828 | *SNHG14* |
| 8829 | *SNHG15* |
| 8830 | *SNHG16* |
| 8831 | *SNHG17* |
| 8832 | *SNHG20* |
| 8833 | *SNHG3* |
| 8834 | *SNHG5* |
| 8835 | *SNHG6* |
| 8836 | *SNHG7* |
| 8837 | *SNHG8* |
| 8838 | *SNORA21* |
| 8839 | *SNORA80E* |
| 8840 | *SNORD126* |
| 8841 | *SNORD12B* |
| 8842 | *SNORD138* |
| 8843 | *SNORD14B* |
| 8844 | *SNORD14C* |
| 8845 | *SNORD14D* |
| 8846 | *SNORD14E* |
| 8847 | *SNORD15A* |
| 8848 | *SNORD33* |
| 8849 | *SNORD35B* |
| 8850 | *SNORD44* |
| 8851 | *SNRNP200* |
| 8852 | *SNRNP25* |
| 8853 | *SNRNP40* |
| 8854 | *SNRNP70* |
| 8855 | *SNRPA* |
| 8856 | *SNRPA1* |
| 8857 | *SNRPB* |
| 8858 | *SNRPB2* |
| 8859 | *SNRPD1* |
| 8860 | *SNRPD2* |
| 8861 | *SNRPD3* |
| 8862 | *SNRPE* |
| 8863 | *SNRPF* |
| 8864 | *SNRPG* |
| 8865 | *SNRPGP15* |
| 8866 | *SNRPN* |
| 8867 | *SNTB2* |
| 8868 | *SNU13* |
| 8869 | *SNW1* |
| 8870 | *SNX1* |
| 8871 | *SNX10* |
| 8872 | *SNX14* |
| 8873 | *SNX16* |
| 8874 | *SNX18* |
| 8875 | *SNX22* |
| 8876 | *SNX27* |
| 8877 | *SNX3* |
| 8878 | *SNX30* |
| 8879 | *SNX31* |
| 8880 | *SNX8* |
| 8881 | *SNX9* |
| 8882 | *SOAT1* |
| 8883 | *SOAT2* |
| 8884 | *SOCS1* |
| 8885 | *SOCS2* |
| 8886 | *SOCS3* |
| 8887 | *SOCS5P5* |
| 8888 | *SOCS6* |
| 8889 | *SOCS7* |
| 8890 | *SOD1* |
| 8891 | *SOD2* |
| 8892 | *SOD3* |
| 8893 | *SOHLH1* |
| 8894 | *SORBS1* |
| 8895 | *SORBS2* |
| 8896 | *SORBS3* |
| 8897 | *SORCS1* |
| 8898 | *SORCS3* |
| 8899 | *SORD* |
| 8900 | *SORL1* |
| 8901 | *SORT1* |
| 8902 | *SOS1* |
| 8903 | *SOS2* |
| 8904 | *SOST* |
| 8905 | *SOSTDC1* |
| 8906 | *SOX10* |
| 8907 | *SOX11* |
| 8908 | *SOX12* |
| 8909 | *SOX13* |
| 8910 | *SOX15* |
| 8911 | *SOX17* |
| 8912 | *SOX18* |
| 8913 | *SOX2* |
| 8914 | *SOX21* |
| 8915 | *SOX21-AS1* |
| 8916 | *SOX4* |
| 8917 | *SOX5* |
| 8918 | *SOX6* |
| 8919 | *SOX7* |
| 8920 | *SOX8* |
| 8921 | *SOX9* |
| 8922 | *SP1* |
| 8923 | *SP110* |
| 8924 | *SP3* |
| 8925 | *SPACA5* |
| 8926 | *SPACA5B* |
| 8927 | *SPACA7* |
| 8928 | *SPAG11B* |
| 8929 | *SPAG17* |
| 8930 | *SPAG6* |
| 8931 | *SPAG9* |
| 8932 | *SPANXA1* |
| 8933 | *SPANXA2* |
| 8934 | *SPANXB1* |
| 8935 | *SPARC* |
| 8936 | *SPARCL1* |
| 8937 | *SPART* |
| 8938 | *SPAST* |
| 8939 | *SPATA13* |
| 8940 | *SPATA18* |
| 8941 | *SPATA2* |
| 8942 | *SPATA20* |
| 8943 | *SPATA21* |
| 8944 | *SPATS2L* |
| 8945 | *SPC24* |
| 8946 | *SPDEF* |
| 8947 | *SPDL1* |
| 8948 | *SPDYA* |
| 8949 | *SPDYE16* |
| 8950 | *SPECC1* |
| 8951 | *SPECC1L* |
| 8952 | *SPEF2* |
| 8953 | *SPEN* |
| 8954 | *SPG11* |
| 8955 | *SPG7* |
| 8956 | *SPHK1* |
| 8957 | *SPHK2* |
| 8958 | *SPI1* |
| 8959 | *SPIB* |
| 8960 | *SPIC* |
| 8961 | *SPIDR* |
| 8962 | *SPIN1* |
| 8963 | *SPIN3* |
| 8964 | *SPINDOC* |
| 8965 | *SPINK1* |
| 8966 | *SPINK4* |
| 8967 | *SPINT1* |
| 8968 | *SPINT2* |
| 8969 | *SPN* |
| 8970 | *SPNS2* |
| 8971 | *SPNS3* |
| 8972 | *SPOCD1* |
| 8973 | *SPOCK1* |
| 8974 | *SPOCK2* |
| 8975 | *SPON1* |
| 8976 | *SPON2* |
| 8977 | *SPOP* |
| 8978 | *SPOUT1* |
| 8979 | *SPP1* |
| 8980 | *SPP2* |
| 8981 | *SPRED1* |
| 8982 | *SPRED2* |
| 8983 | *SPRR1B* |
| 8984 | *SPRR2A* |
| 8985 | *SPRR2F* |
| 8986 | *SPRR3* |
| 8987 | *SPRY1* |
| 8988 | *SPRY2* |
| 8989 | *SPRY4* |
| 8990 | *SPRY4-IT1* |
| 8991 | *SPRYD7* |
| 8992 | *SPSB1* |
| 8993 | *SPSB2* |
| 8994 | *SPSB4* |
| 8995 | *SPTA1* |
| 8996 | *SPTAN1* |
| 8997 | *SPTB* |
| 8998 | *SPTBN1* |
| 8999 | *SPTBN2* |
| 9000 | *SPTBN4* |
| 9001 | *SPTLC1* |
| 9002 | *SPTY2D1* |
| 9003 | *SPZ1* |
| 9004 | *SQLE* |
| 9005 | *SQSTM1* |
| 9006 | *SRA1* |
| 9007 | *SRARP* |
| 9008 | *SRBD1* |
| 9009 | *SRC* |
| 9010 | *SRCIN1* |
| 9011 | *SRD5A2* |
| 9012 | *SREBF1* |
| 9013 | *SREBF2* |
| 9014 | *SRF* |
| 9015 | *SRGAP1* |
| 9016 | *SRGAP2* |
| 9017 | *SRGAP2B* |
| 9018 | *SRGAP3* |
| 9019 | *SRGN* |
| 9020 | *SRI* |
| 9021 | *SRL* |
| 9022 | *SRM* |
| 9023 | *SRMS* |
| 9024 | *SRP14* |
| 9025 | *SRP19* |
| 9026 | *SRP9* |
| 9027 | *SRPK1* |
| 9028 | *SRPK2* |
| 9029 | *SRPRB* |
| 9030 | *SRPX* |
| 9031 | *SRPX2* |
| 9032 | *SRRM1* |
| 9033 | *SRRM1P1* |
| 9034 | *SRRM2* |
| 9035 | *SRSF1* |
| 9036 | *SRSF10* |
| 9037 | *SRSF10P2* |
| 9038 | *SRSF2* |
| 9039 | *SRSF3* |
| 9040 | *SRSF4* |
| 9041 | *SRSF5* |
| 9042 | *SRSF6* |
| 9043 | *SRSF7* |
| 9044 | *SRSF9* |
| 9045 | *SRXN1* |
| 9046 | *SRY* |
| 9047 | *SS18L1* |
| 9048 | *SSAV1* |
| 9049 | *SSBP1* |
| 9050 | *SSBP4* |
| 9051 | *SSH1* |
| 9052 | *SSH2* |
| 9053 | *SSH3* |
| 9054 | *SSNA1* |
| 9055 | *SSPN* |
| 9056 | *SSR1* |
| 9057 | *SSR2* |
| 9058 | *SSR4* |
| 9059 | *SSRP1* |
| 9060 | *SST* |
| 9061 | *SSTR1* |
| 9062 | *SSTR2* |
| 9063 | *SSTR3* |
| 9064 | *SSTR4* |
| 9065 | *SSTR5* |
| 9066 | *SSX2* |
| 9067 | *SSX2B* |
| 9068 | *ST13* |
| 9069 | *ST13P5* |
| 9070 | *ST14* |
| 9071 | *ST2* |
| 9072 | *ST20* |
| 9073 | *ST3GAL1* |
| 9074 | *ST3GAL2* |
| 9075 | *ST3GAL3* |
| 9076 | *ST3GAL4* |
| 9077 | *ST3GAL5* |
| 9078 | *ST3GAL6* |
| 9079 | *ST3GAL6-AS1* |
| 9080 | *ST6GAL1* |
| 9081 | *ST6GALNAC1* |
| 9082 | *ST6GALNAC2* |
| 9083 | *ST7* |
| 9084 | *ST8* |
| 9085 | *ST8SIA1* |
| 9086 | *ST8SIA2* |
| 9087 | *ST8SIA4* |
| 9088 | *STAB1* |
| 9089 | *STAG1* |
| 9090 | *STAG2* |
| 9091 | *STAG3* |
| 9092 | *STAG3L3* |
| 9093 | *STAM* |
| 9094 | *STAMBP* |
| 9095 | *STAMBPL1* |
| 9096 | *STAP2* |
| 9097 | *STAR* |
| 9098 | *STARD13* |
| 9099 | *STARD13-AS* |
| 9100 | *STARD7* |
| 9101 | *STARD8* |
| 9102 | *STAT1* |
| 9103 | *STAT2* |
| 9104 | *STAT3* |
| 9105 | *STAT4* |
| 9106 | *STAT5A* |
| 9107 | *STAT5B* |
| 9108 | *STAT6* |
| 9109 | *STATH* |
| 9110 | *STAU1* |
| 9111 | *STAU2* |
| 9112 | *STC1* |
| 9113 | *STC2* |
| 9114 | *STEAP1* |
| 9115 | *STEAP2* |
| 9116 | *STEAP3* |
| 9117 | *STEAP4* |
| 9118 | *STIM1* |
| 9119 | *STIM2* |
| 9120 | *STING1* |
| 9121 | *STIP1* |
| 9122 | *STK10* |
| 9123 | *STK11* |
| 9124 | *STK11IP* |
| 9125 | *STK17A* |
| 9126 | *STK17B* |
| 9127 | *STK24* |
| 9128 | *STK25* |
| 9129 | *STK26* |
| 9130 | *STK3* |
| 9131 | *STK31* |
| 9132 | *STK32C* |
| 9133 | *STK33* |
| 9134 | *STK35* |
| 9135 | *STK36* |
| 9136 | *STK38* |
| 9137 | *STK38L* |
| 9138 | *STK39* |
| 9139 | *STK4* |
| 9140 | *STK40* |
| 9141 | *STMN1* |
| 9142 | *STMN2* |
| 9143 | *STMN3* |
| 9144 | *STN1* |
| 9145 | *STOM* |
| 9146 | *STOML2* |
| 9147 | *STON1* |
| 9148 | *STPG1* |
| 9149 | *STPG2* |
| 9150 | *STRA6* |
| 9151 | *STRADA* |
| 9152 | *STRAP* |
| 9153 | *STRBP* |
| 9154 | *STRIP1* |
| 9155 | *STRN* |
| 9156 | *STRN3* |
| 9157 | *STRN4* |
| 9158 | *STS* |
| 9159 | *STT3A* |
| 9160 | *STUB1* |
| 9161 | *STX12* |
| 9162 | *STX17* |
| 9163 | *STX1A* |
| 9164 | *STX2* |
| 9165 | *STX5* |
| 9166 | *STXBP1* |
| 9167 | *STYK1* |
| 9168 | *STYX* |
| 9169 | *STYXL1* |
| 9170 | *SUB1* |
| 9171 | *SUCLA2* |
| 9172 | *SUCLG1* |
| 9173 | *SUCO* |
| 9174 | *SUDS3* |
| 9175 | *SUFU* |
| 9176 | *SUGCT* |
| 9177 | *SUGP1* |
| 9178 | *SUGP2* |
| 9179 | *SUGT1* |
| 9180 | *SULF1* |
| 9181 | *SULF2* |
| 9182 | *SULT1A1* |
| 9183 | *SULT1A2* |
| 9184 | *SULT1B1* |
| 9185 | *SULT1C2* |
| 9186 | *SULT1E1* |
| 9187 | *SULT2A1* |
| 9188 | *SULT2B1* |
| 9189 | *SUMF2* |
| 9190 | *SUMO1* |
| 9191 | *SUMO1P3* |
| 9192 | *SUMO2* |
| 9193 | *SUMO2P1* |
| 9194 | *SUMO3* |
| 9195 | *SUN1* |
| 9196 | *SUN2* |
| 9197 | *SUPT16H* |
| 9198 | *SUPT4H1* |
| 9199 | *SUPT5H* |
| 9200 | *SUPT6H* |
| 9201 | *SURF1* |
| 9202 | *SURF4* |
| 9203 | *SUSD1* |
| 9204 | *SUSD3* |
| 9205 | *SUSD6* |
| 9206 | *SUV39H1* |
| 9207 | *SUV39H2* |
| 9208 | *SUZ12* |
| 9209 | *SVIL* |
| 9210 | *SYK* |
| 9211 | *SYMPK* |
| 9212 | *SYNCRIP* |
| 9213 | *SYNE1* |
| 9214 | *SYNE2* |
| 9215 | *SYNGAP1* |
| 9216 | *SYNJ2* |
| 9217 | *SYNJ2BP* |
| 9218 | *SYNM* |
| 9219 | *SYNPO* |
| 9220 | *SYNPO2* |
| 9221 | *SYP* |
| 9222 | *SYPL1* |
| 9223 | *SYT1* |
| 9224 | *SYT13* |
| 9225 | *SYT3* |
| 9226 | *SYT7* |
| 9227 | *SYT9* |
| 9228 | *SYTL2* |
| 9229 | *SYTL5* |
| 9230 | *SYVN1* |
| 9231 | *TAB1* |
| 9232 | *TAB3* |
| 9233 | *TAC1* |
| 9234 | *TACC1* |
| 9235 | *TACC2* |
| 9236 | *TACC3* |
| 9237 | *TACR1* |
| 9238 | *TACR2* |
| 9239 | *TACSTD2* |
| 9240 | *TADA1* |
| 9241 | *TADA2B* |
| 9242 | *TAF1* |
| 9243 | *TAF11* |
| 9244 | *TAF12* |
| 9245 | *TAF15* |
| 9246 | *TAF1A* |
| 9247 | *TAF1B* |
| 9248 | *TAF1C* |
| 9249 | *TAF1L* |
| 9250 | *TAF4* |
| 9251 | *TAF7L* |
| 9252 | *TAF8* |
| 9253 | *TAFAZZIN* |
| 9254 | *TAGLN* |
| 9255 | *TAGLN2* |
| 9256 | *TALDO1* |
| 9257 | *TAM* |
| 9258 | *TAMM41* |
| 9259 | *TANC1* |
| 9260 | *TANC2* |
| 9261 | *TANGO2* |
| 9262 | *TANGO6* |
| 9263 | *TANK* |
| 9264 | *TAOK1* |
| 9265 | *TAP1* |
| 9266 | *TAP2* |
| 9267 | *TAPBP* |
| 9268 | *TAPBPL* |
| 9269 | *TARBP2* |
| 9270 | *TARDBP* |
| 9271 | *TARDBPP1* |
| 9272 | *TARS1* |
| 9273 | *TARS2* |
| 9274 | *TAS1R3* |
| 9275 | *TAS2R13* |
| 9276 | *TAS2R14* |
| 9277 | *TAS2R16* |
| 9278 | *TAS2R31* |
| 9279 | *TAS2R38* |
| 9280 | *TAS2R43* |
| 9281 | *TAS2R46* |
| 9282 | *TAS2R64P* |
| 9283 | *TAT* |
| 9284 | *TATDN1* |
| 9285 | *TAX1BP1* |
| 9286 | *TAX1BP3* |
| 9287 | *TAZ* |
| 9288 | *TBC1D10B* |
| 9289 | *TBC1D13* |
| 9290 | *TBC1D15* |
| 9291 | *TBC1D16* |
| 9292 | *TBC1D17* |
| 9293 | *TBC1D2* |
| 9294 | *TBC1D22B* |
| 9295 | *TBC1D25* |
| 9296 | *TBC1D7* |
| 9297 | *TBC1D9* |
| 9298 | *TBC1D9B* |
| 9299 | *TBCA* |
| 9300 | *TBCB* |
| 9301 | *TBCD* |
| 9302 | *TBCE* |
| 9303 | *TBCEL* |
| 9304 | *TBCK* |
| 9305 | *TBK1* |
| 9306 | *TBL1X* |
| 9307 | *TBL1XR1* |
| 9308 | *TBL2* |
| 9309 | *TBP* |
| 9310 | *TBPL1* |
| 9311 | *TBR1* |
| 9312 | *TBRG4* |
| 9313 | *TBX1* |
| 9314 | *TBX19* |
| 9315 | *TBX2* |
| 9316 | *TBX20* |
| 9317 | *TBX21* |
| 9318 | *TBX22* |
| 9319 | *TBX3* |
| 9320 | *TBX4* |
| 9321 | *TBX5* |
| 9322 | *TBXA2R* |
| 9323 | *TBXAS1* |
| 9324 | *TBXT* |
| 9325 | *TCEA1P2* |
| 9326 | *TCEA2* |
| 9327 | *TCEA3* |
| 9328 | *TCEAL1* |
| 9329 | *TCEAL9* |
| 9330 | *TCERG1* |
| 9331 | *TCERG1L* |
| 9332 | *TCF12* |
| 9333 | *TCF15* |
| 9334 | *TCF19* |
| 9335 | *TCF21* |
| 9336 | *TCF25* |
| 9337 | *TCF3* |
| 9338 | *TCF4* |
| 9339 | *TCF7* |
| 9340 | *TCF7L1* |
| 9341 | *TCF7L2* |
| 9342 | *TCFL5* |
| 9343 | *TCHH* |
| 9344 | *TCHP* |
| 9345 | *TCIM* |
| 9346 | *TCIRG1* |
| 9347 | *TCL1A* |
| 9348 | *TCN1* |
| 9349 | *TCN2* |
| 9350 | *TCOF1* |
| 9351 | *TCP1* |
| 9352 | *TCP10L* |
| 9353 | *TCTA* |
| 9354 | *TCTN1* |
| 9355 | *TDG* |
| 9356 | *TDGF1* |
| 9357 | *TDGF1P3* |
| 9358 | *TDO2* |
| 9359 | *TDP1* |
| 9360 | *TDP2* |
| 9361 | *TDRD1* |
| 9362 | *TDRD3* |
| 9363 | *TDRD7* |
| 9364 | *TDRD9* |
| 9365 | *TEAD1* |
| 9366 | *TEAD3* |
| 9367 | *TEAD4* |
| 9368 | *TEC* |
| 9369 | *TECR* |
| 9370 | *TECTA* |
| 9371 | *TECTB* |
| 9372 | *TEDC1* |
| 9373 | *TEDC2* |
| 9374 | *TEK* |
| 9375 | *TEKT4* |
| 9376 | *TELO2* |
| 9377 | *TENM1* |
| 9378 | *TENM2* |
| 9379 | *TENM3* |
| 9380 | *TENM3-AS1* |
| 9381 | *TENM4* |
| 9382 | *TENT4A* |
| 9383 | *TENT5A* |
| 9384 | *TENT5C* |
| 9385 | *TEP1* |
| 9386 | *TERC* |
| 9387 | *TERF1* |
| 9388 | *TERF2* |
| 9389 | *TERF2IP* |
| 9390 | *TERT* |
| 9391 | *TES* |
| 9392 | *TESC* |
| 9393 | *TESK1* |
| 9394 | *TET1* |
| 9395 | *TET2* |
| 9396 | *TET3* |
| 9397 | *TEX22* |
| 9398 | *TEX264* |
| 9399 | *TEX28P2* |
| 9400 | *TEX37* |
| 9401 | *TEX43* |
| 9402 | *TEX9* |
| 9403 | *TF* |
| 9404 | *TFAM* |
| 9405 | *TFAP2A* |
| 9406 | *TFAP2C* |
| 9407 | *TFAP2D* |
| 9408 | *TFAP2E* |
| 9409 | *TFAP4* |
| 9410 | *TFB1M* |
| 9411 | *TFCP2* |
| 9412 | *TFCP2L1* |
| 9413 | *TFDP1* |
| 9414 | *TFDP2* |
| 9415 | *TFE3* |
| 9416 | *TFEB* |
| 9417 | *TFEC* |
| 9418 | *TFF1* |
| 9419 | *TFF2* |
| 9420 | *TFF3* |
| 9421 | *TFG* |
| 9422 | *TFIP11* |
| 9423 | *TFPI* |
| 9424 | *TFPI2* |
| 9425 | *TFR2* |
| 9426 | *TFRC* |
| 9427 | *TG* |
| 9428 | *TGFA* |
| 9429 | *TGFB1* |
| 9430 | *TGFB1I1* |
| 9431 | *TGFB2* |
| 9432 | *TGFB3* |
| 9433 | *TGFBI* |
| 9434 | *TGFBR1* |
| 9435 | *TGFBR2* |
| 9436 | *TGFBR3* |
| 9437 | *TGFBRAP1* |
| 9438 | *TGIF1* |
| 9439 | *TGIF2LX* |
| 9440 | *TGM1* |
| 9441 | *TGM2* |
| 9442 | *TGM3* |
| 9443 | *TGM4* |
| 9444 | *TH* |
| 9445 | *THADA* |
| 9446 | *THAP11* |
| 9447 | *THAS* |
| 9448 | *THBD* |
| 9449 | *THBS1* |
| 9450 | *THBS2* |
| 9451 | *THBS3* |
| 9452 | *THBS4* |
| 9453 | *THEM6* |
| 9454 | *THEMIS* |
| 9455 | *THM* |
| 9456 | *THOC1* |
| 9457 | *THOC2* |
| 9458 | *THOC3* |
| 9459 | *THOC5* |
| 9460 | *THOC7* |
| 9461 | *THOP1* |
| 9462 | *THPO* |
| 9463 | *THRA* |
| 9464 | *THRAP3* |
| 9465 | *THRB* |
| 9466 | *THSD1* |
| 9467 | *THSD4* |
| 9468 | *THSD7A* |
| 9469 | *THSD7B* |
| 9470 | *THUMPD1* |
| 9471 | *THUMPD3* |
| 9472 | *THY1* |
| 9473 | *TIA1* |
| 9474 | *TIAL1* |
| 9475 | *TIAM1* |
| 9476 | *TIAM2* |
| 9477 | *TICAM1* |
| 9478 | *TICAM2* |
| 9479 | *TIE1* |
| 9480 | *TIFA* |
| 9481 | *TIGAR* |
| 9482 | *TIGD1* |
| 9483 | *TIGIT* |
| 9484 | *TIMD4* |
| 9485 | *TIMELESS* |
| 9486 | *TIMM10* |
| 9487 | *TIMM13* |
| 9488 | *TIMM22* |
| 9489 | *TIMM44* |
| 9490 | *TIMM50* |
| 9491 | *TIMM8A* |
| 9492 | *TIMM9* |
| 9493 | *TIMMDC1* |
| 9494 | *TIMP1* |
| 9495 | *TIMP2* |
| 9496 | *TIMP3* |
| 9497 | *TIMP4* |
| 9498 | *TINAG* |
| 9499 | *TINAGL1* |
| 9500 | *TINCR* |
| 9501 | *TIPARP* |
| 9502 | *TIPIN* |
| 9503 | *TIRAP* |
| 9504 | *TJAP1* |
| 9505 | *TJP1* |
| 9506 | *TJP2* |
| 9507 | *TK1* |
| 9508 | *TKFC* |
| 9509 | *TKT* |
| 9510 | *TKTL1* |
| 9511 | *TLCD1* |
| 9512 | *TLE1* |
| 9513 | *TLE3* |
| 9514 | *TLE4* |
| 9515 | *TLE5* |
| 9516 | *TLE6* |
| 9517 | *TLK2* |
| 9518 | *TLL1* |
| 9519 | *TLN1* |
| 9520 | *TLN2* |
| 9521 | *TLR1* |
| 9522 | *TLR10* |
| 9523 | *TLR2* |
| 9524 | *TLR3* |
| 9525 | *TLR4* |
| 9526 | *TLR5* |
| 9527 | *TLR6* |
| 9528 | *TLR7* |
| 9529 | *TLR8* |
| 9530 | *TLR9* |
| 9531 | *TLX1* |
| 9532 | *TM2D2* |
| 9533 | *TM4SF1* |
| 9534 | *TM4SF4* |
| 9535 | *TM4SF5* |
| 9536 | *TM6SF2* |
| 9537 | *TM9SF2* |
| 9538 | *TM9SF3* |
| 9539 | *TMA7* |
| 9540 | *TMBIM1* |
| 9541 | *TMCC1* |
| 9542 | *TMCO1* |
| 9543 | *TMCO5B* |
| 9544 | *TMED1* |
| 9545 | *TMED10* |
| 9546 | *TMED2* |
| 9547 | *TMED4* |
| 9548 | *TMED5* |
| 9549 | *TMED6* |
| 9550 | *TMED7* |
| 9551 | *TMED7-TICAM2* |
| 9552 | *TMEFF1* |
| 9553 | *TMEFF2* |
| 9554 | *TMEM106B* |
| 9555 | *TMEM116* |
| 9556 | *TMEM125* |
| 9557 | *TMEM126A* |
| 9558 | *TMEM126B* |
| 9559 | *TMEM129* |
| 9560 | *TMEM134* |
| 9561 | *TMEM140* |
| 9562 | *TMEM145* |
| 9563 | *TMEM14A* |
| 9564 | *TMEM14B* |
| 9565 | *TMEM14C* |
| 9566 | *TMEM14DP* |
| 9567 | *TMEM158* |
| 9568 | *TMEM160* |
| 9569 | *TMEM161A* |
| 9570 | *TMEM164* |
| 9571 | *TMEM170B* |
| 9572 | *TMEM176A* |
| 9573 | *TMEM184A* |
| 9574 | *TMEM189* |
| 9575 | *TMEM189-UBE2V1* |
| 9576 | *TMEM192* |
| 9577 | *TMEM200A* |
| 9578 | *TMEM205* |
| 9579 | *TMEM207* |
| 9580 | *TMEM208* |
| 9581 | *TMEM209* |
| 9582 | *TMEM212* |
| 9583 | *TMEM220* |
| 9584 | *TMEM220-AS1* |
| 9585 | *TMEM230* |
| 9586 | *TMEM238L* |
| 9587 | *TMEM240* |
| 9588 | *TMEM25* |
| 9589 | *TMEM258* |
| 9590 | *TMEM259* |
| 9591 | *TMEM33* |
| 9592 | *TMEM39A* |
| 9593 | *TMEM43* |
| 9594 | *TMEM45A* |
| 9595 | *TMEM51* |
| 9596 | *TMEM54* |
| 9597 | *TMEM65* |
| 9598 | *TMEM75* |
| 9599 | *TMEM8B* |
| 9600 | *TMEM9* |
| 9601 | *TMEM91* |
| 9602 | *TMEM97* |
| 9603 | *TMEM9B* |
| 9604 | *TMIGD1* |
| 9605 | *TMIGD2* |
| 9606 | *TMOD1* |
| 9607 | *TMOD3* |
| 9608 | *TMPO* |
| 9609 | *TMPRSS11A* |
| 9610 | *TMPRSS12* |
| 9611 | *TMPRSS13* |
| 9612 | *TMPRSS2* |
| 9613 | *TMPRSS3* |
| 9614 | *TMPRSS4* |
| 9615 | *TMPRSS6* |
| 9616 | *TMSB10* |
| 9617 | *TMSB4X* |
| 9618 | *TMTC1* |
| 9619 | *TMTC4* |
| 9620 | *TMUB1* |
| 9621 | *TMX1* |
| 9622 | *TMX2-CTNND1* |
| 9623 | *TNC* |
| 9624 | *TNF* |
| 9625 | *TNFAIP3* |
| 9626 | *TNFAIP6* |
| 9627 | *TNFAIP8* |
| 9628 | *TNFAIP8L1* |
| 9629 | *TNFAIP8L2* |
| 9630 | *TNFRSF10A* |
| 9631 | *TNFRSF10B* |
| 9632 | *TNFRSF10C* |
| 9633 | *TNFRSF10D* |
| 9634 | *TNFRSF11A* |
| 9635 | *TNFRSF11B* |
| 9636 | *TNFRSF12A* |
| 9637 | *TNFRSF14* |
| 9638 | *TNFRSF17* |
| 9639 | *TNFRSF18* |
| 9640 | *TNFRSF19* |
| 9641 | *TNFRSF1A* |
| 9642 | *TNFRSF1B* |
| 9643 | *TNFRSF25* |
| 9644 | *TNFRSF4* |
| 9645 | *TNFRSF6B* |
| 9646 | *TNFRSF8* |
| 9647 | *TNFRSF9* |
| 9648 | *TNFSF10* |
| 9649 | *TNFSF12* |
| 9650 | *TNFSF13* |
| 9651 | *TNFSF13B* |
| 9652 | *TNFSF14* |
| 9653 | *TNFSF15* |
| 9654 | *TNFSF4* |
| 9655 | *TNFSF8* |
| 9656 | *TNFSF9* |
| 9657 | *TNIK* |
| 9658 | *TNIP1* |
| 9659 | *TNIP2* |
| 9660 | *TNK2* |
| 9661 | *TNKS* |
| 9662 | *TNKS1BP1* |
| 9663 | *TNKS2* |
| 9664 | *TNN* |
| 9665 | *TNNI3* |
| 9666 | *TNNI3K* |
| 9667 | *TNNT1* |
| 9668 | *TNNT2* |
| 9669 | *TNPO1* |
| 9670 | *TNRC6A* |
| 9671 | *TNRC6B* |
| 9672 | *TNS1* |
| 9673 | *TNS3* |
| 9674 | *TNS4* |
| 9675 | *TNXB* |
| 9676 | *TOE1* |
| 9677 | *TOM1L1* |
| 9678 | *TOM1L2* |
| 9679 | *TOMM20* |
| 9680 | *TOMM22* |
| 9681 | *TOMM34* |
| 9682 | *TOMM40L* |
| 9683 | *TOP1* |
| 9684 | *TOP1MT* |
| 9685 | *TOP2A* |
| 9686 | *TOP2B* |
| 9687 | *TOP3A* |
| 9688 | *TOP3B* |
| 9689 | *TOPBP1* |
| 9690 | *TOR1AIP1* |
| 9691 | *TOX* |
| 9692 | *TOX2* |
| 9693 | *TOX3* |
| 9694 | *TP53* |
| 9695 | *TP53AIP1* |
| 9696 | *TP53BP1* |
| 9697 | *TP53BP2* |
| 9698 | *TP53COR1* |
| 9699 | *TP53I11* |
| 9700 | *TP53I3* |
| 9701 | *TP53INP1* |
| 9702 | *TP53INP2* |
| 9703 | *TP53RK* |
| 9704 | *TP53TG1* |
| 9705 | *TP63* |
| 9706 | *TP73* |
| 9707 | *TP73-AS1* |
| 9708 | *TPBG* |
| 9709 | *TPCN2* |
| 9710 | *TPD52* |
| 9711 | *TPH1* |
| 9712 | *TPH2* |
| 9713 | *TPI1* |
| 9714 | *TPM1* |
| 9715 | *TPM2* |
| 9716 | *TPM3* |
| 9717 | *TPM3P6* |
| 9718 | *TPM3P9* |
| 9719 | *TPM4* |
| 9720 | *TPMT* |
| 9721 | *TPO* |
| 9722 | *TPP1* |
| 9723 | *TPP2* |
| 9724 | *TPPP3* |
| 9725 | *TPR* |
| 9726 | *TPSAB1* |
| 9727 | *TPST1* |
| 9728 | *TPT1* |
| 9729 | *TPTE* |
| 9730 | *TPTE2* |
| 9731 | *TPTE2P1* |
| 9732 | *TPTEP2-CSNK1E* |
| 9733 | *TPX2* |
| 9734 | *TRA2A* |
| 9735 | *TRA2B* |
| 9736 | *TRADD* |
| 9737 | *TRAF1* |
| 9738 | *TRAF2* |
| 9739 | *TRAF3* |
| 9740 | *TRAF3IP3* |
| 9741 | *TRAF4* |
| 9742 | *TRAF5* |
| 9743 | *TRAF6* |
| 9744 | *TRAJ56* |
| 9745 | *TRAK1* |
| 9746 | *TRAK2* |
| 9747 | *TRAM1* |
| 9748 | *TRANK1* |
| 9749 | *TRAP* |
| 9750 | *TRAP1* |
| 9751 | *TRAPPC10* |
| 9752 | *TRAPPC12* |
| 9753 | *TRAPPC3* |
| 9754 | *TRAPPC4* |
| 9755 | *TRAPPC8* |
| 9756 | *TRAPPC9* |
| 9757 | *TRAT1* |
| 9758 | *TRBV20OR9-2* |
| 9759 | *TRC-GCA24-1* |
| 9760 | *TRDMT1* |
| 9761 | *TREH* |
| 9762 | *TREM1* |
| 9763 | *TREM2* |
| 9764 | *TREML1* |
| 9765 | *TREML3P* |
| 9766 | *TREX2* |
| 9767 | *TRG* |
| 9768 | *TRIB1* |
| 9769 | *TRIB2* |
| 9770 | *TRIB3* |
| 9771 | *TRIM11* |
| 9772 | *TRIM13* |
| 9773 | *TRIM14* |
| 9774 | *TRIM16* |
| 9775 | *TRIM17* |
| 9776 | *TRIM2* |
| 9777 | *TRIM21* |
| 9778 | *TRIM23* |
| 9779 | *TRIM24* |
| 9780 | *TRIM25* |
| 9781 | *TRIM26* |
| 9782 | *TRIM27* |
| 9783 | *TRIM28* |
| 9784 | *TRIM29* |
| 9785 | *TRIM3* |
| 9786 | *TRIM31* |
| 9787 | *TRIM32* |
| 9788 | *TRIM33* |
| 9789 | *TRIM37* |
| 9790 | *TRIM39* |
| 9791 | *TRIM44* |
| 9792 | *TRIM46* |
| 9793 | *TRIM47* |
| 9794 | *TRIM52* |
| 9795 | *TRIM58* |
| 9796 | *TRIM59* |
| 9797 | *TRIM6* |
| 9798 | *TRIM65* |
| 9799 | *TRIM66* |
| 9800 | *TRIM67* |
| 9801 | *TRIM68* |
| 9802 | *TRIM72* |
| 9803 | *TRIM8* |
| 9804 | *TRIM9* |
| 9805 | *TRIML1* |
| 9806 | *TRIO* |
| 9807 | *TRIOBP* |
| 9808 | *TRIP10* |
| 9809 | *TRIP11* |
| 9810 | *TRIP12* |
| 9811 | *TRIP13* |
| 9812 | *TRIP4* |
| 9813 | *TRIP6* |
| 9814 | *TRIR* |
| 9815 | *TRMO* |
| 9816 | *TRMT1* |
| 9817 | *TRMT10A* |
| 9818 | *TRMT10C* |
| 9819 | *TRMT12* |
| 9820 | *TRMT2A* |
| 9821 | *TRMT44* |
| 9822 | *TRMT9B* |
| 9823 | *TRNF* |
| 9824 | *TRO* |
| 9825 | *TROAP* |
| 9826 | *TRPA1* |
| 9827 | *TRPC1* |
| 9828 | *TRPC4* |
| 9829 | *TRPC5* |
| 9830 | *TRPC6* |
| 9831 | *TRPM1* |
| 9832 | *TRPM2* |
| 9833 | *TRPM4* |
| 9834 | *TRPM6* |
| 9835 | *TRPM7* |
| 9836 | *TRPM8* |
| 9837 | *TRPS1* |
| 9838 | *TRPV1* |
| 9839 | *TRPV2* |
| 9840 | *TRPV4* |
| 9841 | *TRPV6* |
| 9842 | *TRRAP* |
| 9843 | *TRUB2* |
| 9844 | *TS13* |
| 9845 | *TSC1* |
| 9846 | *TSC2* |
| 9847 | *TSC22D1* |
| 9848 | *TSC22D2* |
| 9849 | *TSC22D3* |
| 9850 | *TSC22D4* |
| 9851 | *TSEN15* |
| 9852 | *TSEN34* |
| 9853 | *TSFM* |
| 9854 | *TSG101* |
| 9855 | *TSHZ1* |
| 9856 | *TSKS* |
| 9857 | *TSKU* |
| 9858 | *TSLP* |
| 9859 | *TSN* |
| 9860 | *TSNAX* |
| 9861 | *TSPAN1* |
| 9862 | *TSPAN12* |
| 9863 | *TSPAN17* |
| 9864 | *TSPAN32* |
| 9865 | *TSPAN4* |
| 9866 | *TSPAN6* |
| 9867 | *TSPAN8* |
| 9868 | *TSPAN9* |
| 9869 | *TSPEAR* |
| 9870 | *TSPO* |
| 9871 | *TSPYL2* |
| 9872 | *TSPYL5* |
| 9873 | *TST* |
| 9874 | *TSTD1* |
| 9875 | *TSTD2* |
| 9876 | *TTBK2* |
| 9877 | *TTC17* |
| 9878 | *TTC22* |
| 9879 | *TTC28* |
| 9880 | *TTC3* |
| 9881 | *TTC33* |
| 9882 | *TTC4P1* |
| 9883 | *TTF1* |
| 9884 | *TTF2* |
| 9885 | *TTK* |
| 9886 | *TTLL10* |
| 9887 | *TTLL12* |
| 9888 | *TTLL3* |
| 9889 | *TTN* |
| 9890 | *TTN-AS1* |
| 9891 | *TTPAL* |
| 9892 | *TTR* |
| 9893 | *TTYH2* |
| 9894 | *TUBA1A* |
| 9895 | *TUBA1B* |
| 9896 | *TUBA1C* |
| 9897 | *TUBA3C* |
| 9898 | *TUBA3D* |
| 9899 | *TUBA3E* |
| 9900 | *TUBA4A* |
| 9901 | *TUBA4B* |
| 9902 | *TUBB* |
| 9903 | *TUBB2A* |
| 9904 | *TUBB2B* |
| 9905 | *TUBB3* |
| 9906 | *TUBB4A* |
| 9907 | *TUBB4B* |
| 9908 | *TUBB6* |
| 9909 | *TUBB8* |
| 9910 | *TUBD1* |
| 9911 | *TUBG1* |
| 9912 | *TUBG2* |
| 9913 | *TUBGCP2* |
| 9914 | *TUBGCP3* |
| 9915 | *TUBGCP4* |
| 9916 | *TUFM* |
| 9917 | *TUFT1* |
| 9918 | *TUG1* |
| 9919 | *TULP1* |
| 9920 | *TULP3* |
| 9921 | *TUSC1* |
| 9922 | *TUSC2* |
| 9923 | *TUSC3* |
| 9924 | *TUSC7* |
| 9925 | *TUT4* |
| 9926 | *TVP23A* |
| 9927 | *TVP23B* |
| 9928 | *TWF1* |
| 9929 | *TWF2* |
| 9930 | *TWIST1* |
| 9931 | *TWIST2* |
| 9932 | *TWNK* |
| 9933 | *TWSG1* |
| 9934 | *TXLNA* |
| 9935 | *TXLNG* |
| 9936 | *TXLNGY* |
| 9937 | *TXN* |
| 9938 | *TXN2* |
| 9939 | *TXNDC12* |
| 9940 | *TXNDC15* |
| 9941 | *TXNDC5* |
| 9942 | *TXNDC9* |
| 9943 | *TXNIP* |
| 9944 | *TXNL1* |
| 9945 | *TXNL4A* |
| 9946 | *TXNRD1* |
| 9947 | *TXNRD2* |
| 9948 | *TXNRD3* |
| 9949 | *TYK2* |
| 9950 | *TYMP* |
| 9951 | *TYMS* |
| 9952 | *TYR* |
| 9953 | *TYRO3* |
| 9954 | *TYROBP* |
| 9955 | *TYRP1* |
| 9956 | *U2AF1* |
| 9957 | *U2AF2* |
| 9958 | *U2SURP* |
| 9959 | *UACA* |
| 9960 | *UAP1* |
| 9961 | *UBA1* |
| 9962 | *UBA2* |
| 9963 | *UBA5* |
| 9964 | *UBA52* |
| 9965 | *UBAC1* |
| 9966 | *UBAC2* |
| 9967 | *UBAP1* |
| 9968 | *UBAP2* |
| 9969 | *UBAP2L* |
| 9970 | *UBASH3A* |
| 9971 | *UBB* |
| 9972 | *UBBP1* |
| 9973 | *UBC* |
| 9974 | *UBD* |
| 9975 | *UBE2C* |
| 9976 | *UBE2D1* |
| 9977 | *UBE2D2* |
| 9978 | *UBE2D3* |
| 9979 | *UBE2D4* |
| 9980 | *UBE2E1* |
| 9981 | *UBE2F* |
| 9982 | *UBE2I* |
| 9983 | *UBE2J2* |
| 9984 | *UBE2L3* |
| 9985 | *UBE2M* |
| 9986 | *UBE2N* |
| 9987 | *UBE2NL* |
| 9988 | *UBE2O* |
| 9989 | *UBE2Q1* |
| 9990 | *UBE2Q2* |
| 9991 | *UBE2S* |
| 9992 | *UBE2T* |
| 9993 | *UBE2V1* |
| 9994 | *UBE2V2* |
| 9995 | *UBE2Z* |
| 9996 | *UBE3A* |
| 9997 | *UBE3C* |
| 9998 | *UBE4A* |
| 9999 | *UBE4B* |
| 10000 | *UBFD1* |
| 10001 | *UBL4A* |
| 10002 | *UBP1* |
| 10003 | *UBQLN1* |
| 10004 | *UBQLN1P1* |
| 10005 | *UBR2* |
| 10006 | *UBR4* |
| 10007 | *UBR5* |
| 10008 | *UBTD1* |
| 10009 | *UBTD2* |
| 10010 | *UBTF* |
| 10011 | *UBXN1* |
| 10012 | *UBXN11* |
| 10013 | *UBXN2A* |
| 10014 | *UBXN4* |
| 10015 | *UBXN6* |
| 10016 | *UCA1* |
| 10017 | *UCHL1* |
| 10018 | *UCHL5* |
| 10019 | *UCK2* |
| 10020 | *UCN* |
| 10021 | *UCN2* |
| 10022 | *UCN3* |
| 10023 | *UCP1* |
| 10024 | *UCP2* |
| 10025 | *UFD1* |
| 10026 | *UGCG* |
| 10027 | *UGDH* |
| 10028 | *UGP2* |
| 10029 | *UGT1A* |
| 10030 | *UGT1A1* |
| 10031 | *UGT1A10* |
| 10032 | *UGT1A3* |
| 10033 | *UGT1A4* |
| 10034 | *UGT1A6* |
| 10035 | *UGT1A7* |
| 10036 | *UGT1A8* |
| 10037 | *UGT1A9* |
| 10038 | *UGT2B15* |
| 10039 | *UGT2B17* |
| 10040 | *UGT2B28* |
| 10041 | *UGT2B7* |
| 10042 | *UGT3A2* |
| 10043 | *UHMK1* |
| 10044 | *UHRF1* |
| 10045 | *UHRF2* |
| 10046 | *ULBP2* |
| 10047 | *ULK1* |
| 10048 | *ULK2* |
| 10049 | *UMODL1* |
| 10050 | *UMPS* |
| 10051 | *UNC13B* |
| 10052 | *UNC45A* |
| 10053 | *UNC45B* |
| 10054 | *UNC50* |
| 10055 | *UNC5A* |
| 10056 | *UNC5B* |
| 10057 | *UNC5C* |
| 10058 | *UNC5CL* |
| 10059 | *UNC5D* |
| 10060 | *UNC93A* |
| 10061 | *UNG* |
| 10062 | *UPB1* |
| 10063 | *UPF1* |
| 10064 | *UPF2* |
| 10065 | *UPF3A* |
| 10066 | *UPF3B* |
| 10067 | *UPK1A* |
| 10068 | *UPK2* |
| 10069 | *UPK3A* |
| 10070 | *UPP1* |
| 10071 | *UPP2* |
| 10072 | *UPRT* |
| 10073 | *UQCC1* |
| 10074 | *UQCR10* |
| 10075 | *UQCRB* |
| 10076 | *UQCRC1* |
| 10077 | *UQCRC2* |
| 10078 | *UQCRFS1* |
| 10079 | *UQCRFS1P1* |
| 10080 | *UQCRH* |
| 10081 | *UQCRQ* |
| 10082 | *URB1* |
| 10083 | *URGCP* |
| 10084 | *URI1* |
| 10085 | *UROD* |
| 10086 | *USE1* |
| 10087 | *USF1* |
| 10088 | *USF2* |
| 10089 | *USF3* |
| 10090 | *USH1C* |
| 10091 | *USH2A* |
| 10092 | *USHBP1* |
| 10093 | *USP1* |
| 10094 | *USP10* |
| 10095 | *USP11* |
| 10096 | *USP12* |
| 10097 | *USP13* |
| 10098 | *USP14* |
| 10099 | *USP15* |
| 10100 | *USP17L2* |
| 10101 | *USP18* |
| 10102 | *USP19* |
| 10103 | *USP2* |
| 10104 | *USP20* |
| 10105 | *USP21* |
| 10106 | *USP22* |
| 10107 | *USP24* |
| 10108 | *USP25* |
| 10109 | *USP27X* |
| 10110 | *USP28* |
| 10111 | *USP29* |
| 10112 | *USP3* |
| 10113 | *USP30* |
| 10114 | *USP33* |
| 10115 | *USP34* |
| 10116 | *USP36* |
| 10117 | *USP37* |
| 10118 | *USP38* |
| 10119 | *USP39* |
| 10120 | *USP4* |
| 10121 | *USP43* |
| 10122 | *USP44* |
| 10123 | *USP46* |
| 10124 | *USP47* |
| 10125 | *USP49* |
| 10126 | *USP5* |
| 10127 | *USP51* |
| 10128 | *USP54* |
| 10129 | *USP6* |
| 10130 | *USP6NL* |
| 10131 | *USP7* |
| 10132 | *USP8* |
| 10133 | *USP9X* |
| 10134 | *USP9Y* |
| 10135 | *UTP11* |
| 10136 | *UTP14A* |
| 10137 | *UTP15* |
| 10138 | *UTP18* |
| 10139 | *UTP20* |
| 10140 | *UTP23* |
| 10141 | *UTP25* |
| 10142 | *UTP4* |
| 10143 | *UTRN* |
| 10144 | *UTS2* |
| 10145 | *UTS2R* |
| 10146 | *UVRAG* |
| 10147 | *UXT* |
| 10148 | *UXT-AS1* |
| 10149 | *VAC14* |
| 10150 | *VAMP1* |
| 10151 | *VAMP2* |
| 10152 | *VAMP5* |
| 10153 | *VAMP8* |
| 10154 | *VANGL1* |
| 10155 | *VANGL2* |
| 10156 | *VAPA* |
| 10157 | *VAPB* |
| 10158 | *VARS1* |
| 10159 | *VARS2* |
| 10160 | *VASH1* |
| 10161 | *VASP* |
| 10162 | *VAT1* |
| 10163 | *VAV1* |
| 10164 | *VAV2* |
| 10165 | *VAV3* |
| 10166 | *VBP1* |
| 10167 | *VCAM1* |
| 10168 | *VCAN* |
| 10169 | *VCL* |
| 10170 | *VCP* |
| 10171 | *VDAC1* |
| 10172 | *VDAC2* |
| 10173 | *VDR* |
| 10174 | *VEGFA* |
| 10175 | *VEGFB* |
| 10176 | *VEGFC* |
| 10177 | *VEGFD* |
| 10178 | *VGLL4* |
| 10179 | *VHL* |
| 10180 | *VIL1* |
| 10181 | *VILL* |
| 10182 | *VIM* |
| 10183 | *VIM-AS1* |
| 10184 | *VIP* |
| 10185 | *VIPR1* |
| 10186 | *VIRMA* |
| 10187 | *VLDLR* |
| 10188 | *VMP1* |
| 10189 | *VN1R17P* |
| 10190 | *VN1R86P* |
| 10191 | *VNN1* |
| 10192 | *VPS11* |
| 10193 | *VPS13A* |
| 10194 | *VPS13B* |
| 10195 | *VPS16* |
| 10196 | *VPS18* |
| 10197 | *VPS26C* |
| 10198 | *VPS28* |
| 10199 | *VPS29* |
| 10200 | *VPS33B* |
| 10201 | *VPS35* |
| 10202 | *VPS37A* |
| 10203 | *VPS37B* |
| 10204 | *VPS41* |
| 10205 | *VPS4A* |
| 10206 | *VPS4B* |
| 10207 | *VPS51* |
| 10208 | *VPS52* |
| 10209 | *VPS53* |
| 10210 | *VPS72* |
| 10211 | *VPS8* |
| 10212 | *VPS9D1-AS1* |
| 10213 | *VRK3* |
| 10214 | *VRTN* |
| 10215 | *VSIG4* |
| 10216 | *VSIR* |
| 10217 | *VSNL1* |
| 10218 | *VSTM2A* |
| 10219 | *VSTM2L* |
| 10220 | *VSTM4* |
| 10221 | *VSX2* |
| 10222 | *VTA1* |
| 10223 | *VTCN1* |
| 10224 | *VTI1A* |
| 10225 | *VTN* |
| 10226 | *VWA1* |
| 10227 | *VWA2* |
| 10228 | *VWA8* |
| 10229 | *VWF* |
| 10230 | *WAC* |
| 10231 | *WARS1* |
| 10232 | *WASF1* |
| 10233 | *WASF2* |
| 10234 | *WASF3* |
| 10235 | *WASHC4* |
| 10236 | *WASHC5* |
| 10237 | *WASL* |
| 10238 | *WBP1L* |
| 10239 | *WBP4* |
| 10240 | *WDCP* |
| 10241 | *WDR1* |
| 10242 | *WDR11* |
| 10243 | *WDR13* |
| 10244 | *WDR19* |
| 10245 | *WDR20* |
| 10246 | *WDR24* |
| 10247 | *WDR26* |
| 10248 | *WDR36* |
| 10249 | *WDR43* |
| 10250 | *WDR45* |
| 10251 | *WDR46* |
| 10252 | *WDR47* |
| 10253 | *WDR48* |
| 10254 | *WDR49* |
| 10255 | *WDR5* |
| 10256 | *WDR53* |
| 10257 | *WDR54* |
| 10258 | *WDR6* |
| 10259 | *WDR62* |
| 10260 | *WDR76* |
| 10261 | *WDR77* |
| 10262 | *WDR82* |
| 10263 | *WDR88* |
| 10264 | *WDR90* |
| 10265 | *WDR91* |
| 10266 | *WDR95P* |
| 10267 | *WDSUB1* |
| 10268 | *WDTC1* |
| 10269 | *WEE1* |
| 10270 | *WFDC1* |
| 10271 | *WFDC2* |
| 10272 | *WFDC21P* |
| 10273 | *WHRN* |
| 10274 | *WIF1* |
| 10275 | *WIPF1* |
| 10276 | *WIPF2* |
| 10277 | *WIPF3* |
| 10278 | *WLS* |
| 10279 | *WNK1* |
| 10280 | *WNK2* |
| 10281 | *WNT1* |
| 10282 | *WNT10A* |
| 10283 | *WNT10B* |
| 10284 | *WNT11* |
| 10285 | *WNT16* |
| 10286 | *WNT2* |
| 10287 | *WNT2B* |
| 10288 | *WNT3* |
| 10289 | *WNT3A* |
| 10290 | *WNT4* |
| 10291 | *WNT5A* |
| 10292 | *WNT5B* |
| 10293 | *WNT6* |
| 10294 | *WNT7A* |
| 10295 | *WNT7B* |
| 10296 | *WNT8A* |
| 10297 | *WNT8B* |
| 10298 | *WNT9A* |
| 10299 | *WNT9B* |
| 10300 | *WRAP53* |
| 10301 | *WRN* |
| 10302 | *WRNIP1* |
| 10303 | *WSB2* |
| 10304 | *WSPAR* |
| 10305 | *WT1* |
| 10306 | *WTAP* |
| 10307 | *WWC1* |
| 10308 | *WWC2* |
| 10309 | *WWOX* |
| 10310 | *WWP1* |
| 10311 | *WWP2* |
| 10312 | *WWTR1* |
| 10313 | *XAF1* |
| 10314 | *XAGE1A* |
| 10315 | *XAGE1B* |
| 10316 | *XBP1* |
| 10317 | *XBP1P1* |
| 10318 | *XCL1* |
| 10319 | *XCR1* |
| 10320 | *XDH* |
| 10321 | *XIAP* |
| 10322 | *XIAP-AS1* |
| 10323 | *XIRP1* |
| 10324 | *XIST* |
| 10325 | *XK* |
| 10326 | *XKR5* |
| 10327 | *XPA* |
| 10328 | *XPC* |
| 10329 | *XPNPEP3* |
| 10330 | *XPO1* |
| 10331 | *XPO5* |
| 10332 | *XPO7* |
| 10333 | *XPOT* |
| 10334 | *XPR1* |
| 10335 | *XRCC1* |
| 10336 | *XRCC2* |
| 10337 | *XRCC3* |
| 10338 | *XRCC4* |
| 10339 | *XRCC5* |
| 10340 | *XRCC6* |
| 10341 | *XRCC6P5* |
| 10342 | *XRN2* |
| 10343 | *XRRA1* |
| 10344 | *YAP1* |
| 10345 | *YARS2* |
| 10346 | *YBX1* |
| 10347 | *YBX2* |
| 10348 | *YBX3* |
| 10349 | *YEATS4* |
| 10350 | *YES1* |
| 10351 | *YIF1A* |
| 10352 | *YIPF1* |
| 10353 | *YME1L1* |
| 10354 | *YPEL3* |
| 10355 | *YPEL5* |
| 10356 | *YRDC* |
| 10357 | *YTHDC2* |
| 10358 | *YTHDF1* |
| 10359 | *YTHDF2* |
| 10360 | *YTHDF3* |
| 10361 | *YWHAB* |
| 10362 | *YWHAE* |
| 10363 | *YWHAEP7* |
| 10364 | *YWHAG* |
| 10365 | *YWHAH* |
| 10366 | *YWHAQ* |
| 10367 | *YWHAQP1* |
| 10368 | *YWHAZ* |
| 10369 | *YY1* |
| 10370 | *YY2* |
| 10371 | *ZACN* |
| 10372 | *ZAP70* |
| 10373 | *ZBED1* |
| 10374 | *ZBED6* |
| 10375 | *ZBTB14* |
| 10376 | *ZBTB16* |
| 10377 | *ZBTB17* |
| 10378 | *ZBTB18* |
| 10379 | *ZBTB2* |
| 10380 | *ZBTB20* |
| 10381 | *ZBTB21* |
| 10382 | *ZBTB24* |
| 10383 | *ZBTB3* |
| 10384 | *ZBTB33* |
| 10385 | *ZBTB38* |
| 10386 | *ZBTB40* |
| 10387 | *ZBTB44* |
| 10388 | *ZBTB48* |
| 10389 | *ZBTB7A* |
| 10390 | *ZBTB7B* |
| 10391 | *ZBTB7C* |
| 10392 | *ZBTB8A* |
| 10393 | *ZBTB8B* |
| 10394 | *ZC3H13* |
| 10395 | *ZC3H14* |
| 10396 | *ZC3H18* |
| 10397 | *ZC3HAV1* |
| 10398 | *ZC3HC1* |
| 10399 | *ZCCHC10* |
| 10400 | *ZCCHC14* |
| 10401 | *ZCCHC3* |
| 10402 | *ZCWPW2* |
| 10403 | *ZDHHC1* |
| 10404 | *ZDHHC12* |
| 10405 | *ZDHHC2* |
| 10406 | *ZDHHC4* |
| 10407 | *ZDHHC6* |
| 10408 | *ZDHHC7* |
| 10409 | *ZDHHC8BP* |
| 10410 | *ZDHHC8P1* |
| 10411 | *ZDHHC9* |
| 10412 | *ZEB1* |
| 10413 | *ZEB1-AS1* |
| 10414 | *ZEB2* |
| 10415 | *ZER1* |
| 10416 | *ZFAND1* |
| 10417 | *ZFAND2A* |
| 10418 | *ZFAS1* |
| 10419 | *ZFAT* |
| 10420 | *ZFC3H1* |
| 10421 | *ZFHX3* |
| 10422 | *ZFP30* |
| 10423 | *ZFP36* |
| 10424 | *ZFP36L1* |
| 10425 | *ZFP36L2* |
| 10426 | *ZFP57* |
| 10427 | *ZFP64* |
| 10428 | *ZFP82* |
| 10429 | *ZFP90* |
| 10430 | *ZFP91* |
| 10431 | *ZFPL1* |
| 10432 | *ZFR* |
| 10433 | *ZFX* |
| 10434 | *ZFYVE26* |
| 10435 | *ZFYVE28* |
| 10436 | *ZFYVE9* |
| 10437 | *ZG16* |
| 10438 | *ZG16B* |
| 10439 | *ZGLP1* |
| 10440 | *ZGPAT* |
| 10441 | *ZHX2* |
| 10442 | *ZIC1* |
| 10443 | *ZIC2* |
| 10444 | *ZIC3* |
| 10445 | *ZIC5* |
| 10446 | *ZKSCAN3* |
| 10447 | *ZKSCAN8* |
| 10448 | *ZMAT2* |
| 10449 | *ZMAT3* |
| 10450 | *ZMIZ1* |
| 10451 | *ZMIZ1-AS1* |
| 10452 | *ZMIZ2* |
| 10453 | *ZMPSTE24* |
| 10454 | *ZMYM1* |
| 10455 | *ZMYM4* |
| 10456 | *ZMYND10* |
| 10457 | *ZMYND8* |
| 10458 | *ZNF100* |
| 10459 | *ZNF12* |
| 10460 | *ZNF137P* |
| 10461 | *ZNF142* |
| 10462 | *ZNF146* |
| 10463 | *ZNF148* |
| 10464 | *ZNF155* |
| 10465 | *ZNF160* |
| 10466 | *ZNF165* |
| 10467 | *ZNF169* |
| 10468 | *ZNF175* |
| 10469 | *ZNF185* |
| 10470 | *ZNF189* |
| 10471 | *ZNF205* |
| 10472 | *ZNF207* |
| 10473 | *ZNF217* |
| 10474 | *ZNF22* |
| 10475 | *ZNF232* |
| 10476 | *ZNF236* |
| 10477 | *ZNF24* |
| 10478 | *ZNF25* |
| 10479 | *ZNF277* |
| 10480 | *ZNF28* |
| 10481 | *ZNF280A* |
| 10482 | *ZNF280C* |
| 10483 | *ZNF281* |
| 10484 | *ZNF292* |
| 10485 | *ZNF300* |
| 10486 | *ZNF300P1* |
| 10487 | *ZNF304* |
| 10488 | *ZNF318* |
| 10489 | *ZNF32* |
| 10490 | *ZNF326* |
| 10491 | *ZNF331* |
| 10492 | *ZNF346* |
| 10493 | *ZNF347* |
| 10494 | *ZNF350* |
| 10495 | *ZNF367* |
| 10496 | *ZNF37A* |
| 10497 | *ZNF384* |
| 10498 | *ZNF385D* |
| 10499 | *ZNF398* |
| 10500 | *ZNF407* |
| 10501 | *ZNF410* |
| 10502 | *ZNF420* |
| 10503 | *ZNF429* |
| 10504 | *ZNF43* |
| 10505 | *ZNF432* |
| 10506 | *ZNF436* |
| 10507 | *ZNF438* |
| 10508 | *ZNF442* |
| 10509 | *ZNF468* |
| 10510 | *ZNF471* |
| 10511 | *ZNF48* |
| 10512 | *ZNF480* |
| 10513 | *ZNF492* |
| 10514 | *ZNF506* |
| 10515 | *ZNF507* |
| 10516 | *ZNF510* |
| 10517 | *ZNF516* |
| 10518 | *ZNF518B* |
| 10519 | *ZNF525* |
| 10520 | *ZNF526* |
| 10521 | *ZNF532* |
| 10522 | *ZNF540* |
| 10523 | *ZNF541* |
| 10524 | *ZNF543* |
| 10525 | *ZNF546* |
| 10526 | *ZNF560* |
| 10527 | *ZNF563* |
| 10528 | *ZNF568* |
| 10529 | *ZNF569* |
| 10530 | *ZNF572* |
| 10531 | *ZNF573* |
| 10532 | *ZNF574* |
| 10533 | *ZNF577* |
| 10534 | *ZNF578* |
| 10535 | *ZNF582-AS1* |
| 10536 | *ZNF583* |
| 10537 | *ZNF598* |
| 10538 | *ZNF600* |
| 10539 | *ZNF606* |
| 10540 | *ZNF609* |
| 10541 | *ZNF611* |
| 10542 | *ZNF613* |
| 10543 | *ZNF619* |
| 10544 | *ZNF619P1* |
| 10545 | *ZNF621* |
| 10546 | *ZNF624* |
| 10547 | *ZNF638* |
| 10548 | *ZNF644* |
| 10549 | *ZNF646* |
| 10550 | *ZNF654* |
| 10551 | *ZNF665* |
| 10552 | *ZNF668* |
| 10553 | *ZNF677* |
| 10554 | *ZNF692* |
| 10555 | *ZNF700* |
| 10556 | *ZNF701* |
| 10557 | *ZNF703* |
| 10558 | *ZNF708* |
| 10559 | *ZNF746* |
| 10560 | *ZNF750* |
| 10561 | *ZNF75A* |
| 10562 | *ZNF76* |
| 10563 | *ZNF761* |
| 10564 | *ZNF766* |
| 10565 | *ZNF767P* |
| 10566 | *ZNF768* |
| 10567 | *ZNF774* |
| 10568 | *ZNF785* |
| 10569 | *ZNF79* |
| 10570 | *ZNF804A* |
| 10571 | *ZNF808* |
| 10572 | *ZNF816* |
| 10573 | *ZNF818P* |
| 10574 | *ZNF827* |
| 10575 | *ZNF829* |
| 10576 | *ZNF83* |
| 10577 | *ZNF836* |
| 10578 | *ZNF839* |
| 10579 | *ZNF841* |
| 10580 | *ZNF845* |
| 10581 | *ZNF880* |
| 10582 | *ZNFX1* |
| 10583 | *ZNG1A* |
| 10584 | *ZNG1B* |
| 10585 | *ZNG1E* |
| 10586 | *ZNG1F* |
| 10587 | *ZNHIT1* |
| 10588 | *ZNHIT2* |
| 10589 | *ZNHIT3* |
| 10590 | *ZNHIT6* |
| 10591 | *ZNRD2* |
| 10592 | *ZNRF3* |
| 10593 | *ZP3* |
| 10594 | *ZP4* |
| 10595 | *ZPLD1* |
| 10596 | *ZRANB1* |
| 10597 | *ZRSR2P1* |
| 10598 | *ZSCAN16* |
| 10599 | *ZSCAN18* |
| 10600 | *ZSCAN32* |
| 10601 | *ZSCAN5A* |
| 10602 | *ZSWIM4* |
| 10603 | *ZSWIM7* |
| 10604 | *ZSWIM8* |
| 10605 | *ZWILCH* |
| 10606 | *ZWINT* |
| 10607 | *ZYX* |
| 10608 | *ZZZ3* |
