# Supplementary material for: Uncovering the anti-cancer mechanism of cucurbitacin D against colorectal cancer through network pharmacology and molecular docking
Source: Discov Oncol. 2025 Apr 17;16:551. doi: 10.1007/s12672-025-02056-7 (PMC12006582; doi:10.1007/s12672-025-02056-7)
Supplement: Supplementary file 6 — Additional file 6: Table S3. GO Terms and KEGG pathways associated with the key targets of CuD in CRC. [file 12672_2025_2056_MOESM6_ESM.docx]

**Table S3. GO Terms and KEGG pathways associated with the key targets of CuD in CRC**

| **ID** | **Term** | **Ontology**  **Source** | **Term PValue** | **FDR**  **(Term PValue Corrected with Benjamini-Hochberg)** | **% Associated Genes** | **Nr. Genes** | **Associated Genes Found** |
| --- | --- | --- | --- | --- | --- | --- | --- |
| KEGG:05200 | Pathways in cancer | KEGG | 4.93E-26 | 1.28E-23 | 5.08 | 27.00 | [AKT1, BCL2, BCL2L1, BMP2, CASP3, CASP7, CASP9, CCNA2, CCND1, CDK2, CDK4, EGF, EGFR, ERBB2, ESR1, GSK3B, GSTP1, HSP90B1, JAK2, MAPK8, MMP9, MTOR, MYC, PIK3CA, PIM1, STAT3, VEGFA] |
| KEGG:05417 | Lipid and atherosclerosis | KEGG | 4.14E-21 | 5.38E-19 | 8.37 | 18.00 | [AKT1, BCL2, BCL2L1, CASP1, CASP3, CASP7, CASP9, GSK3B, HSP90B1, IL18, JAK2, MAPK8, MMP9, NLRP3, NOS3, PIK3CA, PYCARD, STAT3] |
| KEGG:05215 | Prostate cancer | KEGG | 5.86E-20 | 3.81E-18 | 14.43 | 14.00 | [AKT1, BCL2, CASP9, CCND1, CDK2, EGF, EGFR, ERBB2, GSK3B, GSTP1, HSP90B1, MMP9, MTOR, PIK3CA] |
| KEGG:04151 | PI3K-Akt signaling pathway | KEGG | 5.12E-20 | 4.44E-18 | 5.65 | 20.00 | [AKT1, BCL2, BCL2L1, CASP9, CCND1, CDC37, CDK2, CDK4, EGF, EGFR, ERBB2, ERBB3, GSK3B, HSP90B1, JAK2, MTOR, MYC, NOS3, PIK3CA, VEGFA] |
| KEGG:05212 | Pancreatic cancer | KEGG | 1.31E-19 | 6.82E-18 | 17.11 | 13.00 | [AKT1, BCL2L1, CASP9, CCND1, CDK4, EGF, EGFR, ERBB2, MAPK8, MTOR, PIK3CA, STAT3, VEGFA] |
| KEGG:05210 | Colorectal cancer | KEGG | 4.91E-17 | 2.13E-15 | 13.95 | 12.00 | [AKT1, BCL2, CASP3, CASP9, CCND1, EGF, EGFR, GSK3B, MAPK8, MTOR, MYC, PIK3CA] |
| KEGG:04933 | AGE-RAGE signaling pathway in diabetic complications | KEGG | 3.26E-16 | 1.21E-14 | 12.00 | 12.00 | [AKT1, BCL2, CASP3, CCND1, CDK4, JAK2, MAPK8, NOS3, PIK3CA, PIM1, STAT3, VEGFA] |
| GO:0033002 | muscle cell proliferation | GO_BP | 2.56E-15 | 8.33E-14 | 6.86 | 14.00 | [AKT1, ANG, CDK1, EGFR, GSTP1, IL10, IL18, JAK2, MMP9, MTOR, MYC, NQO2, PIM1, STAT3] |
| KEGG:05161 | Hepatitis B | KEGG | 3.63E-15 | 1.05E-13 | 8.02 | 13.00 | [AKT1, BCL2, CASP3, CASP9, CCNA2, CDK2, JAK2, MAPK8, MMP9, MYC, PCNA, PIK3CA, STAT3] |
| KEGG:04630 | JAK-STAT signaling pathway | KEGG | 3.63E-15 | 1.05E-13 | 8.02 | 13.00 | [AKT1, BCL2, BCL2L1, CCND1, EGF, EGFR, IL10, JAK2, MTOR, MYC, PIK3CA, PIM1, STAT3] |
| KEGG:05162 | Measles | KEGG | 1.88E-14 | 4.90E-13 | 8.63 | 12.00 | [AKT1, BCL2, BCL2L1, CASP3, CASP9, CCND1, CDK2, CDK4, GSK3B, MAPK8, PIK3CA, STAT3] |
| GO:0062197 | cellular response to chemical stress | GO_BP | 2.61E-14 | 6.16E-13 | 4.36 | 16.00 | [AKT1, BCL2, CASP3, CDK1, EGFR, GSTP1, IL10, JAK2, MAP1LC3A, MAPK8, MMP9, MYC, NOS3, PCNA, PIK3CA, PPIA] |
| KEGG:05167 | Kaposi sarcoma-associated herpesvirus infection | KEGG | 3.56E-14 | 7.72E-13 | 6.74 | 13.00 | [AKT1, CASP3, CASP9, CCND1, CDK4, GSK3B, JAK2, MAPK8, MTOR, MYC, PIK3CA, STAT3, VEGFA] |
| KEGG:05169 | Epstein-Barr virus infection | KEGG | 6.42E-14 | 1.28E-12 | 6.44 | 13.00 | [AKT1, BCL2, CASP3, CASP9, CCNA2, CCND1, CDK2, CDK4, ITGAL, MAPK8, MYC, PIK3CA, STAT3] |
| KEGG:05160 | Hepatitis C | KEGG | 8.23E-14 | 1.43E-12 | 7.64 | 12.00 | [AKT1, CASP3, CASP9, CCND1, CDK2, CDK4, EGF, EGFR, GSK3B, MYC, PIK3CA, STAT3] |
| KEGG:05205 | Proteoglycans in cancer | KEGG | 7.77E-14 | 1.44E-12 | 6.34 | 13.00 | [AKT1, CASP3, CCND1, EGFR, ERBB2, ERBB3, ESR1, MMP9, MTOR, MYC, PIK3CA, STAT3, VEGFA] |
| KEGG:04012 | ErbB signaling pathway | KEGG | 1.39E-13 | 2.27E-12 | 11.76 | 10.00 | [AKT1, EGF, EGFR, ERBB2, ERBB3, GSK3B, MAPK8, MTOR, MYC, PIK3CA] |
| KEGG:05213 | Endometrial cancer | KEGG | 1.84E-13 | 2.81E-12 | 15.52 | 9.00 | [AKT1, CASP9, CCND1, EGF, EGFR, ERBB2, GSK3B, MYC, PIK3CA] |
| KEGG:05222 | Small cell lung cancer | KEGG | 3.16E-13 | 4.56E-12 | 10.87 | 10.00 | [AKT1, BCL2, BCL2L1, CASP3, CASP9, CCND1, CDK2, CDK4, MYC, PIK3CA] |
| KEGG:05219 | Bladder cancer | KEGG | 6.13E-13 | 8.38E-12 | 19.51 | 8.00 | [CCND1, CDK4, EGF, EGFR, ERBB2, MMP9, MYC, VEGFA] |
| KEGG:05206 | MicroRNAs in cancer | KEGG | 8.19E-13 | 1.06E-11 | 4.52 | 14.00 | [BCL2, CASP3, CCND1, EGFR, ERBB2, ERBB3, MIR145, MMP9, MTOR, MYC, PIK3CA, PIM1, STAT3, VEGFA] |
| GO:0048660 | regulation of smooth muscle cell proliferation | GO_BP | 1.06E-12 | 1.31E-11 | 7.59 | 11.00 | [AKT1, ANG, EGFR, GSTP1, IL10, IL18, JAK2, MMP9, MTOR, MYC, NQO2] |
| KEGG:05224 | Breast cancer | KEGG | 1.23E-12 | 1.45E-11 | 7.48 | 11.00 | [AKT1, CCND1, CDK4, EGF, EGFR, ERBB2, ESR1, GSK3B, MTOR, MYC, PIK3CA] |
| KEGG:05226 | Gastric cancer | KEGG | 1.43E-12 | 1.48E-11 | 7.38 | 11.00 | [AKT1, BCL2, CCND1, CDK2, EGF, EGFR, ERBB2, GSK3B, MTOR, MYC, PIK3CA] |
| GO:0048659 | smooth muscle cell proliferation | GO_BP | 1.32E-12 | 1.50E-11 | 7.43 | 11.00 | [AKT1, ANG, EGFR, GSTP1, IL10, IL18, JAK2, MMP9, MTOR, MYC, NQO2] |
| KEGG:05223 | Non-small cell lung cancer | KEGG | 1.42E-12 | 1.54E-11 | 12.50 | 9.00 | [AKT1, CASP9, CCND1, CDK4, EGF, EGFR, ERBB2, PIK3CA, STAT3] |
| KEGG:04115 | p53 signaling pathway | KEGG | 1.61E-12 | 1.61E-11 | 12.33 | 9.00 | [BCL2, BCL2L1, CASP3, CASP9, CCND1, CDK1, CDK2, CDK4, RRM2] |
| KEGG:04066 | HIF-1 signaling pathway | KEGG | 1.79E-12 | 1.73E-11 | 9.17 | 10.00 | [AKT1, BCL2, EGF, EGFR, ERBB2, MTOR, NOS3, PIK3CA, STAT3, VEGFA] |
| GO:0050679 | positive regulation of epithelial cell proliferation | GO_BP | 2.97E-12 | 2.66E-11 | 5.66 | 12.00 | [AKT1, ANG, BMP2, CCND1, EGF, EGFR, ERBB2, IL10, MTOR, MYC, STAT3, VEGFA] |
| GO:0032355 | response to estradiol | GO_BP | 2.91E-12 | 2.71E-11 | 6.92 | 11.00 | [CASP3, CASP9, CCNA2, CCND1, EGFR, ESR1, GSTP1, IL10, MYC, PCNA, STAT3] |
| GO:0009411 | response to UV | GO_BP | 3.58E-12 | 3.10E-11 | 6.79 | 11.00 | [AKT1, BCL2, CASP3, CASP7, CASP9, CCND1, EGFR, MMP9, MYC, PCNA, TYR] |
| KEGG:05163 | Human cytomegalovirus infection | KEGG | 5.99E-12 | 5.02E-11 | 5.33 | 12.00 | [AKT1, CASP3, CASP9, CCND1, CDK4, EGFR, GSK3B, MTOR, MYC, PIK3CA, STAT3, VEGFA] |
| GO:0000302 | response to reactive oxygen species | GO_BP | 1.10E-11 | 8.95E-11 | 5.06 | 12.00 | [AKT1, BCL2, CASP3, CDK1, EGFR, GSTP1, IL10, MAP1LC3A, MAPK8, MMP9, NOS3, PCNA] |
| GO:0048661 | positive regulation of smooth muscle cell proliferation | GO_BP | 1.25E-11 | 9.82E-11 | 9.89 | 9.00 | [AKT1, EGFR, IL10, IL18, JAK2, MMP9, MTOR, MYC, NQO2] |
| GO:0031100 | animal organ regeneration | GO_BP | 1.25E-11 | 9.82E-11 | 9.89 | 9.00 | [CCNA2, CCND1, CDK1, CDK4, EGFR, GSTP1, IL10, MYC, PCNA] |
| GO:0097153 | cysteine-type endopeptidase activity involved in apoptotic process | GO_BP | 1.48E-11 | 1.13E-10 | 4.94 | 12.00 | [AKT1, CASP1, CASP3, CASP7, CASP9, CCNA2, JAK2, MMP9, MYC, NLRP3, PYCARD, VEGFA] |
| KEGG:05131 | Shigellosis | KEGG | 1.70E-11 | 1.23E-10 | 4.88 | 12.00 | [AKT1, BCL2, BCL2L1, CASP1, EGFR, GSK3B, IL18, MAPK8, MTOR, NLRP3, PIK3CA, PYCARD] |
| GO:0034599 | cellular response to oxidative stress | GO_BP | 1.69E-11 | 1.25E-10 | 4.15 | 13.00 | [AKT1, BCL2, CDK1, EGFR, GSTP1, IL10, JAK2, MAP1LC3A, MAPK8, MMP9, NOS3, PCNA, PPIA] |
| KEGG:05135 | Yersinia infection | KEGG | 1.80E-11 | 1.26E-10 | 7.30 | 10.00 | [AKT1, CASP1, CD4, GSK3B, IL10, IL18, MAPK8, NLRP3, PIK3CA, PYCARD] |
| KEGG:05132 | Salmonella infection | KEGG | 1.96E-11 | 1.34E-10 | 4.82 | 12.00 | [AKT1, BCL2, CASP1, CASP3, CASP7, HSP90B1, IL18, MAPK8, MYC, NLRP3, PIK3CA, PYCARD] |
| KEGG:05221 | Acute myeloid leukemia | KEGG | 3.92E-11 | 2.61E-10 | 11.94 | 8.00 | [AKT1, CCNA2, CCND1, MTOR, MYC, PIK3CA, PIM1, STAT3] |
| KEGG:04917 | Prolactin signaling pathway | KEGG | 5.63E-11 | 3.66E-10 | 11.43 | 8.00 | [AKT1, CCND1, ESR1, GSK3B, JAK2, MAPK8, PIK3CA, STAT3] |
| KEGG:05145 | Toxoplasmosis | KEGG | 8.30E-11 | 5.26E-10 | 8.04 | 9.00 | [AKT1, BCL2, BCL2L1, CASP3, CASP9, IL10, JAK2, MAPK8, STAT3] |
| KEGG:05166 | Human T-cell leukemia virus 1 infection | KEGG | 9.40E-11 | 5.82E-10 | 5.02 | 11.00 | [AKT1, BCL2L1, CCNA2, CCND1, CD4, CDK2, CDK4, ITGAL, MAPK8, MYC, PIK3CA] |
| GO:0031099 | regeneration | GO_BP | 1.09E-10 | 6.58E-10 | 4.95 | 11.00 | [BCL2, CCNA2, CCND1, CDK1, CDK4, EGFR, GSTP1, IL10, JAK2, MYC, PCNA] |
| KEGG:05225 | Hepatocellular carcinoma | KEGG | 1.36E-10 | 8.06E-10 | 5.95 | 10.00 | [AKT1, BCL2L1, CCND1, CDK4, EGFR, GSK3B, GSTP1, MTOR, MYC, PIK3CA] |
| KEGG:05164 | Influenza A | KEGG | 1.72E-10 | 9.94E-10 | 5.81 | 10.00 | [AKT1, CASP1, CASP3, CASP9, CDK4, IL18, JAK2, NLRP3, PIK3CA, PYCARD] |
| GO:0097191 | extrinsic apoptotic signaling pathway | GO_BP | 2.19E-10 | 1.24E-09 | 4.64 | 11.00 | [AKT1, BCL2, BCL2L1, CASP3, CASP9, ERBB3, GSK3B, GSTP1, JAK2, NOS3, PYCARD] |
| GO:0004693 | cyclin-dependent protein serine/threonine kinase activity | GO_BP | 2.97E-10 | 1.64E-09 | 6.98 | 9.00 | [AKT1, CASP3, CCNA2, CCND1, CDC37, CDK1, CDK2, CDK4, EGFR] |
| KEGG:04068 | FoxO signaling pathway | KEGG | 3.40E-10 | 1.84E-09 | 6.87 | 9.00 | [AKT1, CCND1, CDK2, EGF, EGFR, IL10, MAPK8, PIK3CA, STAT3] |
| GO:0097472 | cyclin-dependent protein kinase activity | GO_BP | 3.90E-10 | 2.07E-09 | 6.77 | 9.00 | [AKT1, CASP3, CCNA2, CCND1, CDC37, CDK1, CDK2, CDK4, EGFR] |
| KEGG:05235 | PD-L1 expression and PD-1 checkpoint pathway in cancer | KEGG | 4.02E-10 | 2.09E-09 | 8.99 | 8.00 | [AKT1, CD4, EGF, EGFR, JAK2, MTOR, PIK3CA, STAT3] |
| KEGG:05418 | Fluid shear stress and atherosclerosis | KEGG | 5.79E-10 | 2.89E-09 | 6.47 | 9.00 | [AKT1, BCL2, GSTP1, HSP90B1, MAPK8, MMP9, NOS3, PIK3CA, VEGFA] |
| KEGG:05134 | Legionellosis | KEGG | 5.70E-10 | 2.91E-09 | 12.28 | 7.00 | [CASP1, CASP3, CASP7, CASP9, IL18, PYCARD, SAR1B] |
| GO:0044843 | cell cycle G1/S phase transition | GO_BP | 6.87E-10 | 3.37E-09 | 4.17 | 11.00 | [AKT1, BCL2, CCNA2, CCND1, CDK1, CDK2, CDK4, EGFR, MYC, PCNA, RRM2] |
| KEGG:04215 | Apoptosis | KEGG | 7.15E-10 | 3.44E-09 | 18.75 | 6.00 | [BCL2, BCL2L1, CASP3, CASP7, CASP9, MAPK8] |
| KEGG:04510 | Focal adhesion | KEGG | 7.91E-10 | 3.74E-09 | 4.98 | 10.00 | [AKT1, BCL2, CCND1, EGF, EGFR, ERBB2, GSK3B, MAPK8, PIK3CA, VEGFA] |
| GO:0038128 | ERBB2 signaling pathway | GO_BP | 8.72E-10 | 4.05E-09 | 18.18 | 6.00 | [CDC37, EGF, EGFR, ERBB2, ERBB3, PIK3CA] |
| KEGG:05170 | Human immunodeficiency virus 1 infection | KEGG | 1.33E-09 | 5.95E-09 | 4.72 | 10.00 | [AKT1, BCL2, BCL2L1, CASP3, CASP9, CD4, CDK1, MAPK8, MTOR, PIK3CA] |
| GO:0001938 | positive regulation of endothelial cell proliferation | GO_BP | 1.31E-09 | 5.96E-09 | 7.77 | 8.00 | [AKT1, ANG, BMP2, EGF, IL10, MTOR, STAT3, VEGFA] |
| KEGG:04218 | Cellular senescence | KEGG | 1.61E-09 | 7.11E-09 | 5.77 | 9.00 | [AKT1, CCNA2, CCND1, CDK1, CDK2, CDK4, MTOR, MYC, PIK3CA] |
| GO:0034614 | cellular response to reactive oxygen species | GO_BP | 2.38E-09 | 1.03E-08 | 5.52 | 9.00 | [AKT1, CDK1, EGFR, IL10, MAP1LC3A, MAPK8, MMP9, NOS3, PCNA] |
| GO:0097192 | extrinsic apoptotic signaling pathway in absence of ligand | GO_BP | 2.50E-09 | 1.07E-08 | 10.00 | 7.00 | [AKT1, BCL2, BCL2L1, CASP3, CASP9, ERBB3, GSK3B] |
| GO:0038034 | signal transduction in absence of ligand | GO_BP | 2.50E-09 | 1.07E-08 | 10.00 | 7.00 | [AKT1, BCL2, BCL2L1, CASP3, CASP9, ERBB3, GSK3B] |
| GO:0043281 | regulation of cysteine-type endopeptidase activity involved in apoptotic process | GO_BP | 3.89E-09 | 1.63E-08 | 4.22 | 10.00 | [AKT1, CASP1, CASP9, CCNA2, JAK2, MMP9, MYC, NLRP3, PYCARD, VEGFA] |
| KEGG:05214 | Glioma | KEGG | 4.09E-09 | 1.69E-08 | 9.33 | 7.00 | [AKT1, CCND1, CDK4, EGF, EGFR, MTOR, PIK3CA] |
| GO:0000082 | G1/S transition of mitotic cell cycle | GO_BP | 4.39E-09 | 1.79E-08 | 4.17 | 10.00 | [AKT1, BCL2, CCND1, CDK1, CDK2, CDK4, EGFR, MYC, PCNA, RRM2] |
| KEGG:05133 | Pertussis | KEGG | 4.49E-09 | 1.80E-08 | 9.21 | 7.00 | [CASP1, CASP3, CASP7, IL10, MAPK8, NLRP3, PYCARD] |
| KEGG:04919 | Thyroid hormone signaling pathway | KEGG | 4.73E-09 | 1.83E-08 | 6.61 | 8.00 | [AKT1, CASP9, CCND1, ESR1, GSK3B, MTOR, MYC, PIK3CA] |
| GO:0000307 | cyclin-dependent protein kinase holoenzyme complex | GO_CC | 4.68E-09 | 1.85E-08 | 13.95 | 6.00 | [CCNA2, CCND1, CDK1, CDK2, CDK4, PCNA] |
| KEGG:04110 | Cell cycle | KEGG | 5.74E-09 | 2.19E-08 | 6.45 | 8.00 | [CCNA2, CCND1, CDK1, CDK2, CDK4, GSK3B, MYC, PCNA] |
| KEGG:04926 | Relaxin signaling pathway | KEGG | 7.85E-09 | 2.96E-08 | 6.20 | 8.00 | [AKT1, EGFR, MAPK8, MMP13, MMP9, NOS3, PIK3CA, VEGFA] |
| GO:0008637 | apoptotic mitochondrial changes | GO_BP | 9.41E-09 | 3.49E-08 | 6.06 | 8.00 | [AKT1, BCL2, BCL2L1, GSK3B, MAPK8, MMP9, NLRP3, PYCARD] |
| KEGG:04210 | Apoptosis | KEGG | 1.19E-08 | 4.36E-08 | 5.88 | 8.00 | [AKT1, BCL2, BCL2L1, CASP3, CASP7, CASP9, MAPK8, PIK3CA] |
| GO:1902554 | serine/threonine protein kinase complex | GO_CC | 1.27E-08 | 4.57E-08 | 7.95 | 7.00 | [CCNA2, CCND1, CDK1, CDK2, CDK4, PCNA, PYCARD] |
| KEGG:04915 | Estrogen signaling pathway | KEGG | 1.34E-08 | 4.76E-08 | 5.80 | 8.00 | [AKT1, BCL2, EGFR, ESR1, HSP90B1, MMP9, NOS3, PIK3CA] |
| KEGG:05203 | Viral carcinogenesis | KEGG | 1.69E-08 | 5.95E-08 | 4.41 | 9.00 | [CASP3, CCNA2, CCND1, CDK1, CDK2, CDK4, HDAC8, PIK3CA, STAT3] |
| GO:0071222 | cellular response to lipopolysaccharide | GO_BP | 1.69E-08 | 5.95E-08 | 4.41 | 9.00 | [AKT1, CASP1, CDK4, GSTP1, IL10, IL18, NLRP3, PDE4B, PYCARD] |
| GO:0001936 | regulation of endothelial cell proliferation | GO_BP | 2.19E-08 | 7.50E-08 | 5.44 | 8.00 | [AKT1, ANG, BMP2, EGF, IL10, MTOR, STAT3, VEGFA] |
| GO:0097199 | cysteine-type endopeptidase activity involved in apoptotic signaling pathway | GO_BP | 2.18E-08 | 7.57E-08 | 18.52 | 5.00 | [CASP1, CASP3, CASP9, JAK2, MMP9] |
| GO:0001101 | response to acid chemical | GO_BP | 2.31E-08 | 7.81E-08 | 5.41 | 8.00 | [BCL2L1, CASP3, GSTP1, MTOR, MYC, PCNA, PIK3CA, VEGFA] |
| GO:0038127 | ERBB signaling pathway | GO_BP | 2.57E-08 | 8.45E-08 | 5.33 | 8.00 | [AKT1, CDC37, EGF, EGFR, ERBB2, ERBB3, MMP9, PIK3CA] |
| GO:0071219 | cellular response to molecule of bacterial origin | GO_BP | 2.56E-08 | 8.54E-08 | 4.21 | 9.00 | [AKT1, CASP1, CDK4, GSTP1, IL10, IL18, NLRP3, PDE4B, PYCARD] |
| GO:0001541 | ovarian follicle development | GO_BP | 2.69E-08 | 8.75E-08 | 10.53 | 6.00 | [ANG, BCL2, BCL2L1, ESR1, MYC, VEGFA] |
| GO:0043523 | regulation of neuron apoptotic process | GO_BP | 3.13E-08 | 1.00E-07 | 4.11 | 9.00 | [BCL2, BCL2L1, CASP3, CASP9, ERBB3, IL10, JAK2, NQO2, PIK3CA] |
| GO:1901654 | response to ketone | GO_BP | 3.25E-08 | 1.03E-07 | 4.09 | 9.00 | [AKT1, BCL2L1, CASP9, CCND1, CDK4, EGFR, MYC, NLRP3, PCNA] |
| GO:1901215 | negative regulation of neuron death | GO_BP | 3.25E-08 | 1.03E-07 | 4.09 | 9.00 | [AKT1, BCL2, BCL2L1, ERBB3, GSK3B, IL10, JAK2, PIK3CA, STAT3] |
| GO:0008585 | female gonad development | GO_BP | 3.32E-08 | 1.04E-07 | 6.93 | 7.00 | [ANG, BCL2, BCL2L1, CASP3, ESR1, MYC, VEGFA] |
| GO:2000045 | regulation of G1/S transition of mitotic cell cycle | GO_BP | 3.85E-08 | 1.18E-07 | 5.06 | 8.00 | [AKT1, BCL2, CCND1, CDK1, CDK2, CDK4, EGFR, PCNA] |
| GO:1902911 | protein kinase complex | GO_CC | 3.81E-08 | 1.18E-07 | 6.80 | 7.00 | [CCNA2, CCND1, CDK1, CDK2, CDK4, PCNA, PYCARD] |
| KEGG:04660 | T cell receptor signaling pathway | KEGG | 4.07E-08 | 1.22E-07 | 6.73 | 7.00 | [AKT1, CD4, CDK4, GSK3B, IL10, MAPK8, PIK3CA] |
| KEGG:04625 | C-type lectin receptor signaling pathway | KEGG | 4.07E-08 | 1.22E-07 | 6.73 | 7.00 | [AKT1, CASP1, IL10, MAPK8, NLRP3, PIK3CA, PYCARD] |
| KEGG:04217 | Necroptosis | KEGG | 4.05E-08 | 1.22E-07 | 5.03 | 8.00 | [BCL2, CASP1, JAK2, MAPK8, NLRP3, PPIA, PYCARD, STAT3] |
| GO:0046545 | development of primary female sexual characteristics | GO_BP | 4.65E-08 | 1.37E-07 | 6.60 | 7.00 | [ANG, BCL2, BCL2L1, CASP3, ESR1, MYC, VEGFA] |
| GO:0001935 | endothelial cell proliferation | GO_BP | 4.91E-08 | 1.43E-07 | 4.91 | 8.00 | [AKT1, ANG, BMP2, EGF, IL10, MTOR, STAT3, VEGFA] |
| KEGG:04931 | Insulin resistance | KEGG | 5.29E-08 | 1.53E-07 | 6.48 | 7.00 | [AKT1, GSK3B, MAPK8, MTOR, NOS3, PIK3CA, STAT3] |
| GO:0010821 | regulation of mitochondrion organization | GO_BP | 5.40E-08 | 1.54E-07 | 4.85 | 8.00 | [AKT1, BCL2, BCL2L1, GSK3B, MAPK8, MMP9, NLRP3, PYCARD] |
| GO:0014065 | phosphatidylinositol 3-kinase signaling | GO_BP | 5.66E-08 | 1.60E-07 | 4.82 | 8.00 | [AKT1, EGF, EGFR, ERBB2, ERBB3, IL18, JAK2, PIK3CA] |
| GO:0001836 | release of cytochrome c from mitochondria | GO_BP | 7.22E-08 | 2.02E-07 | 8.96 | 6.00 | [AKT1, BCL2, BCL2L1, MMP9, NLRP3, PYCARD] |
| GO:0051384 | response to glucocorticoid | GO_BP | 8.90E-08 | 2.46E-07 | 4.55 | 8.00 | [BCL2, CASP3, CASP9, CCND1, EGFR, GSTP1, IL10, PCNA] |
| KEGG:05230 | Central carbon metabolism in cancer | KEGG | 9.42E-08 | 2.58E-07 | 8.57 | 6.00 | [AKT1, EGFR, ERBB2, MTOR, MYC, PIK3CA] |
| GO:0097421 | liver regeneration | GO_BP | 9.97E-08 | 2.70E-07 | 13.89 | 5.00 | [CCND1, EGFR, IL10, MYC, PCNA] |
| KEGG:04935 | Growth hormone synthesis, secretion and action | KEGG | 1.03E-07 | 2.77E-07 | 5.88 | 7.00 | [AKT1, GSK3B, JAK2, MAPK8, MTOR, PIK3CA, STAT3] |
| KEGG:05152 | Tuberculosis | KEGG | 1.06E-07 | 2.81E-07 | 4.44 | 8.00 | [AKT1, BCL2, CASP3, CASP9, IL10, IL18, JAK2, MAPK8] |
| GO:1902806 | regulation of cell cycle G1/S phase transition | GO_BP | 1.06E-07 | 2.81E-07 | 4.44 | 8.00 | [AKT1, BCL2, CCND1, CDK1, CDK2, CDK4, EGFR, PCNA] |
| GO:0032652 | regulation of interleukin-1 production | GO_BP | 1.10E-07 | 2.88E-07 | 5.83 | 7.00 | [CASP1, GSTP1, IL10, JAK2, NLRP3, PYCARD, STAT3] |
| GO:0032612 | interleukin-1 production | GO_BP | 1.10E-07 | 2.88E-07 | 5.83 | 7.00 | [CASP1, GSTP1, IL10, JAK2, NLRP3, PYCARD, STAT3] |
| KEGG:05218 | Melanoma | KEGG | 1.12E-07 | 2.90E-07 | 8.33 | 6.00 | [AKT1, CCND1, CDK4, EGF, EGFR, PIK3CA] |
| GO:0046660 | female sex differentiation | GO_BP | 1.16E-07 | 2.99E-07 | 5.79 | 7.00 | [ANG, BCL2, BCL2L1, CASP3, ESR1, MYC, VEGFA] |
| GO:0018107 | peptidyl-threonine phosphorylation | GO_BP | 1.37E-07 | 3.50E-07 | 5.65 | 7.00 | [AKT1, BCL2, CDK1, EGF, GSK3B, MAPK8, MTOR] |
| KEGG:05220 | Chronic myeloid leukemia | KEGG | 1.55E-07 | 3.91E-07 | 7.89 | 6.00 | [AKT1, BCL2L1, CCND1, CDK4, MYC, PIK3CA] |
| GO:0051897 | positive regulation of protein kinase B signaling | GO_BP | 1.60E-07 | 4.01E-07 | 4.21 | 8.00 | [EGF, EGFR, ERBB2, ERBB3, ESR1, IL18, MTOR, PIK3CA] |
| GO:0032872 | regulation of stress-activated MAPK cascade | GO_BP | 1.60E-07 | 4.01E-07 | 4.21 | 8.00 | [AKT1, BMP2, EGFR, GSTP1, MYC, PPIA, PYCARD, VEGFA] |
| GO:0070141 | response to UV-A | GO_BP | 1.68E-07 | 4.17E-07 | 25.00 | 4.00 | [AKT1, CCND1, EGFR, MMP9] |
| GO:0070302 | regulation of stress-activated protein kinase signaling cascade | GO_BP | 1.81E-07 | 4.44E-07 | 4.15 | 8.00 | [AKT1, BMP2, EGFR, GSTP1, MYC, PPIA, PYCARD, VEGFA] |
| GO:0022408 | negative regulation of cell-cell adhesion | GO_BP | 1.81E-07 | 4.44E-07 | 4.15 | 8.00 | [AKT1, BMP2, CASP3, ERBB2, IL10, JAK2, NF2, VEGFA] |
| GO:0010950 | positive regulation of endopeptidase activity | GO_BP | 1.88E-07 | 4.57E-07 | 4.12 | 8.00 | [CASP1, CASP9, CCNA2, JAK2, MYC, NLRP3, PYCARD, STAT3] |
| GO:0031960 | response to corticosteroid | GO_BP | 1.96E-07 | 4.71E-07 | 4.10 | 8.00 | [BCL2, CASP3, CASP9, CCND1, EGFR, GSTP1, IL10, PCNA] |
| KEGG:05130 | Pathogenic Escherichia coli infection | KEGG | 2.12E-07 | 5.05E-07 | 4.06 | 8.00 | [CASP1, CASP3, CASP7, CASP9, IL18, MAPK8, NLRP3, PYCARD] |
| GO:0018210 | peptidyl-threonine modification | GO_BP | 2.58E-07 | 6.11E-07 | 5.15 | 7.00 | [AKT1, BCL2, CDK1, EGF, GSK3B, MAPK8, MTOR] |
| GO:0046686 | response to cadmium ion | GO_BP | 2.63E-07 | 6.15E-07 | 7.23 | 6.00 | [AKT1, CDK1, EGFR, MAPK8, MMP9, PCNA] |
| GO:0097200 | cysteine-type endopeptidase activity involved in execution phase of apoptosis | GO_BP | 3.56E-07 | 8.26E-07 | 21.05 | 4.00 | [CASP1, CASP3, CASP7, CASP9] |
| GO:0061377 | mammary gland lobule development | GO_BP | 4.44E-07 | 1.02E-06 | 20.00 | 4.00 | [CCND1, EGF, ESR1, VEGFA] |
| GO:0060749 | mammary gland alveolus development | GO_BP | 4.44E-07 | 1.02E-06 | 20.00 | 4.00 | [CCND1, EGF, ESR1, VEGFA] |
| GO:0043280 | positive regulation of cysteine-type endopeptidase activity involved in apoptotic process | GO_BP | 4.59E-07 | 1.05E-06 | 4.73 | 7.00 | [CASP1, CASP9, CCNA2, JAK2, MYC, NLRP3, PYCARD] |
| KEGG:04657 | IL-17 signaling pathway | KEGG | 5.51E-07 | 1.25E-06 | 6.38 | 6.00 | [CASP3, GSK3B, HSP90B1, MAPK8, MMP13, MMP9] |
| GO:0010507 | negative regulation of autophagy | GO_BP | 5.51E-07 | 1.25E-06 | 6.38 | 6.00 | [AKT1, BCL2, IL10, MTOR, PIK3CA, STAT3] |
| GO:0071364 | cellular response to epidermal growth factor stimulus | GO_BP | 5.99E-07 | 1.34E-06 | 9.80 | 5.00 | [AKT1, EGFR, ERBB2, GSTP1, MYC] |
| GO:0090559 | regulation of membrane permeability | GO_BP | 6.24E-07 | 1.39E-06 | 6.25 | 6.00 | [BCL2, BCL2L1, GSK3B, MAPK8, MTOR, STAT3] |
| GO:0033135 | regulation of peptidyl-serine phosphorylation | GO_BP | 6.55E-07 | 1.44E-06 | 4.49 | 7.00 | [AKT1, BCL2, EGFR, NLRP3, PDE4D, PIK3CA, VEGFA] |
| KEGG:05231 | Choline metabolism in cancer | KEGG | 7.05E-07 | 1.54E-06 | 6.12 | 6.00 | [AKT1, EGF, EGFR, MAPK8, MTOR, PIK3CA] |
| GO:0090199 | regulation of release of cytochrome c from mitochondria | GO_BP | 8.01E-07 | 1.72E-06 | 9.26 | 5.00 | [AKT1, BCL2L1, MMP9, NLRP3, PYCARD] |
| KEGG:04914 | Progesterone-mediated oocyte maturation | KEGG | 7.95E-07 | 1.72E-06 | 6.00 | 6.00 | [AKT1, CCNA2, CDK1, CDK2, MAPK8, PIK3CA] |
| GO:0032651 | regulation of interleukin-1 beta production | GO_BP | 8.43E-07 | 1.80E-06 | 5.94 | 6.00 | [CASP1, GSTP1, JAK2, NLRP3, PYCARD, STAT3] |
| GO:0032611 | interleukin-1 beta production | GO_BP | 8.43E-07 | 1.80E-06 | 5.94 | 6.00 | [CASP1, GSTP1, JAK2, NLRP3, PYCARD, STAT3] |
| GO:0070849 | response to epidermal growth factor | GO_BP | 8.78E-07 | 1.86E-06 | 9.09 | 5.00 | [AKT1, EGFR, ERBB2, GSTP1, MYC] |
| GO:0000079 | regulation of cyclin-dependent protein serine/threonine kinase activity | GO_BP | 9.46E-07 | 1.98E-06 | 5.83 | 6.00 | [AKT1, CASP3, CCNA2, CCND1, CDC37, EGFR] |
| GO:2001056 | positive regulation of cysteine-type endopeptidase activity | GO_BP | 9.96E-07 | 2.07E-06 | 4.22 | 7.00 | [CASP1, CASP9, CCNA2, JAK2, MYC, NLRP3, PYCARD] |
| GO:1990874 | vascular associated smooth muscle cell proliferation | GO_BP | 1.05E-06 | 2.17E-06 | 8.77 | 5.00 | [GSTP1, IL10, JAK2, MMP9, NQO2] |
| GO:1904705 | regulation of vascular associated smooth muscle cell proliferation | GO_BP | 1.05E-06 | 2.17E-06 | 8.77 | 5.00 | [GSTP1, IL10, JAK2, MMP9, NQO2] |
| GO:0097194 | execution phase of apoptosis | GO_BP | 1.12E-06 | 2.29E-06 | 5.66 | 6.00 | [AKT1, BCL2L1, CASP1, CASP3, CASP7, CASP9] |
| GO:1904029 | regulation of cyclin-dependent protein kinase activity | GO_BP | 1.18E-06 | 2.41E-06 | 5.61 | 6.00 | [AKT1, CASP3, CCNA2, CCND1, CDC37, EGFR] |
| KEGG:04370 | VEGF signaling pathway | KEGG | 1.25E-06 | 2.52E-06 | 8.47 | 5.00 | [AKT1, CASP9, NOS3, PIK3CA, VEGFA] |
| GO:0007259 | receptor signaling pathway via JAK-STAT | GO_BP | 1.42E-06 | 2.84E-06 | 4.00 | 7.00 | [EGF, IL10, IL18, JAK2, NF2, STAT3, VEGFA] |
| KEGG:04668 | TNF signaling pathway | KEGG | 1.55E-06 | 3.07E-06 | 5.36 | 6.00 | [AKT1, CASP3, CASP7, MAPK8, MMP9, PIK3CA] |
| GO:2000134 | negative regulation of G1/S transition of mitotic cell cycle | GO_BP | 1.55E-06 | 3.07E-06 | 5.36 | 6.00 | [BCL2, CCND1, CDK1, CDK2, CDK4, PCNA] |
| GO:1902807 | negative regulation of cell cycle G1/S phase transition | GO_BP | 1.90E-06 | 3.74E-06 | 5.17 | 6.00 | [BCL2, CCND1, CDK1, CDK2, CDK4, PCNA] |
| GO:0033138 | positive regulation of peptidyl-serine phosphorylation | GO_BP | 2.10E-06 | 4.11E-06 | 5.08 | 6.00 | [AKT1, BCL2, EGFR, NLRP3, PIK3CA, VEGFA] |
| GO:0032731 | positive regulation of interleukin-1 beta production | GO_BP | 2.20E-06 | 4.26E-06 | 7.58 | 5.00 | [CASP1, JAK2, NLRP3, PYCARD, STAT3] |
| GO:0072559 | NLRP3 inflammasome complex | GO_CC | 2.59E-06 | 4.98E-06 | 33.33 | 3.00 | [CASP1, NLRP3, PYCARD] |
| GO:0030235 | nitric-oxide synthase regulator activity | GO_BP | 2.59E-06 | 4.98E-06 | 33.33 | 3.00 | [AKT1, EGFR, ESR1] |
| KEGG:04920 | Adipocytokine signaling pathway | KEGG | 2.74E-06 | 5.24E-06 | 7.25 | 5.00 | [AKT1, JAK2, MAPK8, MTOR, STAT3] |
| GO:0043279 | response to alkaloid | GO_BP | 3.08E-06 | 5.84E-06 | 4.76 | 6.00 | [BCL2L1, CASP3, CASP7, CCNA2, MAP1LC3A, MTOR] |
| GO:0051881 | regulation of mitochondrial membrane potential | GO_BP | 3.63E-06 | 6.84E-06 | 6.85 | 5.00 | [AKT1, BCL2, BCL2L1, CASP1, MYC] |
| GO:0043276 | anoikis | GO_BP | 4.63E-06 | 8.66E-06 | 11.43 | 4.00 | [AKT1, BCL2, MTOR, PIK3CA] |
| KEGG:04140 | Autophagy | KEGG | 5.00E-06 | 9.28E-06 | 4.38 | 6.00 | [AKT1, BCL2, BCL2L1, MAPK8, MTOR, PIK3CA] |
| GO:0014066 | regulation of phosphatidylinositol 3-kinase signaling | GO_BP | 5.21E-06 | 9.61E-06 | 4.35 | 6.00 | [EGF, EGFR, ERBB3, IL18, JAK2, PIK3CA] |
| GO:0071229 | cellular response to acid chemical | GO_BP | 5.71E-06 | 1.05E-05 | 6.25 | 5.00 | [BCL2L1, MTOR, MYC, PIK3CA, VEGFA] |
| GO:0032732 | positive regulation of interleukin-1 production | GO_BP | 5.71E-06 | 1.05E-05 | 6.25 | 5.00 | [CASP1, JAK2, NLRP3, PYCARD, STAT3] |
| GO:0006809 | nitric oxide biosynthetic process | GO_BP | 5.71E-06 | 1.05E-05 | 6.25 | 5.00 | [AKT1, IL10, JAK2, MTOR, NOS3] |
| GO:1904707 | positive regulation of vascular associated smooth muscle cell proliferation | GO_BP | 5.81E-06 | 1.06E-05 | 10.81 | 4.00 | [IL10, JAK2, MMP9, NQO2] |
| KEGG:04550 | Signaling pathways regulating pluripotency of stem cells | KEGG | 6.40E-06 | 1.16E-05 | 4.20 | 6.00 | [AKT1, GSK3B, JAK2, MYC, PIK3CA, STAT3] |
| GO:1901992 | positive regulation of mitotic cell cycle phase transition | GO_BP | 6.45E-06 | 1.16E-05 | 6.10 | 5.00 | [AKT1, CCND1, CDK1, CDK4, EGFR] |
| GO:0046902 | regulation of mitochondrial membrane permeability | GO_BP | 6.84E-06 | 1.22E-05 | 6.02 | 5.00 | [BCL2, BCL2L1, GSK3B, MAPK8, STAT3] |
| GO:0048145 | regulation of fibroblast proliferation | GO_BP | 7.69E-06 | 1.36E-05 | 5.88 | 5.00 | [CCNA2, CDK4, ESR1, GSTP1, MYC] |
| GO:0042509 | regulation of tyrosine phosphorylation of STAT protein | GO_BP | 7.69E-06 | 1.36E-05 | 5.88 | 5.00 | [IL18, JAK2, NF2, STAT3, VEGFA] |
| GO:0048144 | fibroblast proliferation | GO_BP | 8.15E-06 | 1.43E-05 | 5.81 | 5.00 | [CCNA2, CDK4, ESR1, GSTP1, MYC] |
| KEGG:04932 | Non-alcoholic fatty liver disease | KEGG | 8.42E-06 | 1.47E-05 | 4.00 | 6.00 | [AKT1, CASP3, CASP7, GSK3B, MAPK8, PIK3CA] |
| GO:0046209 | nitric oxide metabolic process | GO_BP | 8.63E-06 | 1.50E-05 | 5.75 | 5.00 | [AKT1, IL10, JAK2, MTOR, NOS3] |
| GO:0032885 | regulation of polysaccharide biosynthetic process | GO_BP | 8.82E-06 | 1.52E-05 | 9.76 | 4.00 | [AKT1, EGF, GSK3B, MTOR] |
| GO:2001057 | reactive nitrogen species metabolic process | GO_BP | 9.12E-06 | 1.56E-05 | 5.68 | 5.00 | [AKT1, IL10, JAK2, MTOR, NOS3] |
| GO:0010822 | positive regulation of mitochondrion organization | GO_BP | 9.64E-06 | 1.64E-05 | 5.62 | 5.00 | [BCL2, GSK3B, MAPK8, MMP9, PYCARD] |
| GO:0007260 | tyrosine phosphorylation of STAT protein | GO_BP | 9.64E-06 | 1.64E-05 | 5.62 | 5.00 | [IL18, JAK2, NF2, STAT3, VEGFA] |
| GO:0071312 | cellular response to alkaloid | GO_BP | 9.73E-06 | 1.64E-05 | 9.52 | 4.00 | [BCL2L1, CASP3, CASP7, CCNA2] |
| GO:0060416 | response to growth hormone | GO_BP | 9.73E-06 | 1.64E-05 | 9.52 | 4.00 | [AKT1, JAK2, MYC, STAT3] |
| GO:0016572 | histone phosphorylation | GO_BP | 9.73E-06 | 1.64E-05 | 9.52 | 4.00 | [CCNA2, CDK1, CDK2, JAK2] |
| GO:0071392 | cellular response to estradiol stimulus | GO_BP | 1.07E-05 | 1.79E-05 | 9.30 | 4.00 | [CCNA2, EGFR, ESR1, IL10] |
| GO:0045740 | positive regulation of DNA replication | GO_BP | 1.07E-05 | 1.79E-05 | 9.30 | 4.00 | [CDK1, EGF, EGFR, PCNA] |
| GO:0071276 | cellular response to cadmium ion | GO_BP | 1.17E-05 | 1.96E-05 | 9.09 | 4.00 | [AKT1, EGFR, MAPK8, MMP9] |
| GO:0006112 | energy reserve metabolic process | GO_BP | 1.33E-05 | 2.20E-05 | 5.26 | 5.00 | [ADRB3, AKT1, GSK3B, MTOR, MYC] |
| GO:0032091 | negative regulation of protein binding | GO_BP | 1.47E-05 | 2.42E-05 | 5.15 | 5.00 | [ADRB3, AKT1, GSK3B, IL10, MAPK8] |
| GO:1901184 | regulation of ERBB signaling pathway | GO_BP | 1.54E-05 | 2.52E-05 | 5.10 | 5.00 | [AKT1, EGF, EGFR, ERBB2, MMP9] |
| GO:0036473 | cell death in response to oxidative stress | GO_BP | 1.54E-05 | 2.52E-05 | 5.10 | 5.00 | [AKT1, BCL2, IL10, JAK2, PPIA] |
| GO:1901216 | positive regulation of neuron death | GO_BP | 1.62E-05 | 2.63E-05 | 5.05 | 5.00 | [CASP3, CASP9, GSK3B, MTOR, NQO2] |
| GO:0032881 | regulation of polysaccharide metabolic process | GO_BP | 1.67E-05 | 2.69E-05 | 8.33 | 4.00 | [AKT1, EGF, GSK3B, MTOR] |
| GO:0035173 | histone kinase activity | GO_BP | 1.70E-05 | 2.72E-05 | 18.75 | 3.00 | [CDK1, CDK2, JAK2] |
| GO:0070303 | negative regulation of stress-activated protein kinase signaling cascade | GO_BP | 1.81E-05 | 2.89E-05 | 8.16 | 4.00 | [AKT1, GSTP1, MYC, PPIA] |
| GO:0032873 | negative regulation of stress-activated MAPK cascade | GO_BP | 1.81E-05 | 2.89E-05 | 8.16 | 4.00 | [AKT1, GSTP1, MYC, PPIA] |
| GO:0014068 | positive regulation of phosphatidylinositol 3-kinase signaling | GO_BP | 1.87E-05 | 2.97E-05 | 4.90 | 5.00 | [EGF, ERBB3, IL18, JAK2, PIK3CA] |
| GO:0014013 | regulation of gliogenesis | GO_BP | 1.96E-05 | 3.08E-05 | 4.85 | 5.00 | [BMP2, EGFR, MTOR, MYC, NF2] |
| GO:0008631 | intrinsic apoptotic signaling pathway in response to oxidative stress | GO_BP | 1.96E-05 | 3.09E-05 | 8.00 | 4.00 | [AKT1, BCL2, JAK2, PPIA] |
| GO:1901655 | cellular response to ketone | GO_BP | 2.06E-05 | 3.19E-05 | 4.81 | 5.00 | [AKT1, CASP9, CDK4, EGFR, MYC] |
| GO:0061702 | inflammasome complex | GO_CC | 2.06E-05 | 3.20E-05 | 17.65 | 3.00 | [CASP1, NLRP3, PYCARD] |
| GO:0046622 | positive regulation of organ growth | GO_BP | 2.13E-05 | 3.27E-05 | 7.84 | 4.00 | [AKT1, CDK1, MTOR, PIM1] |
| GO:1901989 | positive regulation of cell cycle phase transition | GO_BP | 2.16E-05 | 3.30E-05 | 4.76 | 5.00 | [AKT1, CCND1, CDK1, CDK4, EGFR] |
| KEGG:04928 | Parathyroid hormone synthesis, secretion and action | KEGG | 2.26E-05 | 3.43E-05 | 4.72 | 5.00 | [BCL2, EGFR, MMP13, PDE4B, PDE4D] |
| GO:0014009 | glial cell proliferation | GO_BP | 2.30E-05 | 3.47E-05 | 7.69 | 4.00 | [EGFR, MTOR, MYC, NF2] |
| KEGG:04659 | Th17 cell differentiation | KEGG | 2.36E-05 | 3.55E-05 | 4.67 | 5.00 | [CD4, JAK2, MAPK8, MTOR, STAT3] |
| GO:0008593 | regulation of Notch signaling pathway | GO_BP | 2.36E-05 | 3.55E-05 | 4.67 | 5.00 | [AKT1, EGF, EGFR, NOS3, STAT3] |
| GO:0048146 | positive regulation of fibroblast proliferation | GO_BP | 2.48E-05 | 3.66E-05 | 7.55 | 4.00 | [CCNA2, CDK4, ESR1, MYC] |
| GO:0045058 | T cell selection | GO_BP | 2.48E-05 | 3.66E-05 | 7.55 | 4.00 | [BCL2, CD4, MTOR, STAT3] |
| GO:2001243 | negative regulation of intrinsic apoptotic signaling pathway | GO_BP | 2.47E-05 | 3.67E-05 | 4.63 | 5.00 | [AKT1, BCL2, BCL2L1, MMP9, PPIA] |
| GO:0060252 | positive regulation of glial cell proliferation | GO_BP | 2.46E-05 | 3.68E-05 | 16.67 | 3.00 | [EGFR, MTOR, MYC] |
| GO:0033273 | response to vitamin | GO_BP | 2.58E-05 | 3.80E-05 | 4.59 | 5.00 | [CCND1, EGFR, GSTP1, PIM1, TYR] |
| GO:0035590 | purinergic nucleotide receptor signaling pathway | GO_BP | 2.67E-05 | 3.90E-05 | 7.41 | 4.00 | [CASP1, IL18, NLRP3, PYCARD] |
| GO:0045931 | positive regulation of mitotic cell cycle | GO_BP | 2.82E-05 | 4.10E-05 | 4.50 | 5.00 | [AKT1, CCND1, CDK1, CDK4, EGFR] |
| GO:0046632 | alpha-beta T cell differentiation | GO_BP | 2.95E-05 | 4.25E-05 | 4.46 | 5.00 | [BCL2, IL18, MTOR, NLRP3, STAT3] |
| GO:2001237 | negative regulation of extrinsic apoptotic signaling pathway | GO_BP | 3.21E-05 | 4.61E-05 | 4.39 | 5.00 | [AKT1, BCL2L1, GSK3B, GSTP1, NOS3] |
| GO:0002286 | T cell activation involved in immune response | GO_BP | 3.34E-05 | 4.78E-05 | 4.35 | 5.00 | [IL18, ITGAL, MTOR, NLRP3, STAT3] |
| GO:0055119 | relaxation of cardiac muscle | GO_BP | 3.42E-05 | 4.86E-05 | 15.00 | 3.00 | [PDE4B, PDE4D, PIK3CA] |
| GO:0032649 | regulation of interferon-gamma production | GO_BP | 3.49E-05 | 4.93E-05 | 4.31 | 5.00 | [IL10, IL18, PDE4B, PDE4D, PYCARD] |
| GO:0032609 | interferon-gamma production | GO_BP | 3.49E-05 | 4.93E-05 | 4.31 | 5.00 | [IL10, IL18, PDE4B, PDE4D, PYCARD] |
| GO:0006275 | regulation of DNA replication | GO_BP | 3.79E-05 | 5.32E-05 | 4.24 | 5.00 | [CCNA2, CDK1, EGF, EGFR, PCNA] |
| KEGG:04722 | Neurotrophin signaling pathway | KEGG | 3.94E-05 | 5.51E-05 | 4.20 | 5.00 | [AKT1, BCL2, GSK3B, MAPK8, PIK3CA] |
| KEGG:04071 | Sphingolipid signaling pathway | KEGG | 3.94E-05 | 5.51E-05 | 4.20 | 5.00 | [AKT1, BCL2, MAPK8, NOS3, PIK3CA] |
| KEGG:05416 | Viral myocarditis | KEGG | 4.06E-05 | 5.64E-05 | 6.67 | 4.00 | [CASP3, CASP9, CCND1, ITGAL] |
| GO:0003179 | heart valve morphogenesis | GO_BP | 4.06E-05 | 5.64E-05 | 6.67 | 4.00 | [BMP2, CCNA2, MTOR, NOS3] |
| KEGG:04152 | AMPK signaling pathway | KEGG | 4.10E-05 | 5.67E-05 | 4.17 | 5.00 | [AKT1, CCNA2, CCND1, MTOR, PIK3CA] |
| GO:0043388 | positive regulation of DNA binding | GO_BP | 4.33E-05 | 5.96E-05 | 6.56 | 4.00 | [EGF, JAK2, MMP9, MYC] |
| GO:0045428 | regulation of nitric oxide biosynthetic process | GO_BP | 4.62E-05 | 6.32E-05 | 6.45 | 4.00 | [AKT1, IL10, JAK2, MTOR] |
| GO:0031295 | T cell costimulation | GO_BP | 4.62E-05 | 6.32E-05 | 6.45 | 4.00 | [AKT1, CD5, DPP4, PIK3CA] |
| GO:0004517 | nitric-oxide synthase activity | GO_MF | 4.62E-05 | 6.32E-05 | 6.45 | 4.00 | [AKT1, EGFR, ESR1, NOS3] |
| GO:0001658 | branching involved in ureteric bud morphogenesis | GO_BP | 4.62E-05 | 6.32E-05 | 6.45 | 4.00 | [BCL2, BMP2, MYC, VEGFA] |
| GO:0051101 | regulation of DNA binding | GO_BP | 4.99E-05 | 6.79E-05 | 4.00 | 5.00 | [EGF, JAK2, MAPK8, MMP9, MYC] |
| GO:0080164 | regulation of nitric oxide metabolic process | GO_BP | 5.24E-05 | 7.09E-05 | 6.25 | 4.00 | [AKT1, IL10, JAK2, MTOR] |
| GO:0031294 | lymphocyte costimulation | GO_BP | 5.24E-05 | 7.09E-05 | 6.25 | 4.00 | [AKT1, CD5, DPP4, PIK3CA] |
| GO:0002363 | alpha-beta T cell lineage commitment | GO_BP | 6.02E-05 | 8.11E-05 | 12.50 | 3.00 | [BCL2, MTOR, STAT3] |
| GO:0014855 | striated muscle cell proliferation | GO_BP | 6.27E-05 | 8.41E-05 | 5.97 | 4.00 | [CDK1, JAK2, PIM1, STAT3] |
| GO:0014015 | positive regulation of gliogenesis | GO_BP | 6.27E-05 | 8.41E-05 | 5.97 | 4.00 | [BMP2, EGFR, MTOR, MYC] |
| GO:0040014 | regulation of multicellular organism growth | GO_BP | 6.65E-05 | 8.87E-05 | 5.88 | 4.00 | [BCL2, CDK4, PIK3CA, STAT3] |
| GO:0002294 | CD4-positive, alpha-beta T cell differentiation involved in immune response | GO_BP | 6.65E-05 | 8.87E-05 | 5.88 | 4.00 | [IL18, MTOR, NLRP3, STAT3] |
| GO:1903798 | regulation of production of miRNAs involved in gene silencing by miRNA | GO_BP | 6.83E-05 | 9.06E-05 | 12.00 | 3.00 | [EGFR, ESR1, STAT3] |
| GO:0090201 | negative regulation of release of cytochrome c from mitochondria | GO_BP | 6.83E-05 | 9.06E-05 | 12.00 | 3.00 | [AKT1, BCL2L1, NLRP3] |
| GO:0071880 | adenylate cyclase-activating adrenergic receptor signaling pathway | GO_BP | 6.83E-05 | 9.06E-05 | 12.00 | 3.00 | [ADRB3, PDE4B, PDE4D] |
| GO:0070920 | regulation of production of small RNA involved in gene silencing by RNA | GO_BP | 6.83E-05 | 9.06E-05 | 12.00 | 3.00 | [EGFR, ESR1, STAT3] |
| GO:0043369 | CD4-positive or CD8-positive, alpha-beta T cell lineage commitment | GO_BP | 6.83E-05 | 9.06E-05 | 12.00 | 3.00 | [BCL2, MTOR, STAT3] |
| GO:0060675 | ureteric bud morphogenesis | GO_BP | 7.04E-05 | 9.29E-05 | 5.80 | 4.00 | [BCL2, BMP2, MYC, VEGFA] |
| GO:0031571 | mitotic G1 DNA damage checkpoint signaling | GO_BP | 7.04E-05 | 9.29E-05 | 5.80 | 4.00 | [CCND1, CDK1, CDK2, PCNA] |
| GO:0002293 | alpha-beta T cell differentiation involved in immune response | GO_BP | 7.04E-05 | 9.29E-05 | 5.80 | 4.00 | [IL18, MTOR, NLRP3, STAT3] |
| GO:0002287 | alpha-beta T cell activation involved in immune response | GO_BP | 7.04E-05 | 9.29E-05 | 5.80 | 4.00 | [IL18, MTOR, NLRP3, STAT3] |
| GO:0044819 | mitotic G1/S transition checkpoint signaling | GO_BP | 7.45E-05 | 9.78E-05 | 5.71 | 4.00 | [CCND1, CDK1, CDK2, PCNA] |
| GO:0003170 | heart valve development | GO_BP | 7.45E-05 | 9.78E-05 | 5.71 | 4.00 | [BMP2, CCNA2, MTOR, NOS3] |
| GO:0072171 | mesonephric tubule morphogenesis | GO_BP | 7.88E-05 | 1.03E-04 | 5.63 | 4.00 | [BCL2, BMP2, MYC, VEGFA] |
| GO:0010518 | positive regulation of phospholipase activity | GO_BP | 7.88E-05 | 1.03E-04 | 5.63 | 4.00 | [ANG, CCNA2, EGFR, ESR1] |
| GO:0042531 | positive regulation of tyrosine phosphorylation of STAT protein | GO_BP | 8.32E-05 | 1.08E-04 | 5.56 | 4.00 | [IL18, JAK2, STAT3, VEGFA] |
| GO:1900373 | positive regulation of purine nucleotide biosynthetic process | GO_BP | 8.64E-05 | 1.12E-04 | 11.11 | 3.00 | [MYC, NOS3, STAT3] |
| GO:0044321 | response to leptin | GO_BP | 8.64E-05 | 1.12E-04 | 11.11 | 3.00 | [CCNA2, CCND1, STAT3] |
| GO:0033688 | regulation of osteoblast proliferation | GO_BP | 8.64E-05 | 1.12E-04 | 11.11 | 3.00 | [BCL2, BMP2, CCNA2] |
| GO:0032928 | regulation of superoxide anion generation | GO_BP | 8.64E-05 | 1.12E-04 | 11.11 | 3.00 | [AKT1, EGFR, GSTP1] |
| GO:0030810 | positive regulation of nucleotide biosynthetic process | GO_BP | 8.64E-05 | 1.12E-04 | 11.11 | 3.00 | [MYC, NOS3, STAT3] |
| GO:0010971 | positive regulation of G2/M transition of mitotic cell cycle | GO_BP | 8.64E-05 | 1.12E-04 | 11.11 | 3.00 | [CCND1, CDK1, CDK4] |
| GO:0071378 | cellular response to growth hormone stimulus | GO_BP | 9.66E-05 | 1.24E-04 | 10.71 | 3.00 | [JAK2, MYC, STAT3] |
| GO:0002292 | T cell differentiation involved in immune response | GO_BP | 9.76E-05 | 1.25E-04 | 5.33 | 4.00 | [IL18, MTOR, NLRP3, STAT3] |
| GO:0042698 | ovulation cycle | GO_BP | 1.08E-04 | 1.38E-04 | 5.19 | 4.00 | [CASP3, EGFR, ESR1, PCNA] |
| GO:0032729 | positive regulation of interferon-gamma production | GO_BP | 1.08E-04 | 1.38E-04 | 5.19 | 4.00 | [IL18, PDE4B, PDE4D, PYCARD] |
| GO:0072078 | nephron tubule morphogenesis | GO_BP | 1.20E-04 | 1.51E-04 | 5.06 | 4.00 | [BCL2, BMP2, MYC, VEGFA] |
| GO:0061180 | mammary gland epithelium development | GO_BP | 1.20E-04 | 1.51E-04 | 5.06 | 4.00 | [AKT1, CCND1, ESR1, JAK2] |
| GO:1902751 | positive regulation of cell cycle G2/M phase transition | GO_BP | 1.19E-04 | 1.51E-04 | 10.00 | 3.00 | [CCND1, CDK1, CDK4] |
| GO:0071875 | adrenergic receptor signaling pathway | GO_BP | 1.19E-04 | 1.51E-04 | 10.00 | 3.00 | [ADRB3, PDE4B, PDE4D] |
| GO:0072088 | nephron epithelium morphogenesis | GO_BP | 1.32E-04 | 1.65E-04 | 4.94 | 4.00 | [BCL2, BMP2, MYC, VEGFA] |
| GO:0060260 | regulation of transcription initiation from RNA polymerase II promoter | GO_BP | 1.32E-04 | 1.65E-04 | 4.94 | 4.00 | [CCND1, CDK1, CDK4, ESR1] |
| GO:0006801 | superoxide metabolic process | GO_BP | 1.32E-04 | 1.65E-04 | 4.94 | 4.00 | [AKT1, EGFR, GSTP1, NOS3] |
| GO:0030332 | cyclin binding | GO_MF | 1.32E-04 | 1.65E-04 | 9.68 | 3.00 | [CDK1, CDK2, CDK4] |
| GO:0002360 | T cell lineage commitment | GO_BP | 1.32E-04 | 1.65E-04 | 9.68 | 3.00 | [BCL2, MTOR, STAT3] |
| GO:0030669 | clathrin-coated endocytic vesicle membrane | GO_CC | 1.38E-04 | 1.72E-04 | 4.88 | 4.00 | [CD4, EGF, EGFR, NLRP3] |
| GO:0001618 | virus receptor activity | GO_BP | 1.38E-04 | 1.72E-04 | 4.88 | 4.00 | [CD4, CDK1, DPP4, EGFR] |
| GO:0061333 | renal tubule morphogenesis | GO_BP | 1.45E-04 | 1.79E-04 | 4.82 | 4.00 | [BCL2, BMP2, MYC, VEGFA] |
| GO:0060193 | positive regulation of lipase activity | GO_BP | 1.45E-04 | 1.79E-04 | 4.82 | 4.00 | [ANG, CCNA2, EGFR, ESR1] |
| GO:0072028 | nephron morphogenesis | GO_BP | 1.52E-04 | 1.87E-04 | 4.76 | 4.00 | [BCL2, BMP2, MYC, VEGFA] |
| GO:0010517 | regulation of phospholipase activity | GO_BP | 1.52E-04 | 1.87E-04 | 4.76 | 4.00 | [ANG, CCNA2, EGFR, ESR1] |
| GO:0060251 | regulation of glial cell proliferation | GO_BP | 1.59E-04 | 1.94E-04 | 9.09 | 3.00 | [EGFR, MTOR, MYC] |
| GO:0045737 | positive regulation of cyclin-dependent protein serine/threonine kinase activity | GO_BP | 1.59E-04 | 1.94E-04 | 9.09 | 3.00 | [AKT1, CCND1, EGFR] |
| GO:0010962 | regulation of glucan biosynthetic process | GO_BP | 1.59E-04 | 1.94E-04 | 9.09 | 3.00 | [AKT1, GSK3B, MTOR] |
| GO:0005979 | regulation of glycogen biosynthetic process | GO_BP | 1.59E-04 | 1.94E-04 | 9.09 | 3.00 | [AKT1, GSK3B, MTOR] |
| GO:0043367 | CD4-positive, alpha-beta T cell differentiation | GO_BP | 1.59E-04 | 1.95E-04 | 4.71 | 4.00 | [IL18, MTOR, NLRP3, STAT3] |
| GO:0007422 | peripheral nervous system development | GO_BP | 1.59E-04 | 1.95E-04 | 4.71 | 4.00 | [AKT1, CDK1, ERBB2, ERBB3] |
| GO:0031397 | negative regulation of protein ubiquitination | GO_BP | 1.66E-04 | 2.02E-04 | 4.65 | 4.00 | [AKT1, HDAC8, MTOR, PPIA] |
| GO:1900087 | positive regulation of G1/S transition of mitotic cell cycle | GO_BP | 1.74E-04 | 2.10E-04 | 8.82 | 3.00 | [AKT1, CCND1, EGFR] |
| GO:0090322 | regulation of superoxide metabolic process | GO_BP | 1.74E-04 | 2.10E-04 | 8.82 | 3.00 | [AKT1, EGFR, GSTP1] |
| GO:0055023 | positive regulation of cardiac muscle tissue growth | GO_BP | 1.74E-04 | 2.10E-04 | 8.82 | 3.00 | [CDK1, MTOR, PIM1] |
| GO:0033687 | osteoblast proliferation | GO_BP | 1.74E-04 | 2.10E-04 | 8.82 | 3.00 | [BCL2, BMP2, CCNA2] |
| GO:0010039 | response to iron ion | GO_BP | 1.74E-04 | 2.10E-04 | 8.82 | 3.00 | [BCL2, CCND1, MAP1LC3A] |
| GO:2000142 | regulation of DNA-templated transcription, initiation | GO_BP | 1.82E-04 | 2.18E-04 | 4.55 | 4.00 | [CCND1, CDK1, CDK4, ESR1] |
| GO:0071260 | cellular response to mechanical stimulus | GO_BP | 1.82E-04 | 2.18E-04 | 4.55 | 4.00 | [AKT1, CASP1, EGFR, MAPK8] |
| GO:0000271 | polysaccharide biosynthetic process | GO_BP | 1.82E-04 | 2.18E-04 | 4.55 | 4.00 | [AKT1, EGF, GSK3B, MTOR] |
| GO:0016242 | negative regulation of macroautophagy | GO_BP | 1.90E-04 | 2.28E-04 | 8.57 | 3.00 | [AKT1, MTOR, PIK3CA] |
| GO:0004407 | histone deacetylase activity | GO_BP | 1.90E-04 | 2.28E-04 | 8.57 | 3.00 | [HDAC8, MAPK8, VEGFA] |
| GO:1901030 | positive regulation of mitochondrial outer membrane permeabilization involved in apoptotic signaling pathway | GO_BP | 2.07E-04 | 2.45E-04 | 8.33 | 3.00 | [BCL2, GSK3B, MAPK8] |
| GO:0090075 | relaxation of muscle | GO_BP | 2.07E-04 | 2.45E-04 | 8.33 | 3.00 | [PDE4B, PDE4D, PIK3CA] |
| GO:0045746 | negative regulation of Notch signaling pathway | GO_BP | 2.07E-04 | 2.45E-04 | 8.33 | 3.00 | [AKT1, EGF, EGFR] |
| GO:0033558 | protein deacetylase activity | GO_BP | 2.07E-04 | 2.45E-04 | 8.33 | 3.00 | [HDAC8, MAPK8, VEGFA] |
| GO:0032743 | positive regulation of interleukin-2 production | GO_BP | 2.07E-04 | 2.45E-04 | 8.33 | 3.00 | [CD4, PDE4B, PDE4D] |
| GO:0032692 | negative regulation of interleukin-1 production | GO_BP | 2.07E-04 | 2.45E-04 | 8.33 | 3.00 | [GSTP1, IL10, NLRP3] |
| GO:0043154 | negative regulation of cysteine-type endopeptidase activity involved in apoptotic process | GO_BP | 2.07E-04 | 2.46E-04 | 4.40 | 4.00 | [AKT1, MMP9, NLRP3, VEGFA] |
| GO:0042058 | regulation of epidermal growth factor receptor signaling pathway | GO_BP | 2.07E-04 | 2.46E-04 | 4.40 | 4.00 | [AKT1, EGF, EGFR, MMP9] |
| GO:0046889 | positive regulation of lipid biosynthetic process | GO_BP | 2.15E-04 | 2.55E-04 | 4.35 | 4.00 | [AKT1, CCNA2, MTOR, NLRP3] |
| GO:1904031 | positive regulation of cyclin-dependent protein kinase activity | GO_BP | 2.24E-04 | 2.64E-04 | 8.11 | 3.00 | [AKT1, CCND1, EGFR] |
| GO:0042092 | type 2 immune response | GO_BP | 2.24E-04 | 2.64E-04 | 8.11 | 3.00 | [IL10, IL18, NLRP3] |
| GO:0030224 | monocyte differentiation | GO_BP | 2.24E-04 | 2.64E-04 | 8.11 | 3.00 | [CD4, MYC, VEGFA] |
| GO:0060421 | positive regulation of heart growth | GO_BP | 2.43E-04 | 2.85E-04 | 7.89 | 3.00 | [CDK1, MTOR, PIM1] |
| GO:0045907 | positive regulation of vasoconstriction | GO_BP | 2.43E-04 | 2.85E-04 | 7.89 | 3.00 | [AKT1, EGFR, NLRP3] |
| GO:1903321 | negative regulation of protein modification by small protein conjugation or removal | GO_BP | 2.64E-04 | 3.08E-04 | 4.12 | 4.00 | [AKT1, HDAC8, MTOR, PPIA] |
| GO:0070664 | negative regulation of leukocyte proliferation | GO_BP | 2.64E-04 | 3.08E-04 | 4.12 | 4.00 | [CASP3, ERBB2, GSTP1, IL10] |
| GO:0070873 | regulation of glycogen metabolic process | GO_BP | 2.83E-04 | 3.29E-04 | 7.50 | 3.00 | [AKT1, GSK3B, MTOR] |
| GO:0043368 | positive T cell selection | GO_BP | 2.83E-04 | 3.29E-04 | 7.50 | 3.00 | [BCL2, MTOR, STAT3] |
| GO:0032642 | regulation of chemokine production | GO_BP | 2.85E-04 | 3.30E-04 | 4.04 | 4.00 | [GSTP1, IL10, IL18, PYCARD] |
| GO:0032602 | chemokine production | GO_BP | 2.85E-04 | 3.30E-04 | 4.04 | 4.00 | [GSTP1, IL10, IL18, PYCARD] |
| GO:0006919 | activation of cysteine-type endopeptidase activity involved in apoptotic process | GO_BP | 2.85E-04 | 3.30E-04 | 4.04 | 4.00 | [CASP1, CASP9, JAK2, PYCARD] |
| GO:0045742 | positive regulation of epidermal growth factor receptor signaling pathway | GO_BP | 3.05E-04 | 3.51E-04 | 7.32 | 3.00 | [AKT1, EGF, MMP9] |
| GO:0043028 | cysteine-type endopeptidase regulator activity involved in apoptotic process | GO_BP | 3.05E-04 | 3.51E-04 | 7.32 | 3.00 | [CASP1, NLRP3, PYCARD] |
| GO:1902895 | positive regulation of pri-miRNA transcription by RNA polymerase II | GO_BP | 3.28E-04 | 3.76E-04 | 7.14 | 3.00 | [BMP2, IL10, STAT3] |
| GO:0014037 | Schwann cell differentiation | GO_BP | 3.28E-04 | 3.76E-04 | 7.14 | 3.00 | [AKT1, CDK1, ERBB3] |
| GO:1901186 | positive regulation of ERBB signaling pathway | GO_BP | 3.52E-04 | 4.01E-04 | 6.98 | 3.00 | [AKT1, EGF, MMP9] |
| GO:0045429 | positive regulation of nitric oxide biosynthetic process | GO_BP | 3.52E-04 | 4.01E-04 | 6.98 | 3.00 | [AKT1, JAK2, MTOR] |
| GO:0042554 | superoxide anion generation | GO_BP | 3.52E-04 | 4.01E-04 | 6.98 | 3.00 | [AKT1, EGFR, GSTP1] |
| GO:1904407 | positive regulation of nitric oxide metabolic process | GO_BP | 3.77E-04 | 4.27E-04 | 6.82 | 3.00 | [AKT1, JAK2, MTOR] |
| GO:0030212 | hyaluronan metabolic process | GO_BP | 4.02E-04 | 4.55E-04 | 6.67 | 3.00 | [AKT1, EGF, PIM1] |
| KEGG:04930 | Type II diabetes mellitus | KEGG | 4.30E-04 | 4.84E-04 | 6.52 | 3.00 | [MAPK8, MTOR, PIK3CA] |
| GO:1901028 | regulation of mitochondrial outer membrane permeabilization involved in apoptotic signaling pathway | GO_BP | 4.30E-04 | 4.84E-04 | 6.52 | 3.00 | [BCL2, GSK3B, MAPK8] |
| GO:0046006 | regulation of activated T cell proliferation | GO_BP | 4.30E-04 | 4.84E-04 | 6.52 | 3.00 | [CASP3, IL18, PYCARD] |
| GO:0030857 | negative regulation of epithelial cell differentiation | GO_BP | 4.30E-04 | 4.84E-04 | 6.52 | 3.00 | [CCND1, MMP9, VEGFA] |
| GO:0010463 | mesenchymal cell proliferation | GO_BP | 4.30E-04 | 4.84E-04 | 6.52 | 3.00 | [BMP2, MYC, VEGFA] |
| GO:1902808 | positive regulation of cell cycle G1/S phase transition | GO_BP | 4.58E-04 | 5.13E-04 | 6.38 | 3.00 | [AKT1, CCND1, EGFR] |
| GO:0043124 | negative regulation of I-kappaB kinase/NF-kappaB signaling | GO_BP | 4.58E-04 | 5.13E-04 | 6.38 | 3.00 | [ESR1, GSTP1, PYCARD] |
| GO:2000273 | positive regulation of signaling receptor activity | GO_BP | 4.87E-04 | 5.44E-04 | 6.25 | 3.00 | [EGF, IL10, JAK2] |
| GO:0071548 | response to dexamethasone | GO_BP | 4.87E-04 | 5.44E-04 | 6.25 | 3.00 | [CASP9, EGFR, PCNA] |
| GO:1900371 | regulation of purine nucleotide biosynthetic process | GO_BP | 5.18E-04 | 5.75E-04 | 6.12 | 3.00 | [MYC, NOS3, STAT3] |
| GO:0060711 | labyrinthine layer development | GO_BP | 5.18E-04 | 5.75E-04 | 6.12 | 3.00 | [AKT1, CCNA2, IL10] |
| KEGG:05144 | Malaria | KEGG | 5.50E-04 | 6.08E-04 | 6.00 | 3.00 | [IL10, IL18, ITGAL] |
| GO:0035307 | positive regulation of protein dephosphorylation | GO_BP | 5.50E-04 | 6.08E-04 | 6.00 | 3.00 | [JAK2, MTOR, PPIA] |
| GO:0030808 | regulation of nucleotide biosynthetic process | GO_BP | 5.50E-04 | 6.08E-04 | 6.00 | 3.00 | [MYC, NOS3, STAT3] |
| GO:0030225 | macrophage differentiation | GO_BP | 5.50E-04 | 6.08E-04 | 6.00 | 3.00 | [CD4, MMP9, VEGFA] |
| GO:0050798 | activated T cell proliferation | GO_BP | 5.83E-04 | 6.42E-04 | 5.88 | 3.00 | [CASP3, IL18, PYCARD] |
| GO:0048662 | negative regulation of smooth muscle cell proliferation | GO_BP | 5.83E-04 | 6.42E-04 | 5.88 | 3.00 | [ANG, GSTP1, IL10] |
| GO:0043457 | regulation of cellular respiration | GO_BP | 5.83E-04 | 6.42E-04 | 5.88 | 3.00 | [AKT1, MYC, PIK3CA] |
| GO:0016538 | cyclin-dependent protein serine/threonine kinase regulator activity | GO_BP | 5.83E-04 | 6.42E-04 | 5.88 | 3.00 | [CASP3, CCNA2, CCND1] |
| GO:0010863 | positive regulation of phospholipase C activity | GO_BP | 5.83E-04 | 6.42E-04 | 5.88 | 3.00 | [ANG, EGFR, ESR1] |
| GO:1900544 | positive regulation of purine nucleotide metabolic process | GO_BP | 6.17E-04 | 6.77E-04 | 5.77 | 3.00 | [MYC, NOS3, STAT3] |
| GO:0045981 | positive regulation of nucleotide metabolic process | GO_BP | 6.17E-04 | 6.77E-04 | 5.77 | 3.00 | [MYC, NOS3, STAT3] |
| GO:0019213 | deacetylase activity | GO_MF | 6.17E-04 | 6.77E-04 | 5.77 | 3.00 | [HDAC8, MAPK8, VEGFA] |
| GO:0009250 | glucan biosynthetic process | GO_BP | 6.17E-04 | 6.77E-04 | 5.77 | 3.00 | [AKT1, GSK3B, MTOR] |
| GO:0005978 | glycogen biosynthetic process | GO_BP | 6.17E-04 | 6.77E-04 | 5.77 | 3.00 | [AKT1, GSK3B, MTOR] |
| GO:1903202 | negative regulation of oxidative stress-induced cell death | GO_BP | 6.52E-04 | 7.13E-04 | 5.66 | 3.00 | [AKT1, IL10, PPIA] |
| GO:1900274 | regulation of phospholipase C activity | GO_BP | 6.52E-04 | 7.13E-04 | 5.66 | 3.00 | [ANG, EGFR, ESR1] |
| GO:0035196 | production of miRNAs involved in gene silencing by miRNA | GO_BP | 6.52E-04 | 7.13E-04 | 5.66 | 3.00 | [EGFR, ESR1, STAT3] |
| GO:0035094 | response to nicotine | GO_BP | 6.52E-04 | 7.13E-04 | 5.66 | 3.00 | [BCL2, CASP3, NLRP3] |
| GO:0004623 | phospholipase A2 activity | GO_MF | 6.52E-04 | 7.13E-04 | 5.66 | 3.00 | [ANG, CASP3, EGFR] |
| GO:1904645 | response to amyloid-beta | GO_BP | 6.89E-04 | 7.50E-04 | 5.56 | 3.00 | [GSK3B, MMP13, MMP9] |
| GO:1901185 | negative regulation of ERBB signaling pathway | GO_BP | 6.89E-04 | 7.50E-04 | 5.56 | 3.00 | [EGF, EGFR, ERBB2] |
| GO:0010823 | negative regulation of mitochondrion organization | GO_BP | 6.89E-04 | 7.50E-04 | 5.56 | 3.00 | [AKT1, BCL2L1, NLRP3] |
| GO:2000378 | negative regulation of reactive oxygen species metabolic process | GO_BP | 7.27E-04 | 7.88E-04 | 5.45 | 3.00 | [AKT1, BCL2, STAT3] |
| GO:0050999 | regulation of nitric-oxide synthase activity | GO_BP | 7.27E-04 | 7.88E-04 | 5.45 | 3.00 | [AKT1, EGFR, NOS3] |
| GO:0003044 | regulation of systemic arterial blood pressure mediated by a chemical signal | GO_BP | 7.27E-04 | 7.88E-04 | 5.45 | 3.00 | [ADRB3, NOS3, PDE4D] |
| GO:0002042 | cell migration involved in sprouting angiogenesis | GO_BP | 7.27E-04 | 7.88E-04 | 5.45 | 3.00 | [AKT1, EPHB4, VEGFA] |
| KEGG:04340 | Hedgehog signaling pathway | KEGG | 7.67E-04 | 8.27E-04 | 5.36 | 3.00 | [BCL2, CCND1, GSK3B] |
| GO:0070918 | production of small RNA involved in gene silencing by RNA | GO_BP | 7.67E-04 | 8.27E-04 | 5.36 | 3.00 | [EGFR, ESR1, STAT3] |
| GO:0035722 | interleukin-12-mediated signaling pathway | GO_BP | 7.67E-04 | 8.27E-04 | 5.36 | 3.00 | [IL10, JAK2, PPIA] |
| GO:0031050 | dsRNA processing | GO_BP | 7.67E-04 | 8.27E-04 | 5.36 | 3.00 | [EGFR, ESR1, STAT3] |
| KEGG:04923 | Regulation of lipolysis in adipocytes | KEGG | 8.08E-04 | 8.68E-04 | 5.26 | 3.00 | [ADRB3, AKT1, PIK3CA] |
| KEGG:00480 | Glutathione metabolism | KEGG | 8.08E-04 | 8.68E-04 | 5.26 | 3.00 | [GSTP1, RRM1, RRM2] |
| GO:1902893 | regulation of pri-miRNA transcription by RNA polymerase II | GO_BP | 8.08E-04 | 8.68E-04 | 5.26 | 3.00 | [BMP2, IL10, STAT3] |
| GO:0097345 | mitochondrial outer membrane permeabilization | GO_BP | 8.08E-04 | 8.68E-04 | 5.26 | 3.00 | [BCL2, GSK3B, MAPK8] |
| GO:0061614 | pri-miRNA transcription by RNA polymerase II | GO_BP | 8.08E-04 | 8.68E-04 | 5.26 | 3.00 | [BMP2, IL10, STAT3] |
| GO:0072132 | mesenchyme morphogenesis | GO_BP | 8.50E-04 | 9.09E-04 | 5.17 | 3.00 | [BMP2, MYC, NOS3] |
| GO:0071349 | cellular response to interleukin-12 | GO_BP | 8.50E-04 | 9.09E-04 | 5.17 | 3.00 | [IL10, JAK2, PPIA] |
| GO:0033574 | response to testosterone | GO_BP | 8.50E-04 | 9.09E-04 | 5.17 | 3.00 | [CDK4, MYC, NLRP3] |
| GO:0070671 | response to interleukin-12 | GO_BP | 8.93E-04 | 9.52E-04 | 5.08 | 3.00 | [IL10, JAK2, PPIA] |
| GO:0043525 | positive regulation of neuron apoptotic process | GO_BP | 8.93E-04 | 9.52E-04 | 5.08 | 3.00 | [CASP3, CASP9, NQO2] |
| GO:0010332 | response to gamma radiation | GO_BP | 8.93E-04 | 9.52E-04 | 5.08 | 3.00 | [BCL2, BCL2L1, MYC] |
| GO:0009187 | cyclic nucleotide metabolic process | GO_BP | 8.93E-04 | 9.52E-04 | 5.08 | 3.00 | [NOS3, PDE4B, PDE4D] |
| GO:1902110 | positive regulation of mitochondrial membrane permeability involved in apoptotic process | GO_BP | 9.38E-04 | 9.95E-04 | 5.00 | 3.00 | [BCL2, GSK3B, MAPK8] |
| GO:0043536 | positive regulation of blood vessel endothelial cell migration | GO_BP | 9.38E-04 | 9.95E-04 | 5.00 | 3.00 | [AKT1, NOS3, VEGFA] |
